# Taxonomic revision of the Andean genus *Xenophyllum* (Compositae, Senecioneae)

**DOI:** 10.3897/phytokeys.158.50848

**Published:** 2020-09-03

**Authors:** Joel Calvo, Andrés Moreira-Muñoz

**Affiliations:** 1 Instituto de Geografía, Facultad de Ciencias del Mar y Geografía, Pontificia Universidad Católica de Valparaíso, Avenida Brasil 2241, 2362807 Valparaíso, Chile Pontificia Universidad Católica de Valparaíso Valparaíso Chile

**Keywords:** Andes, Asteraceae, Neotropics, nomenclature, taxonomy, typification, *
Werneria
*

## Abstract

The Andean genus *Xenophyllum* (Compositae, Senecioneae) is distributed along the high-Andes from northeastern Colombia to northern Chile and northwestern Argentina, mainly thriving in the paramo and puna ecoregions. It comprises suffruticose plants forming dense mats, hummocks, or clumps of erect stems. They are characterized by displaying involucral bracts fused at the base, supplementary bracts absent, and mostly radiate capitula with white ray corollas, seldom yellow or pink (disciform in one species). Traditionally, *Xenophyllum* species were treated as members of the genus *Werneria*, a morphologically close genus that includes rosettiform or scapiform perennial herbs. As currently circumscribed, *Xenophyllum* mostly differs from *Werneria* in having elongate stems. Herein, the first modern and comprehensive revision of the genus recognizing twenty-two species and two subspecies is presented. *Werneria decumbens* is synonymized with *X. weddellii*, as well as *X. fontii* with *X. humile* and *X. oscartovarii* with *X. dactylophyllum*. Likewise, four varietal names and two sectional names are proposed as new synonyms. Seven names are lectotypified, the name *X. sotarense* is epitypified, *W. decumbens* neotypified, and the supraspecific name W. sect. Integrifoliae Rockh. is typified. The combination X. crassum subsp. orientale**comb. nov.** is made. Descriptions and distribution maps are provided for all accepted species, in addition to an identification key. Ten species are illustrated, three of them for the first time.

## Introduction

**Figure 1.**
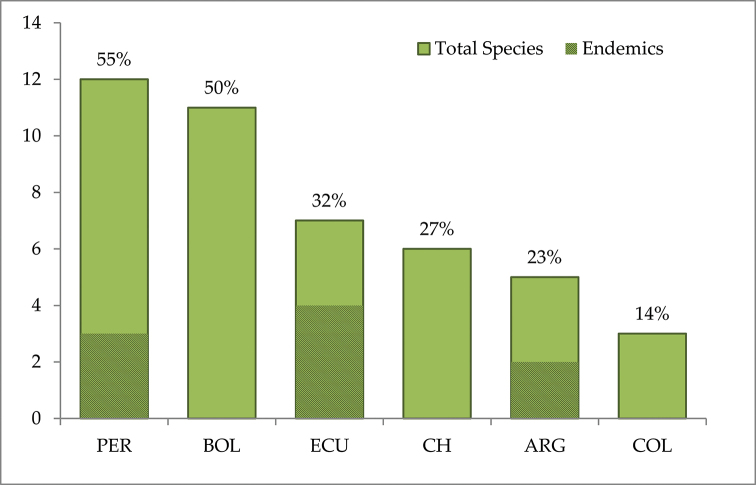
Number of *Xenophyllum* species per country – The percentage of endemic species is shaded. Country abbreviations are explained in Material and methods.

*Xenophyllum* V.A.Funk (Compositae, Senecioneae), is a genus of some 22 species distributed along the highlands of the Andes from northeastern Colombia to northwestern Argentina and northern Chile. The genus extends in distribution from the Colombian department of Norte de Santander (paramo del Almorzadero, ca. 7°N) to La Rioja Province in northwestern Argentina (Sierra de Famatina, ca. 29°S). Peru and Bolivia harbor the highest species diversity (12 and 11 species, respectively), followed by Ecuador (7 spp.), Chile (6 spp.), Argentina (5 spp.), and Colombia (3 spp.). The largest number of endemic species is found in Ecuador, 4 of the 7 species (57%) in the country (Fig. 1). Species of *Xenophyllum* are mostly small suffruticose plants characterized by displaying involucral bracts fused at the base, supplementary bracts absent, radiate capitula (disciform in one species), usually white ray corollas (yellow or pink in a few species), balusterform filament collars, obtuse or auriculate anther bases, truncate style branches with a crown of sweeping trichomes or penicillate, and glabrous or white-villous achenes. Two main habits can be differentiated according to the type of growth form (1) species forming dense mats or hummocks; (2) species forming clumps of somewhat distantly spaced stems or even having a shrubby habit (Calvo and Funk 2020).

Species of the genus were traditionally treated under the morphologically similar genus *Werneria* Kunth until the end of 20^th^ century, when Funk (1997a) coined the genus *Xenophyllum*. This new genus was meant to segregate from *Werneria* those species forming loose or tightly compressed hummocks or well-developed mats, and accordingly, the genus *Werneria* was re-circumscribed to accommodate the rosettiform species growing solitary or in small clumps. However, the type of growth form is not unequivocal for segregating both genera because some species of *Werneria* can also form compressed mats (e.g., *W. aretioides* Wedd., *W. weberbaueriana* Rockh.). We did not find any diagnostic morphological synapomorphies to support either of the two genera, but the presence or absence of elongate stems allows placing most species in one genus or the other. On this basis, *Werneria* comprises rosettiform or scapiform perennial herbs while *Xenophyllum* includes suffruticose species. Although phylogenetic studies are required to elucidate their evolutionary relationships, Funk (1997a) pointed out that these groups are probably not monophyletic. For the time being, and in pursuit of pragmatism, the revision of both groups has been presented separately following Funk’s proposal. Indeed, her treatment was adopted in all subsequent regional catalogues and floras of the Andean countries (Nordenstam 1999; Jørgensen 2014; Freire and Ariza-Espinar 2014; Ávila et al. 2016).

The taxonomic history of the group before the establishment of the genus *Xenophyllum* is detailed in Calvo et al. (in press). After the original publication of *Xenophyllum* in 1997, the taxonomic contributions into the understanding of the genus were limited to a few new combinations (Cabrera and Freire 1999; Calvo et al. 2017), a synopsis of the Peruvian species (Beltrán 2016), and the description of new species (Linares et al. 2010; Calvo and Funk 2020). For this reason, we present the first modern and comprehensive revision of the genus *Xenophyllum*. It includes updated nomenclature, identification keys, detailed descriptions, distributions maps, and drawings.

## Material and methods

This study is mostly based on the revision of 1014 herbarium specimens (types included, duplicates excluded) kept at the following herbaria: A, AAU, B, BA, BM, BOLV, BR, C, CAS, CAUP, COL, CONC, CUVC, F, GB, GH, GOET, HA, HSB, HSP, HUSA, HUTPL, K, LE, LIL, LOJA, LPB, MA, MERL, MO, NY, P, Q, QCA, QCNE, QPLS, RB, SGO, UC, US, USM, and W. Additionally, digital herbarium specimens or supplementary information were obtained from BC, CORD, CUZ, E, ICESI, LL, LP, S, SI, TCD, and UDBC; herbarium acronyms follow Thiers (2020). Field work was also conducted in southern Colombia, Ecuador, southern Peru, Bolivia, and northern Chile, which allowed us to study 13 out of the 22 species in the field. The collections were mainly kept at BOLV, CAUP, CONC, and SGO.

A comprehensive synonymy of the genus *Xenophyllum* was compiled. Types of all accepted names and synonyms were studied. However, we did not locate original material of Werneria dactylophylla var. glanduloso-denticulata Rockh. This appears in Unverified names and we suggest its application based on the opinion of previous authors.

A general description of the genus and detailed species descriptions were prepared. For this, qualitative characters were studied with the aid of a binocular dissecting microscope, while quantitative characters were recorded using a Mitutoyo digital caliper, CD-15DC. A Zeiss Standard 16WL microscope was used for examination of the achene trichomes. Information concerning the habitat, elevation, and flowering period of each species was obtained from the herbarium specimen labels.

Accepted species are presented in an order that represents their morphological affinities. An index to the taxonomic names treated is provided in Appendix I. Likewise, all studied exsiccatae can be found in Appendix II. The information of the examined specimens that do not correspond to type material is detailed at the end of each species. They are consecutively listed in alphabetical order by country, primary political division, and collector surname. When herbarium specimens from a political division have not been studied but the presence of the respective species is expected there, the name of that political division is included and marked accordingly in the Distribution and habitat section. The maps were generated using QGIS 3.4 Madeira. The country abbreviations used in the distribution maps are: ARG (Argentina), BOL (Bolivia), BR (Brazil), CH (Chile), COL (Colombia), ECU (Ecuador), and PER (Peru).

## Morphology

**Figure 2.**
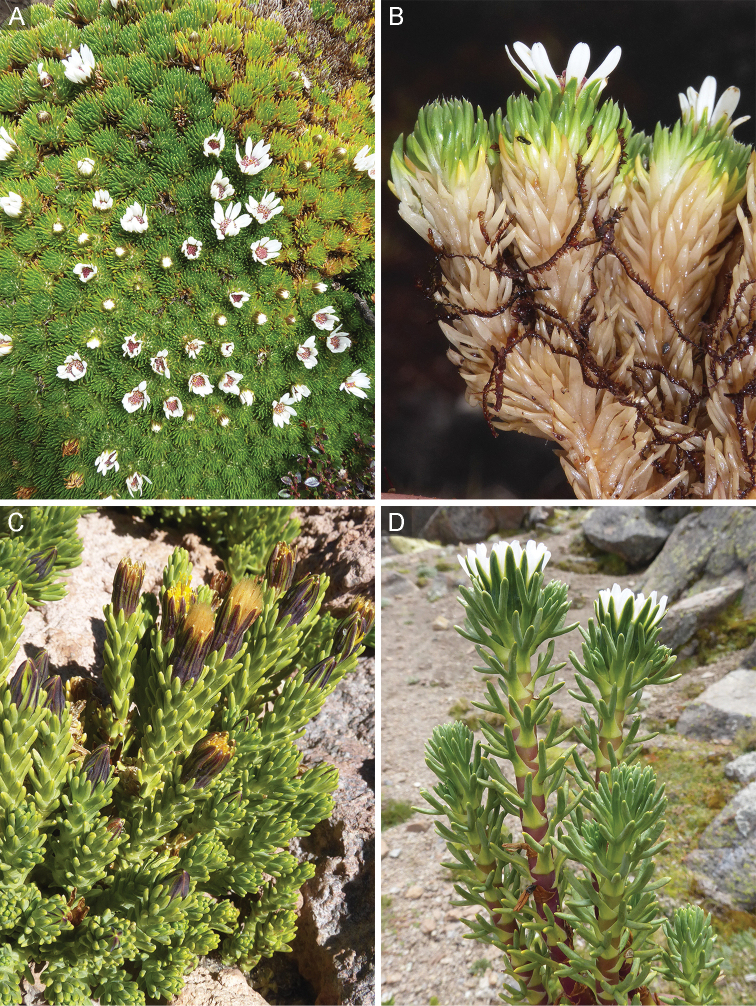
Main habit types in *Xenophyllum***A, B** species forming dense mats or hummocks **C, D** species forming clumps of somewhat distantly spaced stems **A***X. humile* (Ecuador, Pichincha, pr. Papallacta) **B***X. sotarense* (Colombia, Cauca, Sotará Volcano) **C***X. juniperinum* (Chile, Antofagasta, Aucanquilcha Volcano) **D***X. digitatum* (Bolivia, Potosí, cordillera Kari Kari). Pictures by Joel Calvo.

**Figure 3.**
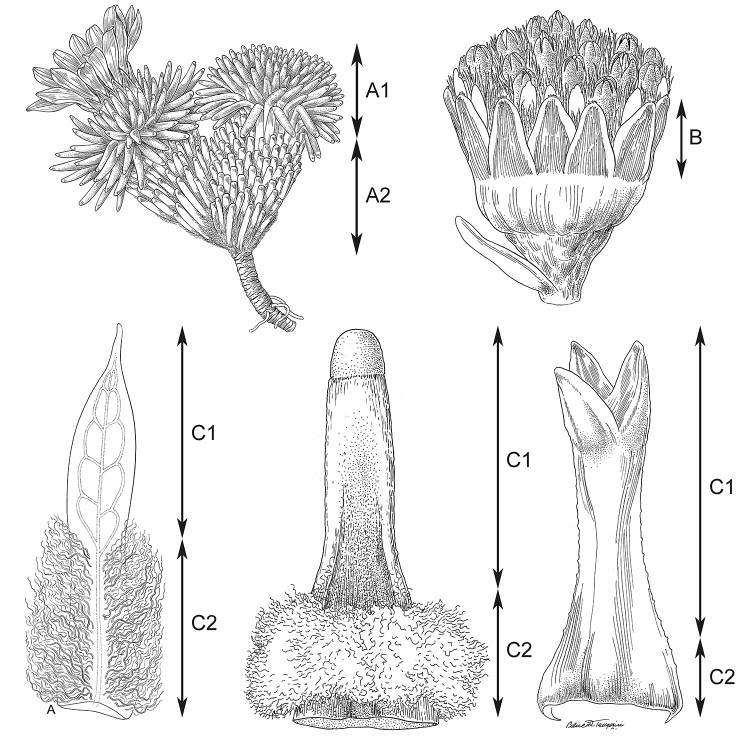
Diagram of measurements used in descriptions **A1** aerial photosynthetic part of the stem (referred to as aerial part in the descriptions of the species forming dense mats or hummocks) **A2** rhizome-like stem of the species forming dense mats or hummocks **B** free part of the involucral bracts (notice the typically fused base) **C1** leaf lamina **C2** sheath-like leaf base. Details taken from Alice Tangerini’s illustrations.

**Habit.** Species of the genus *Xenophyllum* are small suffruticose plants with two main habits according to the type of growth form. One comprises the species forming dense mats or hummocks (Fig. 2A, B), which exhibit the morphology and growth typical of cushion plants. They consist of a compact mass of closely spaced stems with reduced apical dominance. Underneath the living distal stem, the leaves wither and remain attached to the stem (marcescent leaves) or fall off and only the leaf base remnants persist (Fig. 2B). This nonphotosynthetic stem part resembles a rhizome, hence it is here referred to as rhizome-like stem and is described separately from the aerial photosynthetic part (Fig. 3A).

In contrast, the second habit type includes the species forming clumps of somewhat distantly spaced stems that are uniform in appearance along its whole length (Fig. 2C, D). Among these species, *X. staffordiae* (Sandwith) V.A.Funk can be considered shrub as the stems reach a height of ca. 60 cm.

**Leaves.** The leaves are alternate, simple, usually subimbricate to stellate-imbricate (spirally arranged), and are extended into a sheath-like base that is more or less noticeable depending on the amount of indumentum at the leaf attachment. The leaf length provided in the text strictly corresponds to the leaf lamina (sheath-like base excluded, Fig. 3C). The shape of the leaf lamina can be linear, triangular, or spatulate. A single species, *X. esquilachense* (Cuatrec.) V.A.Funk, has somewhat distantly arranged leaves that lack sheath-like base and are abruptly narrowed at the base.

The leaf morphology has a relevant taxonomic value within the genus [see treatment under *Werneria* by Blake (1928)]. Leaf division is useful to distinguish two sets of species (1) 12 species with undivided or entire leaves, which are linear (rarely triangular), truncate to aristate at the apex, and terete (e.g., *X. acerosum* (Cuatrec.) V.A.Funk) or rather flat in cross section (e.g., *X. marcidum* (S.F.Blake) V.A.Funk); and (2) 11 species having the leaf apex divided (*X. poposa* (Phil.) V.A.Funk being the single species included in both sets because of its leaf dimorphism). Within this latter set, in turn, three subsets can easily be recognized according to the division type: species with notched leaf apex (shallowly 2 to 3-divided; e.g., *X. incisum* (Phil.) V.A.Funk), forked leaf apex (deeply 2 to 3-divided; e.g., *X. digitatum* (Wedd.) V.A.Funk), or finger-like leaf apex (with multiple projections or lobes; e.g., *X. dactylophyllum* (Sch.Bip.) V.A.Funk). The leaf margin, on the other hand, can be entire (e.g., *X. weddellii* (Phil.) V.A.Funk), entire and ciliate (e.g., *X. decorum* (S.F.Blake) V.A.Funk), entire and distantly scabrous-ciliate (i.e., *X. esquilachense*), or denticulate (e.g., *X. ciliolatum* (A.Gray) V.A.Funk).

Species of *Xenophyllum* are largely glabrous with an exception of a few species that can have floccose-lanate indumentum (e.g., *X. staffordiae*) or evanescent arachnoid trichomes (e.g., *X. amblydactylum* (S.F.Blake) V.A.Funk). The presence of indumentum is very variable at specific level, and therefore, it is not useful for distinguishing species.

**Capitula.** All species have radiate and erect capitula except for *X. esquilachense* that displays disciform capitula and *X. staffordiae* that has radiate capitula rather nodding. Among the radiate species, most taxa have ray corollas conspicuously surpassing the involucre (e.g., *X. marcidum*). Three species, however, are characterized by their ray corollas not surpassing the involucre, i.e., *X. ciliolatum*, *X. juniperinum* (Hieron.) J.Calvo, and *X. staffordiae*. When the ray corollas are well-developed, this character is very noticeable.

The ray florets are pistillate and fertile. The color of the ray corollas is usually white, sometimes slightly purplish on the abaxial surface near the apex. Three species have yellow or pale yellow ray corollas and *X. roseum* (Hieron.) V.A.Funk is the single species in the genus with pink ray corollas. The corolla limb has 2 to 7 veins (sometimes unconspicuous) and it is subentire to 3-toothed at the apex.

The disc florets are hermaphroditic. The corollas are tubular, 5-lobed at the apex, yellow, whitish, creamy, or purplish.

**Involucres.** The involucres are cupuliform or narrowly cupuliform. They lack supplementary bracts (calyculus) and the involucral bracts are typically fused at the base. The number of involucral bracts, although quite constant within each species, is not very useful for distinguishing purposes because of the significant overlap among several species. Rather useful is the involucral bract length; the provided measurements strictly refer to its free part (Fig. 3B). Likewise, it has to be clarified that the involucre length corresponds to the distance from the apex of the involucral bracts to the peduncle attachment point. The involucre was found to be a defining character of the genus.

**Floral microcharacters.** Species of *Xenophyllum* have balusterform filament collars, obtuse or auriculate anther bases, and anther apical appendages 2–4-times longer than wide.

Further variability has been found in the morphology of the style branch apex. In all species examined it is truncate but differs on its ornamentation; some species bear a crown of sweeping trichomes (e.g., *X. juniperinum*, *X. sotarense* (Hieron.) V.A.Funk) whereas others display a tuft with scanty or many longer trichomes, i.e., penicillate (e.g., *X. digitatum*, *X. rosenii* (R.E.Fr.) V.A.Funk). We found that this character presents intraspecific variability (e.g., *X. dactylophyllum*, *X. rigidum* (Kunth) V.A.Funk), hence it has not been considered valuable for taxonomic purposes. Salomón et al. (2016) stated similar conclusions after studying the floral microcharacters in 72 South American *Senecio* species and several species from other Senecioneae genera. They noticed transitional states in trichome length, making difficult the difference between the typical crown of short trichomes and the apices with a tuft of elongate trichomes.

**Achenes.** The achenes are homomorphic, cylindrical, 6 to 9-ribbed, mostly glabrous except in the Ecuadorian endemic species *X. funkianum* J.Calvo, *X. rigidum*, and *X. roseum* that are white-villous. The achenes of *X. acerosum*, another species endemic to Ecuador, are described as having scattered arachnoid trichomes but such observation is based on the study of immature achenes, and therefore, mature achenes are required for confirming it. An accurate description of the achene size could not be provided for this latter species and for a few other species due to a lack of collections with mature achenes.

In *X. funkianum* and *X. rigidum*, the achene indumentum is composed of twin filiform trichomes, not myxogenic, with acute to subacute, asymmetrical, slightly forked or not apex; see Calvo and Funk (2020, fig. 2D).

The pappus of all species is capillary, composed of barbellate, whitish to partially rose- or purple-colored bristles.

## Taxonomic treatment

### Xenophyllum

Taxon classificationPlantaeAsteralesAsteraceae

V.A.Funk, Novon 7(3): 235. 1997.

C73A8F9A-0533-55F7-ACC8-F7F508E62D7A

Werneria subg. Euwerneria Rockh., Bot. Jahrb. Syst. 70: 285. 1939, *pro parte*, *nom. inval.* (Turland et al. 2018, ICN Art. 21.3, 22.2, and 38.1).
Werneria sect. Digitifoliae Rockh., Bot. Jahrb. Syst. 70: 276, 285. 1939, syn. nov. Type: Werneria digitata Wedd. [≡ Xenophyllum digitatum (Wedd.) V.A.Funk] (Turland et al. 2018, ICN Art. 10.8).
Werneria sect. Aciculares Rockh., Bot. Jahrb. Syst. 70: 277, 291. 1939, syn. nov. Type: Werneria humilis Kunth [≡ Xenophyllum humile (Kunth) V.A.Funk], designated here.

#### Type.

*Xenophyllum dactylophyllum* (Sch.Bip.) V.A.Funk.

#### Description.

Suffruticose plants, forming mats, hummocks, or clumps of erect stems. ***Rhizomes*** 4–10 × 0.1–1 cm, horizontal to oblique, glabrous; rhizome-like stems (when present) up to 35 cm long covered with matted lanate, arachnoid, or pilose indumentum and old leaves or leaf base remnants, rather erect, simple or branched from the base. ***Stems*** 1–20(–60) cm long, simple or branched, glabrous, sparsely pilose, arachnoid, or lanate. ***Leaves*** alternate, simple, subimbricate, imbricate, or stellate-imbricate (rarely somewhat distantly arranged), extending into a sheath-like base that is glabrous or bears arachnoid or long silky trichomes (abruptly narrowed at the base in one species); leaf laminas 2.5–27.6 × 0.5–6.7 mm, linear, triangular, or spatulate, aristate, acute, obtuse, notched, forked, or finger-like at the apex, entire, denticulate, shortly ciliate, or scabrous-ciliate at the margin, flat to terete, sometimes slightly curved forwards in cross section, glabrous (rarely floccose-lanate), 1-nerved above (sometimes barely visible or unconspicuous), 1-nerved beneath (sometimes barely visible or unconspicuous), usually fleshy, matte or shiny (sometimes papillose). ***Capitula*** radiate (disciform in one species), solitary, terminal, erect (rarely somewhat nodding), sessile to subsessile (rarely shortly pedunculate). ***Involucres*** 3.3–19.7 × 2.7–15.8 mm, cupuliform or narrowly cupuliform, with bracts fused at the base, glabrous; involucral bracts 8 to 21, 1.1–14.9 × 0.7–3.9 mm, linear-oblong to subulate, acute to obtuse at the apex, greenish to dark-purplish; supplementary bracts absent; receptacle epaleaceous, rather plane, smooth or seldom alveolate. ***Ray florets*** 8 to 39, pistillate, fertile; corollas 3.6–19.7 × 0.4–3.4 mm, 2 to 7-veined (sometimes unconspicuous), subentire to 3-toothed at the apex, surpassing or not the involucre, white, yellow, or pink (disciform species with peripheral florets having corollas reduced to a vestigial tube or without corolla). ***Disc florets*** 7 to 95, hermaphroditic; corollas 3.1–10.6 mm long, 5-lobed, yellow, whitish, creamy, or purplish; filament collars balusterform; anther bases obtuse or auriculate; anther appendages 2–4-times longer than wide; style branches truncate with a crown of sweeping trichomes or penicillate, yellowish to purplish. ***Achenes*** 1.6–5.4 × 0.4–1.3 mm, cylindrical, 6 to 9-ribbed, glabrous or white-villous (with scattered arachnoid trichomes in one species); pappus capillary, 1 to 2-seriate, composed of bristles 2.1–19 mm long, barbellate, whitish to partially rose- or purple-colored. Chromosome number 2*n* = 104–108(± 4) (Diers 1961).

#### Distribution and habitat.

**Figure 4.**
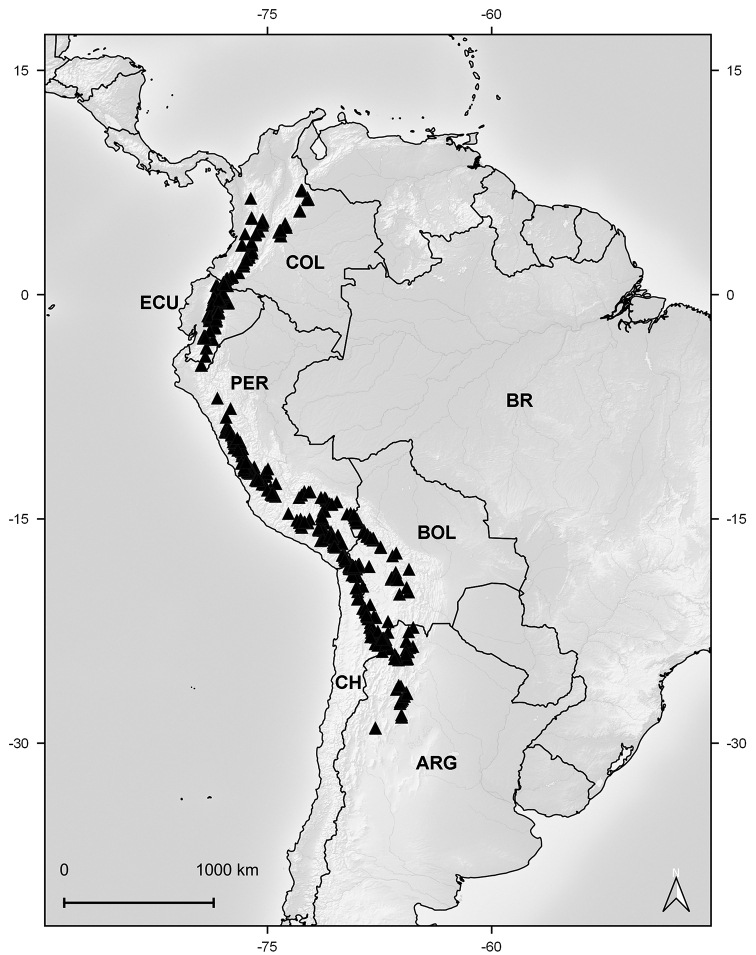
Distribution map of genus *Xenophyllum*.

Colombia, Ecuador, Peru, Bolivia, northern Chile, and northwestern Argentina. The *Xenophyllum* species thrive in the paramo and puna ecoregions, between elevations of (2600–)3000–5500 m (Fig. 4).

#### Etymology.

The generic name *Xenophyllum* means strange leaves (“xénos”: strange, alien, foreign; “phyll-”: relating to leaves).

### Key to the species of *Xenophyllum*

**Table:** 

1	Leaves entire, undivided at the apex	**2**
–	Leaves notched, forked, or finger-like at the apex	**13**
2	Ray corollas not surpassing the involucre, yellow	**3**
–	Ray corollas conspicuously surpassing the involucre, white or pink	**4**
3	Leaves linear, denticulate, erect (not adpressed to the stem); involucral bracts 4.1–7.7 mm long; stems 4–7 cm long, erect or decumbent	**9. *X. ciliolatum***
–	Leaves linear-triangular, minutely, irregularly denticulate, adpressed to the stem; involucral bracts 2.8–3.4 mm long; stems 8–15 cm long, erect	**10. *X. juniperinum***
4	Ray corollas entirely pink	**4. *X. roseum***
–	Ray corollas white, sometimes purplish beneath	**5**
5	Leaf apices aristate (arista 0.5–1 mm long)	**6**
–	Leaf apices acute to obtuse	**7**
6	Plants forming distant, rather solitary, erect stems; involucres ca. 8.6 × 7.3 mm; ray corollas ca. 12 mm long; disc florets ca. 50	**1. *X. acerosum***
–	Plants forming dense mats or hummocks; involucres 4.9–5 × 4.8–5 mm; ray corollas 6.3–6.5 mm long; disc florets 14 to 15	**3. *X. sotarense***
7	Stems glabrous	**8**
–	Stems densely arachnoid	**9**
8	Leaves triangular, denticulate in the upper half; involucral bracts 13 to 15; ray florets 14 to 21 with corollas 7.4–12.9 mm long; disc florets 35 to 48	**8. *X. marcidum***
–	Leaves linear-subulate, entire; involucral bracts 8 to 12; ray florets 9 to 15 with corollas 5.9–8 mm long; disc florets 24 to 32	**11. *X. weddellii***
9	Achenes white-villous	**10**
–	Achenes glabrous	**11**
10	Leaf laminas 5.3–7.8 × 0.8–0.9 mm; ray florets 20 to 23; capitula surpassing the leaves	**5. *X. funkianum***
–	Leaf laminas 12.6–13.5 × 2.3–2.4 mm; ray florets 38 to 41; capitula enclosed among the leaves	**6. *X. rigidum***
11	Involucres 10.2–19.7 mm long; ray florets 13 to 21 with corollas 13–19.7 mm long; disc florets 50 to 95	**7. *X. crassum***
–	Involucres 3.3–9.3 mm long; ray florets 8 to 13 with corollas 3.6–12.2 mm long; disc florets 7 to 39	**12**
12	Leaf laminas spreading at nearly 90° from the sheath-like bases, 2.5–10.8 mm long, finely papillose; plants forming dense mats or hummocks (in paramo and humid puna)	**2. *X. humile***
–	Leaf laminas barely spreading from the sheath-like bases, 2.5–7.1 mm long, not papillose; plants forming clumps of erect stems, rarely dense mats (in subhumid, dry, and desertic puna)	**13. *X. poposa***
13	Capitula disciform	**22. *X. esquilachense***
–	Capitula radiate	**14**
14	Ray corollas not surpassing the involucre, pale yellow; capitula rather nodding; stems 30–60 cm tall	**21. *X. staffordiae***
–	Ray corollas conspicuously surpassing the involucre, white; capitula erect; stems 2–24 cm tall	**15**
15	Leaves 2 to 3-forked or finger-like at the apex	**16**
–	Leaves 3-notched at the apex	**19**
16	Leaves finger-like at the apex (at least 9-divided)	**20. *X. dactylophyllum***
–	Leaves forked (2 to 3-divided)	**17**
17	Leaves 2-forked	**15. *X. rosenii***
–	Leaves 3-forked	**18**
18	Leaf lobes 3.6–5.9 mm long, entire or divided; involucres 12.1–16.7 mm long; ray corollas 9.2–14.1 mm long; disc florets 49 to 63	**16. *X. digitatum***
–	Leaf lobes 1.3–2.6 mm long, entire (rarely notched); involucres 7–8.4 mm long; ray corollas 7.1–8.2 mm long; disc florets 29 to 33	**17. *X. pseudodigitatum***
19	Stems arachnoid; leaf laminas 0.5–1.1 mm wide; disc florets 7 to 18	**13. *X. poposa***
–	Stems glabrous; leaf laminas 1.3–3.7 mm wide; disc florets 22 to 58	**20**
20	Ray florets 20 to 39; disc florets 37 to 58; involucral bracts 5.2–8 mm long	**21**
–	Ray florets 8 to 13; disc florets 22 to 37; involucral bracts 3.1–5.4 mm long	**22**
21	Leaf margins entire and shortly ciliate; central lobe smaller than lateral ones; ray florets 20 to 21 with corollas 10.4–11.9 mm long; achenes 4.8–5.4 mm long	**18. *X. decorum***
–	Leaf margins entire; central lobe larger than lateral ones; ray florets 26 to 39 with corollas 8.3–9.2 mm long; achenes 2.5–3.1 mm long	**14. *X. lorochaqui***
22	Leaf laminas with evanescent arachnoid trichomes above; involucral bracts 10 to 13, 4.9–5.4 mm long; disc corollas 5.4–6 mm long	**19. *X. amblydactylum***
–	Leaf laminas glabrous; involucral bracts 8 to 9, 3.1–4.3 mm long; disc corollas 4.4–5 mm long	**12. *X. incisum***

### 1. Xenophyllum acerosum

Taxon classificationPlantaeAsteralesAsteraceae

(Cuatrec.) V.A.Funk, Novon 7(3): 238. 1997.

453B585F-2467-59CF-8D51-DE937BE09F7B

Werneria acerosa Cuatrec., Brittonia 8: 45. 1954. Type. Ecuador. Azuay: “Oriente” border, eastern Cordillera, between Oña and the río Yacuambi, 3050−3415 m, 10/19 Sep 1945, *F. Prieto 280* (holotype: F-1402673!; isotypes: G-00305681 (digital image!), GH s.n.!, K-000527742 (digital image!), MO-1652045!, NY s.n.!, P-02088566 (digital image!), S-R-6520 (digital image!), UC-986455!, US-00037296!, VEN-34483 (digital image!)).

#### Description.

**Figure 5.**
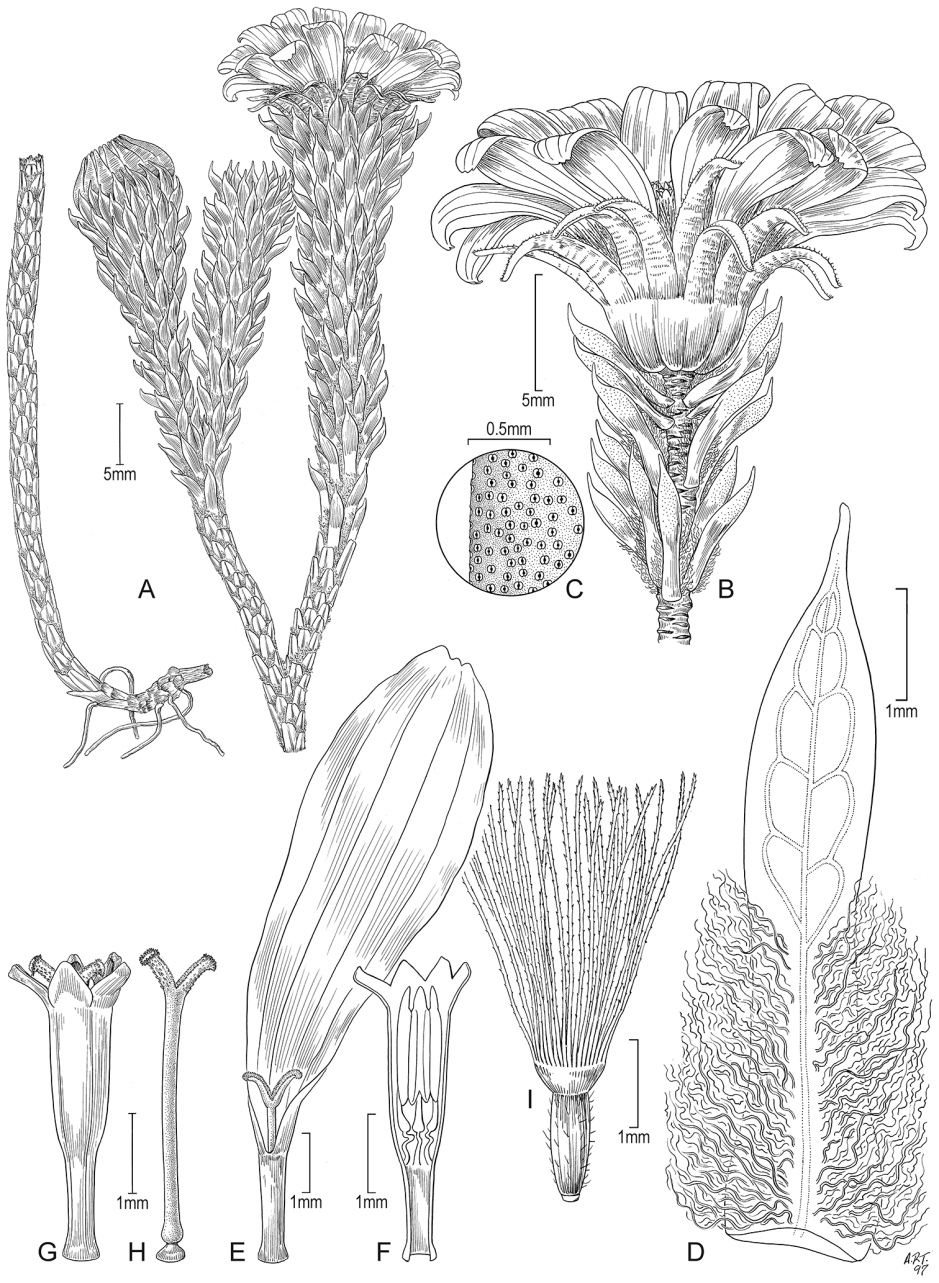
*Xenophyllum acerosum***A** habit **B** stem apical part and capitulum **C** detail of leaf surface **D** adaxial leaf surface **E** ray corolla and style **F** disc corolla and stamens (vertically sectioned, style removed) **G** disc corolla and style branches **H** style **I** immature achene with pappus. All details drawn from *Prieto 280* (US). Illustration by Alice Tangerini.

Suffruticose plant, forming distant, rather solitary, erect stems. ***Rhizomes*** 6–10 × 0.2–0.3 cm, horizontal to oblique, glabrous. ***Stems*** 12–20 cm tall, usually simple, sometimes branched at the upper part, covered with adpressed, arachnoid trichomes, with leaves restricted to the upper part. ***Leaves*** imbricate, extending into a sheath-like base that bears arachnoid trichomes; leaf laminas 4.6–5 × ca. 0.9 mm, linear-acicular, aristate at the apex (arista up to 1 mm long, purplish), entire, terete in cross section, glabrous, unconspicuously nerved on both faces, somewhat fleshy, drying coriaceous, matte, papillose. ***Capitula*** radiate, erect, sessile, partially enclosed among the leaves. ***Involucres*** ca. 8.6 × 7.3 mm, cupuliform; involucral bracts ca. 13, 5.2–5.5 × 1.1–1.6 mm, rather acute at the apex, dark-purplish. ***Ray florets*** ca. 13; corollas ca. 12 × 2.3–2.5 mm, 4 to 5-veined, subentire to 3-toothed at the apex, conspicuously surpassing the involucre, white, purplish beneath. ***Disc florets*** ca. 50; corollas 3.8–4.2 mm long, pale yellow to creamy; style branches truncate with a crown of sweeping trichomes, yellowish. ***Achenes*** cylindrical, with scattered arachnoid trichomes (immature); pappus 3.1–3.3 mm long, barbellate, whitish. Chromosome number unknown. Fig. 5.

#### Distribution and habitat.

**Figure 6.**
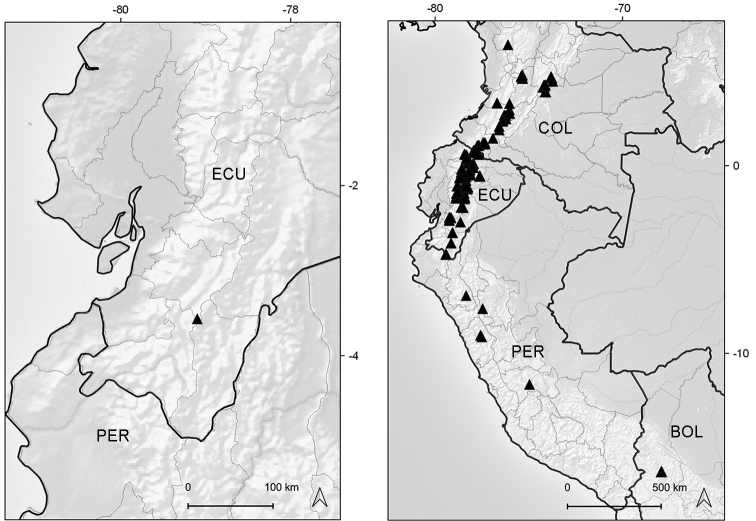
Distribution map of *Xenophyllum acerosum* (left hand) and *X. humile* (right hand).

Endemic to Ecuador (Azuay, Zamora-Chinchipe [expected]). It grows in marshy places of the paramo ecoregion, between elevations of 3050–3070 m (Fig. 6).

Thus far, *X. acerosum* is only known from the type locality in the paramo of Yacumbi, which is located above Saraguro on the border between the Ecuadorian provinces of Azuay and Zamora-Chinchipe.

#### Phenology.

Collected in flower in September and November.

#### Etymology.

The adjective *acerosus -a -um* means needle-shaped, and it refers to the leaf shape of this species.

#### Notes.

*Xenophyllum acerosum* is unique in the genus in its distant, rather solitary, erect stems arising from long rhizomes and by its linear-acicular, aristate leaves. The stems only bear leaves in the upper part.

#### Additional specimen examined.

**Ecuador. Azuay**: Cordillera Cordoncillo, along road from Babes (ca. 2 km E of Urdaneta) towards 28 de Mayo, 3°34'S, 79°6'W, 10 Nov 2018, *P. Sklenář 15820* (PRC n.v., QCA).

### 2. Xenophyllum humile

Taxon classificationPlantaeAsteralesAsteraceae

(Kunth) V.A.Funk, Novon 7(3): 239. 1997.

261DD160-B83E-50C6-A63E-83910513A605

Werneria humilis Kunth, Nov. Gen. Sp. [ed. fol.] 4: 150. 1818. Type. Ecuador. [“in summis Andibus Quitensium” according to the *ind. loc.*], [without date], *F.W.H.A. Humboldt & A.J.A. Bonpland s.n.* (lectotype: P-00320181 (digital image!), designated as “holotype” by Funk (1997a: 239); isolectotypes: B-W-16433-01-0 (digital image!), F-972259 (fragment! [almost nothing left]), P s.n.!).
Werneria humilis var. lindenii Wedd., Chlor. Andina 1: 82. 1856 [“Lindenii”]. Type. Colombia. Tolima: [nevado del] Tolima, limes inferior nivis perpetuae, [without date], *J. Goudot s.n.* (lectotype: P-02088543 (digital image!), designated here; isolectotype: P-02088544 (digital image!)), syn. nov.
Werneria humilis var. *β* Wedd., Chlor. Andina 1: 82. 1856, *nom. inval.* (Turland et al. 2018, ICN Art. 24.1 and 32.1c).
Werneria lehmannii Hieron., Bot. Jahrb. Syst. 28(5): 647. 1901 [“Lehmannii”]. *nom. illeg.* (Turland et al. 2018, ICN Art. 53.1), replaced name, non W. lehmannii Klatt 1894. Werneria articulata S.F.Blake, Contr. U.S. Natl. Herb. 22: 651. 1924, replacement name. Werneria humilis f. articulata (S.F.Blake) Rockh., Bot. Jahrb. Syst. 70: 294. 1939. Type. Ecuador. Imbabura/Pichincha: páramo de Mojanduleur [Mojanda], Otavalo, 3400−4000 m, [without date], *F.C. Lehmann 6230* (lectotype: K s.n.!, designated by Funk (1997a: 238); isolectotype: US-00037320 (right hand specimen, fragment!)).
Werneria humilis var. fontii Cuatrec., Trab. Mus. Nac. Ci. Nat., Ser. Bot. 27: cuadro 22. 1934 [“Fontii”], *nom. nud.* (Turland et al. 2018, ICN Art. 38.1).
Werneria fontii Cuatrec., Trab. Mus. Nac. Ci. Nat., Ser. Bot. 29: 42. 1935 [“Fontii”]. Xenophyllum fontii (Cuatrec.) V.A.Funk, Novon 7(3): 239. 1997. Type. Colombia. Tolima: vert. merid. mont. Tolima, San Juan del Agua, 4200 m, 15 May 1932, *J. Cuatrecasas 2862* (lectotype: MA-246359!, designated as “holotype” by Funk (1997a: 239); isolectotypes: F-844457!, K-000527741 (digital image!), MA-246359b!, MA-246359c!), syn. nov.
Oresigonia brevifolia Willd. ex Rockh., Bot. Jahrb. Syst. 70: 293. 1939, *nom. inval. pro syn.* (Turland et al. 2018, ICN Art. 36.1).
Werneria humilis var. angustifolia Cuatrec., *nom. nud. in sched.* (Turland et al. 2018, ICN Art. 38.1).

#### Description.

**Figure 7.**
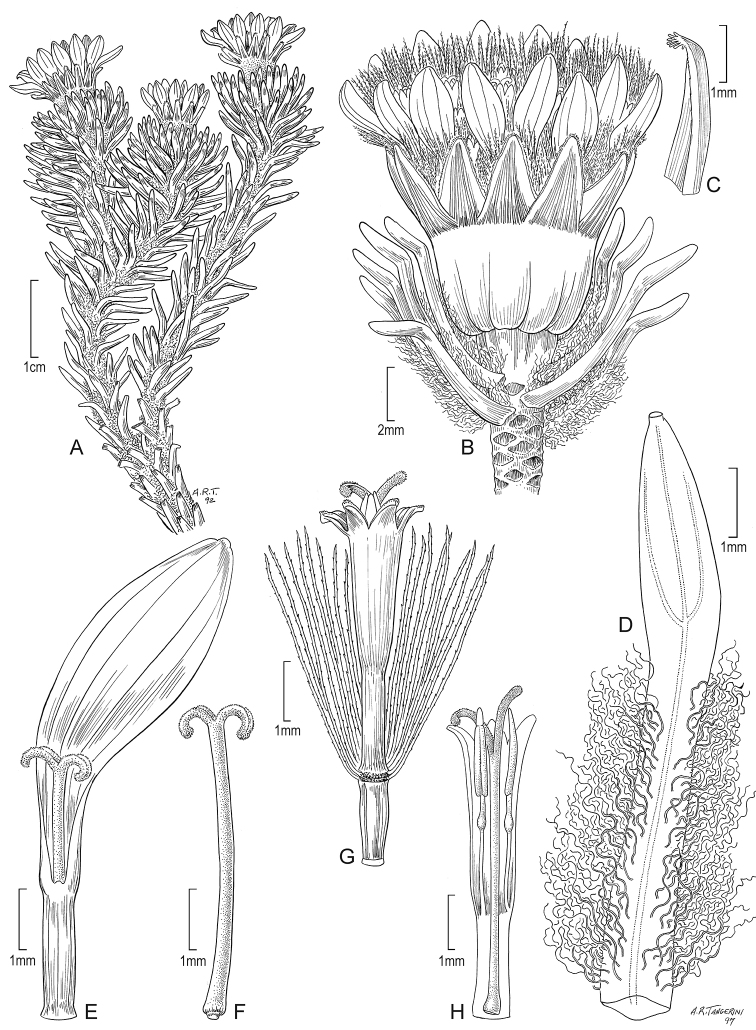
*Xenophyllum humile***A** habit **B** stem apical part and capitulum **C** detail of involucral bract apex **D** adaxial leaf surface **E** ray corolla and style **F** style **G** disc floret (frontward bristles removed) **H** disc corolla, stamens, and style (vertically sectioned). All details drawn from *Funk & Montezuma 11443* (US) except for **A** (drawn from *Øllgaard & Balslev 8749*, US) and **G, H** (drawn from *Funk 8093*, US). Illustration by Alice Tangerini.

Suffruticose plant, forming dense mats or hummocks, with rhizome-like stems up to 15 cm long covered with lanate indumentum and old leaves, rather erect, simple or branched from the base. ***Stems*** 1–3 cm tall (aerial part), lanate. ***Leaves*** stellate-imbricate to subimbricate, extending into a sheath-like base that bears long silky trichomes, usually with the leaf lamina spreading at nearly 90° from the sheath-like base; leaf laminas 2.5–10.8 × 0.5–1.5 mm, linear, obtuse to truncate, usually callous-like tipped at the apex, entire, elliptical to almost terete in cross section, glabrous, 1-nerved above (sometimes barely visible), 1-nerved beneath (sometimes barely visible), fleshy, drying coriaceous, matte, finely papillose. ***Capitula*** radiate, erect, sessile to subsessile (rarely with a short peduncle up to 5 mm long). ***Involucres*** 4.6–9.3 × 2.9–9 mm, cupuliform; involucral bracts (8–)10 to 13, 3.1–6.1 × 1–2.1 mm, obtuse at the apex, greenish to dark-purplish. ***Ray florets*** (8–)10 to 13; corollas 4.2–12.2 × 0.4–2.1 mm, 4 to 7-veined, subentire to 3-toothed at the apex, conspicuously surpassing the involucre, white. ***Disc florets*** (8–)22 to 39; corollas 3.1–5 mm long, whitish, usually purple-tipped; style branches truncate with a crown of sweeping trichomes, purplish. ***Achenes*** 2–2.8 × 0.4–0.6 mm, cylindrical, 7 to 9-ribbed, glabrous; pappus 2.1–7.9 mm long, barbellate, whitish, rarely partially rose-colored. Chromosome number unknown. Figs 2A, 7.

#### Additional iconography.

Funk (1997a: 237, fig. 1A); Funk (1997b: 33, fig. 3B, C); Beltrán (2016: 358, fig. 2C, as photo).

#### Distribution and habitat.

Northwestern Colombia to northern Bolivia. Bolivia (La Paz), Colombia (Antioquia, Caldas, Cauca, Cundinamarca, Huila, Meta, Nariño, Quindío [expected], Risaralda, Tolima, Valle del Cauca), Ecuador (Azuay, Bolívar, Cañar [expected], Carchi, Chimborazo, Cotopaxi, Imbabura, Loja, Morona-Santiago, Napo, Orellana, Pichincha, Sucumbíos, Tungurahua, Zamora-Chinchipe), Peru (Amazonas [n.v.], Ancash, Cajamarca, Junín, La Libertad [expected], San Martín). This species grows in marshes, grasslands, rocky outcrops, and exposed places of the paramo and humid puna ecoregions, between elevations of (2700–)3000–4800 m (Fig. 6).

The species is very common along the paramos of Colombia and Ecuador, but it becomes very scattered towards its southern limit. No collections have been studied from the region comprised between central Peru (Junín) and northern Bolivia (La Paz).

#### Phenology.

Flowering nearly all year round.

#### Etymology.

The epithet *humile* means low-growing, close to the ground.

#### Notes.

*Xenophyllum humile* (Kunth) V.A.Funk is a morphologically polymorphic species with rhizome-like stems covered with lanate indumentum and marcescent leaves, obtuse to truncate leaf apex (usually callous-like tipped), (8–)10 to 13 involucral bracts, and (8–)10 to 13 ray florets with white corollas. The leaves are usually bent backwards due to a sort of articulation at the inflexion point between the leaf lamina and the sheath-like base. It is also characterized by its glabrous achenes, although we studied a single specimen from El Cajas (Azuay, Ecuador) displaying achenes with indumentum (*Ulloa & Minga 1393*, HA).

The species shows high variability in leaf size and density, which led some authors to describe infraspecific taxa or even to propose distinct species. *Werneria fontii* Cuatrec. was described from Tolima (Cordillera Central, Colombia) mainly on the basis of the compact habit of the plants thriving in this area. These plants are certainly smaller than typical forms and display short leaves with the leaf lamina barely spreading from the sheath-like base. Specimens also collected in the paramos of Tolima were previously used by Weddell (1856) to describe a variety that he characterized as having flesher leaves than typical variety, which is in line with the remarks stated by Cuatrecasas (1935) under *W. fontii*. However, these small and compact plants are not exclusive from this region. We studied similar forms from Farallones de Cali (Cordillera Occidental, Colombia), Nariño (southern Colombia), Tungurahua (Ecuador), and from Ancash (central Peru). On the other hand, some specimens from the paramos of Chingaza and Sumapaz (Cordillera Oriental, Colombia) display unusual narrow leaves that Cuatrecasas named *in sched.* as W. humilis var. angustifolia (*Cuatrecasas 25628*, US-01269081; *Cleef 1316*, US-01324876). From label information, it can be understood that these plants grow almost submerged in the water, which would explain the singular morphology of these forms. The Bolivian populations, which represent the southern limit of distribution of *X. humile*, rather exhibit a morphology similar to the typical forms. The puzzling pattern of the different morphologies along the whole distribution area makes unadvisable the recognition of more than a single species.

*Xenophyllum humile* shows morphological affinities with *X. funkianum*, *X. rigidum*, *X. roseum*, and *X. sotarense* (see comments under these species). Some specimens from Cundinamarca (Colombia) have the rhizome-like stems covered with a dense lanate indumentum and with scarce leaves along them (e.g., *Pedraza & Franco 452*, COL; *Cleef & Florschütz 5469*, COL, US). They might be confused with X. crassum subsp. orientale (Cuatrec.) J.Calvo, although their distributions areas do not overlap and *X. humile* has shorter and narrower leaves, shorter involucral bracts, and smaller capitula. We also studied a few specimens containing material mixed with *Werneria pygmaea* Gillies ex Hook. & Arn., a strictly rosettiform species that can be readily differentiated because it grows in lax clumps or solitary plants and its leaves are acute to obtuse at the apex and have the leaf lamina barely spreading from the sheath-like base (vs. leaves obtuse to truncate at the apex or callous-like tipped, usually with the leaf lamina spreading at nearly 90° from the sheath-like base in *X. humile*); see *Cuatrecasas & Idrobo 27005* (COL) and *Soderstrom 1293* (US).

An isolectotype of the name *Werneria humilis* Kunth is kept at the Willdenow Herbarium. This specimen was sent by Humboldt to Willdenow as stated at the bottom right of the sheet. It is not numbered but contains individuals identical to those from the lectotype at P.

Regarding the name *Werneria fontii*, it should be noted that the three specimens at MA are numbered with the same herbarium number, i.e., MA-246359. The isolectotypes, therefore, are indicated as MA-246359b and MA-246359c in order to differentiate them from the lectotype.

#### Additional specimens examined.

**Bolivia. La Paz**: Murillo, bajando de la cumbre 16.7 km hacia Unduavi, pasando Pongo, 16°19'S, 67°57'W, 28 Apr 1991, *S.G. Beck 18750* (LPB, USM); Murillo, entre Pongo y Unduavi, mina 50, subiendo hasta la mina San Luis, 16°17'S, 67°55'W, 28 Oct 1994, *S.G. Beck et al. 21519* (LPB); Nor Yungas, San Luis, al NE en línea recta a 3.5 km del poblado de Pongo, 16°18'S, 67°55'W, 13 Nov 2016, *F. Zenteno et al. 19280* (LPB). **Colombia. Antioquia**: páramo Frontino, cerro de Campanas, 6°27'N, 76°7'W, 29 Oct 1976, *J.D. Boeke & J.B. McElroy 294* (US); **Caldas**: páramo del Ruiz, higher slopes, 29 Aug 1957, *H.G. Barclay 5238* (COL); Villamaría, Nevado del Ruiz, a orillas de la carretera, 23 Sep 1980, *L.A. Camargo 7406* (COL); cordillera Central, vertiente occidental, nevado de Santa Isabel, cabeceras del río Otún, 4°46'N, 75°25'W, 25 Nov 1946, *J. Cuatrecasas 23219* (CONC); cordillera Central, vertiente occidental, páramos del nevado del Ruiz, 5 May 1940, *J. Cuatrecasas 9283* (US); S side of Nevado el Cisne near laguna Verde, 4°50'N, 75°21'W, 28 Jan 1986, *V.A. Funk 8091* (COL, US); S side of Nevado el Cisne near laguna Verde, 4°50'N, 75°21'W, 28 Jan 1986, *V.A. Funk 8093* (COL, US); Cordillera Central alrededores del refugio del Ruiz, carretera El Silencio, 4°50'N, 75°22'W, 7 Oct 1978, *O. Rangel, H. Sturm & S. Zuluaga 1734* (COL); Manizales, sector del Cisne, 4°51'N, 75°21'W, Oct 2010, *W.G. Vargas 22353* (ICESI); **Cauca**: Puracé, P.N.N. Puracé, entre Pilimbalá y el volcán Puracé, 2°22'N, 76°24'W, 14 Nov 2004, *J. Betancur & M. Gutiérrez 11215* (COL); Popayán, volcán Sotará, antecima del volcán, 2°6'N, 76°35'W, 30 Nov 2017, *J. Calvo, T. Buira & C. Velasco 7622* (CAUP); cordillera Central, vertiente occidental, cabeceras del río Páez, páramos entre Perro Muerto y la laguna del Páez, 2°59'N, 76°2'W, 4/5 Dec 1944, *J. Cuatrecasas 19095* (F); S del P.N. Puracé, inmediaciones de la laguna La Magdalena, sector Valencia, 1°55'N, 76°36'W, 15 Sep 1987, *A. Duque 559* (COL); páramo de Las Papas, El Boquerón y alrededores de la laguna La Magdalena, 1°55'N, 76°36'W, 16 Oct 1958, *J.M. Idrobo & H.G. Barclay 4039* (COL); Totoró, vereda Santa Teresa, páramo Carga Chiquillo, 2°26'N, 76°23'W, 9 Jul 2002, *E.L. Muñoz 915* (CAUP); páramo de Moras, between Mozoco and Pitayó, Tierra Adentro, 2°41'N, 76°14'W, Feb 1906, *H. Pittier 1418* (US); Totoró, corregimiento de Gabriel López, trayecto a la laguna la Herradura, 2°30'N, 76°13'W, 17 Apr 2009, *B.R. Ramírez & J.F. Álvarez 21060* (CAUP); Jambaló, Pitayó-Quichaya, área especial de manejo Cresta de Gallo, 2°42'N, 76°19'W, 3 Mar 2000, *G. Reina 472* (CAUP); **Cundinamarca**: P.N.N. Sumapaz, Nazaret, Santa Rosa, 4°11'N, 74°11'W, 2 Oct 1999, *M. Ángel et al. 9* (COL); Cordillera Oriental al sur de Usme, páramo de Chisacá, near edge of laguna Grande (Piñuelal), 4°18'N, 74°7'W, 16 Nov 1958, *H.G. Barclay & P. Juajibioy 6220* (COL); La Calera, P.N.N. Chingaza, lagunas de Buitrago, 4°45'N, 73°49'W, 24 Jun 2017, *L. Camelo, J. Castillo & J. Bernal 205* (COL); Bogotá, localidad de Usme, El Carrizal, alrededores de la laguna El Pato, 4°18'N, 74°9'W, 17 Jan 2013, *N. Castaño & A. Orejuela 1429* (COL); páramo de Palacio, lagunas de Buitrago y alrededores, 4°45'N, 73°49'W, 16 Dec 1971, *A.M. Cleef 294* (COL, US); páramo de Sumapaz, Chisacá, cabeceras del río S. Rosa, aprox. 1 km al S de la laguna Larga, 4°17'N, 74°12'W, 25 Aug 1972, *A.M. Cleef 5277* (US); cabeceras de la quebrada Chuza entre la mina de cal del páramo de Palacio y el páramo de Chingaza, laguna Seca y alrededores, 1 km approx. al NW de la laguna, 4°40'N, 73°47'W, 19 Sep 1972, *A.M. Cleef & P.A. Florschütz 5469* (COL, US); macizo de Bogotá, eastern drainage, páramo de Palacio, quebrada de Casarreales, 14 Dec 1959, *J. Cuatrecasas, M.T. Murillo & R. Jaramillo 25628* (COL, MA, US); páramo de Chisacá, around the laguna de Chisacá, 4°17'N, 74°12'W, 29 Dec 1959, *J. Cuatrecasas & R. Jaramillo 25746* (COL); macizo de Bogotá-macizo de Sumapaz, vertiente oriental, Media Naranja, 4°11'N, 74°15'W, 4 Jan 1969, *J. Cuatrecasas & J.M. Idrobo 27005* (COL [mixed with *Werneria pygmaea*]); La Calera, páramo Chingaza, laguna Seca, 4°40'N, 73°46'W, 28 Dec 1999, *A. Díaz s.n.* (COL); páramo de Chisacá, entre las lagunas y Nazareth, 25 Aug 1977, *S. Díaz-Piedrahita 1131* (COL); Fómeque, P.N.N. Chingaza, inmediaciones del campamento permanente del área administrativa del parque, sendero Suasié, 12 Nov 2015, *D. Giraldo-Cañas et al. 6026* (COL); páramo de Chisacá, laguna Verde, 16 Sep 1952, *T. Hammen 506* (COL); Fómeque, páramo de Chingaza, laguna del Medio, 4°30'N, 73°44'W, 15 Dec 1963, *G. Huertas & L.A. Camargo 5831* (COL); páramo de Chisacá, 5 Apr 1960, *L.E. Mora 1101* (COL); P.N.N. Sumapaz, vereda Santa Rosa, alrededores de la laguna de los Tunjos, 4°16'N, 74°12'W, 7 Aug 1998, *P. Pedraza et al. 274* (COL); laguna de Chisacá, 4°17'N, 74°12'W, 13 Dec 1998, *P. Pedraza & P. Franco 452* (COL); pico el Zapato, 4°14'N, 74°11'W, 14 Jan 1999, *P. Pedraza, C. Pedraza & A. Varón 614* (COL); páramo de Chisacá, alrededores de las lagunas de Chisacá (hoya del río Magdalena), 4°17'N, 74°12'W, 10 Feb 1961, *P. Pinto & J. Hernández 485* (COL); Sumapaz, a 2 km de la división de la carretera a San Juan, 3 Oct 1978, *O. Rangel 1655* (COL); Pasca, páramo de Chisacá al SE de la laguna de los Tunjos, 4°16'N, 74°12'W, 7 Nov 1987, *R. Sánchez 727* (COL); páramo de Siberia, 24 Feb 1952, *M. Schneider 1182* (COL); páramo de Chisacá, 4°17'N, 74°12'W, 5 Oct 1966, *T.R. Soderstrom 1293* (US [mixed with *W. pygmaea*]); macizo de Sumapaz, delante de las lagunas de Chisacá, 4°17'N, 74°11'W, 30 Sep 1963, *L. Uribe Uribe 4492* (COL); páramo de Sumapaz, lagunas de Chisacá, 4°17'N, 74°11'W, 2 Dec 1968, *L. Uribe Uribe 6206* (COL); Fómeque, P.N.N. Chingaza, lagunas de Buitrago, 4°45'N, 73°49'W, 12 Aug 2000, *F. Zapata 66* (COL); **Huila**: vía al Nevado del Huila, 2°47'N, 76°1'W, 8 Dec 1993, *C. Barbosa 9586* (COL); páramo de Las Papas, colinas al SE de la laguna La Magdalena sobre el cerro La Carona y El Boquerón, vertiente del Magdalena, 1°55'N, 76°35'W, 8 Sep 1958, *J.M. Idrobo, P. Pinto & H. Bischler 3109* (COL); **Meta**: páramo de Sumapaz, Cerro Nevado del Sumapaz, pico del Nevado, exposición SE, 3°56'N, 74°6'W, 30 Jan 1972, *A.M. Cleef 1316* (US); páramo de Sumapaz, Cerro Nevado del Sumapaz, pico del Nevado, lado NE, 3°56'N, 74°6'W, 11 Jan 1973, *A.M. Cleef 7628* (COL, US); macizo de Sumapaz, entre el plano del Nevado y la laguna del Sorbedero, 3°56'N, 74°8'W, 13 Jul 1981, *S. Díaz-Piedrahita et al. 2792* (COL); **Nariño**: Pasto, volcán Galeras, 1°13'N, 77°21'W, 1 Oct 1965, *C.E. Acosta 12* (COL); Pasto, volcán Galeras, 1°13'N, 77°21'W, 1 Oct 1965, *C.E. Acosta 13* (COL); in monte ignivomo Azufral, 18 May 1876, *E. André 3281* (US); Cumbal, volcán de Chiles, en la antena vía El Laurel, Maldonado-Carchi, 0°49'N, 77°55'W, Jul 2012, *F. Ávila 2247* (UDBC); Cumbal, volcán de Chiles, en la antena vía El Laurel, Maldonado-Carchi, 0°49'N, 77°55'W, Jul 2012, *F. Ávila 2252* (UDBC); Cumbal, volcán de Chiles, en la antena vía El Laurel, Maldonado-Carchi, 0°49'N, 77°55'W, Jul 2012, *F. Ávila 2259* (UDBC); Cumbal, volcán de Chiles, en la antena vía El Laurel, Maldonado-Carchi, 0°49'N, 77°55'W, Jul 2012, *F. Ávila 2272* (UDBC); Mallama-Piedrahancha, páramo del Infiernillo, km 97 vía Pasto-Tumaco, vereda Pueblo Viejo, entre la cabaña principal de la reserva y el sitio “Las Lagunetas”, 1°3'N, 77°46'W, 8 Jan 2009, *L.M. Caballero et al. 31* (COL); macizo del volcán Galeras, E slope, around La Torre, 1°12'N, 77°21'W, 7 Feb 1965, *J. Cuatrecasas & L.E. Mora 26899* (COL, US); La Cruz, corregimiento de La Estancia, volcán Doña Juana, 1°28'N, 76°55'W, 16 Nov 2001, *S.L. Díaz Ibarra 2087* (CAUP); volcán de El Galeras (N slope) above Pasto, 1°12'N, 77°21'W, 18 Oct 1944, *J.A. Ewan 16323* (US); camino de herradura entre Túquerres y el volcán Azufral, 8 Jan 1952, *A. Fernández & L.E. Mora 1136* (COL); Cumbal, en la base del cerro nevado o volcán, 28 Oct 1955, *A. Fernández 2897* (COL); al N de Yacuanquer, 1°9'N, 77°23'W, 4 Jan 1943, *M. Garganta 488* (COL); volcán Galeras near Pasto, 1°12'N, 77°21'W, 11 Jan 1981, *A. Gentry et al. 30523* (COL); Túquerres, volcán Azufral, E slopes, ca. 9–13 km W of Túquerres, 1°5'N, 77°40'W, 12 May 1989, *J.L. Luteyn, J. Fuertes & O. Rangel 12826* (COL, US); Túquerres, volcán Azufral, 1°5'N, 77°40'W, 10 Feb 1962, *L.E. Mora 1881a* (US); Túquerres, volcán Azufral, 1°5'N, 77°40'W, 10 Feb 1962, *L.E. Mora 1883* (US); Túquerres, volcán Azufral, 1°5'N, 77°43'W, 30 Sep 1979, *L.E. Mora 7536* (COL); Pasto, P.N.N. Galeras, alrededores del volcán Galeras, 1°15'N, 77°26'W, 2 Sep 1999, *P. Pedraza & M. Alvear 667* (COL); Pasto, volcán Galeras, 1°13'N, 77°21'W, 6 Aug 1977, *P. Pinto et al. 1829* (COL); volcán Galeras, páramo, 1°13'N, 77°20'W, 22 Oct 1968, *T. Plowman 1954* (US); alrededores de Cumbal, 20 Jul 1952, *R. Romero-Castañeda 3279* (COL); Pasto, volcán El Galeras, 4 Jun 1946, *R.E. Schultes & M. Villarreal 7955* (COL, CONC, F); Cumbal, volcán Chiles, 0°49'N, 77°56'W, 23 Oct 1978, *H. Sturm 115* (COL); Túquerres, laguna Verde, 1°5'N, 77°43'W, May 1853, *J.J. Triana 2803.1* (COL); **Risaralda**: Cordillera Central, páramo del Quindío, 4°44'N, 75°23'W, 15 Aug 1922, *F.W. Pennell & T.E. Hazen 10020* (US); **Tolima**: at top of Cordillera Central, Florida-Herrera camino, 3°19'N, 76°2'W, 16 Nov 1944, *E.L. Core 1587* (US); Santa Isabel, paso de la Cordillera Central por la quebrada de África, 4°45'N, 75°22'W, 5 Feb 1980, *S. Díaz-Piedrahita & R. Jaramillo 1798* (COL); páramo de Ruiz, 16/17 Dec 1917, *F.W. Pennell 3039* (NY, US); **Valle del Cauca**: Los Farallones, ca. filo de la cordillera, al N del cerro Pance, vert. del Pacífico, 3°21'N, 76°42'W, 26 Jul 1991, *E. Calderón 43A* (COL); alto de Los Farallones, 3°20'N, 76°41'W, 27 Feb 1979, *C. Downey 131* (COL). **Ecuador. Azuay**: P.N. Cajas, cerca de la laguna Toreadora, 2°46'S, 79°13'W, 15 Nov 2000, *L. Endara & M. Nonhebel 496* (QCA); P.N. Cajas, ca. 30 km W of Sayausí, at pass NE toward highest peak, 2°46'S, 79°14'W, 23 Oct 1995, *V.A. Funk & X. Montezuma 11431* (QCA); P.N. Cajas, ca. 30 km W of Sayausí, at pass NE toward highest peak, 2°46'S, 79°14'W, 23 Oct 1995, *V.A. Funk & X. Montezuma 11432* (HA, QCA); P.N. Cajas, ca. 30 km W of Sayausí, at pass NE toward highest peak, 2°46'S, 79°14'W, 23 Oct 1995, *V.A. Funk & X. Montezuma 11433* (QCA); 5 km W of Soldados on Cuenca-San Joaquín-Angas rd., ca. 100 m up slopes N of rd. toward large laguna, near laguna Estrellas Cocha, 2°54'S, 79°15'W, 24 Oct 1995, *V.A. Funk & X. Montezuma 11442* (QCA); 5 km W of Soldados on Cuenca-San Joaquín-Angas rd., ca. 100 m up slopes N of rd. toward large laguna, near laguna Estrellas Cocha, 2°54'S, 79°15'W, 24 Oct 1995, *V.A. Funk & X. Montezuma 11443* (US); 5 km W of Soldados on Cuenca-San Joaquín-Angas rd., ca. 100 m up slopes N of rd. toward large laguna, near laguna Estrellas Cocha, 2°54'S, 79°15'W, 24 Oct 1995, *V.A. Funk & X. Montezuma 11444* (US); W of Cuenca on gravel road between Soldados and Balao (hwy 25), laguna Estrellas Cocha, 6 km W of Arch at Soldados Park entrance, 2°54'S, 79°15'W, 18 Apr 2018, *V.A. Funk & J.M. Bonifacino 14028* (US); W of Cuenca on gravel road between Soldados and Balao (hwy 25), laguna Estrellas Cocha, 6 km W of Arch at Soldados Park entrance, 2°54'S, 79°15'W, 18 Apr 2018, *V.A. Funk & J.M. Bonifacino 14031* (US); carretera Cuenca-Angas, entre Soldados y Angas, 3 Aug 1983, *J. Jaramillo & V. Winnerskjold 5497* (QCA); parque de recreación Cajas, 2 Sep 1984, *J. Jaramillo 7202* (QCA); P.N. Cajas, road Cuenca-Sayausí-Molleturo, km 38.4, from the pass to the top of ridge north of pass, 2°46'S, 79°14'W, 4 Jan 2000, *P.M. Jørgensen, C. Ulloa & E. Narváez 2098* (HA, QCNE); P.N. Cajas, road Cuenca-Sayausí-Molleturo, km 38.4, from the pass to the top of ridge north of pass, 2°46'S, 79°14'W, 4 Jan 2000, *P.M. Jørgensen, C. Ulloa & E. Narváez 2117* (HA, QCNE); arriba laguna Dos Chorreras, llamado “upper meadow”, 2°46'S, 79°9'W, 2 Jul 1995, *B. León & K. Young 3481* (QCA); Cuenca, Sayausí, Tres Cruces, 2°46'S, 79°14'W, 12 Apr 2013, *D. Minga & A. Verdugo 2567* (HA); eastern cordillera, between Oña and the río Yacuambi, 3°34'S, 79°5'W, 10/19 Sep 1945, *F. Prieto 285* (COL, RB); Cajas N.P., E side of cerro Amarillo, 2°45'S, 79°15'W, 14 Jul 1997, *P. Sklenář & V. Sklenářová 2556* (QCA); vicinity of Toreador [laguna Toreadora], between Molleturo and Quinoas, 2°46'S, 79°13'W, 15 Jun 1943, *J.A. Steyermark 53216* (RB); P.N. Cajas, Cuenca-Molleturo km 28, sendero Patoquinoas-Totoras, 2°47'S, 79°12'W, 29 Aug 2003, *C. Ulloa & D. Minga 1393* (HA); carretera Cuenca-San Joaquín-Angas, entre Soldados y Angas, 2°55'S, 79°15'W, 8 Oct 1990, *R. Valencia & J.C. Matheus 395* (QCA); **Bolívar**: Guaranda, subcuenca del río Chimbo, cerro Tililag, 1°36'S, 78°52'W, 23 Jan 1982, *V. Pasaca 1397* (LOJA); **Carchi**: páramo del Ángel, 21 Aug 1957, *H.G. Barclay 5017* (COL); páramo del Ángel, high ridge, 22 Aug 1957, *H.G. Barclay 5104* (COL); páramo del Ángel, on high ridge, 23 Aug 1957, *H.G. Barclay 5137* (COL); along road from El Chical to Tulcán, at summit of road midway through paramo del Ángel, 0°48'N, 77°56'W, 15 Oct 2012, *T.B. Croat et al. 104336* (QCNE); loma Paneíllo, 7.7 km W of Tufiño on road to Maldonado, 0°45'N, 77°50'W, 11 Nov 1988, *L.J. Dorr & L.C. Barnett 6035* (QCA, QCNE); roas between Tulcán and Maldonado, S of volcán Chiles, 0°47'N, 77°58'W, 12 Mar 1985, *B. Eriksen 59003* (QCA, QCNE); paramo El Ángel, between towns of El Ángel and Tulcán near the pass, 4 Mar 1992, *V.A. Funk & M. Gavilanes 11071* (QCA, QCNE); paramo El Ángel, between towns of El Ángel and Tulcán near the pass, 4 Mar 1992, *V.A. Funk & M. Gavilanes 11072* (QCA, QCNE); volcán Chiles, rd. from Tulcán to Maldonado, 32 km W of the bridge at the W edge of Tulcán, 5 Mar 1992, *V.A. Funk & M. Gavilanes 11075* (QCA, QCNE); volcán Chiles, rd. from Tulcán to Maldonado, 38 km W of the bridge at the W edge of Tulcán, 5 Mar 1992, *V.A. Funk & M. Gavilanes 11078* (QCA, QCNE); El Ángel-Tulcán, around laguna El Voladero, 0°42'N, 77°53'W, 9 Aug 1990, *P.M. Jørgensen et al. 92321* (COL, QCA, QCNE); páramo El Ángel, laguna S de El Voladero, 0°37'N, 78°26'W, 18 Dec 1990, *S. León 1116* (QCA); páramo El Ángel, laguna S de El Voladero, 0°37'N, 78°26'W, 1 Sep 1983, *P. Mena 192* (QCA); páramo El Ángel, laguna S de El Voladero, 0°37'N, 78°26'W, [without date], *P. Mena 43* (QCA); Espejo, reserva ecológica El Ángel, sitio de lagunas El Voladero, 0°42'N, 77°53'W, 31 Oct 1993, *W. Palacios 11636* (QCNE); S slopes of volcán Chiles, 0°49'N, 77°57'W, 21 Oct 1987, *P.M. Ramsay & P.J. Merrow-Smith 866* (QCA, QCNE); El Ángel, 19 Aug 1999, *M. Smeets & M. Lind van Wijngaarden 736* (QCA, QCNE); Espejo, asociación 23 de Julio, 0°43'N, 77°55'W, 3 Aug 2003, *D. Suárez, M. Chinchero & M. Cabascango 1325* (QCNE); Espejo, parroquia La Libertad, humedal Potrerillos, 0°42'N, 77°52'W, 3 Aug 2011, *H. Valles & S. Chimbolema 373* (QCA); **Chimborazo**: eastern slope of Mount Chimborazo, 1°27'S, 78°46'W, 24 Jul 1939, *E. Asplund 7819* (LIL); Cordillera Oriental, páramo de la laguna Negra, al N y al E de Alao, 1°46'S, 78°26'W, 11 Aug 1959, *H.G. Barclay & P. Juajibioy 8782* (COL); Colta, vía Ambrosio Lazo, El Puyal, 1°41'S, 78°48'W, 2 May 2013, *J. Caranqui, W. Haro & F. Salas 2182* (QCA); Alausí, laguna de Ozogoche, 2°13'S, 78°37'W, 10 Jul 2013, *J. Caranqui 2439* (QCA); comunidad de Ambrosio Lazo, sector Patococha-Loma Caparina, 1°44'S, 78°53'W, 5 Jun 2009, *D. Cárate et al. 609* (QCA); comunidad de Ambrosio Lazo, quebrada Cóndor, sector Yurak Rume, 1°43'S, 78°54'W, 6 Jun 2009, *D. Cárate et al. 646* (QCA); comunidad de Ambrosio Lazo, Cóndor Alto, 1°43'S, 78°52'W, 7 Jun 2009, *D. Cárate et al. 675* (QCA); nev. Chimborazo, cerca al refugio, 1°29'S, 78°48'W, 13 Jul 2009, *D. Cárate et al. 950A* (QCA); SW slope of volcano Chimborazo, 1°1'S, 78°47'W, 27 Jun 2012, *N. Morueta-Holme, K. Engemann & P. Sandoval 84* (QCA); El Altar, N side of the volcano, on the ridge below the Canoningo peak, 1°41'S, 78°24'W, 19 Aug 1995, *P. Sklenář & V. Kostečková 1039* (QCA); cerro Yanaurcu, on the W side of the N ridge of the mountain, 2°14'S, 78°30'W, 29 Oct 1995, *P. Sklenář & V. Kostečková 148-17* (QCA); W side of the Chimborazo volcano, 1°28'S, 78°52'W, 5 Jul 1999, *P. Sklenář 7508* (QCA, QCNE); **Cotopaxi**: P.N. Cotopaxi, faldas N de Rumiñahui, 0°40'S, 78°30'W, 28 Oct 1982, *H. Balslev, J. Brandbyge & L. Coloma 3347* (QCA); around the Illiniza peaks, 4–10 mi. W from town of Magdalena, 0°39'S, 78°40'W, 2 Apr 1991, *R. Bensman 370* (QCNE); entre Aucacocha y Tambo, 31 Dec 1983, *J. Jaramillo 6297* (QCA); paramo de Quispicacha, E slope of loma Pucyucuchu, 1°5'S, 78°50'W, 24 Oct 2006, *P. Sklenář 9086* (QCA, QCNE); paramo de Quispicacha, E slope of loma Pucyucuchu, 1°5'S, 78°50'W, 23 Oct 2006, *P. Sklenář 9125* (QCA); paramo de Quispicacha, summit plateau of loma Pucyucuchu, 1°5'S, 78°50'W, 25 Oct 2006, *P. Sklenář 9268* (QCA); **Imbabura**: Otavalo, reserva ecológica Cotacachi-Cayapas, SW base of Cotacachi mountain, 0°25'N, 78°20'W, 28 Apr 1996, *J.L. Clark 2534* (QCNE); laguna de Mojanda, 0°8'N, 78°16'W, 13 Aug 1976, *B. Øllgaard & H. Balslev 8749* (US); cúspide cerro Fuya-Fuya, al S de Otavalo, 0°8'N, 78°17'W, 27 Jan 1980, *J. Jaramillo & F. Coello 2078* (QCA); Cotacachi, slopes of volcán Cotacachi, 0°35'N, 78°20'W, 11 Oct 1987, *P.M. Ramsay & P.J. Merrow-Smith 759* (QCA, QCNE); nevado Cotacachi, on the SW side of the SE ridge of the volcano, 0°21'N, 78°21'W, 9 Sep 1995, *P. Sklenář & V. Kostečková 120-1* (QCA); cerro Imbabura, on the E side of the volcano, 0°15'N, 78°10'W, 5 Jun 1995, *P. Sklenář & V. Kostečková 31-20* (QCA); **Loja**: Loma del Oro cerca de Yacuambi, cerca de las lagunas de Condorcillo, 3°34'S, 79°4'W, 25 Oct 1997, *R. Bussmann & S. Lange s.n.* (HUTPL); Cajanuma, Podocarpus, camino a las lagunas del Compadre, 4°7'S, 79°9'W, 13 Dec 2017, *J. Calvo 7681* (HUTPL); Jimbura-Zumba road, km 17, at small lake, 4°44'S, 79°25'W, 27 Jul 1990, *P.M. Jørgensen, C. Ulloa & M. Gavilanes 92210* (QCA, QCNE); lagunas N of km 17 on road Jimbura-Zumba, 4°43'S, 79°26'W, 13 Aug 2001, *S. Laegaard et al. 21588* (QCNE); Podocarpus N.P., along the trail from Nudo de Cajanuma towards lagunas Compadre, ca. 3–4 km from the refuge, 4°7'S, 79°9'W, 19 Aug 2004, *P. Sklenář 8369* (QCA); **Morona-Santiago**: 24 km E of Gualaceo on rd. to Limón, at pass up rd. to tower, 3°0'S, 78°39'W, 25 Oct 1995, *V.A. Funk & R.X. Zamora 11450* (QCA); cerros Yuibug-Pailacajas, E side of the mountain ridge, 1°45'S, 78°27'W, 30 Jul 1997, *P. Sklenář & V. Sklenářová 2903* (QCA); W side of the rocky mountain ridge to the E of cerro Yuibug, 1°44'S, 78°27'W, 1 Aug 1997, *P. Sklenář & V. Sklenářová 3099* (QCA); **Napo**: Archidona, Sumaco Napo-Galeras N.P., Sumaco, crater, 0°34'S, 77°38'W, 16 Mar 1996, *J.L. Clark 2219* (QCNE); rd. from Quito to Baeza, just at the pass, 0°19'S, 78°12'W, 22 Feb 1992, *V.A. Funk et al. 11024* (QCA, QCNE); SW-slope 1.5 km from cerro Quilindaña, 0°47'S, 78°21'W, 1 Apr 1979, *L. Holm-Nielsen 16403* (QCA, US); NW slope of Antisana, N of lago Mauca-Machay, 0°26'S, 78°9'W, 2 Nov 1979, *L. Holm-Nielsen 20787* (QCA); cordillera de los Llanganates, loma between río Muyu, río Toro and río Verde Grande, 5 km WNW of cerro Hermoso, 1°12'S, 78°19'W, 9 Nov 1980, *L. Holm-Nielsen & J. Jaramillo 28223* (QCA); cordillera de los Llanganates, W shoulder of cerro Hermoso, 1.5 km W of the summit, 1°13'S, 78°17'W, 11 Nov 1980, *L. Holm-Nielsen & J. Jaramillo 28475* (QCA); cordillera de los Llanganates, shoulder of cerro Hermoso, 1.5 km W of the summit, 1°13'S, 78°18'W, 12 Nov 1980, *L. Holm-Nielsen & J. Jaramillo 28722* (QCA); Antisana, Jan 1865, *J. Isern 43* (MA); carretera Pifo-Papallacta, páramo de Guamaní, 0°19'S, 78°12'W, 15 Jan 1981, *J. Jaramillo 4129* (QCA); páramo de Guamaní, N of paso de La Virgen, 0°17'S, 78°10'W, 10 Jun 1984, *S. Laegaard 52249* (QCA, QCNE); Archidona, volcán Sumaco, segunda cumbre cerca al cráter, 0°32'S, 77°37'W, 19 Sep 2012, *P. Lozano et al. 79* (QCA); 13.5 km W of Papallacta, 26 Mar 1972, *B. MacBryde & J.D. Dwyer 1207* (QCA); Pisayambo, laguna Cochas Negras, 1°6'S, 78°19'W, 14 Jan 1999, *B. Merino & Á. Sánchez s.n.* (LOJA [mixed with X. crassum subsp. crassum]); Llanganati, below summit of Pan de Azúcar, 1°9'S, 78°18'W, 15 May 1982, *B. Øllgaard et al. 38552* (QCA); Llanganati, ridge between Pan de Azúcar and Las Torres de Llanganati, 1°9'S, 78°17'W, 15 May 1982, *B. Øllgaard et al. 38573* (QCA); páramo de Soguillas, near Las Torres de Llanganati, old crater ridge, 1°8'S, 78°15'W, 16 May 1982, *B. Øllgaard & L. Holm-Nielsen 38755* (QCA); reserva ecológica Oyacachi, 0°13'S, 78°8'W, 16 Dec 2008, *K. Romoleroux, D. Cárate & L.E. López 5355* (QCA); NE side of Antisana, 0°27'S, 78°8'W, 1 Nov 2007, *P. Sklenář & E. Rejzková 10707* (QCA); volcán Antisana, W side of the mountain, 0°30'S, 78°10'W, 21 Jul 1997, *P. Sklenář & V. Sklenářová 2810* (QCA); NE side of volcán Antisana, 0°27'S, 78°8'W, 17 Aug 1997, *P. Sklenář & V. Sklenářová 3403* (QCA); NE side of volcán Antisana, 0°27'S, 78°8'W, 17 Aug 1997, *P. Sklenář & V. Sklenářová 3422* (QCA); NE side of volcán Antisana, 0°27'S, 78°8'W, 18 Aug 1997, *P. Sklenář & V. Sklenářová 3435* (QCA); NE side of volcán Antisana, 0°27'S, 78°8'W, 18 Aug 1997, *P. Sklenář & V. Sklenářová 3538* (QCA); Quijos, reserva ecológica Antisana, páramo de Guamaní, carretera Pifo-Papallacta, La Virgen, 0°20'S, 78°12'W, 24 Jul 1998, *H. Vargas, E. Narváez & W. Quizhpe 1936* (QCNE); Tena, P.N. Llanganates, vía Salcedo-Tena, de laguna Chaloa Cocha desvío a Rayo Filo, 0°57'S, 78°23'W, 20 Sep 1998, *H. Vargas, E. Narváez & S. Orellana 2679* (QCNE); **Orellana**: N side of cerro Sumaco, 100 m NW of campsite, 0°32'S, 77°37'W, 24 Apr 1979, *L. Holm-Nielsen, J. Jaramillo & T. Vries 17109* (QCA); N side of cerro Sumaco, 100 m NW of campsite, 0°32'S, 77°37'W, 24 Apr 1979, *L. Holm-Nielsen, J. Jaramillo & T. Vries 17126* (QCA); N side of cerro Sumaco, upper part of the loma NW of campsite, 0°32'S, 77°37'W, 25 Apr 1979, *L. Holm-Nielsen, J. Jaramillo & T. Vries 17204* (QCA); S side of crater of cerro Sumaco, 0°33'S, 77°37'W, 26 Apr 1979, *L. Holm-Nielsen, J. Jaramillo & T. Vries 17312* (QCA); Sumaco, 0°32'S, 77°37'W, Jun 1865, *J. Isern 313* (MA); S side of cerro Sumaco, 100–200 m S of the main crater, 0°33'S, 77°37'W, 29 Apr 1979, *B. Løjtnant & U. Molau 12942* (QCA); **Pichincha**: vicinity of Quito, Rucu Pichincha, 0°9'S, 78°33'W, 31 Aug 1939, *E. Asplund 8612* (LIL); Cordillera Oriental, entre Pifo y el boquerón de cerro Corrales, páramo de Guamaní, 0°19'S, 78°12'W, 15 Aug 1959, *H.G. Barclay & P. Juajibioy 8900* (COL); flanco SW del volcán Sincholagua, 0°35'S, 78°21'W, 1 Jun 1985, *J. Bosco 145* (QCA); faldas SE volcán Guagua Pichincha, proximidades del refugio, 0°10'S, 78°35'W, 25 May 1985, *J. Bosco & M. Marcillo 72B* (QCA); entre Pifo y Papallacta, 0°19'S, 78°12'W, 26 Nov 1977, *S. Castroviejo, M. Costa & E. Valdés-Bermejo 1053* (MA); Rumiñahui, bosque protector Pasochoa, línea de cumbre del Pasochoa, 0°22'S, 78°27'W, 17 Nov 1990, *C.E. Cerón & R. Alarcón 12307* (QCNE); Cayambe, laguna San Marcos, 0°7'N, 77°57'W, 27 Dec 1999, *B. Cuamacás & E. Gudiño 473* (QCNE); Quito, parroquia Lloa, sector NE del volcán Guagua Pichincha, 0°10'S, 78°35'W, 12 Aug 2014, *D. Fernández et al. 1739* (QCNE); Quito, parroquia Lloa, sector NE del volcán Guagua Pichincha, 0°10'S, 78°35'W, 12 Aug 2014, *D. Fernández et al. 1743* (QCNE); Quito, volcán Atacazo, 0°22'S, 78°35'W, 28 Jun 2000, *D. Fernández, M. Cerna & P. Villacrés 325* (QCNE); Atacazo, 0°21'S, 78°37'W, Sep 1928, *F.G. Firmín 531* (US); road from Cayambe city to Mt. Cayambe, dirt/rock road high on Mt. Cayambe, W slope, 0°0’, 78°1'W, 24 Apr 2018, *V.A. Funk & J.M. Bonifacino 14094* (US); páramo de Guamaní, carretera Quito-Pifo-Papallacta, 0°23'S, 78°9'W, 20 Oct 1990, *E. Guerrón 13* (QCA, QCNE); volcán Cayambe, S slope of the vulcanic cone, valley near road to refugio, 0°3'S, 78°0'W, 18 Jun 1980, *L. Holm-Nielsen 24224* (QCA); volcán Cayambe, S slope of the vulcanic cone, near the refugio, 0°2'S, 77°59'W, 18 Jun 1980, *L. Holm-Nielsen 24245* (QCA); volcán Iliniza, NE slope below the refugio, 0°39'S, 78°42'W, 13 Aug 1980, *L. Holm-Nielsen, B. Øllgaard & C. Sperling 24899* (QCA); volcán Iliniza, NE slope below the refugio, 0°38'S, 78°42'W, 13 Aug 1980, *L. Holm-Nielsen, B. Øllgaard & C. Sperling 24959* (QCA); volcán Iliniza, NE slope below the refugio, 0°38'S, 78°42'W, 13 Aug 1980, *L. Holm-Nielsen, B. Øllgaard & C. Sperling 24982* (QCA); route de Tufiño à Maldonado, 10 km après Tufiño, 6 Jul 1988, *C. Huttel 1385* (QCA); Andium quitensium, crescit in jugis Andium, Dec 1860, *W. Jameson s.n.* (QPLS); vía Chillogallo-San Juan, partidero desde la población de San Juan hacia faldas del Atacazo “antenas militares”, 0°20'S, 78°36'W, 13 Jul 1980, *J. Jaramillo & M. Lascano 3142* (QCA); NE Pasochoa, 0°27'S, 78°28'W, 30 Jul 1980, *J. Jaramillo, R. Narváez & F. Coello 3168* (QCA); 1 km SE of Cayambe on road to hacienda Piemonte, 10 May 1990, *R.M. King, P.M. Peterson & E.J. Judziewicz 10052* (QCA, QCNE); páramo de Guamaní, paso de la carretera Quito-Baeza, 0°19'S, 78°12'W, 25 Aug 1985, *B.B. Larsen & B. Dall 190* (QCA); páramo de Guamaní, carretera Pifo-Papallacta, km 27, 0°19'S, 78°12'W, 13 Jan 1991, *S. León 1135* (QCA); páramo de Guamaní, carretera Pifo-Papallacta, km 27, 0°19'S, 78°12'W, 13 Jan 1991, *S. León 1136* (QCA); in m. Antisana, Sep 1897, *L. Mille 469* (QPLS); upper SE slopes of Guagua Pichincha, between the refuge and the crater rim, 0°11'S, 78°36'W, 9 Jan 1988, *U. Molau, B. Eriksen & B.B. Klitgaard 2403* (QCA, QCNE); faldas occidentales del volcán Antisana, 0°28'S, 78°12'W, 4 Mar 1984, *L. Muñoz 339* (QCA); volcán Cayambe, N slopes, along road to the antenna, 0°5'N, 77°59'W, 9 Jul 1980, *B. Øllgaard et al. 34196* (QCA); road Olmedo-laguna San Marcos, E of the pass, 0°7'N, 77°59'W, 10 Jul 1980, *B. Øllgaard et al. 34456* (QCA); páramo de Guamaní, laguna de Hoyas, 0°15'S, 78°12'W, 8 Aug 1987, *P.M. Ramsay & P.J. Merrow-Smith 186* (QCA, QCNE); páramo de Guamaní, 0°15'S, 78°12'W, 7 Oct 1987, *P.M. Ramsay & P.J. Merrow-Smith 726* (QCA, QCNE); W side of a mountain ridge ca. 2 km to the W from cerro SaraUrcu, 0°6'S, 77°57'W, 29 Aug 1995, *P. Sklenář & V. Kostečková 100-11* (QCA); W side of a mountain ridge ca. 2 km to the W from cerro SaraUrcu, 0°6'S, 77°57'W, 30 Aug 1995, *P. Sklenář & V. Kostečková 111-7* (QCNE); nevado Cayambe, SW slopes left from the road to the refuge, 0°1'N, 78°1'W, 1 Jul 1995, *P. Sklenář & V. Kostečková 49-16* (QCA); nevado Cayambe, SW slopes left from the road to the refuge, 0°1'N, 78°1'W, 1 Jul 1995, *P. Sklenář & V. Kostečková 51-9* (QCNE); Cotopaxi volcano, on the N side of the mountain, 0°39'S, 78°25'W, 28 Jun 1999, *P. Sklenář 7328* (QCA, QCNE); NE slopes of Rucu Pichincha, 0°10'S, 78°34'W, 18 May 1995, *P. Sklenář & V. Kostečková 8-1* (QCNE); in pasc. andin. m. Rucu Pichincha, Jul 1871, *L. Sodiro 61/3* (QPLS); cerro Atacazo, pendiente N cumbre Atacazo, 0°20'S, 78°36'W, 18 Jun 1983, *B. Treiber de Espinosa 101* (QCA); volcán Cayambe, cerca del refugio, 0°0’, 78°1'W, 6 Oct 2010, *C. Ulloa et al. 2416* (QCA); **Sucumbíos**: parroquia El Playón de San Francisco, 0°36'N, 77°40'W, 16 Oct 2008, *D. Reyes et al. 3688* (QCNE); **Tungurahua**: páramo del Caryhuayrazo, lado SW, entrando por el arenal del Chimborazo, 1°24'S, 78°48'W, 1 Aug 1988, *C.E. Cerón, M. Cerón & G. Viteri 4397* (QCNE); páramo del Caryhuayrazo, lado SW, entrando por el arenal del Chimborazo, 1°24'S, 78°48'W, 1 Aug 1988, *C.E. Cerón, M. Cerón & G. Viteri 4408* (QCNE); páramo del Caryhuayrazo, lado SW, entrando por el arenal del Chimborazo, 1°24'S, 78°48'W, 1 Aug 1988, *C.E. Cerón, M. Cerón & G. Viteri 4423* (QCNE); Santiago de Píllaro, páramos de Pisayambo, alrededor de la laguna de Pisayambo, 1°5'S, 78°23'W, 9 Oct 1998, *E. Cueva 207* (QCNE); cordillera de los Llanganates, Achiriqui, páramo de Jaramillo, 12.7 km NW of cerro Hermoso, 1°9'S, 78°21'W, 7 Nov 1980, *L. Holm-Nielsen & J. Jaramillo 28012* (QCA); cordillera de los Llanganates, N end of the loma between río Muyu and río Verde Grande, 5.5 km WNW of cerro Hermoso, 1°12'S, 78°19'W, 9 Nov 1980, *L. Holm-Nielsen & J. Jaramillo 28183* (QCA); cordillera de los Llanganates, N end of the loma between río Muyu and río Verde Grande, 5.5 km WNW of cerro Hermoso, 1°12'S, 78°19'W, 9 Nov 1980, *L. Holm-Nielsen & J. Jaramillo 28186* (QCA); cordillera de los Llanganates, S side of laguna Verde at cerro Hermoso, 1.8 km from the summit, 1°14'S, 78°18'W, 11 Nov 1980, *L. Holm-Nielsen & J. Jaramillo 28423* (QCA); cordillera de los Llanganates, páramo de Jaramillo, 12 km NW of cerro Hermoso, 1°9'S, 78°21'W, 14 Nov 1980, *L. Holm-Nielsen & J. Jaramillo 28781* (QCA); páramo de Jaramillo, 2 Nov 1984, *J. Jaramillo 7359* (QCA); Santiago de Píllaro, P.N. Llanganates, desde el río Millín hasta la colina Ashpachaca, 1°8'S, 78°22'W, 12 Oct 1998, *E. Narváez & W. Quizhpe 302* (QCNE); Santiago de Píllaro, P.N. Llanganates, W of cerro Hermoso, near saddle between headwaters of río Verde and río Topo, 1°11'S, 78°19'W, 12 Nov 1999, *D. Neill et al. 11959* (QCNE); Santiago de Píllaro, P.N. Llanganates, SW ridge of cerro Hermoso, 1°13'S, 78°17'W, 14 Nov 1999, *D. Neill et al. 12077* (QCNE); Santiago de Píllaro, P.N. Llanganates, SW ridge of cerro Hermoso, 1°13'S, 78°17'W, 14 Nov 1999, *D. Neill et al. 12095* (COL, QCNE); Santiago de Píllaro, P.N. Llanganates, SW ridge of cerro Hermoso, 1°13'S, 78°17'W, 14 Nov 1999, *D. Neill et al. 12112* (COL, QCA, QCNE); Ambato, ladera NW del cerro Carihuairhazo, 1°23'S, 78°45'W, 2 Feb 2012, *K. Romoleroux & G. Peyre 5756* (QCA); P.N. Llanganates, 1°7'S, 78°21'W, 15 Feb 2009, *S. Salgado 686* (QCA); volcán Tungurahua, N side of the mountain, trail to the summit, 1°27'S, 78°27'W, 8 Aug 1997, *P. Sklenář & V. Sklenářová 3246* (QCA); cerro Hermoso, SW ridge of the mountain, 1°14'S, 78°18'W, 6 Sep 1997, *P. Sklenář & V. Sklenářová 3604* (QCA); cerro Hermoso, SW ridge of the mountain, 1°14'S, 78°18'W, 6 Sep 1997, *P. Sklenář & V. Sklenářová 3632* (QCA); Patate, P.N. Llanganates, faldas del cerro Pan de Azúcar, en el trayecto páramo de Soguillas-cerro Pan de Azúcar, 1°9'S, 78°17'W, 13 Oct 1998, *H. Vargas, J.C. Ronquillo & N. Granda 2837* (QCNE); Patate, P.N. Llanganates, faldas del cerro Pan de Azúcar, en el trayecto páramo de Soguillas-cerro Pan de Azúcar, 1°9'S, 78°17'W, 13 Oct 1998, *H. Vargas, J.C. Ronquillo & N. Granda 2838* (QCNE); Patate, P.N. Llanganates, alrededores de la laguna Pan de Azúcar, 1°9'S, 78°17'W, 14 Oct 1998, *H. Vargas, J.C. Ronquillo & N. Granda 2869* (QCNE); **Zamora-Chinchipe**: cordillera de Sabanilla, cerca de la carretera Jimbura-Zumba, alrededor de la laguna Negra, 4°42'S, 79°25'W, 22 Oct 1996, *R. Bussmann & S. Lange s.n.* (HUTPL, QCNE). **Peru. Ancash**: cordillera Blanca, quebrada Illanro, nevado Portachuelo, north side to west of glacier, 9°2'S, 77°35'W, 13 Jul 1979, *M. Gibby & J.A. Barrett 185* (BM); Carhuaz, Huascarán N.P., quebrada Ulta, below Ulta pass, 9°7'S, 77°31'W, 28 Jul 1985, *D.N. Smith 11323* (F, MO, USM); **Cajamarca**: surroundings of Cajamarca, Jan 1986, *B. Becker & F.M. Terrones 303* (LPB); surroundings of Cajamarca, 29 Jan 1986, *B. Becker & F.M. Terrones 328* (LPB); Tingo, minas Conga, 6°55'S, 78°21'W, 3 Oct 2005, *A. Granda 2491* (MOL); **Junín**: Jauja, encima de la hacienda Runatullu, 11°39'S, 74°58'W, 23 Apr 1913, *A. Weberbauer 6628* (MOL, USM); **San Martín**: Mariscal Cáceres, valle al oeste del campamento Chochos en el P.N. del río Abiseo, 7°37'S, 77°28'W, 28 Jun 1996, *A. Cano et al. 7388* (USM).

### 3. Xenophyllum sotarense

Taxon classificationPlantaeAsteralesAsteraceae

(Hieron.) V.A.Funk, Novon 7(3): 240. 1997.

B64286E6-2DCD-54EC-9D78-138060E47550

Werneria sotarensis Hieron., Bot. Jahrb. Syst. 21(3): 363. 1895 [“soratensis”]. Type. Colombia. Cauca: Popayán al Sotará, cúspide del Sotará, 4400 m, Feb 1869, *A. Stübel 339b* (lectotype: MA-246367 (fragment!), designated by Funk (1997a: 240)). Epitype, designated here: Colombia. Cauca: Popayán, volcán Sotará, cima del volcán, 2°06’31’’N, 76°35’31’’W, 4440 m, 30 Nov 2017, *J. Calvo, T. Buira & C. Velasco 7640* (MA-01-00928840!; isoepitype: CAUP s.n.!).
Werneria leucobryoides S.F.Blake, J. Washington Acad. Sci. 18: 494. 1928. Type. Ecuador. Napo: in m. Quilindaña ad nives perpetuas, Dec 1897, *E. Festa s.n.* (holotype: NY s.n.!; isotypes: G-00305674 (digital image!), P-02088568 (digital image!), Q-001222!, QPLS s.n.!, US-00037321 (fragment!)).

#### Description.

Suffruticose plant, forming dense mats or hummocks, with rhizome-like stems up to 5 cm long covered with sparse pilose indumentum and old leaves, rather erect, simple or branched from the base. ***Stems*** 1–1.5 cm tall (aerial part), sparsely pilose. ***Leaves*** densely stellate-imbricate, extending into a sheath-like base that bears scattered multicellular trichomes; leaf laminas 4–5 × 1–1.2 mm, linear, aristate at the apex (arista up to 0.5 mm long that falls as the leaves age), entire, thinly hyaline (in dried specimens), elliptical in cross section, glabrous, unconspicuously nerved above, 1-nerved beneath (barely visible), fleshy, drying coriaceous, matte, finely papillose. ***Capitula*** radiate, erect, sessile to subsessile. ***Involucres*** 4.9–5 × 4.8–5 mm, cupuliform; involucral bracts 12 to 13, 3.2–4 × 1.2–1.3 mm, obtuse at the apex, greenish. ***Ray florets*** ca. 13; corollas 6.3–6.5 × 0.8–1.2 mm, 2 to 4-veined, subentire to 2-toothed at the apex, conspicuously surpassing the involucre, white. ***Disc florets*** 14 to 15; corollas 3.3–4 mm long, whitish, usually purple-tipped; style branches truncate with a crown of sweeping trichomes, purplish. ***Achenes*** ca. 2.2 × 0.5 mm, cylindrical, 7 to 8-ribbed, glabrous; pappus 4.8–6.2 mm long, barbellate, whitish. Chromosome number unknown. Fig. 2B.

#### Additional iconography.

Blake (1928: 496, fig. 1Q–S sub *Werneria leucobryoides*).

#### Distribution and habitat.

Southern Colombia to central Ecuador. Colombia (border Cauca/Huila), Ecuador (Chimborazo, Morona-Santiago, Napo, Tungurahua). It grows in rocky outcrops and scree slopes around the upper limit of vegetation of the superparamo ecoregion, between elevations of 4125–4800 m (Fig. 9).

#### Phenology.

Flowering from July to December.

#### Etymology.

The epithet *sotarense* refers to the Sotará Volcano, the *locus classicus* of this species. It is located in southern Colombia on the border between the departments of Cauca and Huila.

#### Notes.

*Xenophyllum sotarense* is characterized by its linear, 4–5 mm long, densely stellate-imbricate leaves, which are typically aristate at the apex. The arista measures up to 0.5 mm long and falls as the leaf ages. The sheath-like base bears scattered multicellular trichomes.

It is morphologically close to *X. humile*, especially to those small and compact plants with leaf laminas barely spreading from the sheath-like bases (see comments under it). To avoid misidentifications the following characters should be carefully studied: leaf apex (aristate or clearly acute when the arista falls in *X. sotarense* vs. obtuse to truncate in *X. humile*) and stem indumentum (sparsely pilose in *X. sotarense* vs. lanate in *X. humile*). *Xenophyllum sotarense* also shows morphological affinities with *X. roseum*. The involucre length (4.9–5 mm in *X. sotarense* vs. 10–12 mm in *X. roseum*), the ray corolla length and color (6.3–6.5 mm, white in *X. sotarense* vs. 11.8–19 mm, pink in *X. roseum*), and the achene indumentum (glabrous in *X. sotarense* vs. white-villous in *X. roseum*) are useful characters for differentiating them.

The lectotype of *Werneria sotarensis* Hieron. at MA consists in a single sterile fragment barely informative and no isolectotypes appear to be extant; the duplicate at B was destroyed in 1943 (F0BN015821). For that reason, an epitype collected in the *locus classicus* has been designated. It is noteworthy that the plants from this locality are restricted on the volcano summit and co-occur with *X. humile*, a species much more abundant in the area that was also found at lower elevations.

The isotypes of *Werneria leucobryoides* S.F.Blake located at the Ecuadorian herbaria Q and QPLS show that the collector of this material was the Italian naturalist Enrico Festa instead of Luis Sodiro as the protologue indicates. This correction has been adopted since these specimens are the only ones that bear a label handwritten by Sodiro. It is feasible to think that the collector information was corrected on the specimens belonging to Sodiro’s personal herbarium but omitted on the duplicates to be distributed.

#### Additional specimens examined.

**Ecuador. Chimborazo**: El Altar, N side of the volcano, on the ridge below the Canoningo peak, 1°41'S, 78°24'W, 19 Aug 1995, *P. Sklenář & V. Kostečková 90-5* (QCNE); **Morona-Santiago**: cerros Yuibug-Pailacajas, E side of the mountain ridge, 1°45'S, 78°27'W, 31 Jul 1997, *P. Sklenář & V. Sklenářová 2941* (QCA, US); cerros Yuibug-Pailacajas, E side of the mountain ridge, 1°45'S, 78°27'W, 31 Jul 1997, *P. Sklenář & V. Sklenářová 3022* (QCA); **Napo**: cerro Antisana, NW of north peak of Antisana, 0°27'S, 78°9'W, 28 Jul 1960, *P.J. Grubb et al. 630* (K, NY); NE side of volcán Antisana, 0°27'S, 78°8'W, 17 Aug 1997, *P. Sklenář & V. Sklenářová 3414* (QCA, US); NE side of volcán Antisana, 0°27'S, 78°8'W, 17 Aug 1997, *P. Sklenář & V. Sklenářová 3418* (QCA); NE side of volcán Antisana, 0°27'S, 78°8'W, 17 Aug 1997, *P. Sklenář & V. Sklenářová 3424* (QCA); volcán Antisana, NE side of the volcano, 0°27'S, 78°8'W, 21 Sep 2017, *P. Sklenář 14116* (QCA); in pascuis summis m. Quilindaña, 0°47'S, 78°19'W, [without date], *L. Sodiro s.n.* (QPLS); **Tungurahua**: Santiago de Píllaro, P.N. Llanganates, SW ridge of cerro Hermoso, 1°13'S, 78°17'W, 14 Nov 1999, *D. Neill et al. 12104* (QCNE); Santiago de Píllaro, P.N. Llanganates, SW ridge of cerro Hermoso, 1°13'S, 78°17'W, 14 Nov 1999, *D. Neill et al. 12167* (QCNE); P.N. Llanganatis, slope on the W side of cerro Hermoso, 1°13'S, 78°17'W, 3 Dec 2010, *P. Sklenář 13132* (QCA); cerro Hermoso, SW ridge of the mountain, 1°14'S, 78°18'W, 6 Sep 1997, *P. Sklenář & V. Sklenářová 3741* (US).

### 4. Xenophyllum roseum

Taxon classificationPlantaeAsteralesAsteraceae

(Hieron.) V.A.Funk, Novon 7(3): 240. 1997.

332245BD-861F-575A-B2E0-391CC4102A94

Werneria rosea Hieron., Bot. Jahrb. Syst. 28(5): 648. 1901. Werneria humilis var. rosea (Hieron.) Rockh., Bot. Jahrb. Syst. 70: 295. 1939. Type. Ecuador. Azuay: páramo del Cajas, W Andes of Cuenca, 3800−4300 m, [without date], *F.C. Lehmann 5687* (lectotype: K-000527743 (digital image!), designated by Funk (1997a: 240); isolectotype: US-00037306 (fragment!)).
Werneria purpurea Spruce ex Rockh., Bot. Jahrb. Syst. 70: 295. 1939, *nom. inval. pro syn.* (Turland et al. 2018, ICN Art. 36.1).

#### Description.

**Figure 8.**
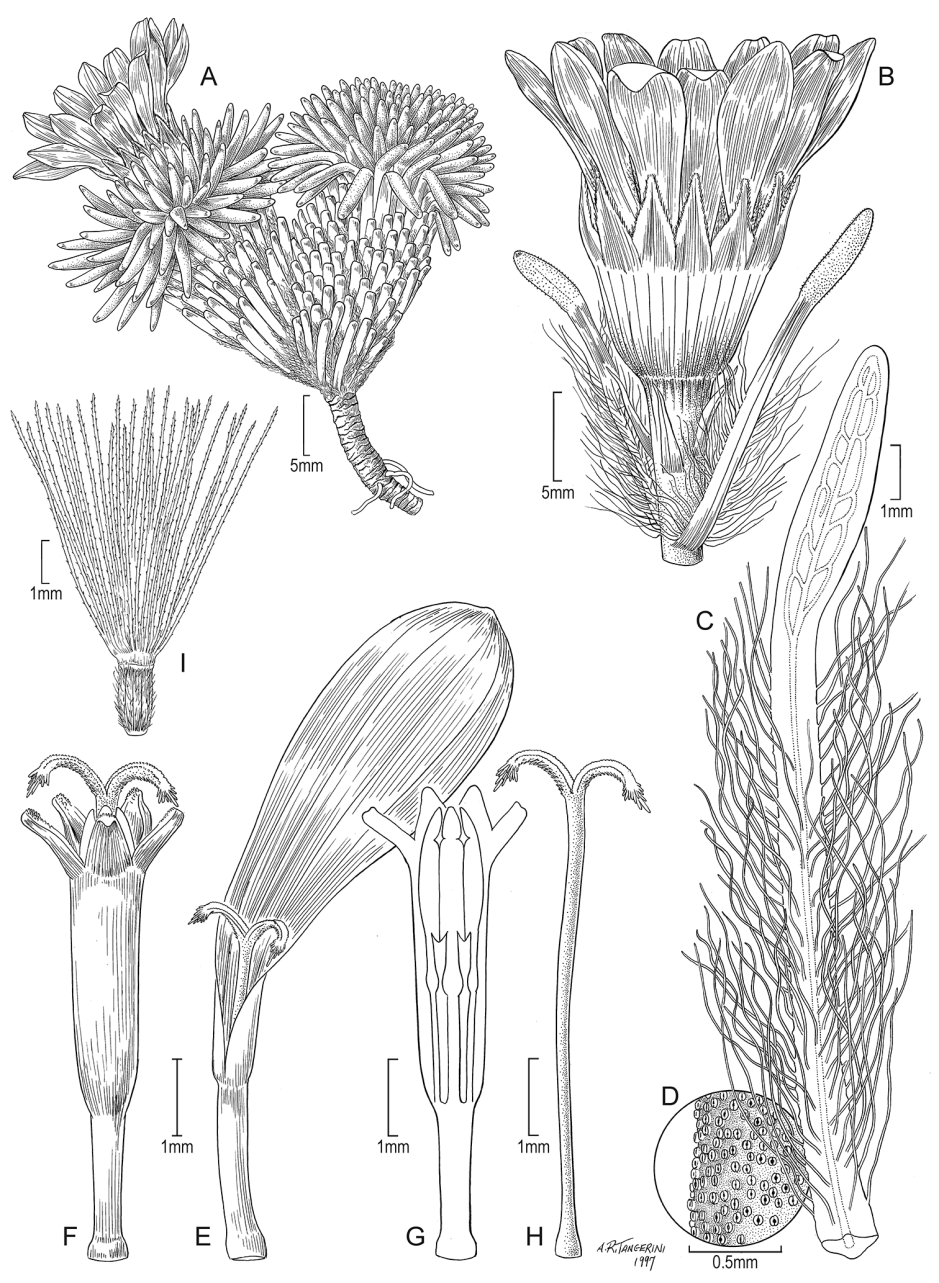
*Xenophyllum roseum***A** habit **B** capitulum **C** adaxial leaf surface **D** detail of leaf surface **E** ray corolla and style **F** disc corolla and style branches **G** disc corolla and stamens (vertically sectioned, style removed) **H** style **I** immature achene with pappus. All details drawn from *Jameson s.n.* (US) except for **A** (drawn from *Funk & Montezuma 11441*, US). Illustration by Alice Tangerini.

Suffruticose plant, forming dense mats or hummocks, with rhizome-like stems up to 6.5 cm long covered with matted lanate indumentum and leaf bases, rather erect, simple or branched from the base. ***Stems*** 1.5–2 cm tall (aerial part), lanate. ***Leaves*** stellate-imbricate, extending into a sheath-like base that bears long silky trichomes, usually with the leaf lamina spreading at nearly 90° from the sheath-like base; leaf laminas 4.6–10.6 × 1.3–1.5 mm, linear, obtuse, callous-like tipped at the apex (the young ones usually bearing a quickly deciduous arista up to 0.5 mm long), entire, elliptical to almost terete in cross section (rather flat when dried), glabrous, unconspicuously nerved above, 1-nerved beneath (barely visible; in dried specimens the nerves are noticeable on both faces), fleshy, matte, papillose. ***Capitula*** radiate, erect, sessile. ***Involucres*** 10–12 × 7.9–8 mm, cupuliform; involucral bracts ca. 13, 5.5–7.6 × 1.6–2 mm, acute at the apex, greenish, purple-edged. ***Ray florets*** 11 to 13; corollas 11.8–19 × 2.5–3 mm, 4-veined, subentire to 3-toothed at the apex, conspicuously surpassing the involucre, pink. ***Disc florets*** 28 to 34; corollas 5.7–8 mm long, yellowish; style branches penicillate, yellowish. ***Achenes*** cylindrical, white-villous with trichomes ca. 0.5 mm long (immature); pappus ca. 7 mm long, barbellate, whitish. Chromosome number unknown. Fig. 8.

#### Distribution and habitat.

**Figure 9.**
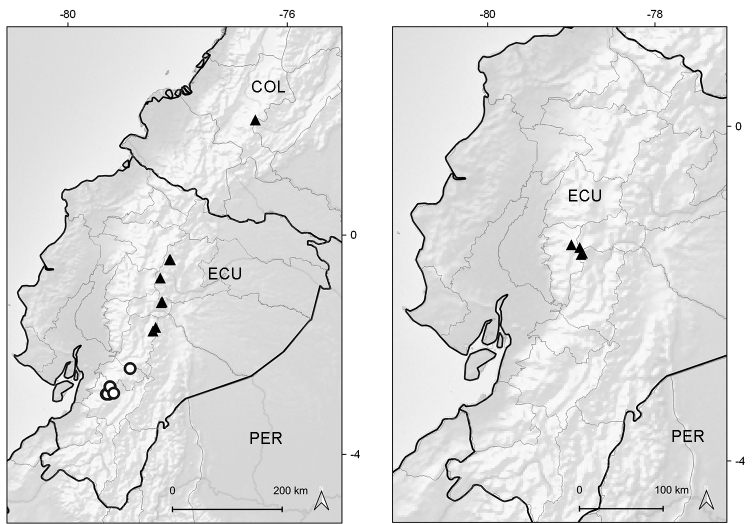
Distribution map of *Xenophyllum sotarense* (left hand, closed triangle), *X. roseum* (left hand, open circle), and *X. funkianum* (right hand).

Endemic to Ecuador (Azuay, Cañar, Loja). It grows in moist grasslands and exposed places of the paramo ecoregion, between elevations of 3650–4275 m (Fig. 9).

*Xenophyllum roseum* is a narrow endemic species only known from El Cajas National Park (Azuay) and the Culebrillas Lagoon (Cañar). A single collection from Loja (*Jameson s.n.*, US) would also support its presence in this province. However, the label information does not provide a precise locality (“province of Loxa”), and therefore, it has not been represented on the map. New collections are required to confirm that *X. roseum* thrives in Loja.

#### Phenology.

Flowering from August to March.

#### Etymology.

The adjective *roseus -a -um* means rose-like, rose-colored, pink-colored. It refers to the characteristic pink ray corollas of this species.

#### Notes.

*Xenophyllum roseum* is well defined by its linear, stellate-imbricate leaves that usually have the leaf lamina spreading at nearly 90° from the sheath-like base, and by the obtuse leaf apex that usually bears a quickly deciduous arista when young. The achenes are covered with a white-villous indumentum and the ray corollas are pink. This latter character makes this species unique within the genus. The color is very noticeable on living plants but in old dried specimens it might be less obvious, and therefore, somewhat equivocal for identification purposes.

It is morphologically similar to *X. humile* and *X. sotarense*. From the former species, it differs in the ray corolla length and color (11.8–19 mm, pink in *X. roseum* vs. 4.2–12.2 mm, white in *X. humile*), disc corolla length and color (5.7–8 mm, yellowish in *X. roseum* vs. 3.1–5 mm, whitish in *X. humile*), and achene indumentum (white-villous in *X. roseum* vs. glabrous in *X. humile*). The deciduous arista that usually adorns the young leaves of *X. roseum* also helps to differentiate it from *X. humile*. With respect to *X. sotarense*, the differences are detailed under this latter species. Among the aforementioned species, *X. roseum* overlaps its distribution area with that of *X. humile*.

#### Additional specimens examined.

**Ecuador. Azuay**: road Cuenca-Angas, 28 Dec 1976, *J.D. Boeke 650* (QCA, US); P.N. Cajas, cerca de la laguna de Soldados, 2°54'S, 79°18'W, 17 Nov 2000, *L. Endara & M. Nonhebel 554* (QCA); Cajas, ca. 30 km W of Sayausí, at pass NE toward highest peak, 2°46'S, 79°14'W, 23 Oct 1995, *V.A. Funk & X. Montezuma 11426* (HA, QCA, US); 5 km W of Soldados on Cuenca-San Joaquín-Angus rd., ca. 100 m up slopes N of rd. toward large laguna, near laguna Estrellas Cocha, 2°54'S, 79°15'W, 24 Oct 1995, *V.A. Funk & X. Montezuma 11441* (US); W of Cuenca on gravel road between Soldados and Balao (hwy 25), laguna Estrellas Cocha, 6 km W of Arch at Soldados Park entrance, 2°54'S, 79°15'W, 18 Apr 2018, *V.A. Funk & J.M. Bonifacino 14033* (US); Assuay, Oct 1864, *W. Jameson s.n.* (E); P.N. Cajas, páramo de Soldados, road Cuenca-San Joaquín-Soldados, at the pass above Soldados, 2°54'S, 79°17'W, 8 Jan 2000, *P.M. Jørgensen et al. 1738* (HA, QCNE, US); P.N. Cajas, road Cuenca-Sayausí-Molleturo, at the pass, km 38.4, 2°46'S, 79°14'W, 3 Jan 2000, *P.M. Jørgensen, C. Ulloa & E. Narváez 2083* (HA, US); Cuenca, Sayausí, Tres Cruces, 2°46'S, 79°14'W, 13 Oct 2017, *D. Minga, M. Jiménez & N. Guzmán 3242* (HA); área nacional de recreación Cajas, Totorococha-Mazan valley, 2°53'S, 79°10'W, 12 Sep 1987, *P.M. Ramsay & P.J. Merrow-Smith 448* (QCNE); Andibus Ecuadorensibus, in repisis m. Azuay, Aug 1857/9, *R. Spruce 6013* (BM, C, E, F [fragment], GH, GOET, K, LE, NY, P, TCD, W); P.N. Cajas, km 35.7 redondel Cuenca-Molleturo, en el paso, sendero Paragüillas, 2°46'S, 79°14'W, 13 Jan 2003, *C. Ulloa, P.M. Jørgensen & X. Clavijo 1173* (HA); Cuenca, Sayausí, P.N. Cajas, Tres Cruces, sendero del estacionamiento hacia arriba, 2°46'S, 79°14'W, 28 Apr 2015, *C. Ulloa et al. 2534* (HA); **Cañar**: El Tambo, camino de la laguna Culebrillas hasta Ingapirca, cerca de la comunidad de Cajontambo, 2°26'S, 78°52'W, 11 Mar 2009, *D. Minga & F. Nugra 1594* (HA); **Loja**: province of Loxa, Sep 1864, *W. Jameson s.n.* (US).

### 5. Xenophyllum funkianum

Taxon classificationAnimaliaAsteralesAsteraceae

J.Calvo, PhytoKeys: 139: 30. 2020.

591709A5-586C-51A9-A8CA-395993C6FC2E

#### Type.

Ecuador. Chimborazo: Mt. Chimborazo area, at the end of *Polylepis* road and beginning of hike to *Polylepis* forest, 1°31’50’’S, 78°52’55’’W, 4233 m, 20 Apr 2018, *V.A. Funk & J.M. Bonifacino 14059* (holotype: US!; isotypes: MO!, QCA!).

#### Description.

**Figure 10.**
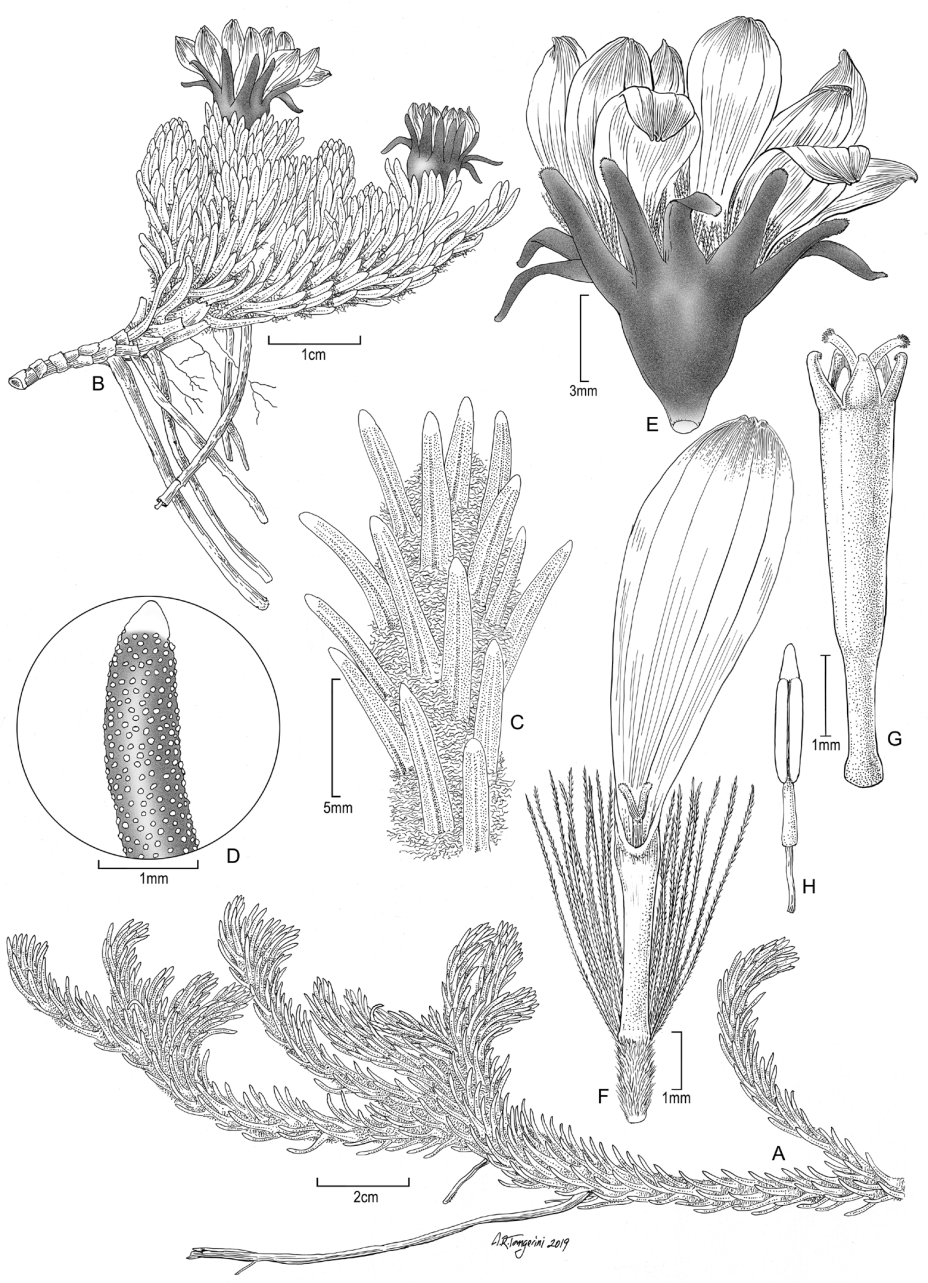
*Xenophyllum funkianum***A** habit **B** habit with capitula **C** stem apical part **D** detail of leaf apex **E** capitulum **F** ray floret (frontward bristles removed) **G** disc corolla and style branches **H** stamen. All details drawn from *Funk & Bonifacino 14059* (US). Illustration by Alice Tangerini.

Suffruticose plant, forming lax mats, with rhizome-like stems 20–35 cm long covered with arachnoid indumentum and leaf base remnants resembling paleae, horizontal, creeping, simple or branched from the base. ***Stems*** 2–3 cm tall (aerial part), arachnoid. ***Leaves*** imbricate, extending into a sheath-like base that bears arachnoid trichomes; leaf laminas 5.3–7.8 × 0.8–0.9 mm, linear, rather acute, callous-like tipped at the apex, entire, elliptical in cross section, glabrous, unconspicuously nerved above, 1-nerved beneath (only visible in the lower third), fleshy, shiny, papillose. ***Capitula*** radiate, erect, sessile. ***Involucres*** 9–11 × 5–7 mm, cupuliform; involucral bracts 13 to 14, 4.7–6.9 × 1–1.7 mm, acute at the apex, dark-burgundy. ***Ray florets*** 12 to 13; corollas 8.9–11.6 × 2.3–3 mm, 4-veined, subentire to 3-toothed at the apex, conspicuously surpassing the involucre, white, somewhat purplish beneath. ***Disc florets*** 20 to 23; corollas 5–5.6 mm long, yellowish; style branches truncate with a crown of sweeping trichomes, yellowish. ***Achenes*** cylindrical, white-villous (immature); pappus 3.9–6.2 mm long, barbellate, whitish. Chromosome number unknown. Fig. 10.

#### Additional iconography.

Calvo and Funk (2020: 32, fig. 2, as photo).

#### Distribution and habitat.

Endemic to Ecuador (Bolívar, Chimborazo). This species grows in exposed places and on sandy soils of the dry superparamo ecoregion, at elevations of 4100–4300 m (Fig. 9).

The species is known from a few collections near the Chimborazo Volcano. Its distribution area partially overlaps with those of *X. humile* and *X. rigidum*. See Calvo and Funk (2020) for further details.

#### Phenology.

Flowering from April to July.

#### Etymology.

The epithet honors the American botanist Vicki A. Funk (1947–2019), who described the genus *Xenophyllum* and greatly contributed to the understanding of the family Compositae.

#### Notes.

*Xenophyllum funkianum* is distinguished by its creeping rhizome-like stems 20–35 cm long, the straight linear leaves extending into a sheath-like base that bears arachnoid trichomes, the dark-burgundy sessile involucres with 13 to 14 involucral bracts, the 12 to 13 ray florets with white corollas somewhat purplish beneath, and by having white-villous achenes.

The morphologically closest species is *X. rigidum*. However, they can be differentiated by the leaf lamina size (5.3–7.8 × 0.8–0.9 mm in *X. funkianum* vs. 12.6–13.5 × 2.3–2.4 mm in *X. rigidum*), involucre size (9–11 × 5–7 mm in *X. funkianum* vs. 11.5–12.8 × 7.2–11.3 mm in *X. rigidum*), number of disc florets (20 to 23 in *X. funkianum* vs. 38 to 41 in *X. rigidum*), and by the way of growing of the rhizome-like stems (distinctly horizontal in *X. funkianum* vs. rather erect in *X. rigidum*). *Xenophyllum funkianum* is, furthermore, a smaller plant and the capitula are not enclosed among the leaves as in *X. rigidum*. Another similar species is *X. humile*; they differ in involucre length (9–11 mm in *X. funkianum* vs. 4.6–9.3 mm in *X. humile*), achene indumentum (white-villous in *X. funkianum* vs. glabrous in *X. humile*), and habit (rather creeping in *X. funkianum* vs. forming dense mats or hummocks in *X. humile*). Moreover, *X. funkianum* differs in having the leaf lamina barely spreading from the sheath-like base (vs. leaf lamina usually spreading at nearly 90° from the sheath-like base in *X. humile*).

#### Additional specimens examined.

**Ecuador. Bolívar**: road to Salinas, 1.8 km W of Guaranda-Ambato hwy., 1°25'S, 79°0'W, 25 Jun 1989, *L.J. Dorr & I. Valdespino 6474* (QCA, QCNE, US); **Chimborazo**: Mt. Chimborazo area, side road ends and connects to trail that leads to the *Polylepis* forest, 1°32'S, 78°53'W, 20 Apr 2018, *V.A. Funk & J.M. Bonifacino 14061* (US); W side of the Chimborazo volcano, arenal around loma Guagua Lozán, 1°27'S, 78°54'W, 3 Jul 1999, *P. Sklenář 7528* (QCA, QCNE).

### 6. Xenophyllum rigidum

Taxon classificationAnimaliaAsteralesAsteraceae

(Kunth) V.A.Funk, Novon 7(3): 240. 1997.

60E31C7D-A3B7-5FF5-AD8E-682FA5E32085

Werneria rigida Kunth, Nov. Gen. Sp. [ed. fol.] 4: 149. 1818. Type. Ecuador. [“in summis Andibus Quitensium” according to the *ind. loc.*], [without date], *F.W.H.A. Humboldt & A.J.A. Bonpland s.n.* (lectotype: Humboldt and Bonpland’s collection at P as the first-step lectotype, designated as “holotype” by Funk (1997a: 240); P-00320180 (digital image!) as the second-step lectotype, designated here; isolectotypes: B-W-16432-01-0 (digital image!), HAL-0113456 (digital image!), P-02088567 (digital image!), P-02088569 (digital image!), P-02088570 (digital image!)).
Oresigonia pycnophylla Willd. ex Rockh., Bot. Jahrb. Syst. 70: 292. 1939, *nom. inval. pro syn.* (Turland et al. 2018, ICN Art. 36.1).

#### Description.

**Figure 11.**
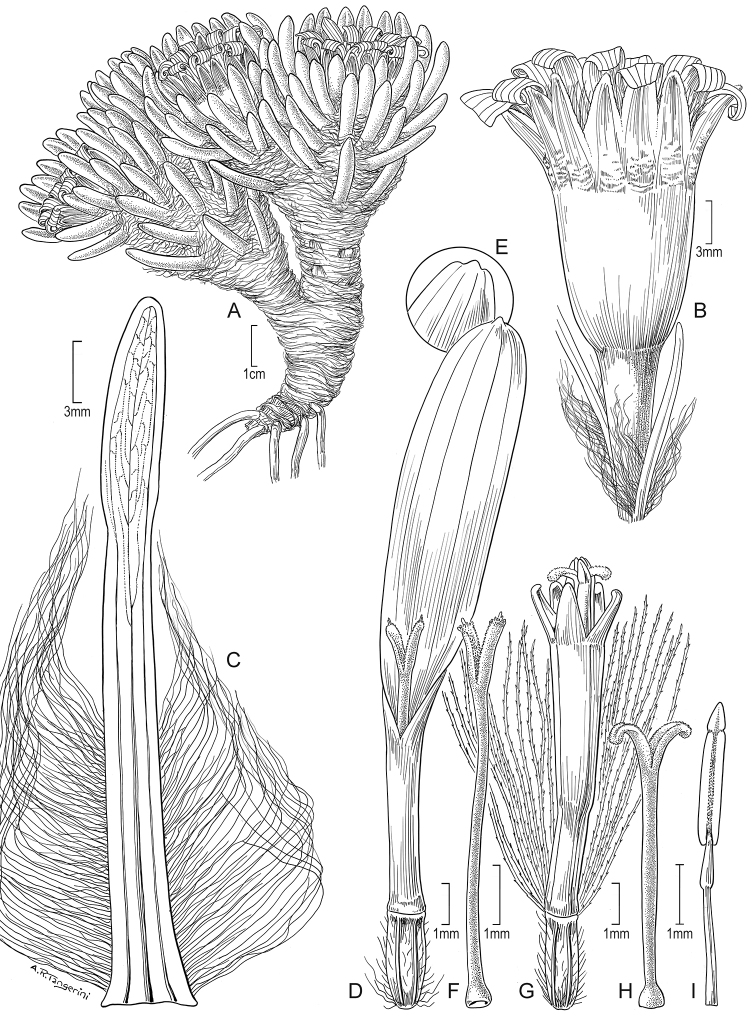
*Xenophyllum rigidum***A** habit **B** capitulum **C** adaxial leaf surface **D** ray floret (pappus removed) **E** detail of ray corolla apex **F** style of ray floret **G** disc floret (frontward bristles removed) **H** style of disc floret **I** stamen. All details drawn from *Asplund 8391* (US) except for **A** (drawn from *Grubb et al. 569*, NY), **C** (drawn from *Asplund 17334*, NY), and **G–I** (drawn from *Anthony & Tate 316*, US). Illustration by Alice Tangerini.

Suffruticose plant, forming mats or hummocks, with rhizome-like stems up to 16 cm long covered with matted lanate indumentum and leaf bases, rather erect, simple or branched from the base. ***Stems*** 2.5–4 cm tall (aerial part), lanate. ***Leaves*** stellate-imbricate, extending into a sheath-like base that bears long silky trichomes; leaf laminas 12.6–13.5 × 2.3–2.4 mm, linear, obtuse at the apex, entire, elliptical in cross section, glabrous, unconspicuously nerved above, 1-nerved beneath (only visible in the lower half), fleshy, drying coriaceous, rather matte, papillose. ***Capitula*** radiate, erect, sessile, completely enclosed among the leaves. ***Involucres*** 11.5–12.8 × 7.2–11.3 mm, cupuliform; involucral bracts ca. 13, 6.9–8.5 × 1.7–2 mm, rather acute at the apex, dark-purplish. ***Ray florets*** ca. 13; corollas 9.6–11 × 1.5–1.9 mm, 4 to 6-veined, subentire to 3-toothed at the apex, conspicuously surpassing the involucre, white. ***Disc florets*** 38 to 41; corollas 5.1–6.2 mm long, whitish, usually yellow-tipped; style branches truncate with a crown of sweeping trichomes or slightly penicillate, purplish. ***Achenes*** 2.9–3.8 × 1–1.1 mm, cylindrical, white-villous with trichomes ca. 1 mm long (ribs invisible); pappus 6.8–8.4 mm long, barbellate, whitish. Chromosome number unknown. Fig. 11.

#### Additional iconography.

Funk (1997b: 33, fig. 3D).

#### Distribution and habitat.

**Figure 12.**
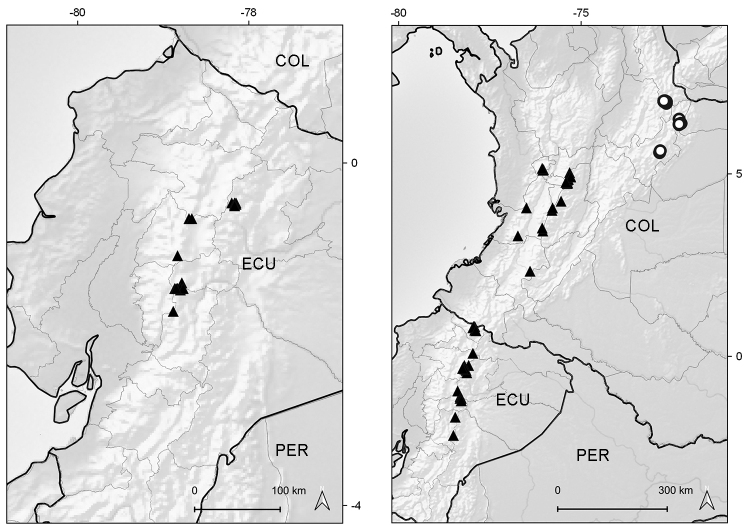
Distribution map of *Xenophyllum rigidum* (left hand), X. crassum subsp. crassum (right hand, closed triangle), and X. crassum subsp. orientale (right hand, open circle).

Endemic to Ecuador (Bolívar, Chimborazo, Cotopaxi, Napo, Pichincha, Tungurahua). It grows in rocky outcrops and on sandy soils, rather dry, around the upper limit of vegetation of the superparamo ecoregion, at elevations of (3500–)3900–5100 m (Fig. 12).

#### Phenology.

Flowering from April to November.

#### Etymology.

The epithet *rigidum* means stiff, inflexible and it describes the leaves of this species.

#### Notes.

*Xenophyllum rigidum* is readily distinguished by its tough, linear, and apically obtuse leaves, as well as by its white-villous achenes. The rhizome-like stems are very robust and covered with a matted lanate indumentum where the leaf bases are sunked in. Another characteristic feature of this species is that the capitula are completely enclosed among the leaves.

*Xenophyllum rigidum* is morphologically close to *X. funkianum*, X. crassum (S.F.Blake) V.A.Funk subsp. crassum (see comments under these taxa), and *X. humile*. From the latter species, it differs in having larger leaves (12.6–13.5 × 2.3–2.4 mm vs. 2.5–10.8 × 0.5–1.5 mm in *X. humile*), longer involucral bracts (6.9–8.5 mm vs 3.1–6.1 mm in *X. humile*), higher number of disc florets (38 to 41 vs. (8–)22 to 39 in *X. humile*), and white-villous achenes (vs. glabrous in *X. humile*).

The specimen B-W-16432-01-0 kept at the Willdenow Herbarium is considered part of the original material and designated as isolectotype. Willdenow received the specimen from Humboldt as stated by Schlechtendal on the blue label at the bottom of the sheet. Although unnumbered, it contains an individual identical to those from the lectotype. See Hind and Jeffrey (2001) for further details on Humboldt and Bonpland’s material in the Willdenow Herbarium.

#### Additional specimens examined.

**Ecuador. Bolívar**: volcán Chimborazo, W side of the mountain, ca. 4 km from the road Ambato-Guaranda, 1°28'S, 78°48'W, 14 Sep 1995, *P. Sklenář & V. Kostečková 1274* (QCA); **Chimborazo**: southern slope of Mount Chimborazo, 1°29'S, 78°48'W, 18 Aug 1939, *E. Asplund 8391* (CAS, US); comunidad de Ambrosio Lazo, sector Patococha-Loma Caparina, 1°44'S, 78°53'W, 5 Jun 2009, *D. Cárate et al. 593* (QCA); rd. to el refugio, ca. 17 km from Ambato-Guaranda rd., 0.5 km from refugio Whymper, 1°28'S, 78°50'W, 21 Oct 1995, *V.A. Funk 11422* (US); Mt. Chimborazo, along road toward los refugios del Chimborazo, near the end of the access road at the superparamo, near camping spaces, 1°28'S, 78°50'W, 20 Apr 2018, *V.A. Funk & J.M. Bonifacino 14053* (US); cerca del refugio E. Whymper, lado SW del Chimborazo, 1°28'S, 78°50'W, 14 Oct 1980, *S. Halloy B-182* (NY); Chimborazo, Nov 1864, *J. Isern 83* (C, MA); E side of the Chimborazo volcano, top of the terminal moraine around a small glacial lake, 1°28'S, 78°46'W, 4 Jul 1997, *P. Sklenář & V. Sklenářová 2228* (QCA, US); W side of the Chimborazo volcano, 1°28'S, 78°52'W, 5 Jul 1999, *P. Sklenář 7538* (QCA); S side of Chimborazo volcano, 1°28'S, 78°51'W, 1 Nov 2006, *P. Sklenář 9338* (QCA); **Cotopaxi**: around the Illiniza peaks, 4 mi. W of town of Magdalena, 0°39'S, 78°40'W, 2 Apr 1991, *R. Bensman 358* (QCNE); paramo de Quispicacha, summit plateau of loma Pucyucuchu, 1°5'S, 78°50'W, 25 Oct 2006, *P. Sklenář 9269* (QCA, QCNE); Iliniza, slope along the main pathway to the saddle, 0°39'S, 78°42'W, 5 Dec 2010, *V. Zeisek 13161* (QCA); **Napo**: Antisana, Oct 1923, *H.E. Anthony & G.H.H. Tate 316* (US); Mount Antisana, 17 Aug 1955, *E. Asplund 17334* (NY); Antisana, 13 Aug 1979, *J. Black 163* (AAU); Quijos, reserva ecológica Antisana, faldas SW del volcán Antisana, 0°29'S, 78°10'W, 28 Nov 1998, *A. Freire & L. Haro 2953* (QCNE, US); on south side of western glacier Antisana, 19 Jul 1960, *P.J. Grubb et al. 569* (NY); on boggy plain above hacienda Antisana, 8 Aug 1960, *P.J. Grubb et al. 704* (K); lado WNW del Antisana, 0°28'S, 78°9'W, 3 Feb 1980, *S. Halloy B-62* (LIL); paramo nordwestlich von Antisana, 27 Mar 1934, *E. Heinrichs 652* (MA); Quijos, reserva ecológica Antisana, SW slopes of volcán Antisana, just 100 m below glacier, 0°29'S, 78°9'W, 28 Nov 1998, *D. Neill et al. 11495* (QCNE); volcán Antisana, W side of the mountain, 0°30'S, 78°10'W, 21 Jul 1997, *P. Sklenář & V. Sklenářová 2793* (QCA); Quijos, reserva ecológica Antisana, faldas occidentales del volcán Antisana, NE de laguna Santa Lucía, 0°28'S, 78°10'W, 1 Aug 1998, *H. Vargas & E. Narváez 2113* (QCNE, US); Quijos, reserva ecológica Antisana, faldas SW del volcán Antisana, 0°30'S, 78°10'W, 28 Nov 1998, *H. Vargas & E. Narváez 3103* (QCNE, US); Quijos, reserva ecológica Antisana, faldas SW del volcán Antisana, 0°30'S, 78°10'W, 28 Nov 1998, *H. Vargas & E. Narváez 3117* (QCNE, US); Antisana, west side, Mar 1880, *E. Whymper s.n.* (BM); **Pichincha**: Mt. Antisana, 16 Jul 1939, *E.K. Balls 7308* (K); Antisana, falda WSW, 14 Sep 1986, *A. Ehrenburg 172* (QCA); faldas occidentales del volcán Antisana, 0°28'S, 78°12'W, 8 Mar 1984, *L. Muñoz 365* (LOJA, QCA); **Tungurahua**: Mocha, ca. 2 km NW of the mountain Carihuairazo, 1°24'S, 78°47'W, 23 Apr 1995, *J.L. Clark 725* (COL, QCNE).

### 7. Xenophyllum crassum

Taxon classificationAnimaliaAsteralesAsteraceae

(S.F.Blake) V.A.Funk, Novon 7(3): 239. 1997.

1B7C6428-255F-5D92-AB18-7F792C8C639E

Werneria crassa S.F.Blake, J. Washington Acad. Sci. 18: 495. 1928. Type. Colombia. Caldas: páramo del Quindío, 3700−4200 m, 15/20 Aug 1922, *F.W. Pennell & T.E. Hazen 10031* (lectotype: K-000527739 (digital image!), designated by Funk (1997a: 239); isolectotypes: GH s.n.!, NY s.n.!).

#### Description.

Suffruticose plant, forming dense mats or hummocks, with rhizome-like stems up to 17 cm long covered with matted lanate indumentum and leaf bases, rather erect, simple or branched from the base. ***Stems*** 1.5–4 cm tall (aerial part), lanate. ***Leaves*** stellate-imbricate, extending into a sheath-like base that bears long silky trichomes, usually with the leaf lamina spreading at nearly 90° from the sheath-like base; leaf laminas 6.5–27.6 × 1.2–2.2 mm, linear, obtuse, usually callous-like tipped at the apex, entire, flat to elliptical in cross section, glabrous, 1-nerved above, 1-nerved beneath, fleshy, matte or shiny. ***Capitula*** radiate, erect, sessile to subsessile (rarely with a short peduncle up to 8 mm long). ***Involucres*** 10.2–19.7 × 7.8–15.8 mm, cupuliform; involucral bracts 11 to 21, 6–14.9 × 1.6–2.2 mm, rather acute at the apex, greenish to dark-purplish. ***Ray florets*** 13 to 21; corollas 13–19.7 × 1.7–3.4 mm, 4 to 6-veined, subentire to 3-toothed at the apex, conspicuously surpassing the involucre, white. ***Disc florets*** 50 to 95; corollas 6.6–8.1 mm long, yellow; style branches truncate with a crown of sweeping trichomes, yellowish, purple-tipped. ***Achenes*** 2.6–4 × 0.8–1 mm, cylindrical, 6 to 8-ribbed, glabrous; pappus 7.8–19 mm long, barbellate, whitish. Chromosome number unknown.

#### Notes.

Funk (1997a) stated that the holotype of the name *Werneria crassa* S.F.Blake (US-1141291) may had been destroyed while on loan to B, and accordingly, she designated the duplicate at K as the lectotype (Turland et al. 2018, ICN Art. 9.3).

### Key to the subspecies of *Xenophyllum crassum*

**Table:** 

1	Leaf laminas 14.7–27.6 mm long; involucral bracts 16 to 21, 8.6–14.9 mm long; ray florets 16 to 21; disc florets ca. 95	**7a. X. crassum subsp. crassum**
–	Leaf laminas 6.5–12.7 mm long; involucral bracts 11 to 13, 6–8.2 mm long; ray florets 13 to 15; disc florets ca. 50	**7b. X. crassum subsp. orientale**

### 7a. Xenophyllum crassum subsp. crassum

Taxon classificationAnimaliaAsteralesAsteraceae

29632815-299B-5A52-BDDC-5EBDBCAFC7E7

#### Description.

**Figure 13.**
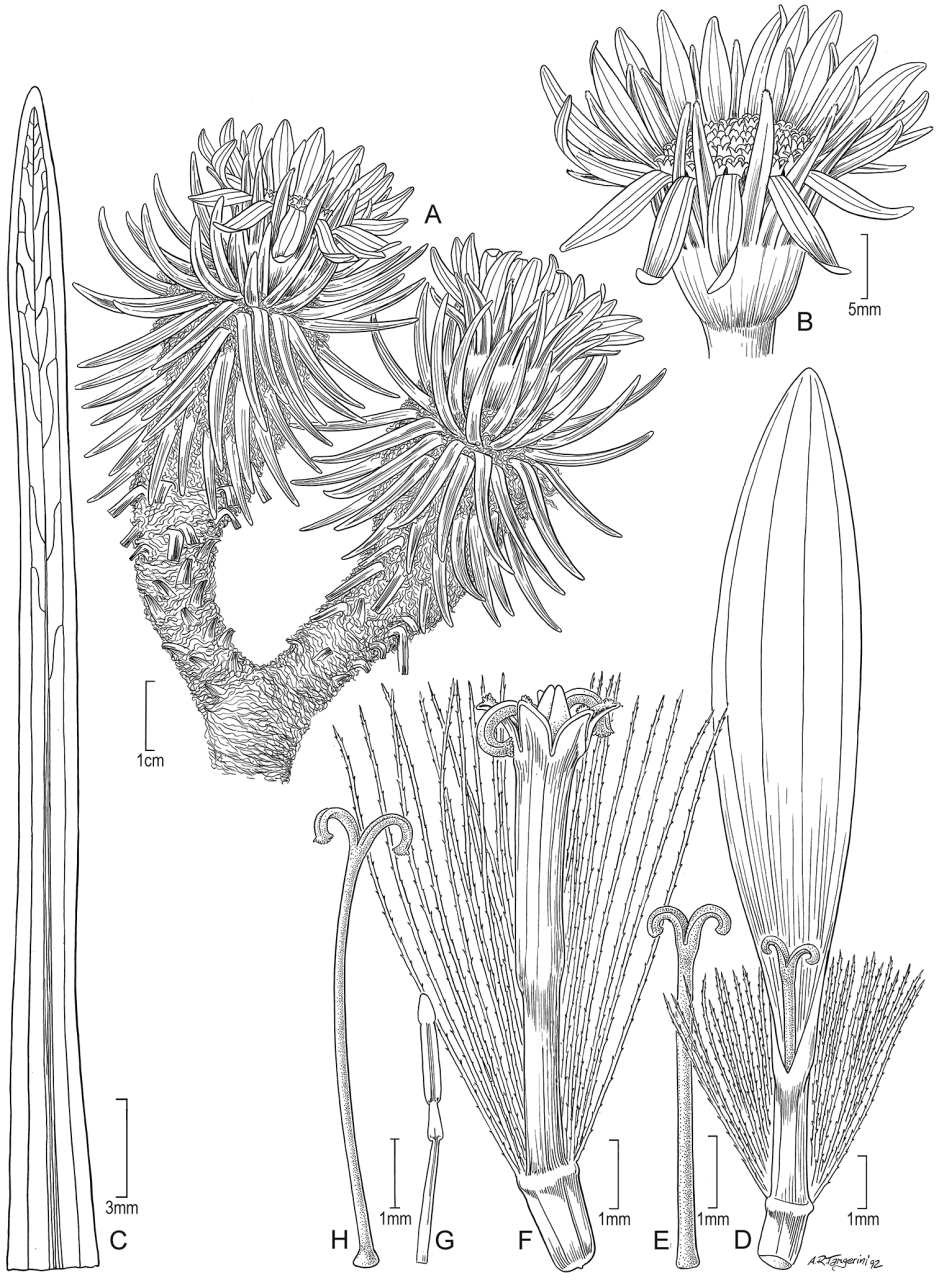
Xenophyllum crassum subsp. crassum**A** habit **B** capitulum **C** adaxial leaf surface **D** ray floret (frontward bristles removed) **E** style of ray floret **F** disc floret (frontward bristles removed) **G** stamen **H** style of disc floret. All details drawn from *Funk & Gavilanes 11070* (US). Illustration by Alice Tangerini.

***Stems*** 2.5–4 cm tall (aerial part), lanate. ***Leaves*** stellate-imbricate, extending into a sheath-like base that bears long silky trichomes, usually with the leaf lamina spreading at nearly 90° from the sheath-like base; leaf laminas 14.7–27.6 × 1.2–2.2 mm, linear, obtuse, usually callous-like tipped at the apex, entire, flat to elliptical in cross section, glabrous, 1-nerved above, 1-nerved beneath, fleshy, rather matte. ***Capitula*** radiate, erect, sessile to subsessile. ***Involucres*** 12.6–19.7 × 11–15.8 mm, cupuliform; involucral bracts 16 to 21, 8.6–14.9 × 1.5–2.2 mm, acute at the apex, greenish to dark-purplish. ***Ray florets*** 16 to 21; corollas 14.5–19.7 × 2.9–3.4 mm, 4 to 6-veined, subentire to 3-toothed at the apex, conspicuously surpassing the involucre, white. ***Disc florets*** ca. 95; corollas 6.5–8.1 mm long, yellow. ***Achenes*** 2.6–4 × 0.8–1 mm, cylindrical, 6 to 8-ribbed, glabrous; pappus 14–19 mm long, barbellate, whitish. Chromosome number unknown. Fig. 13.

#### Additional iconography.

Funk (1997b: 33, fig. 3A, sub *X. crassum*).

#### Distribution and habitat.

Central Colombia to central Ecuador. Colombia (Caldas, Cauca, Nariño, Risaralda, Tolima, Quindío, Valle del Cauca), Ecuador (Carchi, Chimborazo, Cotopaxi [expected], Morona-Santiago [expected], Napo, Pichincha, Tungurahua). It grows in marshes and moist grasslands of the paramo ecoregion, between elevations of 3125–4500 m (Fig. 12).

#### Phenology.

Flowering nearly all year round.

#### Etymology.

The adjective *crassus -a -um* means thick and it refers to the robust rhizome that this species displays.

#### Notes.

This taxon forms dense mats or hummocks and it is characterized by its linear, apically obtuse leaves, which are flat to elliptical in cross section and prominently 1-nerved on both faces. The rhizome-like stems are robust, long, and covered with a matted lanate indumentum that covers the leaf bases.

This subspecies can be differentiate from X. crassum subsp. orientale by the leaf length (14.7–27.6 mm vs. 6.5–12.7 mm in subsp. orientale), number and length of the involucral bracts (16 to 21, 8.6–14.9 mm vs. 11 to 13, 6–8.2 mm in subsp. orientale), number of ray florets (16 to 21 vs. 13 to 15 in subsp. orientale), number of disc florets (ca. 95 vs. ca. 50 in subsp. orientale), and pappus length (14–19 mm vs. 7.8–8 mm in subsp. orientale). Both taxa have a well defined distribution area. The typical subspecies grows along the Cordillera Central and Cordillera Occidental in Colombia and extends southwards to central Ecuador. In contrast, X. crassum subsp. orientale is restricted in the northern part of the Cordillera Oriental of Colombia.

The habit of X. crassum subsp. crassum also resembles that of *X. rigidum*, but X. crassum subsp. crassum differs in having longer and narrower leaves (14.7–27.6 × 1.2–2.2 mm vs. 12.6–13.5 × 2.3–2.4 mm in *X. rigidum*), higher number of involucral bracts and ray florets (16 to 21 vs. ca. 13 in *X. rigidum*), and by displaying glabrous achenes (vs. white-villous in *X. rigidum*). Their distribution areas only overlap in the Antisana Volcano (Napo, Ecuador).

#### Additional specimens examined.

**Colombia. Caldas**: páramo del Ruiz, middle slopes, 30 Aug 1957, *H.G. Barclay 5285* (COL); Cordillera Central, páramo de Las Letras, SW of Letras, 5°2'N, 75°19'W, 4 Dec 1958, *H.G. Barclay & P. Juajibioy 6245* (COL, US); Manizales, N.P. Los Nevados, Casa del Cisne, 4°51'N, 75°21'W, 19 Sep 1999, *D. Stancik 3381* (COL); Villa María, P.N.N. Nevados, cerca al refugio El Cisne, 4°50'N, 75°22'W, 28 Nov 2010, *W.G. Vargas 22382* (COL, ICESI); **Cauca**: P.N. Puracé, 2°20'N, 76°24'W, 31 Aug 1972, *H. Sánchez 351* (CUVC); **Nariño**: Cumbal, volcán de Chiles, en la antena vía El Laurel, Maldonado-Carchi, 0°49'N, 77°55'W, Jul 2012, *F. Ávila 2232* (UDBC); Cumbal, volcán de Chiles, en la antena vía El Laurel, Maldonado-Carchi, 0°49'N, 77°55'W, Jul 2012, *F. Ávila 2236* (UDBC); **Quindío**: páramo Quindío, Oct 2004, *W.G. Vargas 15711* (COL, ICESI); **Risaralda**: Santuario-San José del Palmar, serranía de Tatamá, P.N.N. Tatamá, sectores Mirlas, Piedra Bomba, Frailejonal, Lagunas y Ventanas, acceso desde Santuario, 5°6'N, 76°3'W, 16 Jul 2009, *J. Betancur, F. Gómez & J.N. Gómez 14253* (COL); Pereira, alrededores de la laguna de Otún, 4°46'N, 75°25'W, 3 Feb 1980, *S. Díaz-Piedrahita, H. Valencia & R. Jaramillo 19700* (COL); Santuario, vereda Las Colonias, 400 m arriba la cascada, 5°7'N, 76°2'W, 3 Feb 1983, *J.H. Torres et al. 1633* (COL); Santuario, macizo de Tamaná, cerca al campamento “El Reposo”, 5°9'N, 76°4'W, 7 Feb 1983, *J.H. Torres et al. 1663* (COL); Macizo de Tamaná, valle de San Francisco, cerca al campamento, 14 Feb 1983, *J.H. Torres et al. 1944* (COL); Pereira, P.N.N. Nevados, laguna del Otún, 4°46'N, 75°25'W, 20 Nov 2008, *W.G. Vargas 19700* (ICESI); Pereira, laguna del Otún, 4°46'N, 75°25'W, 28 Oct 2009, *W.G. Vargas 21036* (COL, ICESI); **Tolima**: Cordillera Central, Nevado del Ruiz, páramos entre Termales y Nevado y Líbano, above turn-off of road to Líbano, 4°56'N, 75°20'W, 18 Dec 1958, *H.G. Barclay & P. Juajibioy 6409* (COL); Santa Isabel, camino del paso de la quebrada del África, 4°45'N, 75°22'W, 7 Feb 1980, *S. Díaz-Piedrahita & R. Jaramillo 1918* (COL); road on N side of Nevado del Ruiz, near fork in road that leads to El Rosario, approx. 8 km E of intersection with road to Manizales, 4°55'N, 75°17'W, 23 Jan 1986, *V.A. Funk 8044* (COL); road on N side of Nevado del Ruiz, near fork in road that leads to El Rosario, approx. 8 km E of intersection with road to Manizales, 4°55'N, 75°17'W, 23 Jan 1986, *V.A. Funk 8046* (COL); Cajamarca, corregimiento Anaime, La Castellana, 5 Jun 1994, *B. Restrepo 430* (COL); Cajamarca, páramo de Anaime, reserva Semillas de Agua, 4°15'N, 75°33'W, 6 Jun 2000, *W.G. Vargas 8108* (ICESI); **Valle del Cauca**: Riofrío, Venecia, páramo El Duende, 4°4'N, 76°30'W, 3 Jun 2010, *J.C. Benavides 4859* (COL); Los Farallones, cuenca del río Timba, vert. oriental, quebrada Valle Escondido, 3°18'N, 76°44'W, 23 Aug 1991, *E. Calderón 88A* (COL); cabeceras de los ríos Tuluá y Bugalagrande, páramo de Las Vegas, 4°4'N, 75°47'W, 22 Mar 1946, *J. Cuatrecasas 20322* (CONC); Pradera, I.P. Bolo Azul, finca La Cabaña, 3°26'N, 76°3'W, 28 Nov 1989, *S. Sarria 591* (COL, CUVC); Palmira, La Nevera, 3°31'N, 76°4'W, 23 Mar 2008, *W.G. Vargas 18840* (ICESI); Tuluá, corregimiento de Barragán, 4°0'N, 75°48'W, 21 Nov 1997, *W.G. Vargas 4201* (COL). **Ecuador. Carchi**: road between Tulcán and Maldonado, S of volcán Chiles, 0°47'N, 77°58'W, 12 Mar 1985, *B. Eriksen 59010* (QCA, QCNE); paramo El Ángel, between towns of El Ángel and Tulcán near the pass, 4 Mar 1992, *V.A. Funk & M. Gavilanes 11069* (QCA, QCNE); paramo El Ángel, between towns of El Ángel and Tulcán near the pass, 4 Mar 1992, *V.A. Funk & M. Gavilanes 11070* (QCA, QCNE, US); volcán Chiles, rd. from Tulcán to Maldonado, 36 km W of the bridge at the W edge of Tulcán, 5 Mar 1992, *V.A. Funk & M. Gavilanes 11073* (QCA, QCNE); volcán Chiles, rd. from Tulcán to Maldonado, 38 km W of the bridge at the W edge of Tulcán, 5 Mar 1992, *V.A. Funk & M. Gavilanes 11077* (QCA, QCNE); Espejo, reserva ecológica El Ángel, sitio de lagunas El Voladero, 0°42'N, 77°53'W, 31 Oct 1993, *W. Palacios 11680* (QCNE); S slopes of volcán Chiles, 0°49'N, 77°57'W, 21 Oct 1987, *P.M. Ramsay & P.J. Merrow-Smith 869* (QCA, QCNE); páramo del Ángel, lado ecuatoriano del volcán Chiles, 0°48'N, 77°56'W, May 1989, *O. Rangel et al. 4406* (COL, US); SW side of volcán Chiles, 0°48'N, 77°57'W, 20 Jun 1995, *P. Sklenář & V. Kostečková 37-8* (QCA); SW side of volcán Chiles, 0°48'N, 77°57'W, 22 Jun 1995, *P. Sklenář & V. Kostečková 48-1* (QCA); volcán Chiles, SW side of the volcano, 0°48'N, 77°57'W, 23 Jun 1995, *P. Sklenář & V. Kostečková 713* (QCNE); Espejo, reserva ecológica El Ángel, asociación 23 de Julio, 0°43'N, 77°55'W, 3 Aug 2003, *D. Suárez, M. Chinchero & M. Cabascango 1355* (QCNE); **Chimborazo**: Altar, hacienda Releche, sector Pashuazo, 1°40'S, 78°27'W, 23 Jul 2009, *D. Cárate, F. Naranjo & S. Chiriboga 1054* (QCA); Atillo, frente al campamento de ingenieros del ejército, 2°10'S, 78°30'W, 12 Apr 2009, *D. Cárate, J. Salvador & S. Rojas 151* (QCA); **Napo**: road Quito-Baeza, near the pass at Papallacta, 0°20'S, 78°12'W, 30 Oct 1983, *B. Eriksen & B.B. Larsen 45435* (QCA, QCNE); páramo de Papallacta, sector El Paso, 0°19'S, 78°12'W, 28 Oct 1984, *A. Freire 27* (QCA); Pisayambo, laguna Cochas Negras, 1°6'S, 78°19'W, 14 Jan 1999, *B. Merino & Á. Sánchez s.n.* (LOJA [mixed with *X. humile*]); Llanganati, páramo SE of choza Aucacocha, between Aucacocha and Pan de Azúcar, 1°9'S, 78°18'W, 15 May 1982, *B. Øllgaard et al. 38514* (QCA); reserva ecológica Oyacachi, 0°15'S, 78°5'W, 29 Sep 2007, *K. Romoleroux et al. 4642* (QCA); La Virgen de Papallacta, a 50 m desde la vía al Tena, hacia el interior del páramo, 0°21'S, 78°11'W, 31 Mar 2010, *C.V. Sandoya-Sánchez, E. Gortaire & J. Irazábal 335* (QCA); NE side of volcán Antisana, 0°27'S, 78°8'W, 18 Aug 1997, *P. Sklenář & V. Sklenářová 3559* (QCA); Tena, P.N. Llanganates, vía Salcedo-Tena, de laguna Chaloa Cocha desvío a Rayo Filo, 0°57'S, 78°23'W, 20 Sep 1998, *H. Vargas, E. Narváez & S. Orellana 2630* (QCNE); **Pichincha**: Cordillera Oriental, cerro de Corrales, páramo de Guamaní, on north side of boquerón, highest point of road to Papallacta, 0°19'S, 78°12'W, 16 Aug 1959, *H.G. Barclay & P. Juajibioy 8855* (COL); Quito, embalse Salve Faccha, 0°20'S, 78°15'W, 31 Aug 2001, *D. Fernández, G. Pérez & L. Calvopiña 416* (QCNE); páramo de Guamaní, carretera Quito-Pifo-Papallacta, 0°23'S, 78°9'W, 20 Oct 1990, *E. Guerrón 15* (QCA); páramo de Guamaní, close to paso de La Virgen, 0°20'S, 78°13'W, 8 Feb 1984, *S. Laegaard 51328* (QCA); páramo de Guamaní, paso de la carretera Quito-Baeza, 0°19'S, 78°12'W, 25 Aug 1985, *B.B. Larsen & B. Dall 191* (QCA); páramo de Guamaní, carretera Pifo-Papallacta, km 23, 0°18'S, 78°14'W, 4 Nov 1990, *S. León 1012* (LOJA, QCA); páramo de Guamaní, laguna de Hoyas, 0°15'S, 78°12'W, 8 Aug 1987, *P.M. Ramsay & P.J. Merrow-Smith 140* (QCA); páramo de Guamaní, on the right of the road Quito-Papallacta, 0°20'S, 78°12'W, 28 Jun 1997, *P. Sklenář & V. Sklenářová 2010* (QCA); páramo de Guamaní, along the road to the antennas, 0°19'S, 78°12'W, 19 Jun 1999, *P. Sklenář 7314* (QCA); N side of nevado Cayambe, on the right side along the road towards the military antennas, 0°5'N, 77°58'W, 6 Aug 2004, *P. Sklenář 8175* (QCA); **Tungurahua**: P.N. Llanganates, laguna Cable, 1°8'S, 78°18'W, 28 Nov 1996, *J.L. Clark & J. Fair 3521* (QCNE); Llanganates, entre bordes de Mesa Tablón y entrada de Aucacocha, 1°9'S, 78°19'W, 27 Dec 1983, *J. Jaramillo, V. Zak & J. Yépez 6004* (F, QCA); Aucacocha, sector NE de la laguna Aucacocha, 1°9'S, 78°19'W, 29 Dec 1983, *J. Jaramillo 6091* (QCA); Aucacocha, sector NE de la laguna de Aucacocha, 1°9'S, 78°19'W, 30 Dec 1983, *J. Jaramillo, V. Zak & J. Yépez 6262* (QCA); Santiago de Píllaro, P.N. Llanganates, base of cerro Hermoso on W side, near lake, 1°13'S, 78°17'W, 13 Nov 1999, *D. Neill et al. 12049* (QCNE); Santiago de Píllaro, P.N. Llanganates, SW flank of cerro Hermoso, above lake, 1°13'S, 78°17'W, 14 Nov 1999, *D. Neill et al. 12064* (QCA, QCNE); Patate, P.N. Llanganates, alrededores de la laguna Pan de Azúcar, 1°9'S, 78°17'W, 14 Oct 1998, *H. Vargas, J.C. Ronquillo & N. Granda 2870* (QCNE).

### 7b. Xenophyllum crassum subsp. orientale

Taxon classificationAnimaliaAsteralesAsteraceae

(Cuatrec.) J.Calvo comb. nov.

EDF52A6C-C5F6-5763-9ECD-7D217E59E3DF

urn:lsid:ipni.org:names:77211291-1

Werneria crassa subsp. orientalis Cuatrec., Phytologia 45: 29. 1980. Type. Colombia. Santander: páramo del Almorzadero, vertiente norte, 3600−3800 m, 28 Nov 1941, *J. Cuatrecasas 13511* (holotype: US-00037302!; isotypes: BC-634974 (digital image!), F-1233032!).
Werneria crassa f. minor Cuatrec., *nom. nud. in sched.* (Turland et al. 2018, ICN Art. 38.1).

#### Description.

***Stems*** 1.5–2.5 cm tall (aerial part), lanate. ***Leaves*** stellate-imbricate, extending into a sheath-like base that bears long silky trichomes, usually with the leaf lamina spreading at nearly 90° from the sheath-like base; leaf laminas 6.5–12.7 × 1.2–2 mm, linear, obtuse, usually callous-like tipped at the apex, entire, flat to elliptical in cross section, glabrous, 1-nerved above, 1-nerved beneath, fleshy, rather shiny (in dried specimens). ***Capitula*** radiate, erect, sessile to subsessile (rarely with a short peduncle up to 8 mm long). ***Involucres*** 10.2–12.2 × 7.8–10.6 mm, cupuliform; involucral bracts 11 to 13, 6–8.2 × 1.6–2.1 mm, acute to obtuse at the apex, greenish to dark-purplish. ***Ray florets*** 13 to 15; corollas 13–13.2 × 1.7–2.6 mm, 4 to 5-veined, subentire to 3-toothed at the apex, conspicuously surpassing the involucre, white. ***Disc florets*** ca. 50; corollas 6.6–6.9 mm long, yellow. ***Achenes*** cylindrical, glabrous (immature); pappus 7.8–8 mm long, barbellate, whitish. Chromosome number unknown.

#### Distribution and habitat.

Endemic to Colombia (Arauca [expected], Boyacá, Norte de Santander, Santander). It grows in marshes and wet exposed areas of the paramo ecoregion, between elevations of 3200–4600 m (Fig. 12).

#### Phenology.

Flowering nearly all year round.

#### Etymology.

The adjective *orientalis -is -e* means eastern, which refers to the further eastern distribution of this taxon in relation to the typical subspecies. It occurs in the northern part of the Cordillera Oriental of Colombia (Eastern Ranges).

#### Notes.

Xenophyllum crassum subsp. orientale is characterized by the leaf length (6.5–12.7 mm), the number of involucral bracts (11 to 13), and the number of ray florets (13 to 15). In dried specimens, the leaves are rather shiny. This taxon was synonymized with X. crassum subsp. crassum by Ávila et al. (2016) but no discussion supporting such taxonomic decision was provided. The aforementioned morphological differences against the typical subspecies, in addition to the fact that their distribution areas are well defined and do not overlap, lead us to maintain the treatment by Cuatrecasas (1980).

It should be noted that X. crassum subsp. orientale has been confused with *Werneria pygmaea*, a species that grows in the same habitat. Indeed, we detected several specimens that contain mixed material, including an isotype (see comments below). Xenophyllum crassum subsp. orientale has erect, robust rhizome-like stems whereas *W. pygmaea* is a strictly rosettiform species not forming hummocks. Further differences are detailed in Calvo et al. (in press). Some confusion with *X. humile* is also likely (see comments under it).

The presumed isotype of W. crassa subsp. orientalis Cuatrec. kept at COL (COL-000005514) comes from “extremo S en Peralonso, 3100 m”, a locality slightly different from that indicated in the protologue, i.e., “vertiente norte, 3700−3800 m” [as “3600−3800 m” on the labels]. It is excluded from the type material as it does not belong to the same gathering. On the other hand, it has to be noted that the isotype at BC contains an individual corresponding to *W. pygmaea* (above on the right hand, with fragments of *Disterigma* sp. (Ericaceae) at the base).

#### Additional specimens examined.

**Colombia. Boyacá**: Cordillera Oriental, Sierra Nevada del Cocuy, Alto Ritacuva, 6°30'N, 72°19'W, 11 Apr 1959, *H.G. Barclay & P. Juajibioy 7352* (COL, US); páramo de Monguí, laguna Colorada, 5°38'N, 72°50'W, 1 Sep 1998, *J.A. Calleja & J.C. Jaramillo 204* (COL); Sierra Nevada del Cocuy, alto valle Lagunillas, 500 m al NNE de la laguna Pintada, 6°22'N, 72°19'W, 3 Oct 1972, *A.M. Cleef & P.A. Florschütz 5784* (COL, US); Sierra Nevada del Cocuy, quebrada Bocatoma, morrena al W de la lagunita glaciar occidental, 6°23'N, 72°16'W, 5 Nov 1972, *A.M. Cleef & P.A. Florschütz 5870* (COL); Sierra Nevada del Cocuy, quebrada Bocatoma, laguna rellena, 6°23'N, 72°16'W, 3 Mar 1973, *A.M. Cleef 8736* (COL, US); Sierra Nevada del Cocuy, quebrada Bocatoma, orilla SE de la laguna rellena, 6°23'N, 72°16'W, 3 Mar 1973, *A.M. Cleef 8762* (COL, US); páramo de la Sarna entre Sogamoso y Vado Hondo, 5°35'N, 72°51'W, 5 Apr 1973, *A.M. Cleef 9411a* (COL); Nevado de Cocuy, alto valle de Las Lagunillas, 6°21'N, 72°19'W, 12 Sep 1938, *J. Cuatrecasas 1486* (COL, US); **Norte de Santander**: Chitagá, corregimiento Presidente, vereda Presidente, alrededores de la quebrada El Salado, en el ascenso a la terraza pantanosa entre las lagunas El Salado y El Tambor, 7°0'N, 72°42'W, 22 May 2007, *D.I. Capacho-Navia et al. 243* (COL); Chitagá, corregimiento Presidente, vereda Presidente, laguna El Tambor, sobre margen al costado SE, 7°0'N, 72°42'W, 15 Mar 2007, *D.I. Capacho-Navia et al. 362* (COL [mixed with *Werneria pygmaea*]); Cordillera Oriental, páramo del Almorzadero, vertiente norte, 6°59'N, 72°44'W, 28 Nov 1941, *J. Cuatrecasas 13505* (COL [mixed with *W. pygmaea*], US); **Santander**: páramo del Almorzadero, 6°59'N, 72°44'W, 17 Nov 1978, *J. Aguirre & A.M. Cleef 880* (COL); Cordillera Oriental, páramo de Almorzadero, on road between Chitagá and Cerrito, about half way to Boquerón, 6°56'N, 72°40'W, 31 Dec 1959, *H.G. Barclay & P. Juajibioy 10363* (COL); páramo de Almorzadero, on road between Chitagá and Cerrito, approx. 7 km S of highest point of paramo, 6°59'N, 72°40'W, 31 Dec 1959, *H.G. Barclay & P. Juajibioy 10370* (COL [mixed with *W. pygmaea*], US); Alto del Almorzadero, vertiente sur, 6°58'N, 72°43'W, 20 Jul 1940, *J. Cuatrecasas & H. García Barriga 10012* (COL, US); páramo del Almorzadero, km 50–51, 7°0'N, 72°44'W, 20 Sep 1969, *J. Cuatrecasas & L. Rodríguez 27887* (COL, US).

### 8. Xenophyllum marcidum

Taxon classificationAnimaliaAsteralesAsteraceae

(S.F.Blake) V.A.Funk, Novon 7(3): 240. 1997.

7D1FCF8D-C682-594E-A57C-07E540CDFCF1

Werneria marcida S.F.Blake, J. Washington Acad. Sci. 18: 492. 1928. Type. Peru. Lima: Río Blanco, 4570 m, 20/25 Mar 1923, *J.F. Macbride 3032* (holotype: F-534102!; isotypes: BM s.n.!, G-00305676 (fragment, digital image!), G-00305677 (digital image!), GH s.n.!, MA-246360!, S-R-6525 (digital image!), US-00037326!, W-3718!).
Werneria sedoides S.F.Blake, J. Washington Acad. Sci. 18: 493. 1928. Type. Peru. Ancash: Punco, Estación 21 miles West of Huallanca, 4115 m, 1 Oct 1922, *J.F. Macbride & W. Featherstone 2475* (holotype: F-518901!; isotypes: US-00037308!, G-00305678 (fragment, digital image!), G-00305679 (digital image!)).

#### Description.

**Figure 14.**
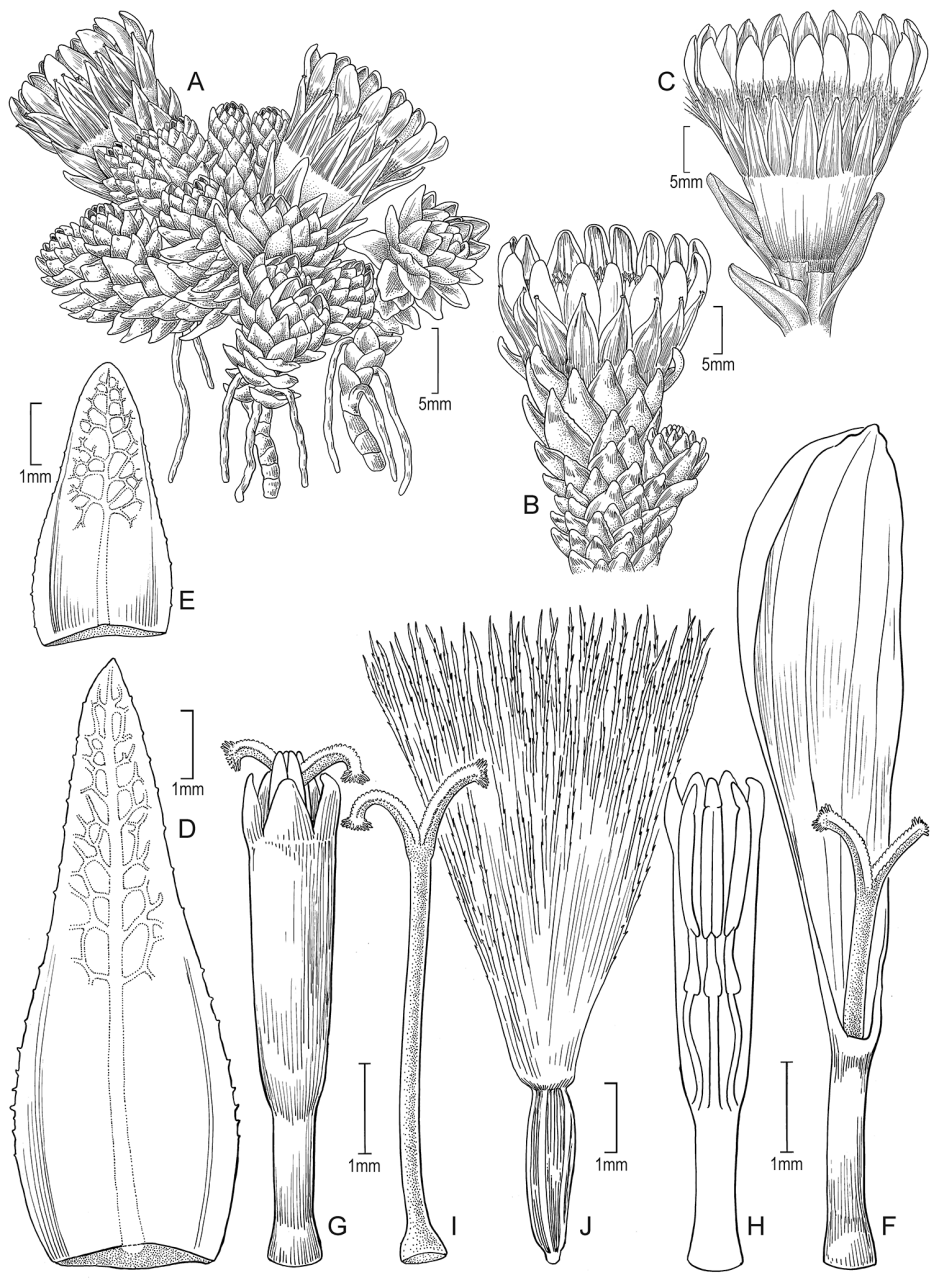
*Xenophyllum marcidum***A** habit **B** stem apical part and capitulum **C** capitulum **D** adaxial leaf surface **E** uppermost leaf **F** ray corolla and style **G** disc corolla and style branches **H** disc corolla and stamens (vertically sectioned, style removed) **I** style **J** achene with pappus. All details drawn from *Funk & Bernal 11285* (US) except for A (drawn from *Stafford 749*, K) and **C–E, H** (drawn from *Macbride & Featherstone 2475*, US). Illustration by Alice Tangerini.

Suffruticose plant, forming dense mats or hummocks, with rhizome-like stems up to 8 cm long covered with old leaves, rather erect, simple or branched from the base. ***Stems*** 1–2 cm tall (aerial part), glabrous. ***Leaves*** densely imbricate, extending into a glabrous sheath-like base; leaf laminas 3.3–7.6 × 1.7–2.6 mm, triangular, acute at the apex, denticulate in the upper half, rather flat in cross section, glabrous, 1-nerved above (barely visible), 1-nerved beneath, rather fleshy, matte. ***Capitula*** radiate, erect, sessile to subsessile. ***Involucres*** 8.8–10.8 × 5.7–10 mm, cupuliform; involucral bracts 13 to 15, 4.8–6.7 × 1.8–2.2 mm, acute at the apex, greenish to dark-purplish. ***Ray florets*** 14 to 21; corollas 7.4–12.9 × 1.6–2.3 mm, 3 to 4-veined, subentire to 2-toothed at the apex, conspicuously surpassing the involucre, white. ***Disc florets*** 35 to 48; corollas 5–6.2 mm long, yellowish; style branches truncate with a crown of sweeping trichomes, purplish. ***Achenes*** 2.8–3 × ca. 1 mm, cylindrical, ca. 7-ribbed, glabrous; pappus 5–8.8 mm long, barbellate, whitish to partially purple-colored. Chromosome number unknown. Fig. 14.

#### Additional iconography.

Blake (1928: 496, fig. 1H–J sub *Werneria marcida*, fig. 1N–P sub *W. sedoides*); Funk (1997a: 237, fig. 1B); Beltrán (2016: 358, fig. 2B, as photo).

#### Distribution and habitat.

**Figure 15.**
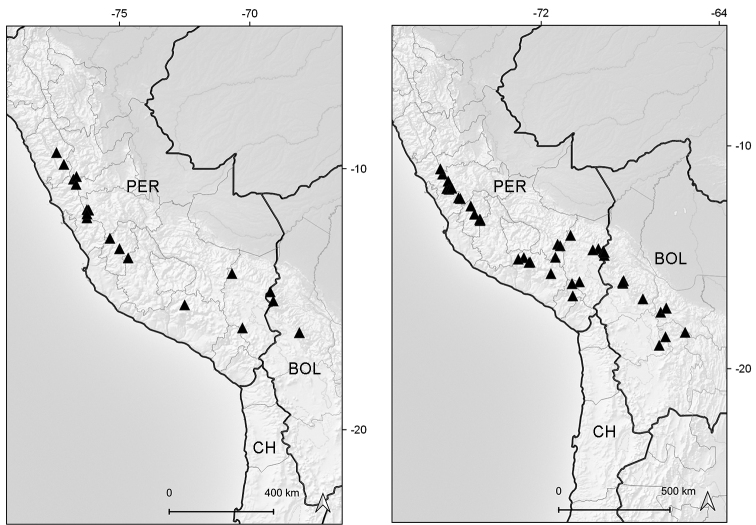
Distribution map of *Xenophyllum marcidum* (left hand) and *X. ciliolatum* (right hand).

Central Peru to northern Bolivia. Bolivia (La Paz), Peru (Ancash, Arequipa, Ayacucho, Huancavelica, Huánuco, Junín [expected], Lima, Moquegua [expected], Pasco [expected], Puno). It grows on exposed rocky slopes and cryoturbated soils of the subhumid and humid puna ecoregions, between elevations of 4550–5350 m (Fig. 15).

#### Phenology.

Flowering from February to September.

#### Etymology.

The adjective *marcidus -a -um* means withered. It probably refers to the fact that a large proportion of the stem bears marcescent leaves and the green ones are restricted to the distal part.

#### Notes.

*Xenophyllum marcidum* is characterized by its triangular, densely imbricate leaves. Among the *Xenophyllum* members forming dense mats or hummocks, this species is readily distinguishable due to the leaves are denticulate in the upper half, acute at the apex, and rather flat in cross section. The capitula are radiate and have 14 to 21 ray florets with white, considerably long corollas.

Because of the marginally denticulate leaves, on dried specimens *X. marcidum* might be confused with *X. ciliolatum*, a species that has a partially overlapping distribution area. They can be easily differentiated by the habit (forming dense mats or hummocks in *X. marcidum* vs. forming clumps of erect or decumbent stems in *X. ciliolatum*), leaf lamina shape (triangular in *X. marcidum* vs. linear in *X. ciliolatum*), number of involucral bracts (13 to 15 in *X. marcidum* vs. ca. 8 in *X. ciliolatum*), number of ray florets (14 to 21 in *X. marcidum* vs. 8 to 11 in *X. ciliolatum*), and by the ray corolla length and color (7.4–12.9 mm, white in *X. marcidum* vs. 6.2–7.3 mm, yellow in *X. ciliolatum*).

#### Additional specimens examined.

**Bolivia. La Paz**: Murillo, nev. Charquini, above aqueduct on N facing slope, 16°17'S, 68°6'W, 12 Apr 1995, *V.A. Funk & N. Bernal 11285* (LPB, US); Bautista Saavedra, Charazani, miterhalb Medallani, 15°4'S, 69°6'W, 17 Feb 1994, *B. Herzog 795* (LPB); Murillo, subida del paso de Zongo (acueducto), al nev. Charquini, 16°17'S, 68°6'W, 30 Jul 1982, *X. Menhofer 1461* (LPB, US); Murillo, paso de Zongo, nev. Charquini, 16°17'S, 68°6'W, 1 Aug 1982, *X. Menhofer 1461A* (LPB); Franz Tamayo, Pelechuco, al N en línea recta a 0.58 km del campamento Chocollo, 14°43'S, 69°13'W, 25 Nov 2017, *F. Zenteno, D. Villalba & L. Mamani 21274* (LPB). **Peru. Ancash**: Recuay, carretera a Pachacoto, abra de Yanashallash, 9°50'S, 77°8'W, 28 May 2001, *A. Cano 11496* (USM); Carhuaz, Huascarán N.P., lateral valley of quebrada Ishinca, trail to lago Ishinca, 9°23'S, 77°25'W, 12 Feb 1985, *D.N. Smith, R. Valencia & A. Gonzales 9469* (LPB, MO, QCA, USM); collado sobre el río Pumapampa, 18 Mar 1983, *O. Tovar et al. 9627* (USM); collado encima río Pumapampa, 18 Mar 1983, *O. Tovar et al. 9666* (USM); nevado de Cajat, entre Huaraz-La Unión, 22 Mar 1983, *O. Tovar et al. 9842* (USM); **Arequipa**: Castilla, Orcopampa, minas de Poracota, quebrada Huamanihuayta, 15°13'S, 72°30'W, 20 Apr 2011, *H. Beltrán 7101* (USM); **Ayacucho**: Ayacucho, abra Apacheta, camino a Pars, 13°25'S, 74°40'W, 4 Aug 2010, *A. Cano, W. Mendoza & A. Delgado 19760* (USM [mixed with *Werneria orbignyana* Wedd.]); **Huancavelica**: Huachocolpa, alrededores de la unidad minera Caudalosa, 13°4'S, 75°0'W, 23 Mar 2015, *P. Gonzáles 3544* (USM); San José de Acobambilla, top of quebrada Ocrococha, 12°40'S, 75°22'W, 12 Aug 1961, *J.R. Lloyd & J.K. Marshall 309* (K); **Huánuco**: Dos de Mayo, Lauricocha, 10°18'S, 76°39'W, 1955, *A. Cardich 208* (USM); Dos de Mayo, Gayco, 10°24'S, 76°46'W, Apr 1956, *A. Cardich 217* (USM); **Lima**: Huarochirí, desvío de carretera central hacia Chinchan y Marcapomacocha, 11°34'S, 76°15'W, 23 Sep 2014, *H. Beltrán & W. Aparco 7750* (USM); Oyón, encima de mina Uchuchacua, parte más alta por carretera, 10°36'S, 76°40'W, 5 Apr 2015, *H. Beltrán & W. Aparco 7769* (USM); Huarochirí, Chicla, abra Anticona (Ticlio), 11°35'S, 76°11'W, 29 Apr 2017, *H. Beltrán, S. Castillo & M. Arakaki 7986* (USM); Ticlio bajo, 11°35'S, 76°11'W, 16 May 1959, *L. Diers 940* (USM); Huarochirí, San Damián, Chanape, 11°53'S, 76°15'W, 5 Jul 2013, *P. Gonzáles & B. Brito 2632* (USM); Anticona, pequeño arroyo con orientación SW, 11°35'S, 76°11'W, 5 Aug 2012, *E. Linares & A. Galán 3077* (USM); **Puno**: Carabaya, Corani, Minaspata, 14°1'S, 70°41'W, 15 Oct 2017, *P. Gonzáles 3832* (USM); San Antonio de Esquilache, 16°6'S, 70°17'W, 18 May 1937, *D. Stafford 749* (K).

### 9. Xenophyllum ciliolatum

Taxon classificationAnimaliaAsteralesAsteraceae

(A.Gray) V.A.Funk, Novon 7(3): 239. 1997.

356400F8-BD18-5C33-96A1-CA68B13E94C4

Werneria ciliolata A.Gray, Proc. Amer. Acad. Arts 5: 140. 1861. Type. Peru. Junín: Andes Peru, Casa Cancha, [without date], *Capt. Wilkes Expedition s.n.* (lectotype: US-00037299!, designated by Calvo et al. (2017: 229); isolectotype: GH-00967832 (fragment, digital image!)).
Werneria ciliata Wedd. ex Sch.Bip., Linnaea 34: 530. 1866, *nom. inval. pro syn.* (Turland et al. 2018, ICN Art. 36.1).

#### Description.

Suffruticose plant, forming clumps of erect or decumbent stems. ***Rhizomes*** 5–8 × 0.3–0.5 cm, horizontal to oblique, glabrous. ***Stems*** 4–7 cm tall, simple or branched, glabrous, with leaves usually clustered in the upper part. ***Leaves*** imbricate, erect (not adpressed to the stem), extending into a glabrous sheath-like base; leaf laminas 4.9–8.6 × 0.6–1.2 mm, linear, rather acute at the apex, denticulate, elliptical to slightly curved forwards in cross section, glabrous, 1-nerved above (barely visible), 1-nerved beneath (sometimes purple-colored), rather fleshy, matte. ***Capitula*** radiate, erect, sessile. ***Involucres*** 8.9–12.4 × 4–7.3 mm, narrowly cupuliform; involucral bracts ca. 8, 4.1–7.7 × 1.4–2.5 mm, acute at the apex, greenish to dark-purplish. ***Ray florets*** 8 to 11; corollas 6.2–7.3 × 0.8–1.2 mm, 3-veined, subentire to 2-toothed at the apex, not surpassing the involucre, yellow. ***Disc florets*** 11 to 28; corollas 5–7.3 mm long, yellow; style branches truncate with a crown of sweeping trichomes, yellowish. ***Achenes*** 2–3.3 × 1–1.2 mm, cylindrical, 7 to 8-ribbed, glabrous; pappus 6.2–7.4 mm long, barbellate, whitish. Chromosome number unknown.

#### Iconography.

Beltrán (2016: 358, fig. 2E, as photo).

#### Distribution and habitat.

Central Peru to central Bolivia. Bolivia (Cochabamba, La Paz, Oruro, Potosí), Peru (Arequipa, Ayacucho, Cusco, Huancavelica, Junín, Lima, Moquegua, Pasco [expected], Puno). This species grows in rocky outcrops, scree slopes, cryoturbated soils, and exposed grasslands of the subhumid and humid puna ecoregions, between elevations of 3900–5350 m (Fig. 15).

#### Phenology.

Flowering from February to November.

#### Etymology.

The adjective *ciliolatus -a -um* is the diminutive of *ciliatus -a -um*, which describes the denticulate leaf margin of this species.

#### Notes.

*Xenophyllum ciliolatum* is one of the species displaying yellow ray corollas not surpassing the involucre. It is characterized by having erect or decumbent stems 4–7 cm tall, linear leaves 4.9–8.6 mm long with denticulate margin, ca. 8 involucral bracts, and 8 to 11 ray florets. The leaves are erect (not adpressed to the stem) and tend to be clustered upwards.

The morphologically closest species is *X. juniperinum*, known from southern Peru (Tacna), northern Chile, and western Bolivia (Oruro and Potosí). They differ in habit (erect or decumbent stems 4–7 cm long in *X. ciliolatum* vs. erect stems 8–15 cm long in *X. juniperinum*), leaf disposition (clustered up the stem and erect in *X. ciliolatum* vs. uniformly arranged along the stem and adpressed in *X. juniperinum*), leaf lamina shape (linear in *X. ciliolatum* vs. linear-triangular in *X. juniperinum*), leaf margin (denticulate in *X. ciliolatum* vs. minutely, irregularly denticulate in *X. juniperinum*), and involucral bract length (4.1–7.7 mm in *X. ciliolatum* vs. 2.8–3.4 in *X. juniperinum*). Their distribution areas do not overlap. *Xenophyllum ciliolatum* also shows morphological affinities with *X. marcidum* (see comments under it) and *X. weddellii*. From this latter species, *X. ciliolatum* differs in having denticulate leaf margin (vs. entire in *X. weddellii*), yellow ray corollas not surpassing the involucre (vs. white and conspicuously surpassing the involucre in *X. weddellii*), and disc corollas 5–7.3 mm long (vs. 4.5–5.2 mm long in *X. weddellii*). These species co-occur in southern Peru (Arequipa, Moquegua, Puno).

The lectotype designation of the name *Werneria ciliolata* A.Gray by Calvo et al. (2017) should be rather attributed to Funk (1997a) although she used the term “holotype”. It is here corrected to lectotype because it cannot be established that the author only used this element and that the gathering is represented by a single specimen (McNeill 2014).

#### Additional specimens examined.

**Bolivia. Cochabamba**: cordillera del Tunari, laderas de la cumbre del Tunari, 17°17'S, 66°23'W, 25 Mar 1990, *G. Navarro 596* (BOLV); cordillera del Tunari, laderas altas de la cumbre del Tunari, 17°17'S, 66°23'W, 25 Mar 1990, *G. Navarro 628* (BOLV); cordillera del Tunari, cumbres del pico Tunari, 17°17'S, 66°23'W, 18 Feb 1990, *G. Navarro 637* (BOLV); Quillacollo, cordillera alta del Tunari, cima de la Falso Tunari, 17°28'S, 66°38'W, 22 Aug 2010, *M. Zárate 3770* (BOLV); **La Paz**: Los Andes, subiendo cerca de Peñas, por las lagunas de Hichucota, hacia la cumbre al lado del glacial Ankokota, 16°3'S, 68°18'W, 22 May 1999, *S.G. Beck 24639* (LPB); Los Andes, above cumbre (pass) on rd. through Hichu-Kkota valley on rd. to mina La Fabulosa, 21 km from base of lag. Khara Kkota, 16°10'S, 68°20'W, 29 Apr 1995, *V.A. Funk 11403* (LPB, US); Franz Tamayo, Ulla Ulla, cordillera Apolobamba cerca al pueblo de Pelechuco 17 km NE, 14°55'S, 69°10'W, 22 Aug 1986, *P. Holt 14A* (LPB); Franz Tamayo, Ulla-Ulla, nev. Manuel Llipani, 20 Jul 1982, *X. Menhofer 10* (LPB); Franz Tamayo, paso de Pelechuco, 14°47'S, 69°10'W, 22 Jul 1982, *X. Menhofer 23* (LPB); Omasuyos, Hichu Cota, subiendo a la cumbre, 16°7'S, 68°21'W, 28 Apr 1985, *M. Moraes 153* (LPB); Loayza, Pata Mina-Viloco, 17 Aug 1982, *O. Murgia 390* (LPB); Los Andes, valle de Hichu Kkota, 17 Nov 1983, *C. Ostria 54* (LPB); Loayza, bajando de Viloco hacia Choquetanga, 16°52'S, 67°26'W, 15 Aug 1994, *N. Salinas et al. 3294* (LPB); Franz Tamayo, Pelechuco, al N en línea recta a 3.8 km del campamento Chocollo, 14°43'S, 69°13'W, 25 Nov 2017, *F. Zenteno, D. Villalba & L. Mamani 21275* (LPB); **Oruro**: Eduardo Abaroa, Challapata, desde Challapata subiendo hasta el final del camino hacia Azanaque, luego subiendo hasta las cumbres del Azanaque, 18°57'S, 66°42'W, 29 May 2016, *I. Jiménez 8406* (LPB); **Potosí**: Colquechaca, 18°22'S, 65°32'W, 1 Feb 2010, *F.S. Zenteno 16389* (USM); Azanaque, 18°34'S, 66°25'W, 3 May 2016, *F.S. Zenteno 17034* (USM). **Peru. Arequipa**: pr. Chivay, nevado Huarancante, 15°44'S, 71°34'W, 3 Apr 2005, *C. Aedo & A. Galán 11103* (MA); Castilla, Orcopampa, minas de Poracota, quebrada Sora, 15°13'S, 72°30'W, 20 Apr 2011, *H. Beltrán 7100* (USM); Castilla, Orcopampa, minas de Poracota, cerca a quebrada Faculla, 15°14'S, 72°33'W, 20 Apr 2011, *H. Beltrán 7111* (USM [mixed with *X. digitatum*]); La Unión, Huaynacotas, Sarajorepampa, 15°1'S, 72°47'W, 18 Mar 2011, *D. Montesinos 2950* (HSP, MOL, USM); Castilla, Tapay, cerro Blanco, Apacheta, 14 Sep 2011, *N. Vega 1744* (USM); **Ayacucho**: Páucar del Sara Sara, Oyolo, a 15 km al NO de Pampamarca, camino a Sayla, 15°5'S, 73°2'W, 14 Sep 2013, *C. Tejada 226* (HSP); **Cusco**: Espinar, hacienda K’achachi (Uchupata), 15°0'S, 71°22'W, 22 Jun 1956, *C. Vargas 11223* (US); Canas, alturas de Layo, 14°29'S, 71°9'W, 12 Aug 1957, *C. Vargas 11871* (US); Canas, Langui, alturas, 14°25'S, 71°16'W, 14 Sep 1961, *C. Vargas 13620* (LPB, US); **Huancavelica**: Huamanga, Licapa, 13°18'S, 74°45'W, 29 Oct 2009, *A. Cano et al. 19436* (USM); Huaytará, Pilpichaca (abra Apacheta), 13°18'S, 74°46'W, 4 Jul 2010, *A. Cano, W. Mendoza & A. Delgado 19658* (USM); Huaytará, Pilpichaca (abra Apacheta), 13°21'S, 74°45'W, 4 Jul 2010, *A. Cano, W. Mendoza & A. Delgado 19721* (USM); Huaytará, Pillpichaca (Llillinta-Ingahuasi), 13°18'S, 74°45'W, 26 Jun 2010, *A. Cano et al. 19849* (USM); Huachocolpa, alrededores de la unidad minera Caudalosa, 13°4'S, 75°0'W, 23 Mar 2015, *P. Gonzáles 3545* (USM); Huaytará, 7 km lineales al NE del abra Apacheta, en el límite entre Huancavelica y Ayacucho, distr. Pilpichaca, 13°18'S, 74°46'W, 11 Apr 2005, *J. Roque 4799* (USM); Huaytamayoc-Tansiri, 12°42'S, 75°10'W, May 1956, *O. Tovar 2560* (USM); **Junín**: Anticona, abajo entre Casapalca y Oroya, 11°35'S, 76°11'W, 26 Jun 1954, *O. Tovar & L. Constance 9194* (USM-29378 [mixed with *X. decorum* and *X. digitatum*]); Yauli, Ticlio, 11°35'S, 76°11'W, 28 Jun 1999, *G. Yarupaitán 1688* (US); **Lima**: Yauyos, Laraos, camino Jalcacha a Palca, 12°20'S, 75°43'W, 25 May 1995, *H. Beltrán 1702* (USM); Huaral, Pacaros, abra de Antajirca, límite entre Lima y Pasco, 11°2'S, 76°32'W, 20 Jun 2015, *H. Beltrán & W. Aparco 7842* (USM); Yauyos, Laraos, Carhuanisho, 12°22'S, 75°38'W, 4 Jun 2017, *H. Beltrán 8086* (USM); Huarochirí, arriba de la laguna de Chumpicocha, 11°57'S, 76°9'W, 27 May 1953, *E. Cerrate 2006* (USM); Huarochirí, San Damián, Chanape, Soyracocha, 11°54'S, 76°1'W, 10 Jul 2013, *P. Gonzáles & B. Brito 2658* (USM); Huarochirí, San Damián, abra entre Chanape y la comunidad Checca, 11°55'S, 76°15'W, 14 Jul 2013, *P. Gonzáles & B. Brito 2665* (USM); **Moquegua**: General Sánchez Cerro, Yunga, Perusa, 16°11'S, 70°37'W, 3 Mar 2018, *D. Montesinos & J. Calvo 5956* (HSP); Mariscal Nieto, Calacoa, faldas Ticsani, SE, 16°44'S, 70°35'W, 4 Mar 2018, *D. Montesinos & J. Calvo 6006* (HSP); **Puno**: Ananea ad pagum Rinconada et circa lacum alpestrim Comuni, 14°37'S, 69°26'W, 21 Oct 1976, *L. Bernardi, A. Charpin & F. Jacquemoud 16772* (US); unpaved track across pampa to the N and W of the road between abra Pampilla and Ananea, 14°40'S, 69°41'W, 16 Mar 2014, *V.A. Funk, M. Diazgranados & E. Cochachin 13180* (USM); Carabaya, Corani, Minaspata, 14°1'S, 70°41'W, 15 Oct 2017, *P. Gonzáles 3837* (USM); San Antonio de Esquilache, 16°6'S, 70°17'W, 13 May 1937, *D. Stafford 730* (BM, K); Crestón, San Antonio [de Esquilache], 1 Jul 1937, *T.G. Tutin 1203* (BM).

### 10. Xenophyllum juniperinum

Taxon classificationAnimaliaAsteralesAsteraceae

(Hieron.) J.Calvo, Phytotaxa 326(3): 228. 2017.

A941181E-6972-5160-AFA2-D07A3E26241A

Werneria juniperina Hieron., Bot. Jahrb. Syst. 21(3): 365. 1895. Syntypes. Chile/Bolivia. Tacora-Sajama, 4200−4300 m, Oct 1876, *A. Stübel 106* (B, destroyed; photo F0BN015812!); Bolivia. Oruro: alrededores de Tomarapi, 4200−4400 m, Oct 1876, *A. Stübel 116* (B, destroyed; photo F0BN015812!). Neotype, designated by Calvo et al. (2017: 228): Chile. Arica-Parinacota: camino a Tacora antes del Portezuelo, 18°07’59’’S, 69°32’37’’W, 4505 m, 26 May 2011, *A. Moreira-Muñoz, M. Muñoz & V. Morales 1640* (SGO s.n.!; isoneotype: CONC-180071!).
Werneria lycopodioides S.F.Blake, J. Washington Acad. Sci. 18: 493. 1928. Xenophyllum lycopodioides (S.F.Blake) V.A.Funk, Novon 7(3): 240. 1997. Type. Peru. Tacna: Tacna, volcán Tacora, co. Quiñuta [cerro Queñuta], 5000 m, Apr 1926, *E. Werdermann 1164* (holotype: GH s.n.!; isotypes: B s.n.!, BM s.n.!, CAS s.n.!, CONC-28852!, E-00301054 (digital image!), F-694487!, G-00305675 (digital image!), K-000527738 (digital image!), LIL-26668!, LPB s.n.!, LP-002608 (digital image!), M-0147059 (digital image!), MO s.n.!, NY s.n.!, OS-0000378 (digital image!), UC s.n.!, US-00622867!, US-01256002!).

#### Description.

**Figure 16.**
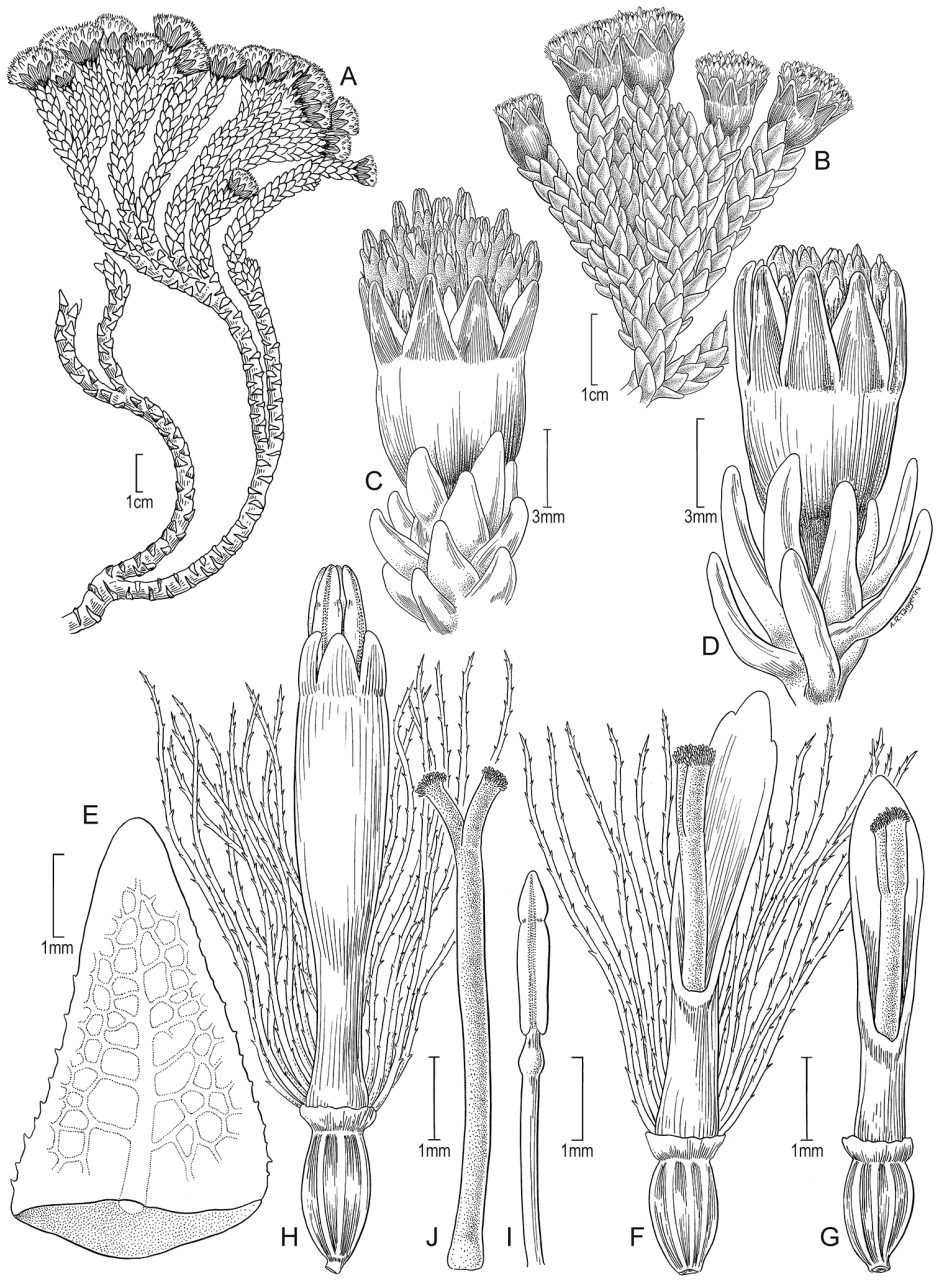
*Xenophyllum juniperinum***A** habit **B** stem apical part and capitula **C, D** uppermost leaves and capitulum (notice leaf variability) **E** adaxial leaf surface **F** ray floret (frontward bristles removed) **G** ray floret (pappus removed) **H** disc floret (frontward bristles removed) **I** stamen **J** style. **A, B** drawn from *Werdermann 1164* (GH), **C–E** (drawn from *Werdermann 1164*, CAS), and **F, G** (drawn from *Werdermann 1164*, US). Illustration by Alice Tangerini.

Suffruticose plant, forming clumps of erect stems. ***Rhizomes*** 5–10 × 0.4–0.6 cm, horizontal to oblique, glabrous. ***Stems*** 8–15 cm tall, usually simple, glabrous, with leaves uniformly arranged along it. ***Leaves*** imbricate, adpressed to the stem, extending into a glabrous sheath-like base; leaf laminas 5–7 × 1.1–2 mm, linear-triangular, rather acute at the apex, minutely, irregularly denticulate, obtusely triangular in cross section, glabrous, 1-nerved above (barely visible), 1-nerved beneath, fleshy, drying coriaceous, matte. ***Capitula*** radiate, erect, sessile. ***Involucres*** 7–7.7 × 4–6.2 mm, narrowly cupuliform; involucral bracts 8 to 11, 2.8–3.4 × 1.8–2.8 mm, acute at the apex, dark-purplish. ***Ray florets*** 8 to 13; corollas 4.5–5.5 × 0.4–0.7 mm, unconspicuously veined, subentire to slightly 3-toothed at the apex, not surpassing the involucre, yellow. ***Disc florets*** 28 to 36; corollas 5.5–5.7 mm long, yellow; style branches truncate with a crown of sweeping trichomes, yellowish. ***Achenes*** 3.4–3.9 × 0.8–1 mm, cylindrical, 7 to 8-ribbed, glabrous; pappus 5.2–9 mm long, barbellate, whitish. Chromosome number unknown. Figs 2C, 16.

#### Additional iconography.

Blake (1928: 496, fig. 1K–M sub *Werneria lycopodioides*); Beltrán (2016: 358, fig. 2A, sub *X. lycopodioides*, as photo); Calvo et al. (2017: 228, fig. 1, as photo).

#### Distribution and habitat.

**Figure 17.**
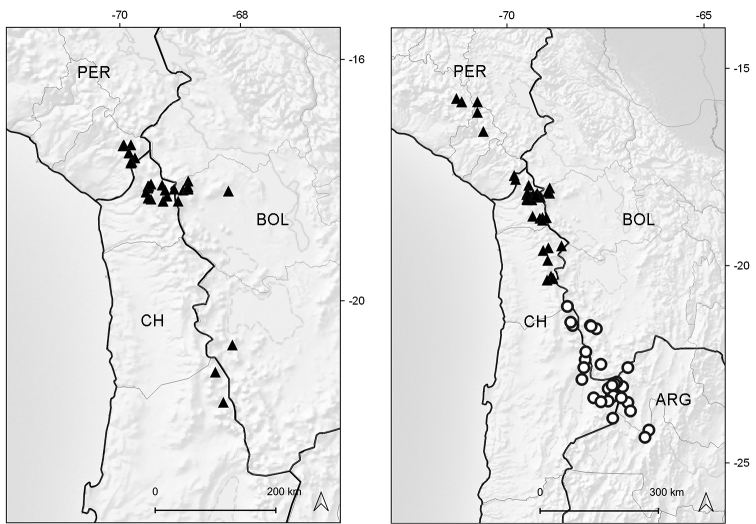
Distribution map of *Xenophyllum juniperinum* (left hand), *X. weddellii* (right hand, closed triangle), and *X. incisum* (right hand, open circle).

Southern Peru to northern Chile. Bolivia (Oruro, Potosí), Chile (Antofagasta, Arica-Parinacota, Tarapacá [expected]), Peru (Tacna). It grows in exposed places on rocky and sandy soils of the dry and desertic puna ecoregions, between elevations of 4000–5200 m (Fig. 17).

Most populations of this species are located between eastern Tacna (Peru), northeastern Arica-Parinacota (Chile), and northwestern Oruro (Bolivia). However, a few collections are from further south (northeastern Antofagasta in Chile and western Potosí in Bolivia). Its presence in the Chilean region of Tarapacá is expected.

#### Phenology.

Flowering from April to December.

#### Etymology.

It is named after the genus *Juniperus* L. (Cupressaceae) due to the leaves of this species resembling those of some species belonging to the mentioned genus.

#### Notes.

*Xenophyllum juniperinum* forms clumps of erect stems 8–15 cm long, which bear imbricate leaves that are adpressed to the stem and uniformly arranged along it. The leaves are somewhat broadened at the base and the margin is minutely, irregularly denticulate. The yellow ray corollas not surpassing the involucre is a distinctive character of this species, which is shared with *X. ciliolatum* (see comments under it for comparison purposes).

Although the syntypes of the name *Werneria juniperina* Hieron. were apparently destroyed at B in 1943, it should be mentioned that Rockhausen (1939) had the opportunity to study them and designated the collection *Stübel 106* as “typus”. See Calvo et al. (2017) for further details on the neotypification.

#### Additional specimens examined.

**Bolivia. Oruro**: Sajama, cerca 8 km SO hacia la quebrada de Tirallani (pasando quebrada Sururia), 18°9'S, 68°52'W, 25 Mar 2005, *S.G. Beck 31109* (LPB); Sajama, subiendo al cerro Sajama, desde la quebrada Tirallani, 18°9'S, 68°52'W, 27 Mar 2005, *S.G. Beck 31148* (LPB); Sajama, de Tambo Quemado 10 km hacia Chachacomani y subiendo sobre una mina de azufre, sobre el glaciar Acotango, 18°21'S, 69°17'W, 14 Mar 2012, *S.G. Beck 32750* (LPB, US); Sajama, de Tambo Quemado 10 km hacia Chachacomani y subiendo sobre una mina de azufre, sobre el glaciar Acotango, 18°21'S, 69°17'W, 14 Mar 2012, *S.G. Beck 32753* (LPB); Sajama, de Tambo Quemado 10 km hacia Chachacomani y subiendo sobre una mina de azufre, sobre el glaciar Acotango, 18°21'S, 69°17'W, 14 Mar 2012, *S.G. Beck 32757* (LPB); P.N. Sajama, 5 km antes de la entrada para el pueblo de Sajama, 18°10'S, 68°57'W, 15 Nov 1999, *G. Bourdy 2197* (LPB); Sajama, S side of nev. Sajama just below snowline on E side of río Sururia, N walk up from 4300 m where rd. circles nev., 18°8'S, 68°53'W, 19 Apr 1995, *V.A. Funk 11352* (LPB, US); Sajama, ladera Sajama, 18 Aug 1982, *E. Jordan 158* (LPB, US); Sajama, S del nevado Sajama, 18°7'S, 68°52'W, 17 Mar 1984, *M. Liberman 812* (LPB, US); Sajama, Turco, 18°11'S, 68°12'W, 1993, *J. Mallea 15* (LPB); Sajama, Turco, 18°11'S, 68°12'W, 1993, *J. Mallea 24* (LPB); Sajama, nevado Sajama, 18 Aug 1982, *X. Menhofer 1506* (LPB, US); Sajama, Berg nördl. volcán Payachatas, 18°9'S, 69°6'W, 17 Sep 2001, *B.J. Ruthsatz 10539* (LPB); Sajama, Chachacomani, Acotango, 18°21'S, 69°2'W, 23 Sep 2001, *B.J. Ruthsatz 10549* (LPB); **Potosí**: Daniel Campos, a 11 km al W de San Pedro Quemes, 20°44'S, 68°8'W, 21 Jun 1999, *C. Salles 229* (LPB). **Chile. Antofagasta**: El Loa, trayecto entre cerro Carasilla y salar Ascotán, ladera exposición N, 21°41'S, 68°17'W, 28 Mar 1985, *M. Arroyo, C. Villagrán & J. Armesto 85-394B* (CONC); Ollagüe, volcán Aucanquilcha, ladera E, cerca de la línea del andarivel abandonado, 21°11'S, 68°25'W, 23 Apr 2019, *J. Calvo & A. Moreira-Muñoz 7930* (CONC, SGO); **Arica-Parinacota**: Parinacota, cerro Guane Guane, 18°10'S, 69°15'W, 20 Apr 1980, *M. Arroyo, C. Villagrán & J. Moreno 2623A* (CONC); Parinacota, camino desde Pacollo hasta nevados de Putre, 18°4'S, 69°29'W, 15 Apr 1984, *M. Arroyo 84-857* (CONC); Parinacota, cerro Choquelimpie, 18°16'S, 69°13'W, 19 Apr 1984, *M. Arroyo 84-912* (CONC); Tacora, 14 km hacia Aguas Calientes, después 1 km más arriba, 17°42'S, 69°48'W, 11 Feb 2015, *S.G. Beck 34533* (LPB); Parinacota, camino entre Pacollo y Colpitas, 18°5'S, 69°18'W, 18 Jan 2000, *E. Belmonte 20005* (CONC, MA); Parinacota, quebrada Vilasamanani, 18°18'S, 69°32'W, 15 Dec 1988, *E. Belmonte 88619* (CONC); Parinacota, quebrada Ichusgualla, 18°12'S, 69°34'W, 16 Dec 1988, *E. Belmonte 88635* (CONC); above road to Tacora (rt. A23), slope of co. de Llancoma, 18°8'S, 69°32'W, 7 Mar 2014, *V.A. Funk, M. Diazgranados & J.M. Bonifacio 13103* (US); NW slope of Volcán Tarapacá along small trail to the snow line, 18°6'S, 69°31'W, 9 Mar 2014, *V.A. Funk, M. Diazgranados & J.M. Bonifacio 13136* (US); cerro Chapiquiña, sobre el portezuelo, antenas, 18°19'S, 69°29'W, 9 Jun 2012, *A. Moreira-Muñoz 1936* (SGO); bajando desde Portezuelo Taapacá hacia Putre, en el límite comunal, 18°6'S, 69°32'W, 20 Oct 2012, *A. Moreira-Muñoz et al. 2037* (SGO, US); pasado Azufrera Tacora, 17°42'S, 69°49'W, 17 Mar 2015, *A. Moreira-Muñoz & F. Luebert 2408* (SGO); Aguas Calientes, Tacora, 17°43'S, 69°49'W, 17 Sep 1955, *M. Ricardi 3377* (CONC); Aguas Calientes, volcán Tacora, 17°43'S, 69°49'W, 19 Sep 1955, *M. Ricardi 3405* (CONC); nevados de Putre, cerro Taapaca, 18°6'S, 69°32'W, 15 Apr 2011, *S. Teillier & A. Buben 6579* (CONC). **Peru. Tacna**: Tarata, Poma, carretera Tarata-Puno, 17°25'S, 69°56'W, 4 Dec 1997, *A. Cano 7946* (USM); Tarata, cordillera del Barroso, 17°33'S, 69°51'W, 29 Mar 1998, *A. Cano 8244* (USM); 32 km N of Tarata, ca. 50 km S of abra Livini (Puno), road from Tarata to Mazo-Cruz (Puno), rd. 36, 17°26'S, 69°57'W, 11 Mar 2014, *V.A. Funk, M. Diazgranados & E. Cochachin 13139* (US, USM); Tarata, laguna Casire [Casiri], 17°25'S, 69°49'W, 3 Apr 1998, *M.I. La Torre 2375* (USM); Pasan los Vientos, cordillera del Barroso, 7 Dec 1997, *J. Roque et al. 538* (US, USM); Tarata, cordillera del Barroso, carretera Alto Perú-Palca, 17°33'S, 69°51'W, 7 Dec 1997, *J. Roque 559* (USM [mixed with *X. poposa*]); Tarata, cordillera del Barroso, carretera Alto Perú-Palca, 17°33'S, 69°51'W, 7 Dec 1997, *J. Roque 560* (USM).

### 11. Xenophyllum weddellii

Taxon classificationPlantaeAsteralesAsteraceae

(Phil.) V.A.Funk, Novon 7(3): 240. 1997.

C51245A6-D0F0-5632-9E35-1B5A78201A4E

Werneria weddellii Phil., Anales Mus. Nac., Santiago de Chile 8: 40. 1891 [“Weddelli”]. Type. Chile. Tarapacá: laguna de Huasco, 1 Mar 1885, *F. Philippi s.n.* (lectotype: SGO-044581!, designated as “holotype” by Funk (1997a: 240); isolectotype: LP-002610 (digital image!)).
Werneria decumbens Hieron., Bot. Jahrb. Syst. 21(3): 364. 1895. Type. Chile/Bolivia. Tacora a Tomarapi, 4200−4400 m, Oct 1876, *A. Stübel 100c* (B, destroyed; photo F0BN015808!). Neotype, designated here: Bolivia. Oruro: Parque Nacional Samaja, laguna Huainyacota [Huaña Khota], 18°02'S, 68°55'W, 4350 m, 11 Aug 2007, *M. Velayos, C. Aedo & C. Monge 11263* (MA-758188!; isoneotype: LPB s.n.!), syn. nov.

#### Description.

Suffruticose plant, forming clumps of rather decumbent stems. ***Rhizomes*** 7–10 × 0.5–0.8 cm, horizontal to oblique, glabrous. ***Stems*** 3–8 cm tall, simple or branched, glabrous, with leaves rather uniformly arranged along it. ***Leaves*** imbricate, extending into a glabrous sheath-like base; leaf laminas 3.4–7(–11.8) × 1.3–1.6 mm, linear-subulate, acute at the apex, entire, obtusely triangular in cross section, glabrous, unconspicuously nerved above, 1-nerved beneath (barely visible), fleshy, matte. ***Capitula*** radiate, erect, sessile. ***Involucres*** 7–9 × 5–7 mm, cupuliform; involucral bracts 8 to 12, 3.2–5.9 × 1.4–2.3 mm, acute to obtuse at the apex, greenish, sometimes purple-edged. ***Ray florets*** 9 to 15; corollas 5.9–8 × 1–1.2 mm, 3 to 4-veined, subentire at the apex, conspicuously surpassing the involucre, white, rarely pale purplish beneath. ***Disc florets*** 24 to 32; corollas 4.5–5.2 mm long, whitish to creamy, sometimes with the tube purplish; style branches truncate with a crown of sweeping trichomes, purplish. ***Achenes*** 3.8–4 × 0.9–1 mm, cylindrical, 8 to 9-ribbed, glabrous; pappus 6.3–7.5 mm long, barbellate, whitish. Chromosome number unknown.

#### Iconography.

Beltrán (2016: 358, fig. 2F, as photo).

#### Distribution and habitat.

Southern Peru to northern Chile. Bolivia (Oruro), Chile (Arica-Parinacota, Tarapacá), Peru (Arequipa, Moquegua, Puno, Tacna [n.v.]). It grows along the banks of marshes and salt lagoons of the subhumid and dry puna ecoregions, between elevations of 3800–4700 m (Fig. 17).

#### Phenology.

Flowering nearly all year round.

#### Etymology.

This species honors H.A. Weddell (1819–1877), a British botanist (French by choice) and physician who devoted part of his life to the study of the South American flora.

#### Notes.

*Xenophyllum weddellii* is a species forming clumps of rather decumbent stems with linear, entire, glabrous leaves. It has 8 to 12 involucral bracts and 9 to 15 ray florets with white corollas that conspicuously surpass the involucre. On living plants, the color of the leaves is typically glaucous green.

The leaf apex is acute but we studied a specimen showing two lateral divisions near the apex (*Moreira-Muñoz & Diazgranados 2625*, SGO). It is identified as *X. weddellii* because the remaining characters perfectly fit with this species. Two emerging protrusions near the apex were also observed in a few other collections, which indicates that Moreira-Muñoz’s collection can be considered as an extreme of variability.

This species is morphologically similar to *X. ciliolatum* (see comments under it) and *X. incisum*. From this latter species, it mainly differs in the leaf lamina shape (linear-subulate, tapering upwards in *X. weddellii* vs. linear-oblong, similar width from base to apex in *X. incisum*) and leaf apex (acute in *X. weddellii* vs. 3-notched with the lobes rather truncate at the apex in *X. incisum*). It is interesting to mention that both species have similar ecological requirements and they usually thrive near the banks of the salt lagoons and moist valley bottoms. They could be referred to as allopatric species since their distribution areas are well defined and do not overlap.

The single original collection that we found comes from the Huasco Lagoon (Tarapacá, Chile). However, Philippi (1891) indicated additional sites in the protologue, i.e., “Machuca” and “regione inter Copacoya et Inacaliri”. These localities are located further south in the region of Antofagasta, where this species appears to be absent. In contrast, *X. incisum* is not rare in this region and, indeed, the provenance of the type material is “Inter Copacoya et Inacaliri”. The lack of original material of *X. weddellii* from these localities and the fact that we did not study any collection from there suggests that Philippi might had confused both species.

The holotype designation of the name *Werneria weddellii* Phil. has been corrected to lectotype according to McNeill (2014). In addition, the name *Werneria decumbens* Hieron. is synonymized with *X. weddellii* as suggested by Funk (1997a). Since the original material was likely destroyed at B in 1943, a neotype is designated. The provenance of the selected collection perfectly matches the protologue information.

#### Additional specimens examined.

**Bolivia. Oruro**: Sajama, al borde del río Sajama, en el pueblo de Sajama, 18°8'S, 68°58'W, 24 Aug 2008, *S.G. Beck 32617* (LPB); Sajama, 18°10'S, 68°55'W, 9 Feb 1998, *F. Loza de la Cruz 264* (LPB, USM). **Chile. Arica-Parinacota**: N de Misitune, 18°15'S, 69°22'W, 25 Nov 2001, *C. Aedo 6968* (CONC, MA); Parinacota, salar de Surire, 18°51'S, 69°6'W, 29 Mar 1992, *G. Arancio 92-454* (CONC); salar de Surire, 18°47'S, 69°6'W, 27 Mar 1996, *M. Argomedo 9* (CONC); Parinacota, cerca de laguna de Cotacotani, camino a Guane Guane, 18°10'S, 69°14'W, 9 Mar 1984, *M. Arroyo 84-728* (CONC); Parinacota, salar de Surire, aledaños del salar, 18°48'S, 69°10'W, 6 Aug 1986, *E. Belmonte 86119* (CONC); Parinacota, lago Chungará, 18°14'S, 69°10'W, 9 May 1996, *E. Belmonte 96068* (CONC); Las Cuevas, 18°12'S, 69°28'W, 23 Mar 1997, *E. Belmonte 97049* (CONC); Arica, altiplano de Arica, Las Cuevas, 18°12'S, 69°28'W, 15 Nov 1977, *H. Escobar 206* (CONC); rt. 11, on the edge of lago Chungará, large island, 18°16'S, 69°9'W, 6 Mar 2014, *V.A. Funk, M. Diazgranados & J.M. Bonifacino 13087* (US); rd. from rt. 11 to Parque Nacional Lauca (rt. A201), ca. 15 km W of intersection of rt. A201, A211 & A235, 18°19'S, 69°29'W, 8 Mar 2014, *V.A. Funk, M. Diazgranados & J.M. Bonifacino 13127* (US); rd. from rt. 11 to Parque Nacional Lauca (rt. A201), ca. 5 km W of intersection of rt. A201, A211 & A235, 18°19'S, 69°30'W, 8 Mar 2014, *V.A. Funk, M. Diazgranados & J.M. Bonifacino 13133* (US); Putre, Ch11-road to Putre at kilometer 150, 18°9'S, 69°29'W, 15 Dec 1999, *M.F. Gardner & S.G. Knees 6266* (E); Aguas Calientes, 17°43'S, 69°49'W, Nov 1955, *U. Levi 11* (CONC); camino de Putre a Chucuyo, km 10, 18°13'S, 69°16'W, 12 Feb 1964, *C. Marticorena, O. Matthei & M. Quezada 198* (CONC); termas de Jurasi, 18°12'S, 69°30'W, 26 May 2011, *A. Moreira-Muñoz, M. Muñoz & V. Morales 1658* (SGO); base del cerro Chapiquiña, bofedal Ojo de Agua, 18°19'S, 69°29'W, 9 Jun 2012, *A. Moreira-Muñoz 1941* (SGO); lado este Portezuelo Chapiquiña, frente Ojo de Agua, 18°19'S, 69°29'W, 19 Oct 2012, *A. Moreira-Muñoz et al. 2016* (SGO); quebrada profunda antes de Colpitas, después de cruce del río, 17°58'S, 69°27'W, 20 Oct 2012, *A. Moreira-Muñoz et al. 2022* (SGO); quebrada Ancochallane, 18°15'S, 69°24'W, 18 Jun 2015, *A. Moreira-Muñoz 2495A* (SGO); camino Umirpa a Tignamar, ca. km 28, 18°45'S, 69°21'W, 26 May 2016, *A. Moreira-Muñoz & M. Diazgranados 2625* (SGO); Parinacota, entre Cotacotani y Chungará, 18°15'S, 69°13'W, 20 Apr 1980, *J. Moreno 2668* (CONC); Parinacota, entre central de Chapiquiña y Portezuelo de Chapiquiña, 18°20'S, 69°29'W, 17 May 1989, *H. Niemeyer, C. Fernández & A. Hoffmann 8992* (CONC); Aguas Calientes, Tacora, 17°43'S, 69°49'W, 17 Sep 1955, *M. Ricardi 3379* (CONC); Arica, Misitune, lecho del río Lauca, 18°20'S, 69°22'W, 7 Sep 1963, *F. Schlegel 4751* (CONC); Tacora-Humapalca-río Azufre, 17°49'S, 69°47'W, 4 Jan 2013, *S. Teillier 7740* (CONC); Arica, Chilcaya, 18°47'S, 69°0'W, 20 Apr 1927, *C. Troll 3320* (B, CONC); Parinacota, portezuelo de Putre, 18°12'S, 69°20'W, 18 May 1979, *C. Villagrán et al. 1175* (CONC); **Tarapacá**: Iquique, Huara, road from Lirima to Pachica, along the río Chancacolla (after La Rinconada), 19°52'S, 68°58'W, 11 Dec 2008, *R. Baines et al. 363* (E); La Cruz, 20°22'S, 68°59'W, 3 Dec 1948, *V. Castillo s.n.* (CONC); South edge of salar Huasco, NW of intersection of rt. A-687 & A-683, 20°20'S, 68°51'W, 4 Mar 2014, *V.A. Funk, M. Diazgranados & J.M. Bonifacino 13082* (US); Iquique, Huara, km 108 on A 483 to Colchane, 19°33'S, 68°57'W, 18 Feb 2003, *M.F. Gardner & S.G. Knees 6525* (E); comuna de Pica, salar de Huasco, bofedal de Huasco-Lípez, 20°19'S, 68°50'W, 23 Mar 2003, *S. Teillier & G. Mieres 5447* (CONC); Iquique, camino de Chusmiza a quebrada de Aroma, 19°37'S, 69°5'W, 20 Mar 1982, *C. Villagrán & M. Arroyo 4093* (CONC); Iquique, trayecto entre Cariquima y Guaitani, 19°30'S, 68°37'W, 6 Sep 1997, *C. Villagrán, F. Hinojosa & C. Latorre 9167* (CONC). **Peru. Arequipa**: [without locality, pr. Vincocaya according to coordinates], 15°51'S, 71°9'W, 21 Dec 2006, *F. Cáceres, E. López & L. Castillo 6025* (HUSA); río Sumbay, 15°51'S, 71°9'W, 22 Dec 2006, *F. Cáceres, E. López & L. Castillo 6063* (HUSA); río Sumbay, 15°51'S, 71°9'W, 23 Dec 2006, *F. Cáceres, E. López & L. Castillo 6149* (HUSA); río Sumbay, 15°51'S, 71°9'W, 23 Dec 2006, *F. Cáceres, E. López & L. Castillo 6178* (HUSA); Caylloma, Yanque, entre Imata y Chalhuanca, 15°46'S, 71°17'W, 17 May 2017, *V. Quipuscoa et al. 5570* (HSP); **Moquegua**: Ubinas, Querala, Gasahuasi, 16°7'S, 70°45'W, 6 Apr 2011, *D. Montesinos 3090* (HSP, USM); General Sánchez Cerro, Chojata, quebrada Huarata, 16°36'S, 70°36'W, 4 Mar 2018, *D. Montesinos & J. Calvo 5984* (HSP); **Puno**: San Román, hacienda Tincopalca, 15°51'S, 70°45'W, 11 Mar 1953, *E. Petersen & J.P. Hjerting 1090* (C, LIL, USM).

### 12. Xenophyllum incisum

Taxon classificationPlantaeAsteralesAsteraceae

(Phil.) V.A.Funk, Novon 7(3): 239. 1997.

96925374-0387-5357-A453-E8FAC13D939D

Werneria incisa Phil., Anales Mus. Nac., Santiago de Chile 8: 41. 1891. Type. Chile. Antofagasta: inter Copacoya et Inacaliri, 19 Feb 1885, *F. Philippi s.n.* (lectotype: Philippi’s collection at SGO as the first-step lectotype, designated as “holotype” by Funk (1997a: 239); SGO-060388! as the second-step lectotype, designated here; isolectotypes: LP-002607 (digital image!), SGO-000006431 (ruined!)).

#### Description.

Suffruticose plant, forming clumps of rather decumbent stems. ***Rhizomes*** 4–8 × 0.4–0.6 cm, horizontal to oblique, glabrous. ***Stems*** 4–8 cm tall, simple or branched, glabrous, with leaves rather uniformly arranged along it. ***Leaves*** imbricate, extending into a glabrous sheath-like base; leaf laminas 3.2–7.3 × 1.4–1.9 mm, linear-oblong, 3-notched at the apex, sometimes with the central lobe wider than lateral ones (rarely subentire), entire, obtusely triangular to slightly curved forwards in cross section, glabrous, unconspicuously nerved above, 1-nerved beneath, fleshy, matte; leaf lobes 0.5–1 × 0.5–0.6 mm, obtuse to truncate. ***Capitula*** radiate, erect, sessile. ***Involucres*** 6.4–8.3 × 4.6–6.1 mm, cupuliform; involucral bracts 8 to 9, 3.1–4.3 × 1.1–2.6 mm, obtuse at the apex, greenish. ***Ray florets*** 8 to 13; corollas 6.2–8.9 × 0.6–1.2 mm, 2 to 4-veined, subentire to 3-toothed at the apex, conspicuously surpassing the involucre, white. ***Disc florets*** 26 to 37; corollas 4.4–5 mm long, pale yellow to creamy; style branches truncate with a crown of sweeping trichomes, purplish. ***Achenes*** 2.3–2.4 × 0.8–0.9 mm, cylindrical, ca. 8-ribbed, glabrous; pappus 4.8–8 mm long, barbellate, whitish. Chromosome number unknown. Fig. 19C.

#### Additional iconography.

Cabrera (1978: 473, fig. 200G, H, sub *Werneria incisa*).

#### Distribution and habitat.

Restricted to northern Chile, northwestern Argentina, and southwestern Bolivia. Argentina (Jujuy, Salta), Bolivia (Potosí), Chile (Antofagasta). This species grows along the banks of salt lagoons and desert plains with a certain humidity of the dry and desertic puna ecoregions, between elevations of (2600–)3500–4900 m (Fig. 17).

#### Phenology.

Flowering from October to March.

#### Etymology.

The epithet *incisum* means sharply and deeply cut into, which describes the leaf apex of this species. However, its leaf apex is rather notched (shallowly cut into).

#### Notes.

This species can be properly identified by the combination of the following characters: rather decumbent, 4–8 cm tall, glabrous stems, linear-oblong leaf lamina, 3-notched leaf apex with truncate lobes (rarely subentire), 8 to 9 involucral bracts, 8 to 13 ray florets with white corollas conspicuously surpassing the involucre, and glabrous achenes.

*Xenophyllum incisum* is somewhat variable in leaf apex shape. Typical forms have 3-notched leaf apex, usually with the central lobe wider than lateral ones, but in some specimens the apex is barely notched or even subentire (e.g., *Moreira-Muñoz 298*, CONC; *Arroyo et al. 97315*, CONC). In these specimens, however, the leaf apex is truncate and slightly curved forwards as typically in the species. Similarly, we studied one specimen having the leaf apex with each lobe 3-notched (*Villagrán et al. 9321*, CONC).

This species has morphological affinities with *X. lorochaqui* J.Calvo & V.A.Funk, *X. weddellii* (see comments under these species), and *X. poposa*. With regard to the latter species, the main and unequivocal character that allows a proper identification is the stem indumentum (glabrous in *X. incisum* vs. arachnoid in *X. poposa*). The characters concerning the leaf apex have traditionally been used for discriminating among these species, however, they are barely useful because *X. poposa* also shows high variability in leaf apex (see further comments under it). A rather useful character is the leaf section, which is obtusely triangular to slightly curved forwards in *X. incisum* vs. terete or almost so in *X. poposa*. The sheath-like base is also more broadened in *X. incisum*.

The holotype designation by Funk (1997a) is corrected to lectotype because the gathering indicated by Philippi in the protologue is not represented by a single specimen. Moreover, a second-step lectotypification is made because two specimens of this gathering were found at SGO.

#### Additional specimens examined.

**Argentina. Jujuy**: Susques, a 30 km de Jama, entre Jama y Susques, 23°28'S, 66°56'W, 22 Jan 2000, *A. Bardón s.n.* (LIL); Achibarca, 23°41'S, 66°52'W, 11 Mar 1927, *A. Castellanos s.n.* (BA); bolsón del Vilama, 22°35'S, 66°56'W, 28 Sep 1939, *J.S. Collan 44* (LIL); **Salta**: Tusle [Tuzgle], pr. mina Concordia, 24°10'S, 66°24'W, 29 Oct 1901, *R.E. Fries 707* (US); abra del Gallo, ca. 40 km al SW de S. Antonio de los Cobres, en el camino a Pastos Grandes, 24°21'S, 66°30'W, 17 Dec 1946, *A. Krapovickas 3209* (K, LIL). **Bolivia. Potosí**: Sud Lípez, laguna Verde 36 km hacia laguna Colorada, 22°30'S, 67°37'W, 28 Apr 2000, *S.G. Beck 27510* (LPB); Nor Lípez, ruta laguna Hedionda-Alota, 23 km de laguna Hedionda, 21°32'S, 67°52'W, 23 Sep 2006, *S.G. Beck 32410* (LPB, USM); Sud Lípez, laguna Pastos Grandes, al E de la laguna, 21°36'S, 67°44'W, 20 May 1989, *E. García 1101* (LPB, USM); Sud Lípez, cerro Tapaquillcha, 21°31'S, 67°53'W, 13 Apr 1980, *M. Liberman 206* (LPB). **Chile. Antofagasta**: El Loa, salar de Quisquiro, 23°17'S, 67°21'W, Jan 1997, *G. Arancio 10676* (CONC); El Loa, sector N del salar de Aguas Calientes, 23°4'S, 67°22'W, Jan 1997, *G. Arancio 10697* (CONC); El Loa, salar de Aguas Calientes, 23°27'S, 67°37'W, 17 Mar 1992, *G. Arancio 92-341* (CONC); El Loa, camino de San Pedro de Atacama a paso Jama, quebrada Llano Vilama, 22°53'S, 68°6'W, 6 Apr 1997, *M. Arroyo, L. Cavieres & A. Humaña 97217* (CONC); El Loa, ojos del río Salado, pequeño salar en el extremo N, 23°21'S, 67°22'W, 8 Apr 1997, *M. Arroyo, L. Cavieres & A. Humaña 97276* (CONC, MA); El Loa, salar Quisquiro, lado E, 23°18'S, 67°18'W, 8 Apr 1997, *M. Arroyo, L. Cavieres & A. Humaña 97315* (CONC); El Loa, cerro Nevados de Poquis, ladera SO, 23°4'S, 67°4'W, 9 Apr 1997, *M. Arroyo, L. Cavieres & A. Humaña 97373* (CONC); El Loa, salar de Aguas Calientes, 23°7'S, 67°26'W, 11 Apr 1997, *M. Arroyo, L. Cavieres & A. Humaña 97463* (CONC); El Loa, laguna El Chivato Muerto, 23°26'S, 67°26'W, 14 Feb 1994, *G. Baumann 352* (CONC); salar de Ascotán, 21°31'S, 68°20'W, 19 Jan 1957, *F. Behn 19596* (CONC); Antofagasta, Loa, Toconao, N del salar de Quisquiro, quebrada de Taina, 23°11'S, 67°19'W, 4 Mar 2019, *J. Calvo 7914* (CONC, SGO); Antofagasta, Loa, San Pedro de Atacama, Machuca-El Tatio, ca. 6.2 km al S de El Tatio, 22°23'S, 68°1'W, 5 Mar 2019, *J. Calvo 7924* (SGO); salar de Ascotán, along the margins of the salar, 21°2'S, 68°28'W, 4 Mar 2014, *V.A. Funk, M. Diazgranados & J.M. Bonifacio 13077* (US); salar de Ascotán, along the margins of the salar, 21°2'S, 68°28'W, 4 Mar 2014, *V.A. Funk, M. Diazgranados & J.M. Bonifacio 13078* (US); salar de Tara, 23°2'S, 67°20'W, 7 Dec 1988, *A. Hoffmann s.n.* (CONC); Cebollar, entre el salar de Ascotán y el salar de San Martín, 21°26'S, 68°23'W, 20 Feb 1964, *C. Marticorena, O. Matthei & M. Quezada 405* (CONC); vegas del río Zapaleri, 22°57'S, 67°13'W, 18 Dec 1996, *A. Moreira-Muñoz 257* (CONC); borde W salar de Tara, 23°0'S, 67°16'W, 18 Dec 1996, *A. Moreira-Muñoz 289* (CONC); borde sur salar de Tara, 22°59'S, 67°18'W, 18 Dec 1996, *A. Moreira-Muñoz 298* (CONC); Mucar [laguna], 23°21'S, 67°6'W, 14 Dec 1965, *L. Peña 5* (CONC); Machuca, 22°35'S, 68°3'W, 18 Feb 1885, *F. Philippi s.n.* (GB); Tarapacá [without locality and date, probably a duplicate of Philippi 18 Feb 1885], *F. Philippi s.n.* (US); El Loa, San Pedro de Atacama, Socaire, laguna Sico, 23°52'S, 67°19'W, 5 Apr 1997, *S. Teillier 4058* (CONC); El Loa, salar de Ascotán, 21°31'S, 68°20'W, Feb 1997, *S. Teillier 4226* (CONC); El Loa, trayecto entre Talabre y laguna Lejía, 23°21'S, 67°48'W, 2 Apr 1998, *C. Villagrán, F. Hinojosa & C. Latorre 9321* (CONC).

### 13. Xenophyllum poposa

Taxon classificationPlantaeAsteralesAsteraceae

(Phil.) V.A.Funk, Novon 7(3): 240. 1997 [“poposum”].

67068DE1-36E7-5A8B-8782-97F7AD27F1D4

Werneria poposa Phil., Anales Mus. Nac., Santiago de Chile 8: 40. 1891. Type. Chile. Antofagasta: Copacoya, 18 Feb 1885, *F. Philippi s.n.* (lectotype: Philippi’s collection at SGO as the first-step lectotype, designated as “holotype” by Funk (1997a: 240); SGO-000006433! as the second-step lectotype, designated here; isolectotypes: LP-002609 (digital image!), SGO-000006434!).
Werneria lorentziana Hieron., Bot. Jahrb. Syst. 21(3): 364. 1895 [“Lorentziana”]. Syntypes. Chile/Peru. [“prope Tacora, 4200 m, Oct 1876, *A. Stübel 107*” according to the *ind. loc.*] (B, destroyed); Bolivia. Oruro: [“prope Tomarapi, 4200−4400 m, Oct 1876, *A. Stübel 117*” according to the *ind. loc.*] (B, destroyed). Neotype, designated as “lectotype” by Freire and Ariza-Espinar (2014: 227): Argentina. Salta: alrededores del Nevado del Castillo, 19/23 Mar 1873, *P.G. Lorentz & G. Hieronymus 117* (CORD-00005637 (digital image!); isoneotype: GOET s.n.!).
Werneria humilis Griseb. ex Hieron., Bot. Jahrb. Syst. 21(3): 364. 1895, *nom. inval. pro syn.* (Turland et al. 2018, ICN Art. 36.1).
Werneria incisa var. pubescens Rockh., Bot. Jahrb. Syst. 70: 290. 1939. Xenophyllum incisum var. pubescens (Rockh.) Cabrera & S.E.Freire, Monogr. Syst. Bot. Missouri Bot. Gard. 74: 1245. 1999. Type. Argentina. La Rioja: Sierra Famatina, laguna Moradita, 13 Mar 1907, *F. Kurtz 14632* (lectotype: CORD-00005636 (digital image!), designated as “isotype” by Cabrera and Freire (1999: 1245)), syn. nov.

#### Description.

**Figure 18.**
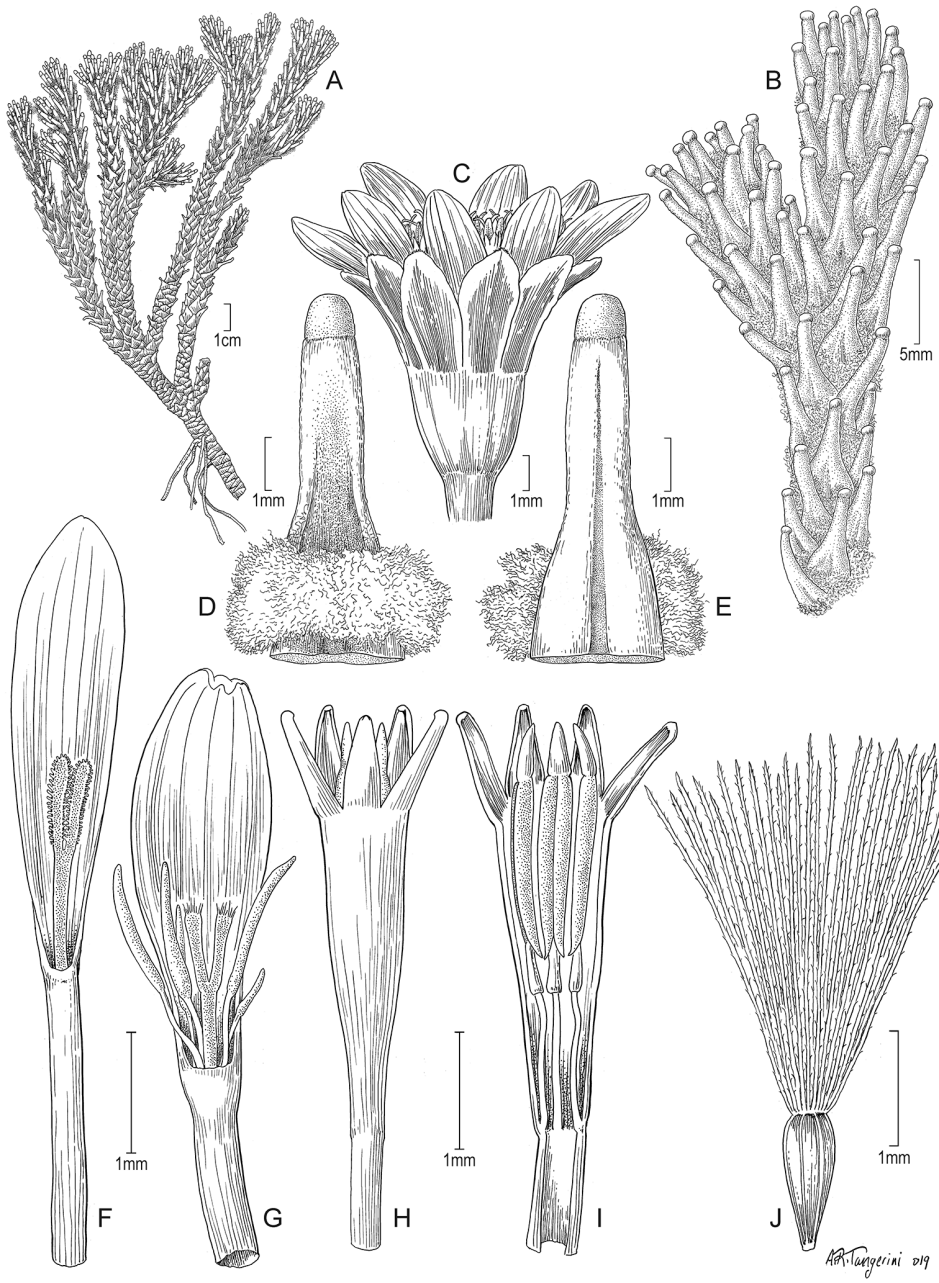
*Xenophyllum poposa***A** habit **B** stem apical part **C** capitulum **D** adaxial leaf surface **E** abaxial leaf surface **F** ray corolla and style **G** ray corolla and style (notice staminodes) **H** disc corolla **I** disc corolla and stamens (vertically sectioned, style removed) **J** achene with pappus. All details drawn from *Funk et al. 13138* (US) except for A, B, D (drawn from *Venturi 2964*, US). Illustration by Alice Tangerini.

**Figure 19.**
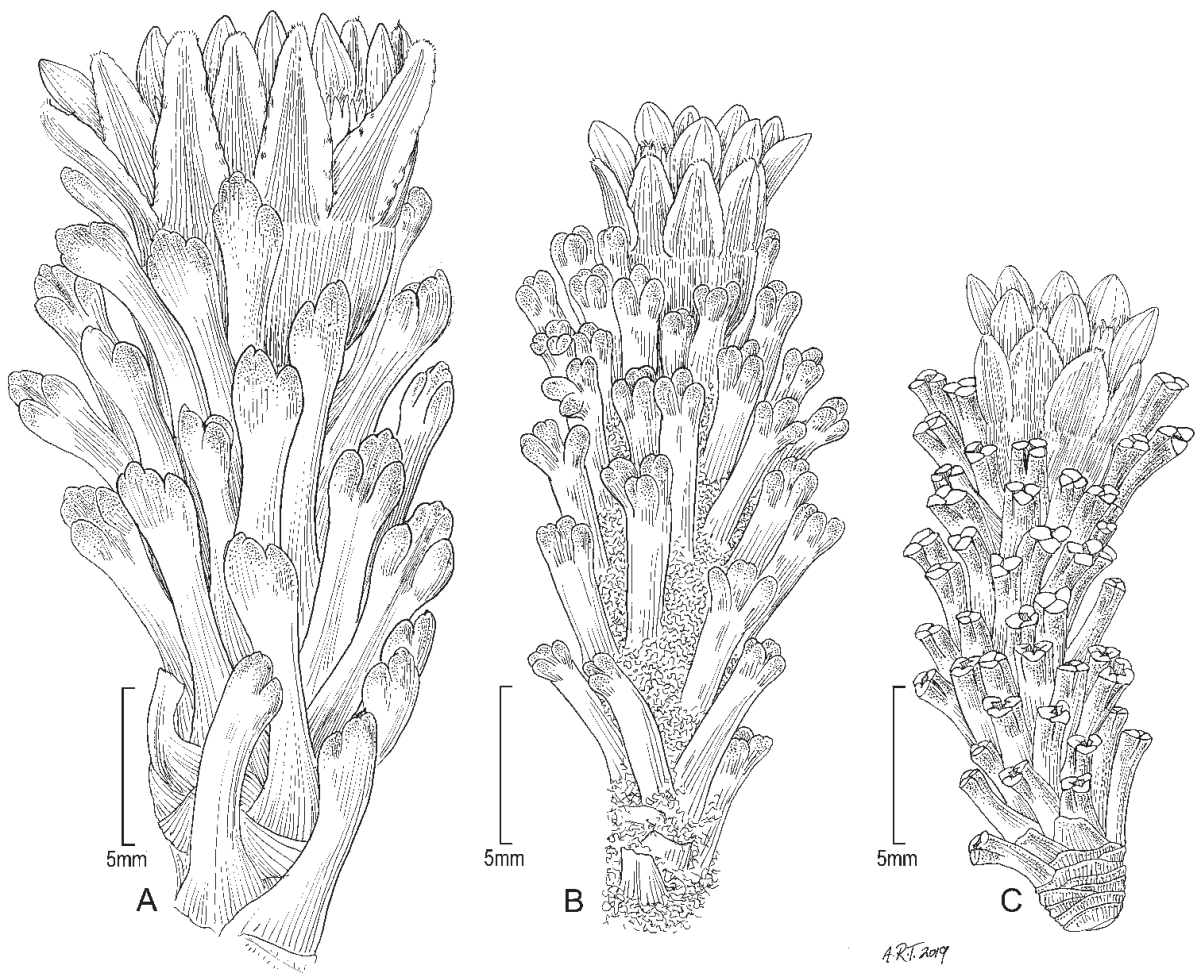
Detail of leaves and capitulum **A***Xenophyllum lorochaqui*, drawn from *Castellanos s.n.* (BA) **B***X. poposa*, drawn from *Cárdenas 362* (US) **C***X. incisum*, drawn from *Funk et al. 13078* (US). Illustration by Alice Tangerini.

Suffruticose plant, forming clumps of erect stems (rarely dense mats). ***Rhizomes*** 4–10 × 0.3–0.7 cm, horizontal to oblique, glabrescent. ***Stems*** 2–15 cm tall, simple or branched, arachnoid, usually with functional green leaves restricted to the upper part. ***Leaves*** imbricate, extending into a sheath-like base that bears arachnoid trichomes; leaf laminas 2.5–7.1 × 0.5–1.1 mm, linear, rounded to truncate or 2 to 3-notched at the apex (usually white callous-tipped), entire, rather terete in cross section, glabrous, unconspicuously nerved on both faces, fleshy, matte; leaf lobes (when present) 0.6–1.5 × 0.3–0.4 mm, rounded to truncate, usually white callous-tipped. ***Capitula*** radiate, erect, sessile. ***Involucres*** (3.3–)5.6–8 × (2.7–)3–5.3 mm, narrowly cupuliform; involucral bracts 8 to 9, (1.1–)1.6–3.9 × 0.7–2.1 mm, acute to obtuse at the apex, greenish to partially dark-purplish. ***Ray florets*** 8 to 11; corollas 3.6–8.9 × 0.5–1.2 mm, 4-veined (sometimes unconspicuous), subentire to 3-toothed at the apex, conspicuously surpassing the involucre, white. ***Disc florets*** 7 to 18; corollas 3.2–5.9 mm long, whitish to creamy (rarely purplish); style branches truncate with a crown of sweeping trichomes, purplish. ***Achenes*** 2.8–2.9 × 0.7–0.8 mm, cylindrical, ca. 7-ribbed, glabrous; pappus (2.3–)4.1–5 mm long, barbellate, whitish to partially rose-colored. Figs 18, 19B.

#### Additional iconography.

Cabrera (1978: 473, fig. 200I, J, sub *Werneria poposa*); Freire and Ariza-Espinar (2014: 227, sub X. incisum var. pubescens A–H, 228, *X. poposa* A–G); Beltrán (2016: 358, fig. 2D, as photo, 359, fig. 3E, sub *X. incisum*, as photo).

#### Distribution and habitat.

**Figure 20.**
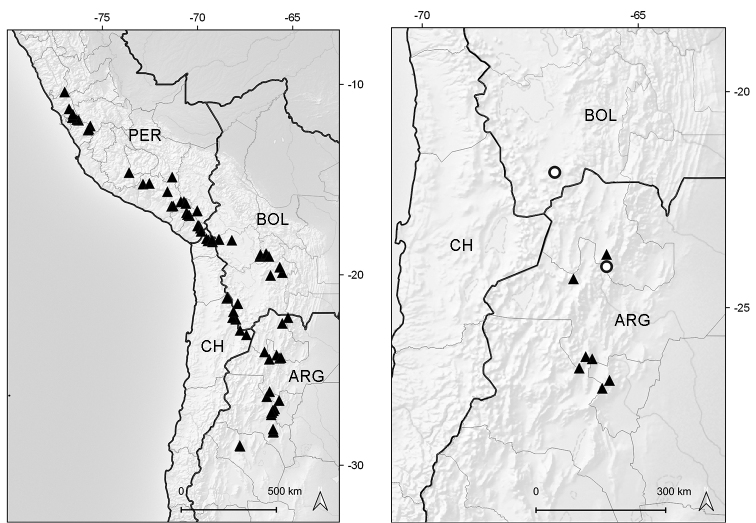
Distribution map of *Xenophyllum poposa* (left hand), *X. lorochaqui* (right hand, closed triangle), and *X. rosenii* (right hand, open circle).

Central Peru to northwestern Argentina. Argentina (Catamarca, Jujuy, La Rioja, Salta, Tucumán), Bolivia (Oruro, Potosí), Chile (Antofagasta, Arica-Parinacota, Tarapacá [expected]), Peru (Arequipa, Ayacucho, Cusco, Junín, Lima, Moquegua, Puno [expected], Tacna). It grows in rocky outcrops, grasslands, exposed slopes, and flat sandy pampas of the subhumid, dry, and desertic puna ecoregions, between elevations of (2600–)3000–5200 m (Fig. 20).

#### Phenology.

Flowering nearly all year round.

#### Etymology.

The epithet *poposa* is the vernacular name of this plant as Philippi (1891) stated in the protologue “ubi *Poposa* vocabatur” [called poposa]. He also added “infusum plantae contra dolores colicos propinant” [for relieving stomach cramps and colic] referring to its medicinal properties. It is important to underline that poposa is a noun in apposition not to be declined. The correct epithet is, therefore, *poposa* instead of the widely misapplied *poposum* (Funk 1997a; Freire and Ariza-Espinar 2014; Beltrán 2016).

#### Notes.

*Xenophyllum poposa* forms clumps of erect stems (rarely dense mats) characterized by having arachnoid indumentum. When the stems are very short and barely protrude from the soil, then, it tends to develop a mat-forming habit. The leaves are rather terete with the sheath-like base weakly broadened at the base. The leaf apex is usually undivided, rounded to truncate, and callous-tipped, however, plants having 2 to 3-notched apex are also found. It has 8 to 9 involucral bracts, 8 to 11 ray florets (with white corollas surpassing the involucre), 7 to 18 disc florets, and glabrous achenes. It is noteworthy that we observed a few ray florets with staminodes (Fig. 18G).

The type material of this species shows plants with clearly truncate and callous-tipped leaf apex, which probably led Rockhausen (1939) to relate a collection displaying notched leaf apex to *X. incisum* and describe it as Werneria incisa var. pubescens Rockh. He differentiated the new variety from the typical one by the longer leaves and the pubescent-lanate sheath-like bases. A detailed study of the lectotype (CORD-00005636) reveals that the stem bears arachnoid indumentum and that the sheath-like bases are not conspicuously broadened. These characters are distinctive of *X. poposa* rather than *X. incisum*. Moreover, this latter species is restricted to the edges of salt lagoon edges and desert plains with a certain humidity. The type material of Rockhausen’s variety was collected in the upper part of the Famatina Range (La Rioja, Argentina), which better matches the habitat of *X. poposa* (exposed slopes). On this basis, and because we believe that the glabrousness is a diagnostic character of *X. incisum*, we do not follow Rochkausen’s treatment as previous botanists did (Cabrera 1948; Freire and Ariza-Espinar 2014). Otherwise, we consider it appropriate to recognize W. incisa var. pubescens as part of the variability encompassed by *X. poposa*. Our decision is also supported by the fact that we studied further specimens corresponding to *X. poposa* that display notched leaf apex. These populations appear very scattered from southern Peru to northwestern Argentina, namely in Moquegua, Oruro, Potosí, Tucumán, Catamarca, and La Rioja. In Moquegua some specimens are strikingly tiny, barely protruding from the soil, and exhibit 2 to 3-notched leaf apex (*Beltrán 7736*, USM; *Funk et al. 13155*, US, USM). Due to field work in this region we can state that these forms grow mixed with plants displaying undivided, rounded to truncate leaf apex. It also deserves to be underlined that the different leaf morphologies can even be found within the same individual. The specimen *Funk et al. 13155* (US), for example, has undivided, 2-notched, and 3-notched leaves. In Potosí (Bolivia), we visited another population composed of midsize plants showing 3-notched leaves where each lobe can be, in turn, 2-notched (*Calvo & Zárate 7869*, BOLV, LPB; *Cárdenas 362*, US [see Fig. 19B]). No individuals with undivided leaves were found there. On the other hand, the specimens from Famatina (La Rioja, Argentina) are quite robust plants having stems 11–13 cm long and 2 to 3-notched leaves. Based on the study of herbarium specimens, we also can conclude that the populations having notched leaf apex from Oruro, Tucumán, and Catamarca grow mixed with or close to the typical plants. All these examples demonstrate that a great intrapopulation and interpopulation variability exists in terms of both leaf apex and habit. Our efforts for trying to propose an infraspecific classification in pursuit of recognizing the distinct morphologies failed because of the intermediate forms and the lack of defined distribution patterns. Therefore, we believe that *X. poposa* should be treated as a highly variable species.

The distribution area of this species partially overlaps with that of *X. incisum* in the region comprised between Antofagasta (Chile), Potosí (Bolivia), Salta, and Jujuy (Argentina). However, they occur in different ecological niches as already mentioned above. The morphological differences between them are commented under *X. incisum*. Likewise, *X. poposa* might be confused with *X. lorochaqui* (see notes under it).

Regarding nomenclatural issues, it is noteworthy that Funk’s typification of the name *Werneria poposa* Phil. has been narrowed to a single specimen because two duplicates of the same gathering were found at SGO (Turland et al. 2018, ICN Art. 9.17). The specimen SGO-000006433 has therefore been selected as the second-step lectotype because it is most complete and best preserved.

The original material of *Werneria lorentziana* Hieron. comprises the syntypes *Stübel 107* and *Stübel 117*, both apparently destroyed. Rockhausen (1939) designated as “typus” the collection *Stübel 117* but indicated the locality that in the protologue corresponds to the number *Stübel 107*. Since these specimens are not extant anymore and we did not find pictures of them at F, such mismatch cannot be elucidated. Later, Freire and Ariza-Espinar (2014) misguidedly designated as lectotype the specimen *Lorentz & Hieronymus 117* (CORD). Although the collector number coincides with one of the syntypes, this element is not part of the original material and has to be treated as a neotype.

The typification of W. incisa var. pubescens should be attributed to Cabrera and Freire (1999) although they used the term “isotipo”. With this action the authors presumed that a duplicate was kept at B but the specimen probably was destroyed during the Second World War. On this basis, the term isotype is corrected to lectotype (Turland et al. 2018, ICN Art. 9.10). Accordingly, the later lectotypification by Freire and Ariza-Espinar (2014) becomes superfluous.

#### Additional specimens examined.

**Argentina. Catamarca**: El Cajón, Negroara, 26°24'S, 66°22'W, 17 Jan 1914, *L. Castillón 3360* (LE, LIL); Andalgalá, quebrada de los Cazadores, 27°21'S, 66°8'W, 23 Nov 1948, *R. Filipòvich 61* (LIL); Ambato, sierra de Ambato (falda E), el crestón del cerro Manchado, en los alrededores de La Mancha, 28°14'S, 66°1'W, 28 Mar 1968, *A.T. Hunziker & A.E. Cocucci 20019* (RB); Cerrillos, 1 Mar 1944, *T. Meyer 8282* (LIL); Sierra de Ambato, Portezuelo de Joyango, 28°6'S, 66°2'W, 15 Feb 1910, *L. Roger s.n.* (LIL); Andalgalá, co. Pabellón (Aconquija), 27°13'S, 66°6'W, 19 Feb 1942, *E. Rohmeder s.n.* (LIL); Andalgalá, cerro Pabellón, 27°13'S, 66°6'W, 16 Feb 1942, *E. Rohmeder s.n.* (LIL); cerro Tesoro, 27°7'S, 66°3'W, 7 Sep 1950, *F. Vervoorst 749* (LIL); **Jujuy**: Susques, cerro Tuzgle, 24°3'S, 66°29'W, 2 Mar 1944, *A.L. Cabrera 8370* (CONC); Yavi, cerro Negro, 22°33'S, 65°33'W, 25 Feb 1940, *T. Meyer 18476* (LIL); Tumbaya, Piedra Sonada, 24°21'S, 65°37'W, 19 Sep 1948, *S.A. Pierotti 7508* (LIL); Yavi/Sta. Victoria, cerro Poposayo, cumbre, 22°15'S, 65°15'W, 1 Feb 1953, *H. Sleumer 3684* (LIL); **La Rioja**: Famatina, Sierra de Famatina, mina La Mejicana, 29°0'S, 67°46'W, 27 Apr 1951, *B. Sparre 8827* (LIL); Sierra de Famatina, 1 May 1903, *T. Stuckert 13110* (LIL); **Salta**: abra el Acay, 24°26'S, 66°14'W, 20 Feb 2007, *S. Cuello 154* (LIL); Rosario de Lerma, Piedra Sonada, 24°21'S, 65°37'W, 19 Sep 1948, *S.A. Pierotti 7496* (LIL); Nevado del Cajón, 26°8'S, 66°13'W, 1 Mar 1914, *D. Rodríguez 1383* (BA); Caldera, subida al Nevado del Castillo cerca de Tres Lagunas, 24°13'S, 65°51'W, 16 Mar 1952, *H. Sleumer & F. Vervoorst 3015* (LIL); **Tucumán**: Tafí del Valle, cerro Alto de la Mina, cumbres Calchaquíes, 26°36'S, 65°43'W, 19 Feb 1990, *H. Ayarde 369* (LIL); cerro de las Ánimas, 27°2'S, 65°57'W, 3 Jan 1914, *L. Castillón 3302* (LIL); cerro Muñoz, 26°52'S, 65°50'W, Jan 1916, *L. Castillón s.n.* (BR [mixed with *X. lorochaqui*]); ladera NE del Chimbería, circo del Cochuna, 27°14'S, 66°7'W, 26 Jul 1984, *A. Grau s.n.* (LIL); Tafí, cerro de las Ánimas, 27°2'S, 65°57'W, Dec 1914, *M. Lillo s.n.* (LIL); Tafí, Calchaquíes, morro de la Mina, 26°36'S, 65°43'W, 9 Mar 1952, *B. Sparre 9734* (LIL); Chicligasta, estancia Las Pavas-Sierras Altas, 27°8'S, 66°1'W, 12 Mar 1924, *S. Venturi 2964* (CONC, LIL, US); Chicligasta, cumbre alta del Pueblo Viejo, 15 Dec 1925, *S. Venturi 4643* (LIL); Tafí, sierra del Cajón, Los Chuscos, 7 Feb 1926, *S. Venturi 6646* (US). **Bolivia. Oruro**: Sajama, cerro Jasasuni, 18°9'S, 68°52'W, 27 Mar 2005, *S.G. Beck 31142* (LPB); Sajama, S side of nev. Sajama just below snowline on E side of río Sururia, N walk up from 4300 m where rd. circles nev., 18°8'S, 68°53'W, 19 Apr 1995, *V.A. Funk 11351* (LPB, US); Sebastián Pagador, valley above and E of pueblo of Condo, S of Challapata and E of Huari, 19°0'S, 66°40'W, 21 Apr 1995, *V.A. Funk & M. Estárez 11360* (LPB); Eduardo Abaroa, Challapata, localidad Livichuco, camino a cerro Toro, arriba de la laguna K’asiri, 18°57'S, 66°24'W, 29 Feb 2016, *I. Jiménez et al. 7981* (LPB, USM); Eduardo Abaroa, Challapata, localidad Livichuco, camino a cerro Toro, arriba de la laguna K’asiri, 18°57'S, 66°24'W, 29 Feb 2016, *I. Jiménez et al. 7989* (LPB); Eduardo Abaroa, Azanaque, 18°56'S, 66°42'W, 3 Mar 2016, *I. Jiménez & R. Villegas 8127* (LPB); Sajama, Turco, 18°11'S, 68°12'W, 1993, *J. Mallea 22* (LPB); altiplano central, commune de Condo, 19°2'S, 66°44'W, 7 Apr 1989, *L. Naessany 15* (LPB, US); Cruce Culta, 19°2'S, 66°15'W, 23 Feb 2016, *F. Zenteno 16550* (USM); Avaroa, Livichuco, K’asiri, en línea recta a 6.93 km al SSE, Ayllu Qaqachaqa, 18°56'S, 66°24'W, 29 Feb 2016, *F. Zenteno et al. 16836* (USM); **Potosí**: cordillera Kari Kari, aprox. 3.3 km arriba de la laguna San Sebastián, 19°36'S, 65°41'W, 13 Feb 2019, *J. Calvo & M. Zárate 7869* (BOLV, LPB); cerro of Potosí, Mar 1933, *M. Cárdenas 362* (US); Antonio Quijarro, Vilacollo, loma cerca al lago K’asilla, 22 Dec 2005, *S. Condo Klaus 15* (LPB); prov. Quijarro, Yura, 20°2'S, 66°10'W, Apr 1973, *E.T. de Sahonero s.n.* (US); Sud Lípez, cerro Tapaquillcha, 21°31'S, 67°53'W, 11 Apr 1980, *M. Liberman 152* (LPB); José M. Linares Lizarazu, comunidad Alkatuyo, cerro Ichurata, 53 km SE de Potosí, 17.3 km al N de la escuela de Alkatuyo, 19°53'S, 65°33'W, 20 May 1993, *F. Marino 183* (LPB); Sud Lípez, Tapaquillcha, 21°31'S, 67°53'W, 2 Oct 2001, *B.J. Ruthsatz 10570* (LPB); SE de Uyuni, 5 May 1993, *M. Sauvain 828* (LPB); Tomás Frías, serranía Kari-Kari, alrededores de lagunas de Kari-Kari, 19°36'S, 65°41'W, 28 Feb 2006, *M. Zárate 2212* (BOLV). **Chile. Antofagasta**: El Loa, quebrada del Inca, cerro Aucanquilcha, 21°14'S, 68°28'W, 2 Apr 1985, *M. Arroyo 85-595* (CONC); El Loa, camino a portezuelo del Cajón, cerro Toco, ladera N, 22°55'S, 67°46'W, 3 Apr 1997, *M. Arroyo, L. Cavieres & A. Humaña 97022* (CONC); El Loa, sector géiser del Tatio, hacia al E, casi en el límite con Bolivia, 22°20'S, 67°59'W, 19 May 1997, *M. Baeza, P. Aqueveque & G. Kottirsch 588* (CONC); Chiu Chiu, Inacaliri, cerros de Colana, 21°56'S, 68°7'W, 15 May 2018, *J. Calvo 7731* (CONC, SGO); Loa, Toconao, cordón S de los cerros de La Pacana, 23°9'S, 67°26'W, 4 Mar 2019, *J. Calvo 7909* (SGO); Ollagüe, volcán Aucanquilcha, ladera NE, cerca del campamento Aucanquilcha, 21°11'S, 68°26'W, 23 Apr 2019, *J. Calvo & A. Moreira-Muñoz 7935* (SGO); El Loa, Toconce, 22°15'S, 68°10'W, Apr 1993, *L. Loyola 93-4* (CONC); **Arica-Parinacota**: Parinacota, cerro Guane Guane, 18°10'S, 69°15'W, 18 Apr 1980, *M. Arroyo, C. Villagrán & J. Moreno 2622* (CONC); Parinacota, cerro Choquelimpie, 18°16'S, 69°13'W, 19 Apr 1984, *M. Arroyo 84-913* (CONC); Parinacota, Guane Guane, 18°10'S, 69°15'W, 7 Apr 1988, *E. Belmonte 88090* (CONC); bajando desde Portezuelo Taapacá hacia Putre, en el límite comunal, 18°6'S, 69°32'W, 20 Oct 2012, *A. Moreira-Muñoz et al. 2038* (SGO); Las Cuevas, Chaku Vilañuñumani, 18°11'S, 69°26'W, 20 Mar 2015, *A. Moreira-Muñoz & F. Luebert 2469* (SGO); Las Cuevas, antes del Chaku, 18°11'S, 69°25'W, 20 Mar 2015, *A. Moreira-Muñoz & F. Luebert 2471* (SGO); arriba de Las Cuevas, cerro Milagro, 18°13'S, 69°27'W, 19 Jun 2015, *A. Moreira-Muñoz 2500* (SGO); Aguas Calientes, Tacora, 17°43'S, 69°49'W, 17 Sep 1955, *M. Ricardi 3370* (CONC). **Peru. Arequipa**: Castilla, Orcopampa, minas de Poracota, alrededor de laguna Tintarcocha, 15°12'S, 72°32'W, 20 Apr 2011, *H. Beltrán 7102* (USM); La Unión, Cotahuasi, 15°14'S, 72°52'W, 30 Jun 2002, *F. Cáceres 2807* (HUSA, USM); Caylloma, 15°38'S, 71°35'W, 19 Mar 2006, *F. Cáceres 5508* (HUSA); dist. Chiguata, 16°24'S, 71°22'W, 29 Jun 2006, *F. Cáceres 5732* (HUSA); Chiguata, faldas del nevado Pichu Pichu, 16°23'S, 71°17'W, 25 Apr 2004, *V. Quipuscoa et al. 2947* (HSP); Castilla, Tapay, cerro Blanco, Apacheta, 17 Sep 2011, *N. Vega 1785* (USM); **Ayacucho**: Coracora, Parinacochas, Alqaywacho, Hurayhuma, 14°38'S, 73°36'W, 13 May 2000, *F. Pietrellini 193* (USM); **Cusco**: Espinar, hda. Kachcachi (Uchupata), 14°52'S, 71°20'W, 22 Jun 1956, *C. Vargas 11222* (CUZ, LPB, US); **Junín**: cordilleras entre Huancayo y Yauyos, 12°11'S, 75°38'W, [without date], *A. Weberbauer s.n.* (MOL); **Lima**: Huarochirí, Huachupampa, 11°43'S, 76°33'W, 28 Aug 1993, *J. Albán & G. Yarupaitán 8094* (USM); Yauyos, Laraos, camino Jalcacha a Palca, 12°20'S, 75°43'W, 25 May 1995, *H. Beltrán 1715* (USM); Yauyos, Laraos, camino de Jalcacha a Palca, 12°24'S, 75°45'W, 4 Nov 1992, *H. Beltrán 414* (USM); Huarochirí, Huillpa, 11°51'S, 76°21'W, [without date], *I. Espinoza 70* (USM); Huarochirí, San Damián, Chanape, 11°53'S, 76°15'W, 5 Jul 2013, *P. Gonzáles & B. Brito 2642* (USM); Huarochirí, San Pedro de Casta, 11°45'S, 76°35'W, Dec 1982, *A. Holzapfel 17* (USM); Auquimarca, 10°24'S, 76°59'W, Jan 1949, *A. Peraldo 3794* (LIL); Canta, Mishquipuquio, cerca a Canta, 11°34'S, 76°33'W, Aug 1949, *S. Sánchez 36* (USM); Canta, Lachaqui, Cieneguilla por el camino a Tararhua, 22 Jul 2001, *G. Vilcapoma 5540* (USM); Canta, Huascoy, 11°17'S, 76°45'W, 27 Oct 1974, *P. Waechter s.n.* (USM); Huarochirí, San Juan de Iris, 11°41'S, 76°31'W, 18 Aug 1993, *G. Yarupaitán & J. Albán 1065* (USM); Huarochirí, Huachupampa, lugar conocido como Armas, 11°43'S, 76°33'W, 4 Jul 1993, *G. Yarupaitán & J. Albán 954* (USM); **Moquegua**: Mariscal Nieto, Carumas, cerca a un acueducto, 16°50'S, 70°31'W, 15 Jun 2013, *H. Beltrán 7736* (USM); across the carretera-binacional from laguna Suches, 9 km to the W from the turn off to laguna Suches, 16°53'S, 70°26'W, 12 Mar 2014, *V.A. Funk, M. Diazgranados & E. Cochachin 13147* (US, USM); across the carretera-binacional from laguna Suches, 9 km to the W from the turn off to laguna Suches, 16°53'S, 70°26'W, 12 Mar 2014, *V.A. Funk, M. Diazgranados & E. Cochachin 13148* (US, USM); across the carretera-binacional from laguna Suches, 9 km to the W from the turn off to laguna Suches, 16°53'S, 70°26'W, 12 Mar 2014, *V.A. Funk, M. Diazgranados & E. Cochachin 13149* (USM); across the carretera-binacional from laguna Suches, 10 km to the W from the turn off to laguna Suches, 16°53'S, 70°26'W, 12 Mar 2014, *V.A. Funk, M. Diazgranados & E. Cochachin 13150* (US); across the carretera-binacional from laguna Suches, 10 km to the W from the turn off to laguna Suches, 16°53'S, 70°26'W, 12 Mar 2014, *V.A. Funk, M. Diazgranados & E. Cochachin 13151* (US); area between the carretera-binacional and the interoceanica sur, on unpaved road that connects the two main roads and borders a large bofedal, 16°50'S, 70°32'W, 12 Mar 2014, *V.A. Funk, M. Diazgranados & E. Cochachin 13155* (US, USM); near unpaved road in mining concession, 16°39'S, 70°1'W, 15 Mar 2014, *V.A. Funk, M. Diazgranados & E. Cochachin 13167* (US, USM); above unpaved mining road that is high above the valley [pr. Paripiña, according to coordinates], 16°38'S, 70°1'W, 15 Mar 2014, *V.A. Funk, M. Diazgranados & E. Cochachin 13168* (USM); General Sánchez Cerro, Ubinas, cumbre nevada del cerro Pirhuani Querala, 16°9'S, 70°43'W, 7 Apr 2011, *D. Montesinos 3098* (HSP, USM); General Sánchez Cerro, Yunga, Choco-Choco, 16°15'S, 70°37'W, 11 Sep 2012, *D. Montesinos 3933* (USM); General Sánchez Cerro, Ubinas, Chaclaya, parte baja del cerro Chaclaya, 16°10'S, 70°53'W, 14 Sep 2005, *D. Montesinos 549* (USM); Mariscal Nieto, Calacoa, faldas Ticsani, SE, 16°44'S, 70°35'W, 4 Mar 2018, *D. Montesinos & J. Calvo 6004* (HSP); **Tacna**: Tarata, Poma, carretera Tarata-Puno, 17°25'S, 69°56'W, 4 Dec 1997, *A. Cano 7944* (USM); Tarata, Poma, carretera Tarata-Puno, 17°25'S, 69°56'W, 4 Dec 1997, *A. Cano 7953* (USM); 32 km N of Tarata, ca. 50 km S of abra Livini (Puno), road from Tarata to Mazo-Cruz (Puno), rd. 36, 17°26'S, 69°57'W, 11 Mar 2014, *V.A. Funk, M. Diazgranados & E. Cochachin 13138* (US, USM); rd. from Tarata to abra Livini (Puno), 0.5 km before intersection with rt. 36, 17°25'S, 69°56'W, 11 Mar 2014, *V.A. Funk, M. Diazgranados & E. Cochachin 13142* (US); Tarata, cordillera del Barroso, carretera Alto Perú-Palca, 17°33'S, 69°51'W, 7 Dec 1997, *J. Roque 559* (USM [mixed with *X. juniperinum*]); bajando de Livini hacia Tarata, 17°26'S, 70°0'W, 28 Nov 1959, *C. Vargas 13016* (CUZ, LPB, US).

### 14. Xenophyllum lorochaqui

Taxon classificationPlantaeAsteralesAsteraceae

J.Calvo & V.A.Funk, PhytoKeys: 139: 34. 2020.

1464463C-88DC-5CC3-8324-95B48B570E53

#### Type.

Argentina. Catamarca: El Cajón, Negroara, 15 Jan 1914, *L. Castillón 3365* (holotype: LIL-26677!; isotypes: BM s.n.!, BR s.n.!, US-00622893!, W-334!).

#### Description.

**Figure 21.**
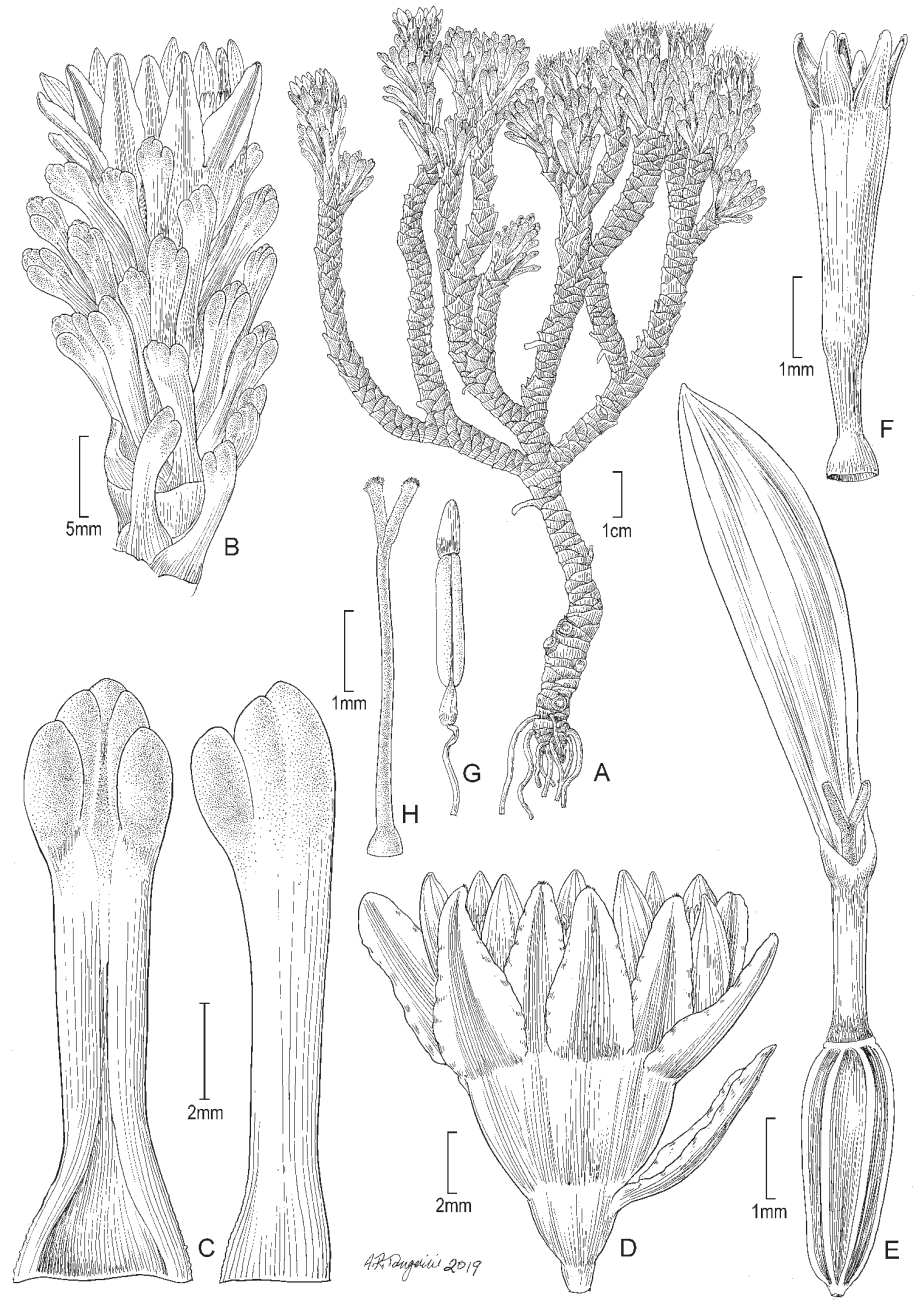
*Xenophyllum lorochaqui***A** habit **B** stem apical part and capitulum **C** adaxial leaf surface (left hand) and vertical leaf profile (right hand) **D** capitulum **E** ray floret (pappus removed) **F** disc corolla **G** stamen **H** style. All details drawn from *Castellanos s.n.* (BA) except for **A, B** (drawn from *Díaz s.n.*, GH). Illustration by Alice Tangerini.

Suffruticose plant, forming clumps of erect stems. ***Rhizomes*** 5–10 × 0.6–0.8 cm, horizontal to oblique, glabrous. ***Stems*** 10–20 cm tall, branched, glabrous, usually with leaves restricted to the upper part. ***Leaves*** imbricate, extending into a sheath-like base glabrescent or with evanescent arachnoid trichomes; leaf laminas 7.9–11.5 × 2.3–2.7 mm, linear, broadened at the apex, 3-notched at the apex, with the central lobe longer than lateral ones, entire, curved forwards in cross section, glabrous, unconspicuously nerved on both faces, fleshy, matte; central leaf lobe 1–1.6 mm long (lateral ones 0.5–0.8 mm long), 1.3–1.6 mm wide at the maximum width point, entire or barely notched, obtuse. ***Capitula*** radiate, erect, sessile. ***Involucres*** 10.3–10.9 × 6.6–8.7 mm, cupuliform; involucral bracts ca. 13, 5.9–7 × 1.4–2.5 mm, obtuse at the apex, greenish. ***Ray florets*** 26 to 39; corollas 8.3–9.2 × 1.1–1.4 mm, 4-veined, subentire to 3-toothed at the apex, conspicuously surpassing the involucre, white. ***Disc florets*** 40 to 57; corollas 4.2–5.5 mm long, yellowish; style branches truncate with a crown of sweeping trichomes, yellowish. ***Achenes*** 2.5–3.1 × 0.7–0.9 mm, cylindrical, 8 to 9-ribbed, glabrous; pappus 4.3–6.1 mm long, barbellate, whitish. Chromosome number unknown. Figs 19A, 21.

#### Distribution and habitat.

Endemic to Argentina (Catamarca, Jujuy, Salta, Tucumán). This species grows in exposed rocky slopes and on bare soils of the dry puna ecoregion, between elevations of 3500–5000 m (Fig. 20).

#### Phenology.

Flowering from January to March.

#### Etymology.

The epithet *lorochaqui* is the vernacular name of this plant and it means parrot’s foot (“loro” from Spanish: parrot; “chaqui” from Quichua: foot). It probably responds to the resemblance of the leaves with a parrot’s foot.

#### Notes.

*Xenophyllum lorochaqui* can be identified by the glabrous erect stems 10–20 cm tall (usually only bearing leaves in the upper part), leaf lamina 7.9–11.5 mm long, leaf apex 3-notched with the central lobe entire or barely notched and longer than the lateral ones, involucre with ca. 13 involucral bracts, and by displaying 26 to 39 ray florets with white corollas.

This species shows morphological affinities with *X. incisum*, *X. dactylophyllum*, and *X. poposa*. The differences against *X. incisum* are the leaf lamina length (3.2–7.3 mm vs. 7.9–11.5 mm in *X. lorochaqui*), the length of the leaf apex lobes (similar among them vs. central lobe longer than lateral ones in *X. lorochaqui*), the involucre length (6.4–8.3 mm vs 10.3–10.9 mm in *X. lorochaqui*), the involucral bract number (8 to 9 vs. ca. 13 in *X. lorochaqui*), and the ray floret number (8 to 13 vs. 26 to 39 in *X. lorochaqui*). With regard to *X. dactylophyllum*, the leaf apex is at least 9-divided (finger-like) with the primary division extending deeper than the subsequent ones, whereas in *X. lorochaqui* the leaf apex is 3-notched with the central lobe longer than lateral ones. From *X. poposa*, the distinguishable characters are the stem indumentum (arachnoid in *X. poposa* vs. glabrous in *X. lorochaqui*), the leaf lamina length (2.5–7.1 mm in *X. poposa* vs. 7.9–11.5 mm in *X. lorochaqui*), the involucral bract number (8 to 9 in *X. poposa* vs. ca. 13 in *X. lorochaqui*), and the ray floret number (8 to 11 in *X. poposa* vs. 26 to 39 in *X. lorochaqui*). Among these species, the distribution area of *X. lorochaqui* partially overlaps with that of *X. incisum* and *X. poposa*.

#### Additional specimens examined.

**Argentina. Jujuy**: Tumbaya, cerro Moreno, 23°46'S, 65°44'W, 7 Feb 1929, *S. Venturi 9289* (US); **Salta**: Cafayate, sierra de los Quilmes, 26°11'S, 66°4'W, 28 Jan 1943, *A. Castellanos s.n.* (BA); abra del Gallo, ca. 40 km al SW de S. Antonio de los Cobres, en el camino a Pastos Grandes, 24°20'S, 66°30'W, 17 Dec 1946, *A. Krapovickas 3215* (LIL, SI); nevado del Cajón, 26°8'S, 66°13'W, 1 Mar 1914, *D. Rodríguez 1382* (BA, BR); **Tucumán**: cerro Muñoz, 26°52'S, 65°50'W, Jan 1916, *L. Castillón s.n.* (BR [mixed with *X. poposa*]); Tafí, cumbres de San José (La Mina), 26°41'S, 65°40'W, Mar 1933, *Díaz s.n.* (GH, LIL); Tafí, cumbre de Chasquivil, 26°41'S, 65°40'W, 12 Jan 1945, *D. Olea 252* (LIL); Chicligasta, estancia Las Rosas, 15 Jan 1927, *S. Venturi 6342* (US); Tafí, sierra del Cajón, Los Chuscos, 7 Feb 1926, *S. Venturi 6647* (US).

### 15. Xenophyllum rosenii

Taxon classificationPlantaeAsteralesAsteraceae

(R.E.Fr.) V.A.Funk, Novon 7(3): 240. 1997.

F612FD8D-FD09-51AA-804F-4B7D397D746D

Werneria rosenii R.E.Fr., Nova Acta Regiae Soc. Sci. Upsal., ser. 4, 1(1): 90. 1905 [“Rosenii”]. Type. Argentina. Jujuy: nevado de Chañi, 5200 m, 29 Nov 1901, *R.E. Fries 862* (lectotype: Fries’ collection at UPS as the first-step lectotype, designated as “holotype” by Funk (1997a: 240); UPS-V-833144 (digital image!) as the second-step lectotype, designated here; isolectotypes: CORD-00005639 (digital image!), P-02088549 (digital image!), S-R-6527 (digital image!), UPS-V-833133 (digital image!), US-00037307!).

#### Description.

Suffruticose plant, forming clumps of erect stems. ***Rhizomes*** 6–10 × 0.4–0.5 cm, horizontal to oblique, glabrous. ***Stems*** 4–8 cm tall, simple or branched, glabrous, usually with leaves restricted to the upper part. ***Leaves*** imbricate, extending into a glabrous sheath-like base; leaf laminas 7–11 × 3–4.5 mm, linear-spatulate (broadened upwards), 2-forked at the apex (incision depth ca. 1/3 of the total length), entire, flat to elliptic or almost terete upwards in cross section, glabrous, 1-nerved above (barely visible), 1-nerved beneath, fleshy, matte; leaf lobes 2.3–4.5 × 1–1.5 mm, acute to obtuse. ***Capitula*** radiate, erect, sessile. ***Involucres*** 11–13.2 × 8–10 mm, cupuliform; involucral bracts 11 to 12, 5.6–7.2 × 1.9–3.2 mm, rather acute at the apex, greenish, purple-edged. ***Ray florets*** ca. 18; corollas ca. 9.2 × 1.7 mm, 3 to 4-veined, subentire to 3-toothed at the apex, conspicuously surpassing the involucre, white. ***Disc florets*** ca. 50; corollas 5–5.1 mm long, yellowish; style branches penicillate, purplish. ***Achenes*** ca. 3.5 × 0.9 mm, cylindrical, 6 to 8-ribbed, glabrous; pappus 8–10 mm long, barbellate, whitish. Chromosome number unknown.

#### Iconography.

Cabrera (1948: 52, fig. 2, sub *Werneria rosenii*).

#### Distribution and habitat.

Restricted to northwestern Argentina and southwestern Bolivia. Argentina (Jujuy, Salta [expected]), Bolivia (Potosí). It grows in wet meadows and rocky places of the dry puna ecoregion, between elevations of 4600–5200 m (Fig. 20).

*Xenophyllum rosenii* is hitherto known from very few gatherings, i.e., the type locality in Jujuy (Argentina) plus two collections from southern Sur Lípez (Bolivia). Jørgensen (2014) also indicated this species from Oruro (Bolivia) on the basis of a misidentified collection that actually corresponds to *X. digitatum* (*Liberman 352*, LPB).

#### Phenology.

Collected in flower in March and November.

#### Etymology.

It is named after Eric von Rosen (1879–1948), a Swedish aristocrat, explorer, and ethnographer.

#### Notes.

*Xenophyllum rosenii* is unique in the genus by its 2-forked leaves. The species has been confused with *X. digitatum* (e.g., *Meneses 5446*, LPB; *Treviño 761*, HSP; *Tupayachi 736*, US). A detailed study of the leaves highlights that these species can be straightforwardly discriminated from one another by the leaf apex (2-forked in *X. rosenii* vs. 3-forked in *X. digitatum*). The other characters appear to be useless because of a significant overlapping among them.

Since two specimens of the gathering *Fries 862* were found at UPS, the lectotypification has been further narrowed to a single specimen by way of a second-step lectotype (UPS-V-833144).

#### Additional specimens examined.

**Bolivia. Potosí**: Sur Lípez, San Antonio de Lípez, 21°52'S, 66°56'W, 1966, *A.F.G. Cope s.n.* (K, US); Sud Lípez, 5.5 mi SW of San Antonio de Lípez (Viejo) on road towards Quetena Grande, 21°52'S, 66°56'W, 20 Mar 1993, *P.M. Peterson, R.J. Soreng & S. Laegaard 13040* (US).

### 16. Xenophyllum digitatum

Taxon classificationPlantaeAsteralesAsteraceae

(Wedd.) V.A.Funk, Novon 7(3): 239. 1997.

90427A97-FAFF-5FD3-A28D-BCCE39C082C9

Werneria digitata Wedd., Chlor. Andina 1: 86. 1856. Type. Bolivia. Potosí: del nivel de las nieves de la quebrada de las lagunas de Potosí, Mar [without year], *A. d’Orbigny 1407* (lectotype: P-02088550 (digital image!), designated by Funk (1997a: 239); isolectotypes: BR s.n.!, F-102321 (fragment!), P-02088551 (digital image!)).
Werneria digitata var. lanata Rockh., Bot. Jahrb. Syst. 70: 287. 1939. Type. Peru. Junín: An der Lima-Oroya-Bahn, hacienda San Florenzi bei Yauli, 4700 m, [“Jan 1902” according to the protologue], *A. Weberbauer 356* (lectotype: G-00237251 (digital image!), designated here), syn. nov.

#### Description.

Suffruticose plant, forming clumps of erect stems. ***Rhizomes*** 5–12 × 0.5–0.9 cm, horizontal to oblique, glabrous. ***Stems*** 10–13 cm tall, simple or branched, glabrous, usually with leaves restricted to the upper part. ***Leaves*** imbricate, extending into a glabrous sheath-like base; leaf laminas 7.7–11.5 × 2.8–6.7 mm, linear-spatulate (broadened upwards), 3-forked at the apex (incision depth 1/3–1/2 of the total length), entire, usually with some minute cilia in the lower half, flat to elliptic or almost terete upwards in cross section, glabrous or arachnoid above (evanescent), 1-nerved above (barely visible), 1-nerved beneath, fleshy, matte; leaf lobes 3.6–5.9 × 0.8–1.2 mm, entire or 2(–3)-divided, acute to obtuse, sometimes mucronate. ***Capitula*** radiate, erect, sessile to subsessile. ***Involucres*** 12.1–16.7 × 9.8–15.7 mm, cupuliform; involucral bracts 12 to 13, 6.3–9.3 × 2.4–3.9 mm, obtuse at the apex, greenish, purple-edged. ***Ray florets*** 21 to 36; corollas 9.2–14.1 × 1.4–1.9 mm, 4 to 5-veined, subentire to 3-toothed at the apex, conspicuously surpassing the involucre, white. ***Disc florets*** 49 to 63; corollas 5.5–7.2 mm long, yellowish; style branches penicillate, purplish. **Achenes** 3.5–3.9 × 1–1.1 mm, cylindrical, 7 to 8-ribbed, glabrous; pappus 6.7–8.9 mm long, barbellate, whitish to partially rose-colored. Chromosome number 2*n* = 104(±4) (Diers 1961). Fig. 2D.

#### Additional iconography.

Weddell (1856: pl. 17D, sub *Werneria digitata*); Ricardi and Marticorena (1966: 26, fig. 6, sub *W. pseudodigitata*); Beltrán (2016: 359, fig. 3B, as photo); Gonzáles et al. (2016: 169, fig. 1H, I, sub *X. pseudodigitatum*, as photo).

#### Distribution and habitat.

Central Peru to northern Chile. Bolivia (Oruro, Potosí), Chile (Antofagasta, Arica-Parinacota, Tarapacá), Peru (Apurímac [expected], Arequipa, Ayacucho, Cusco, Huancavelica, Junín, Lima, Moquegua, Puno). It grows in wet rocky outcrops, marsh and stream edges, and on cryoturbated soils of the puna ecoregion, between elevations of 3700–5300 m (Fig. 23).

#### Phenology.

Flowering nearly all year round.

#### Etymology.

The adjective *digitatus -a -um* refers to the shape of the leaves, which have divisions arranged like those of a bird’s foot (3-forked).

#### Notes.

*Xenophyllum digitatum* is characterized by its 3-forked leaves, where each lobe can be, in turn, 2 to 3-divided. The depth of the primary division reaches 1/3–1/2 of the total leaf length and the leaf margin usually have some cilia in the lower half. It has quite large involucre (12.1–16.7 × 9.8–15.7 mm) with 12 to 13 involucral bracts, 21 to 36 ray florets (with white corollas conspicuously surpassing the involucre), 49 to 63 disc florets, and glabrous achenes.

It is a variable species mainly concerning leaf lobe shape and leaf indumentum. Typical forms have entire leaf lobes but some specimens display 2-divided lobes (*Meneses 5446*, LPB; *Yager 4697*, LPB), or even 3-divided (*Humbert 30793*, USM; *Hutchinson 1212*, CONC, F, GH, LE, NY, UC, US, USM). The leaf lobe apex is acute to obtuse, although populations with clearly mucronate apex also exist such as those from Ausangate in Cusco (e.g., *Humbert s.n.* [Vargas 12143], US, CUZ). The leaf lamina is usually glabrous but specimens with evanescent arachnoid indumentum on the adaxial surface are also found. Based on this latter character, Rockhausen (1939) described the plants with indumentum as Werneria digitata var. lanata Rockh. We consider it as part of the variability encompassed by *X. digitatum*, and accordingly, Rockhausen’s name is treated as a heterotypic synonym.

This species has been confused with *X. pseudodigitatum* (Rockh.) V.A.Funk and *X. rosenii*. For details on their morphological differences see the comments under the respective species.

#### Additional specimens examined.

**Bolivia. Oruro**: Sajama, ladera E del río Sururia, 18°13'S, 68°54'W, 7 May 1981, *M. Liberman 352* (LPB); Sajama, al sur del volcán Sajama, 18°7'S, 68°52'W, 1 May 1993, *M. Sauvain 827* (LPB); Avaroa, Cruce Ventilla, Tirani, en línea recta a 13.1 km al W, Ayllu Puraca, 19°4'S, 66°19'W, 26 Feb 2016, *F. Zenteno 16686* (USM); **Potosí**: cordillera Kari Kari, aprox. 3.3 km arriba de la laguna San Sebastián, 19°36'S, 65°41'W, 13 Feb 2019, *J. Calvo & M. Zárate 7871* (BOLV); Sud Lípez, cerro Tapaquillcha, ladera S, 21°31'S, 67°53'W, 13 Apr 1980, *M. Liberman 191* (LPB); Tomás Frías, aprox. 4 km saliendo por carretera Potosí-Tarija, cordillera de la ciudad de Potosí, 19°36'S, 65°41'W, 28 Feb 2006, *M. Zárate 2216* (BOLV, LPB). **Chile. Antofagasta**: El Loa, el Tatio, 22°20'S, 68°1'W, 20 Oct 1976, *M. Castillo s.n.* (CONC); El Tatio, quebrada cerca del campamento de la CORFO, 22°22'S, 68°0'W, 26 Apr 1969, *M. Mahu 4108* (SGO); El Loa, Ujina, 20°58'S, 68°37'W, 17 Jan 1943, *E. Pisano & J. Venturelli 1724* (CONC, SGO); **Arica-Parinacota**: Parinacota, cerro Guane Guane, 18°10'S, 69°15'W, 20 Apr 1980, *M. Arroyo, C. Villagrán & J. Moreno 2660* (CONC); Parinacota, quebrada Cataguanchuta, 18°14'S, 69°31'W, 17 Dec 1988, *E. Belmonte 88668* (CONC); Portezuelo de Chapiquiña, faldeos al lado norte del campamento, 18°19'S, 69°30'W, 10 Feb 1964, *C. Marticorena, O. Matthei & M. Quezada 110* (CONC); quebrada Ancochallane, 18°15'S, 69°24'W, 18 Jun 2015, *A. Moreira-Muñoz 2495* (SGO); Portezuelo de Chapiquiña, 18°19'S, 69°30'W, 26 Mar 1961, *M. Ricardi, C. Marticorena & O. Matthei 201* (B, CONC); **Tarapacá**: Iquique, Collaguasi, quebrada La Represa, 20°59'S, 68°38'W, 22 Jan 1993, *S. Teillier 3011* (CONC). **Peru. Arequipa**: Castilla, Orcopampa, minas de Poracota, cerca a quebrada Faculla, 15°14'S, 72°33'W, 20 Apr 2011, *H. Beltrán 7111* (USM [mixed with *X. ciliolatum*]); Castilla, Orcopampa, minas de Poracota, cerca a quebrada Faculla, 15°14'S, 72°32'W, 20 Apr 2011, *H. Beltrán 7114* (USM); Condesuyos, Salamanca, bofedal de Tintarcocha, 15°13'S, 72°30'W, 26 Mar 2012, *I. Treviño 761* (HSP); **Ayacucho**: Huamanga, Ocollo, hacia abra Apacheta, 13°29'S, 74°28'W, 26 Jun 2010, *A. Cano et al. 19873* (USM); **Cusco**: Anta, Mollepata, 13°24'S, 72°43'W, 12 May 2013, *H. Beltrán 7672* (USM); cordillera de Vilcanota, camino al cerro Orqo Q’ocha, 13°45'S, 71°4'W, Mar 2008, *C. García 86* (LPB); cordillera de Vilcanota, camino al cerro Orqo Q’ocha, 13°45'S, 71°4'W, Mar 2008, *C. García 87* (LPB); Paucartambo, hacienda Churu, Jan 1926, *F.L. Herrera 1033* (CONC, F, GH, MO, NY); Quispicanchi, Paucartambo, hacienda Ccapana, 13°34'S, 71°25'W, Apr 1926, *F.L. Herrera 1056* (F); Quispicanchi, Paucartambo, hacienda Ccapana, 13°34'S, 71°25'W, Apr 1926, *F.L. Herrera 1081* (BM, CONC, F, GH, MA, NY); cordillera del Auzangate, paso de Hualla-Hualla, 13°38'S, 71°6'W, 28 May 1958, *H. Humbert et al. 30793* (USM); Quispicanchi, laguna Ampatuni [Ampatune], 13°35'S, 71°10'W, 29/30 May 1958, *H. Humbert s.n.* [C. Vargas 12143] (CUZ, US); cordillera de Vilcanota, cuenca de la laguna Sibinacocha, cerro Rititica, 13°45'S, 71°4'W, 5 Apr 2012, *R.I. Meneses et al. 5446* (LPB); cordillera de Vilcanota, cuenca de la laguna Sibinacocha, cerro Orqo Q’ocha, 13°45'S, 71°4'W, 7 Apr 2012, *R.I. Meneses et al. 5467* (LPB); Urubamba, laguna de Kellococha, proximidades de los nevados Balcunniyuc e Yllahuamán, 13°12'S, 72°15'W, 20 Aug 1988, *A. Tupayachi 736* (US); cordillera de Vilcanota, Sibinacocha, 13°45'S, 71°4'W, 12 Mar 2008, *K. Yager 4697* (LPB); **Huancavelica**: Huaytará, Pilpichaca (abra Apacheta), 13°20'S, 74°44'W, 4 Jul 2010, *A. Cano, W. Mendoza & A. Delgado 19686* (USM); Huaytará, Pilpichaca (abra Apacheta), 13°20'S, 74°43'W, 4 Jul 2010, *A. Cano, W. Mendoza & A. Delgado 19734* (USM); Angaraes, Ccochaccasa, 12°56'S, 74°47'W, 4 Apr 2014, *A. Cano, B. Britto & N. Valencia 21926* (USM); Huaytará, alrededores de abra Apacheta, 13°18'S, 74°41'W, 3 Sep 2013, *P. Gonzáles et al. 2781* (USM); Huachocolpa, alrededores de la unidad minera Caudalosa, 13°4'S, 75°0'W, 23 Mar 2015, *P. Gonzáles 3559* (USM); Huachocolpa, alrededores de la unidad minera Caudalosa, 13°4'S, 75°0'W, 23 Mar 2015, *P. Gonzáles 3562* (USM); nevado Ajchi, Aug 1961, *O. Tovar 3423* (USM); **Junín**: Anticona pass, ca. 140 km E of Lima, 11°35'S, 76°11'W, 21 Dec 1978, *M. Dillon & B.L. Turner 1478* (USM); Lima-La Oroya highway, cumbre at top of divide, above Morococha, 11°36'S, 76°8'W, 29 Jun 1982, *A. Gentry & R. Tredwell 37301* (USM); Yauli, paso de Ticlio, carretera Lima-Oroya, 11°35'S, 76°11'W, 6 Dec 1960, *E. Pallardelly 1* (USM); abra de Ticlio, 11°35'S, 76°11'W, Dec 1986, *S. Rivas et al. s.n.* (USM); Anticona, abajo entre Casapalca y Oroya, 11°35'S, 76°11'W, 26 Jun 1954, *O. Tovar & L. Constance 9194* (USM-29378 [mixed with *X. ciliolatum* and *X. decorum*], USM-29379, USM-34159); Yauli, Ticlio, 11°35'S, 76°11'W, 28 Jun 1999, *G. Yarupaitán 1690* (US); **Lima**: Yauyos, Laraos (zona de Pacocha e Ipillo), 12°20'S, 75°43'W, 20 Nov 1996, *H. Beltrán 2579* (USM); Huarochirí, paso de Anticona, 11°35'S, 76°11'W, 1 May 1999, *H. Beltrán & E. Li 3305* (USM); Yauyos, Laraos, Viscollo, 12°25'S, 75°36'W, 12 May 2001, *H. Beltrán 4193* (USM); Anticona, límite entre Lima y Junín, 11°27'S, 76°13'W, 24 Jan 2004, *H. Beltrán 5863* (USM); Huarochirí, Chicla, abra Anticona (Ticlio), 11°36'S, 76°11'W, 29 Apr 2017, *H. Beltrán, S. Castillo & M. Arakaki 7964* (USM); Huarochirí, Chicla, abra Anticona (Ticlio), 11°35'S, 76°11'W, 29 Apr 2017, *H. Beltrán, S. Castillo & M. Arakaki 7989* (USM); límite con dpto. Junín, entre Casapalca y Ticlio, 11°35'S, 76°11'W, 1 Dec 1977, *S. Castroviejo, M. Costa & E. Valdés-Bermejo 1120* (MA); puerto de Ticlio, entre Casapalca y Morococha, 11°35'S, 76°11'W, 6 Mar 1979, *A. Ceballos et al. 8* (MA); Huarochirí, Ticlio, 11°35'S, 76°11'W, 5 Mar 1966, *E. Cerrate, C. Acleto & J. Gómez 4289* (USM); La Oroya, Anticona, 11°35'S, 76°10'W, 25 Apr 1971, *E. Cerrate et al. 4907* (USM); Ticlio, top of divide between Lima and La Oroya, 11°35'S, 76°11'W, 29 Jan 1983, *A. Gentry et al. 39749* (USM); Huarochirí, San Damián, Chanape, 11°53'S, 76°15'W, 5 Jul 2013, *P. Gonzáles & B. Brito 2629* (USM); Huarochirí, Ticlio, canyon of the río Rimac, on the carretera central, 11°35'S, 76°11'W, 19 Aug 1957, *P.C. Hutchison 1212* (CONC, F, GH, LE, NY, UC, US [all specimens mixed with *X. decorum*], USM); Anticona, 11°35'S, 76°14'W, 5 Aug 2012, *E. Linares & A. Galán 3099* (USM); Anticona, 11°35'S, 76°11'W, 4 Aug 2012, *E. Linares & A. Galán 3100* (USM); Ticlio, 11°35'S, 76°11'W, Feb 1974, *O. Tovar 7191* (USM); Huarochirí, Ticlio, 11°35'S, 76°11'W, Nov 1975, *F. Weberling 5891* (USM); Huarochirí, San Juan de Iris, bajando por la cumbre de Tres Cruces, 11°41'S, 76°31'W, 18 Aug 1993, *G. Yarupaitán & J. Albán 1063* (USM); Huarochirí, San Juan de Iris, bajando de la cumbre de Tres Cruces, 11°41'S, 76°31'W, 18 Aug 1993, *G. Yarupaitán & J. Albán 1067* (USM); Huarochirí, paso de Anticona, Ticlio, 11°35'S, 76°11'W, 29 Oct 1994, *G. Yarupaitán 1501* (USM); **Moquegua**: above unpaved mining road that is high above the valley [pr. Paripiña, according to coordinates], 16°38'S, 70°1'W, 15 Mar 2014, *V.A. Funk, M. Diazgranados & E. Cochachin 13169* (US, USM); near former bofedal that is now mostly dead as a result of the mining operation [pr. Paripiña, according to coordinates], 16°39'S, 70°1'W, 15 Mar 2014, *V.A. Funk, M. Diazgranados & E. Cochachin 13171* (US, USM); Mariscal Nieto, Calacoa, pr. Achacalane, 16°41'S, 70°33'W, 4 Mar 2018, *D. Montesinos & J. Calvo 5999* (HSP); **Puno**: Carabaya, Quelcaya, 13°58'S, 70°50'W, 15 Feb 2009, *E. Mondragón & J. Postigo 90* (USM); San Antonio de Esquilache, 16°6'S, 70°17'W, 18 May 1937, *D. Stafford 748* (BM, F, K).

### 17. Xenophyllum pseudodigitatum

Taxon classificationPlantaeAsteralesAsteraceae

(Rockh.) V.A.Funk, Novon 7(3): 240. 1997.

4610516D-344A-5869-BFAB-108791C74E27

Werneria pseudodigitata Rockh., Bot. Jahrb. Syst. 70: 288. 1939. Type. Argentina. Salta: Umgebung des Nevado del Castillo, 3050−4570 m, 19/23 Mar 1873, *P.G. Lorentz & G. Hieronymus 96* (lectotype: GOET s.n.!, designated by Funk (1997a: 240); isolectotypes: CORD-00005638 (digital image!), K s.n.!).
Werneria castillensis Hieron., *nom. nud. in sched.* (Turland et al. 2018, ICN Art. 38.1).

#### Description.

**Figure 22.**
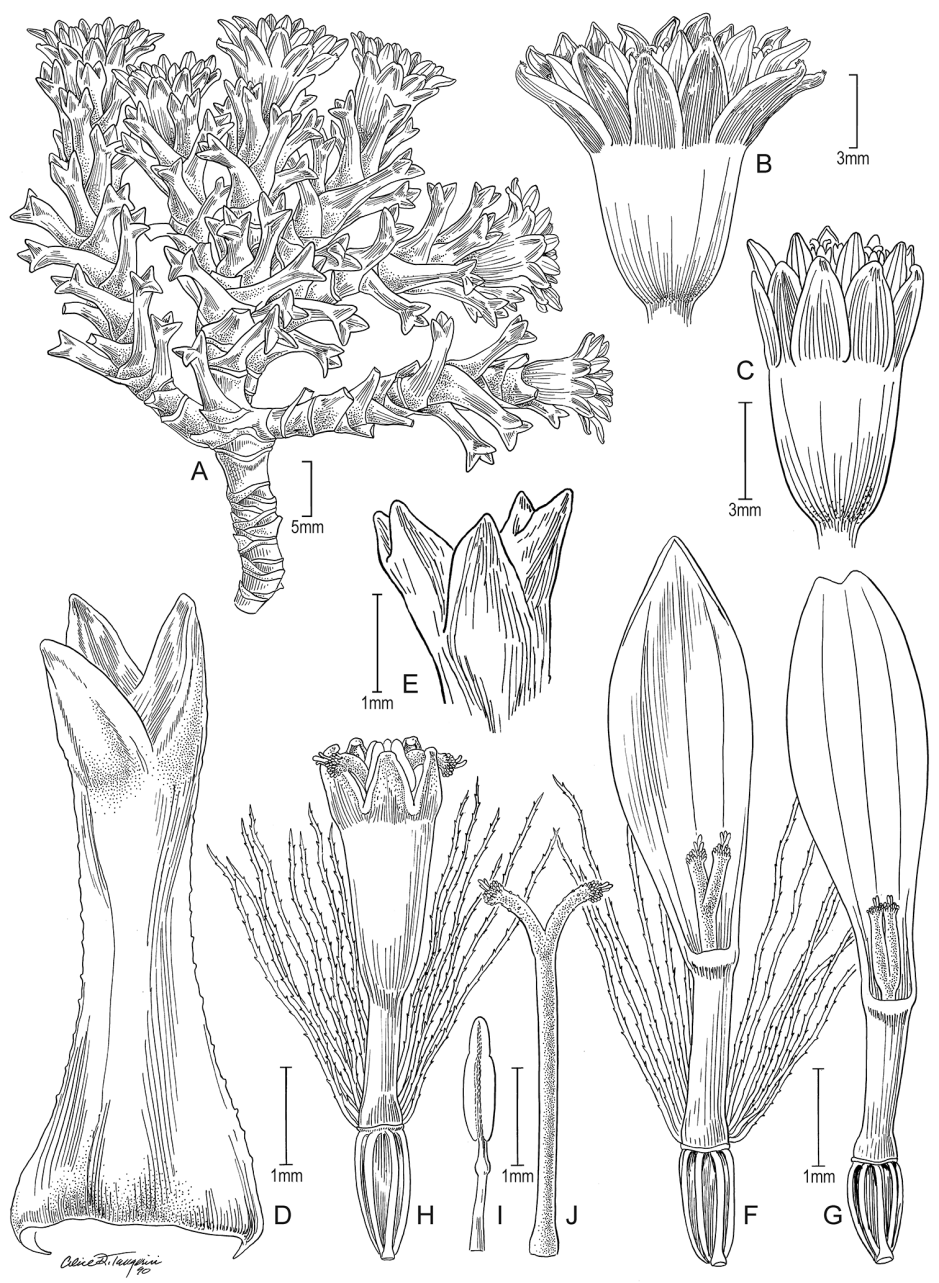
*Xenophyllum pseudodigitatum***A** habit **B, C** capitula at different stages of development **D** leaf **E** leaf apex variability **F** ray floret (frontward bristles removed) **G** ray floret (pappus removed) **H** disc floret (frontward bristles removed) **I** stamen **J** style. All details drawn from *Ellenberg 4295* (US) except for **B, C, F** (drawn from *Petersen & Hjerting 159*, C). Illustration by Alice Tangerini.

Suffruticose plant, forming clumps of erect stems. ***Rhizomes*** 5–8 × 0.4–0.6 cm, horizontal to oblique, glabrous. ***Stems*** 5–12 cm tall, simple or branched, glabrous, usually with leaves restricted to the upper part. ***Leaves*** imbricate, extending into a glabrous sheath-like base; leaf laminas 6–9.6 × 2–3.8 mm, linear-spatulate (broadened upwards), 3-forked at the apex (incision depth 1/8–1/7 of the total length), entire, sometimes with some minute cilia in the lower half, flat to elliptic or almost terete upwards in cross section, glabrous, 1-nerved above (barely visible), 1-nerved beneath, fleshy, matte; leaf lobes 1.3–2.6 × 1–1.6 mm, acute to mucronate (rarely notched). ***Capitula*** radiate, erect, sessile. ***Involucres*** 7–8.4 × 5.2–8 mm, cupuliform; involucral bracts 10 to 11, 3.4–3.9 × 2–2.8 mm, obtuse at the apex, greenish. ***Ray florets*** 20 to 25; corollas 7.1–8.2 × 1–1.2 mm, 3 to 4-veined, subentire to 3-toothed at the apex, conspicuously surpassing the involucre, white. ***Disc florets*** 29 to 33; corollas 4.2–5.1 mm long, yellowish; style branches penicillate, purplish. ***Achenes*** 2.3–2.5 × 0.5–0.7 mm, cylindrical, 7 to 9-ribbed, glabrous; pappus 5.2–7.1 mm long, barbellate, whitish. Chromosome number unknown. Fig. 22.

#### Additional iconography.

Cabrera (1978: 473, fig. 200A–F, sub *Werneria pseudodigitata*); Funk (1997a: 237, fig. 1C); Freire and Ariza-Espinar (2014: 228, *X. pseudodigitatum* A–E).

#### Distribution and habitat.

**Figure 23.**
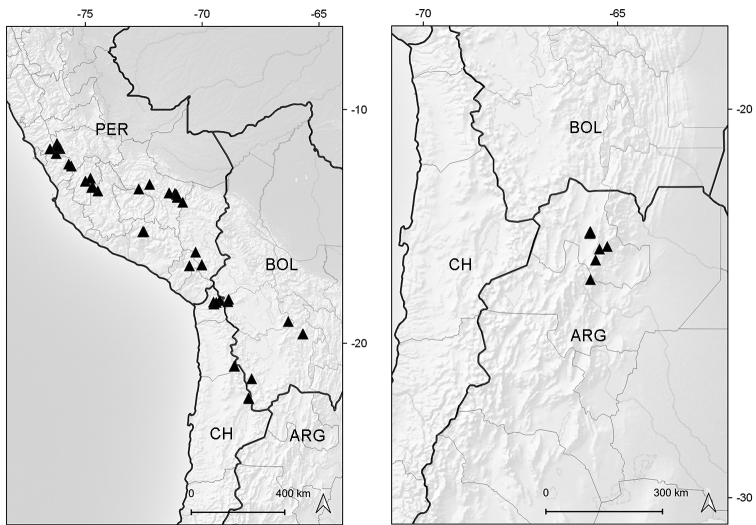
Distribution map of *Xenophyllum digitatum* (left hand) and *X. pseudodigitatum* (right hand).

Endemic to Argentina (Jujuy, Salta). It grows in rocky slopes on rather wet soils of the dry puna ecoregion, between elevations of 3700–5125 m (Fig. 23).

#### Phenology.

Flowering from January to May.

#### Etymology.

The epithet *pseudodigitatum* refers to the resemblance between this species and *X. digitatum*. It probably responds to the fact that both species have 3-forked leaves.

#### Notes.

*Xenophyllum pseudodigitatum* is characterized by its 3-forked leaves. The leaf lobes are 1.3–2.6 mm long, which represents 1/8–1/7 of the total leaf length, and they are acute to mucronate at the apex (rarely notched); on living plants, they are noticeably spread out. The involucre length ranges from 7 to 8.4 mm and has 10 to 11 involucral bracts. The radiate capitula are composed of 20 to 25 ray florets and 29 to 33 disc florets.

This is a narrow endemic species to northwestern Argentina that has erroneously been reported from northern Chile (Ricardi and Marticorena 1966; Freire and Ariza-Espinar 2014; Moreira-Muñoz et al. 2016) and southern Peru (Gonzáles et al. 2016). All these records are confusions with *X. digitatum*. In order to avoid further misidentifications, the following characters should be studied: leaf lobe length (1.3–2.6 mm in *X. pseudodigitatum* vs. 3.6–5.9 mm in *X. digitatum*), involucre length (7–8.4 mm in *X. pseudodigitatum* vs. 12.1–16.7 mm in *X. digitatum*), number of involucral bracts (10 to 11 in *X. pseudodigitatum* vs. 12 to 13 in *X. digitatum*), ray corolla length (7.1–8.2 mm in *X. pseudodigitatum* vs. 9.2–14.1 mm in *X. digitatum*), and number of disc florets (29 to 33 in *X. pseudodigitatum* vs. 49 to 63 in *X. digitatum*). Their distribution areas do not overlap.

#### Additional specimens examined.

**Argentina. Jujuy**: Humahuaca, mina Aguilar, 23°12'S, 65°43'W, 11 Jan 1968, *A.L. Cabrera et al. 19009* (SI); mina Aguilar, abra Aguilar, 23°9'S, 65°42'W, 19 Feb 1969, *A.L. Cabrera, J. Crisci & R. Kiesling 19843* (C); Volcán, abra del Córdoba, 23°53'S, 65°34'W, Feb 1922, *L. Castillón 1761* (LIL); Estación Volcán, 23°53'S, 65°34'W, Feb 1920, *L. Castillón 7140* (A, F); El Aguilar, oberhalb, 5° E. Hochandine Polsterflur, 23°11'S, 65°42'W, 1 Feb 1971, *H. Ellenberg 4295* (US); Tilcara, subida a la abra de Remate, 23°32'S, 65°16'W, 15 Feb 1953, *A. Fiebrig s.n.* (LIL); near mina La Esperanza, just before entrance to town of El Aguilar, side rd. to mina La Esperanza, 23°10'S, 65°43'W, 9 Mar 1993, *V.A. Funk & L. Katinas 11157* (US); rd. to mina La Esperanza, 23°10'S, 65°43'W, 9 Mar 1993, *V.A. Funk & L. Katinas 11169* (US); Humahuaca, mina Aguilares, arriba de la mina, 23°11'S, 65°43'W, 29 Mar 1952, *E. Petersen & J.P. Hjerting 159* (C, LIL); Humahuaca, subida al cerro Aguilar, 23°11'S, 65°43'W, 17 Jan 1953, *H. Sleumer 3448* (LIL); Humahuaca, cerro Aguilar, cumbre, 23°11'S, 65°43'W, 17 Jan 1953, *H. Sleumer 3452* (LIL); Tilcara, cumbre del cerro, 23°36'S, 65°28'W, 3 May 1927, *S. Venturi 6254* (US).

### 18. Xenophyllum decorum

Taxon classificationPlantaeAsteralesAsteraceae

(S.F.Blake) V.A.Funk, Novon 7(3): 239. 1997.

E5CC9369-44E9-51D3-B2CA-7A2AC932AE42

Werneria decora S.F.Blake, J. Washington Acad. Sci. 18: 491. 1928. Type. Peru. Lima: Casapalca, 4725 m, 21 May 1922, *J.F. Macbride & W. Featherstone 849* (holotype: F-517377!; isotypes: G-00237250 (fragment, digital image!), GH s.n.!, S-R-6523 (digital image!), US-00037303!).

#### Description.

Suffruticose plant, forming clumps of erect stems. ***Rhizomes*** 4–10 × 0.4–0.5 cm, horizontal to oblique, glabrous. ***Stems*** 11–19 cm tall, usually branched, glabrous, usually with leaves restricted to the upper part. ***Leaves*** imbricate, extending into a glabrous sheath-like base; leaf laminas 7.2–16 × 2.2–3.7 mm, linear-oblong, slightly broadened towards the apex, 3-notched at the apex, with the lateral lobes wider than central one, entire and shortly ciliate (cilia ca. 0.3 mm long), flat to slightly curved forwards in cross section, glabrous, 1-nerved above (barely visible), 1-nerved beneath, somewhat fleshy, matte; leaf lobes 0.6–1.5 × 1–1.6 mm (central one significantly smaller), rounded to subtruncate. ***Capitula*** radiate, erect, sessile. ***Involucres*** 10–12.6 × 8–10 mm, cupuliform; involucral bracts 13 to 14, 5.2–8 × 2–2.9 mm, acute to obtuse at the apex, greenish. ***Ray florets*** 20 to 21; corollas 10.4–11.9 × ca. 1.5 mm, 3-veined, subentire to 2-toothed at the apex, conspicuously surpassing the involucre, white. ***Disc florets*** 37 to 58; corollas 6–7 mm long, yellow; style branches truncate with a crown of sweeping trichomes or slightly penicillate, purplish. ***Achenes*** 4.8–5.4 × 1–1.2 mm, cylindrical, 7 to 8-ribbed, glabrous; pappus 8.7–9.8 mm long, barbellate, whitish. Chromosome number unknown.

#### Iconography.

Blake (1928: 496, fig. 1D, E sub *Werneria decora*); Beltrán (2016: 359, fig. 3C, as photo).

#### Distribution and habitat.

**Figure 24.**
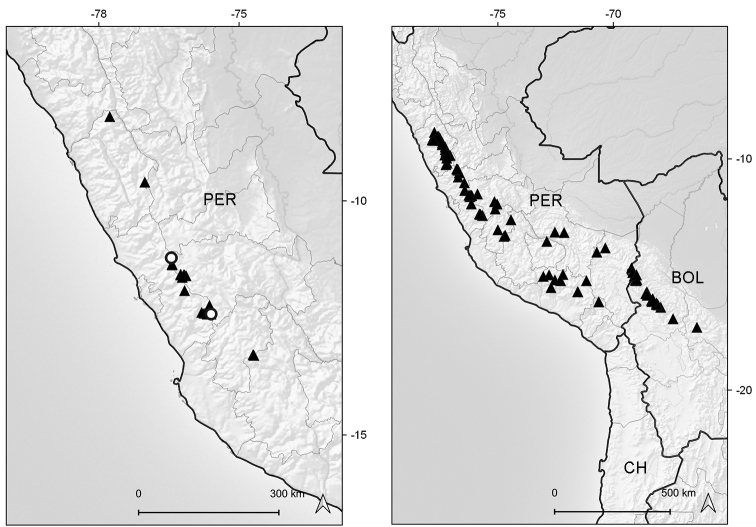
Distribution map of *Xenophyllum decorum* (left hand, closed triangle), *X. amblydactylum* (left hand, open circle), and *X. dactylophyllum* (right hand).

Endemic to Peru (Ancash, Ayacucho, Huancavelica, Huánuco [expected], Junín, Lima). It grows in rocky outcrops, scree slopes, and on cryoturbated soils of the subhumid and humid puna ecoregions, between elevations of 3850–5000 m (Fig. 24).

#### Phenology.

Flowering from February to September.

#### Etymology.

The adjective *decorus -a -um* means handsome, elegant, decorous. Indeed, Blake (1928) pointed out in the protologue that it is “an attractive and very distinct species”.

#### Notes.

This is a distinctive species due to the shortly ciliate leaf margin and the 3-notched leaf apex (the lateral lobes being conspicuously larger than the central one). The leaf lamina is glabrous, linear-oblong, slightly broadened towards the apex, flat in cross section but tending to be curved forwards. It has 13 to 14 involucral bracts, 20 to 21 ray florets with white corollas, 37 to 58 disc florets, and glabrous achenes.

*Xenophyllum decorum* has the leaf apex somewhat similar to that of *X. incisum*, however, their distribution areas are geographically very distant. In addition, these species differ in the leaf length (7.2–16 mm in *X. decorum* vs. 3.2–7.3 in *X. incisum*), leaf margin (shortly ciliate in *X. decorum* vs. not ciliate in *X. incisum*), number of involucral bracts (13 to 14 in *X. decorum* vs. 8 to 9 in *X. incisum*), number of ray florets (20 to 21 in *X. decorum* vs. 8 to 13 in *X. incisum*), and length of ray corollas (10.4–11.9 mm in *X. decorum* vs. 6.2–8.9 mm in *X. incisum*).

It has to be noted that we had problems in identifying *Beltrán 7987* (USM). This specimen exhibits the characteristic flat and ciliate leaves of *X. decorum* but has the leaf apex strongly divided (ca. 2.5 mm deep) with each lobe entire or divided once again. It might be treated as an intermediate form between *X. decorum* and *X. dactylophyllum* or *X. digitatum* since these species co-occur in the region where Beltrán’s specimen was collected.

#### Additional specimens examined.

**Peru. Ancash**: Huari, San Marcos, altura del km 111–112 de la carretera al campamento minero Antamina, 9°36'S, 77°1'W, 26 Mar 2004, *A. Cano et al. 14168* (USM); Pallasca, Pampas, cordillera Pelagatos, 8°12'S, 77°46'W, 18 Apr 2011, *A. Cano et al. 20203* (USM); collado encima río Pumapampa, 18 Mar 1983, *O. Tovar et al. 9710* (USM); **Ayacucho**: Cangayllo-Huaytará, Paras-Pilpichaca, alrededor del abra Apacheta, 13°17'S, 74°42'W, 23 Aug 2014, *P. Gonzáles et al. 3329* (USM); **Huancavelica**: Huaytará, alrededores de abra Apacheta, 13°18'S, 74°41'W, 3 Sep 2013, *P. Gonzáles et al. 2783* (USM); **Junín**: Anticona pass, ca. 140 km E of Lima on Hwy. to La Oroya, 11°35'S, 76°11'W, 16 Dec 1978, *M. Dillon & B.L. Turner 1308* (USM); Lima-La Oroya highway, cumbre at top of divide, above Morococha, 11°36'S, 76°8'W, 29 Jun 1982, *A. Gentry & R. Tredwell 37301* (USM); Ticlio, 11°35'S, 76°11'W, 11 Jul 1982, *B. Maass 575* (USM); Yauli, Anticona arriba, 11°35'S, 76°10'W, 24 Mar 1979, *O. Tovar, S. Rivas & Sáenz 7806* (USM); Anticona, abajo entre Casapalca y Oroya, 11°35'S, 76°11'W, 26 Jun 1954, *O. Tovar & L. Constance 9194* (USM-29378 [mixed with *X. ciliolatum* and *digitatum*]); **Lima**: Huarochirí, paso de Anticona, 11°35'S, 76°11'W, 1 May 1999, *H. Beltrán & E. Li 3306* (USM); Yauyos, Laraos, pampas de Quiray cerca a la laguna de Huinso, 12°23'S, 75°48'W, 4 Feb 2000, *H. Beltrán 3418* (USM); Yauyos, Laraos, estancia de Quiray, 6 horas de camino al pueblo, 12°24'S, 75°45'W, 11 May 2001, *H. Beltrán 4059* (USM); Yauyos, Laraos, Viscollo, 12°25'S, 75°36'W, 12 May 2001, *H. Beltrán 4188* (USM); Huarochirí, desvío de carretera central hacia Chinchan y Marcapomacocha, 11°34'S, 76°15'W, 23 Sep 2014, *H. Beltrán & W. Aparco 7740* (USM); Huarochirí, Chicla, abra Anticona (Ticlio), 11°35'S, 76°11'W, 29 Apr 2017, *H. Beltrán, S. Castillo & M. Arakaki 7988* (USM); Yauyos, Tomas, abra Chaucha, 12°14'S, 75°38'W, 10 Aug 2017, *H. Beltrán 8469* (USM); Huarochirí, Ticlio, 11°35'S, 76°11'W, 8 Aug 1982, *K. Biegman s.n.* (USM); Yauyos, Laraos, camino a Mina, 12°25'S, 75°41'W, 14 Apr 2012, *A. Cano & H. Trinidad 20646* (USM); Huarochirí, San Damián, Chanape, 11°55'S, 76°10'W, 7 Jul 2013, *P. Gonzáles & B. Brito 2655* (USM); Huarochirí, Ticlio, canyon of the río Rimac, on the carretera central, 11°35'S, 76°11'W, 19 Aug 1957, *P.C. Hutchison 1212* (CONC, F, GH, LE, NY, UC, US [all specimens mixed with *X. digitatum*]); Anticona, 11°35'S, 76°15'W, 5 Aug 2012, *E. Linares & A. Galán 3101* (USM); Canta, La Viuda (km 165 carretera Lima-cerro de Pasco), 11°21'S, 76°26'W, 7 Aug 1964, *I. Meza 227* (USM); Canta, Cullhuay, laderas de la laguna de Chuchún, 11°22'S, 76°26'W, 17 Aug 1996, *G. Vilcapoma 4461* (USM).

### 19. Xenophyllum amblydactylum

Taxon classificationPlantaeAsteralesAsteraceae

(S.F.Blake) V.A.Funk, Novon 7(3): 238. 1997.

109407CF-7FAE-5C4B-BE01-5B7EC3095385

Werneria amblydactyla S.F.Blake, J. Washington Acad. Sci. 18: 490. 1928. Type. Peru. Junín: Andes, Peru, Alpamarca, [without date], *Capt. Wilkes Expedition s.n.* (holotype: US-00037297!; isotype: GH s.n.!).

#### Description.

Suffruticose plant, forming clumps of erect stems. ***Rhizomes*** 10–12 × ca. 0.5 cm, horizontal to oblique, glabrous. ***Stems*** 4–7 cm tall, rather branched, glabrous, usually with leaves restricted to the upper part. ***Leaves*** subimbricate, extending into a sheath-like base that bears evanescent arachnoid trichomes; leaf laminas 4.3–10 × 1.3–1.4 mm, linear, barely broadened at the apex, 3-notched at the apex, entire, usually with some minute cilia near the base, flat to obtusely triangular in cross section, with evanescent arachnoid trichomes above, 1-nerved above (barely visible), 1-nerved beneath, somewhat fleshy, matte; leaf lobes 0.8–1.4 × 0.6–1 mm, obtuse. ***Capitula*** radiate, erect, sessile. ***Involucres*** 8.8–9.8 × 5.5–7 mm, cupuliform; involucral bracts 10 to 13, 4.9–5.4 × 1.2–1.8 mm, acute at the apex, greenish. ***Ray florets*** ca. 11; corollas 9–10.2 × ca. 1.2 mm, 4-veined, subentire to 2-toothed at the apex, conspicuously surpassing the involucre, white. ***Disc florets*** ca. 22; corollas 5.4–6 mm long, yellowish; style branches truncate with a crown of sweeping trichomes or slightly penicillate, yellowish. ***Achenes*** cylindrical, glabrous (immature); pappus ca. 5.6 mm long, barbellate, whitish. Chromosome number unknown.

#### Iconography.

Blake (1928: 496, fig. 1B, C sub *Werneria amblydactyla*).

#### Distribution and habitat.

Endemic to Peru (Junín, Lima). It grows in rocky outcrops of the puna ecoregion, at elevations of ca. 4600 m (Fig. 24).

#### Phenology.

Collected in flower in May.

#### Etymology.

The epithet *amblydactylum* means having blunt fingers (“ambly-”: blunt, obtuse; “dactyl-”: finger). It refers to the leaf apex lobes of this species.

#### Notes.

*Xenophyllum amblydactylum* forms clumps of erect stems 4–7 cm tall that bear linear, 4.3–10 mm long leaves. The leaf apex is 3-notched and the lobes are small (0.8–1.4 × 0.6–1 mm) and connivent. The leaf lamina has arachnoid trichomes on the adaxial surface that are quicky evanescent. The involucre is 8.8–9.8 mm long and it is composed of 10 to 13 involucral bracts. The capitula are radiate and display ca. 11 ray florets with white corollas.

This is a poorly known and collected species. Aside from the type material, we only studied one collection from southeastern Lima Department (*Beltrán 4174*, USM) and a specimen that was bought as medicinal in a farmers market in the city of Lima (*Yarupaitán 1499b*, USM). Further collections are required in order to improve the understanding of its variability and properly describe the achenes.

It might be confused with *X. dactylophyllum* because their distribution areas overlap. However, several characters let anyone discriminate them from one another, i.e., leaf apex (3-notched in *X. amblydactylum* vs. at least 9-divided in *X. dactylophyllum*), leaf lobe length (0.8–1.4 mm in *X. amblydactylum* vs. primary division 2–3 mm in *X. dactylophyllum*), involucre length (8.8–9.8 mm in *X. amblydactylum* vs. 10.8–12.8 mm in *X. dactylophyllum*), ray floret number (ca. 11 in *X. amblydactylum* vs. 17 to 22 in *X. dactylophyllum*).

#### Additional specimens examined.

**Peru. Lima**: Yauyos, Laraos, Carhuanisho, 12°25'S, 75°36'W, 12 May 2001, *H. Beltrán 4174* (USM); [without locality, bought as medicinal in the farmers market in ave. Aviación, La Victoria, Lima, 29 Oct 1994], *G. Yarupaitán 1499b* (USM).

### 20. Xenophyllum dactylophyllum

Taxon classificationPlantaeAsteralesAsteraceae

(Sch.Bip.) V.A.Funk, Novon 7(3): 239. 1997.

0E41AA54-F397-5DFB-BD0E-33F74548877D

Werneria dactylophylla Sch.Bip., Bonplandia (Hannover) 4: 53, 55. 1856. Type. Peru. Puno: pr. Agapata [Ayapata] ad nives eternas, 4875 m, Jun 1854, *W. Lechler 1807* (lectotype: G-00305680 (digital image!), designated by Funk (1997a: 239); isolectotypes: BR s.n.!, GH s.n.!, GOET s.n.!, K-000527745 (digital image!), LE s.n.!, LL-00374330 (digital image!), NY s.n.!, P-02088571 (digital image!), P-02088572 (digital image!), P-02088573 (digital image!), P-02088575 (digital image!), S-R-6522 (digital image!), W s.n.!).
Werneria dactylophylla f. glabriuscula Rockh., Bot. Jahrb. Syst. 70: 286. 1939. Type. Bolivia. San Francisco-Tal, Ancohuma, 5000 m, 3 Jul 1928, *C. Troll 2076* (lectotype: M-0147065 (digital image!), designated here; isolectotype: B s.n.!, CONC-80182!).
Xenophyllum oscartovarii E.Linares, J.Campos, Nauray, Vicente Orell. & A.Galán, Arnaldoa 17(1): 104. 2010 [“oscartovari”]. Type. Peru. Arequipa: Caylloma, sector Derivación, 19L 0179145 8636313 [erroneous, ca. 15°00'S, 72°11'W], 4740 m, 4 Feb 2009, *E. Linares & A. Galán 2354* (holotype: USP n.v.; isotype: AQP n.v.), syn. nov.

#### Description.

**Figure 25.**
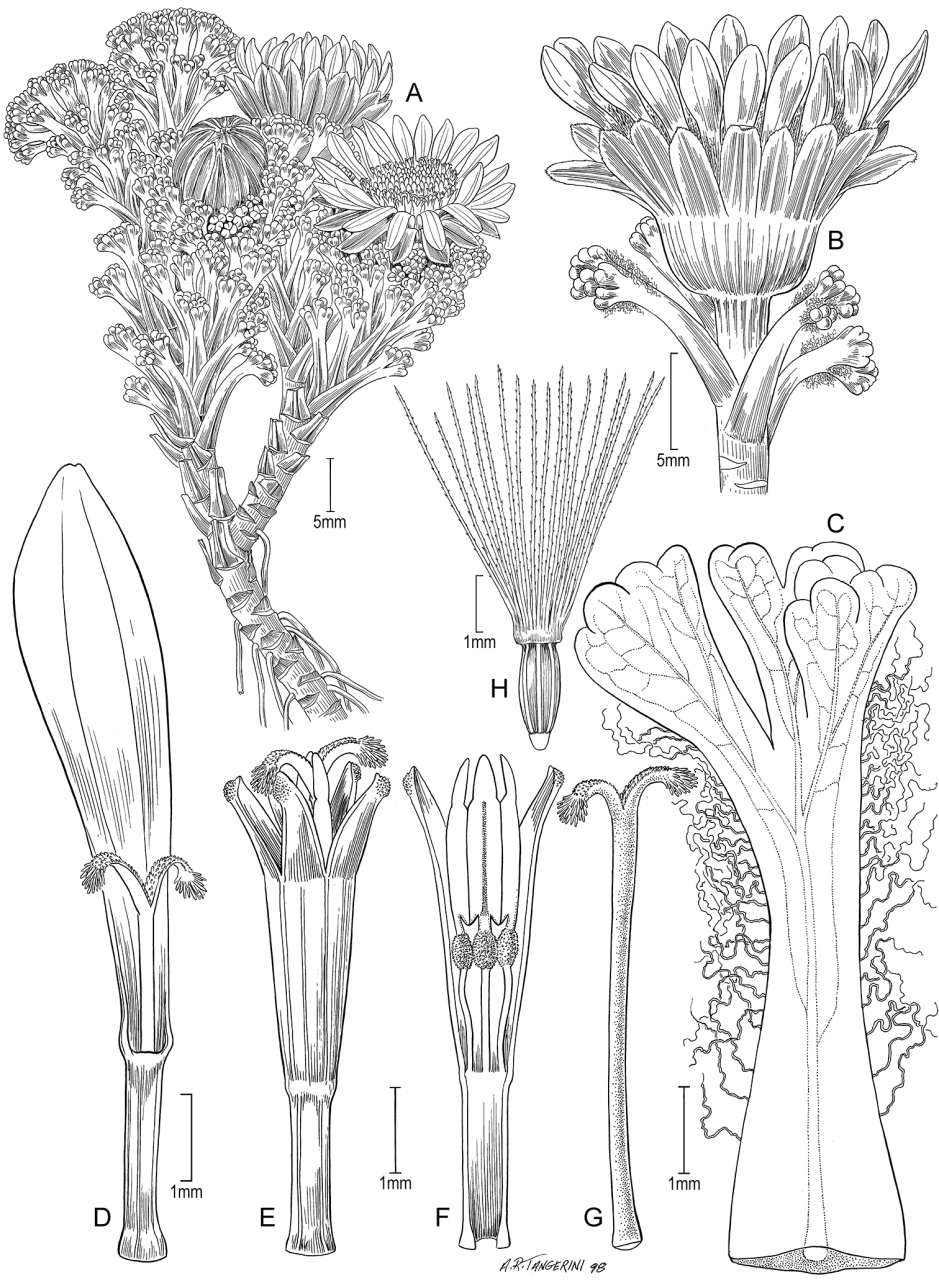
*Xenophyllum dactylophyllum***A** habit **B** stem apical part and capitulum **C** leaf **D** ray corolla and style **E** disc corolla and style branches **F** disc corolla and stamens (vertically sectioned, style removed) **G** style **H** achene with pappus. All details drawn from *Funk & González-Quint 11372* (US). Illustration by Alice Tangerini.

Suffruticose plant, forming clumps of erect stems or cushions. ***Rhizomes*** 5–15 × 0.5–0.8 cm, horizontal to oblique, glabrous. ***Stems*** 11–24 cm tall, branched, glabrous, usually with leaves restricted to the upper part. ***Leaves*** imbricate, extending into a sheath-like base glabrous or with long silky trichomes; leaf laminas 5.7–9.9 × 2.4–4.1 mm, spatulate (finger-like at the apex), at least 9-divided at the apex, with the primary division extending deeper than the subsequent ones, entire, sometimes with some minute cilia, elliptical to terete upwards in cross section, glabrous or floccose-lanate, 1-nerved above (barely visible), 1-nerved beneath, somewhat fleshy, matte; primary division 2–3 mm long, 3 to 6 divided, rounded to truncate, sometimes with some scattered cilia on the margin. ***Capitula*** radiate, erect, sessile. ***Involucres*** 10.8–12.8 × 8.2–9.5 mm, cupuliform; involucral bracts 11 to 20, 6.6–7.6 × 1.9–2 mm, obtuse at the apex, greenish, sometimes purple-edged. ***Ray florets*** 17 to 22; corollas 9.2–11 × 1.3–1.6 mm, 4-veined, subentire to 3-toothed at the apex, conspicuously surpassing the involucre, white. ***Disc florets*** 37 to 50; corollas 5.3–6.3 mm long, yellowish; style branches truncate with a crown of sweeping trichomes or slightly penicillate, purplish. ***Achenes*** 3.1–4.8 × 0.9–1.3 mm, cylindrical, 7 to 9-ribbed, glabrous; pappus 8.1–8.9 mm long, barbellate, whitish. Chromosome number 2*n* = 108(±4) (Diers 1961). Fig. 25.

#### Additional iconography.

Linares et al. (2010: 102, fig. 1, sub *X. oscartovarii*, as photo); Beltrán (2016: 359, fig. 3A, as photo).

#### Distribution and habitat.

Central Peru to central Bolivia. Bolivia (Cochabamba, La Paz, Potosí [n.v.]), Peru (Ancash, Apurímac, Arequipa, Ayacucho, Cusco, Huancavelica, Huánuco, Junín, Lima, Moquegua, Pasco, Puno). This species grows in rocky outcrops, scree slopes, cryoturbated soils, and exposed grasslands of the subhumid and humid puna ecoregions, between elevations of (2800–)3400–5500 m (Fig. 24).

*Xenophyllum dactylophyllum* has been reported from the Bolivian department of Potosí on the basis of the collection *Steinbach s.n.* (Jørgensen 2014; Tropicos 2020). This material, which has not been located, was collected nearby Andacaba, a locality ca. 30 km south of the city of Potosí. If the identification is confirmed, it would represent the southern distribution limit of this species. The specimen US-00974139 most likely corresponds to a mislabeling because both the locality (San Carlos de Bariloche, Río Negro Province, Argentina) and the elevation (600 m) do not correspond to the known distribution area and altitudinal rank of this species.

#### Phenology.

Flowering nearly all year round.

#### Etymology.

The epithet *dactylophyllum* means finger-like leaves and it refers to the resemblance between the leaves of this plant and a hand.

#### Notes.

*Xenophyllum dactylophyllum* is characterized by having glabrous or floccose-lanate finger-like leaves, with at least 9 divisions at the apex. The divisions are usually arranged in multiples of three, the primary one extending deeper than the subsequent ones. The capitula are radiate, erect, with 17 to 22 ray florets with white corollas conspicuously surpassing the involucre.

Another species with finger-like leaves is *X. staffordiae*, which has occasionally been confused with *X. dactylophyllum*. They can be readily differentiated by the habit (11–24 cm tall in *X. dactylophyllum* vs. 30–60 cm tall in *X. staffordiae*), leaf divisions (at least 9-divided, with the primary division extending deeper than the subsequent ones in *X. dactylophyllum* vs. at least 12-divided, with all divisions similar in length in *X. staffordiae*), capitulum position (erect in *X. dactylophyllum* vs. rather nodding in *X. staffordiae*), and length and color of the ray corollas (9.2–11 mm, surpassing the involucre, white in *X. dactylophyllum* vs. 6.4–8.3 mm, not surpassing the involucre, pale yellow in *X. staffordiae*). Their distribution areas overlap from central to southern Peru. Other morphologically similar species are *X. amblydactylum* and *X. lorochaqui* (see comments under these species).

Our efforts in locating the type material of *X. oscartovarii* E.Linares, J.Campos, Nauray, Vicente Orell. & A.Galán were unfruitful. Due to the original description and the pictures included in the publication (Linares et al. 2010), we believe that it fairly corresponds to *X. dactylophyllum*. Oddly, this latter species was not mentioned in the taxonomic discussion of the presumed new species. In addition, we studied several specimens of *X. dactylophyllum* collected close to the type locality of *X. oscartovarii*.

#### Additional specimens examined.

**Bolivia. Cochabamba**: im Geröll des Cerro Tunari, 17°17'S, 66°23'W, May 1911, *T. Herzog 2094* (S n.v.; locality confirmed by M. Zárate *in litt.*); **La Paz**: Murillo, Huayna Potosí, 29 Apr 1977, *S.G. Beck 1* (LPB); Bautista Saavedra, 1 km antes de la cumbre, donde hay desvíos por Charazani, Ulla Ulla y Amarete, 3 Aug 1985, *S.G. Beck 11347* (LPB); Inquisivi, cordillera Tres Cruces, al pie de glaciar Atoroma, 16°55'S, 67°25'W, 23 Dec 1978, *S.G. Beck 183* (LPB); Murillo, Milluni 11 km hacia Tuni, pie del glacial Huayna Potosí y María Lloco, 5 Jan 1980, *S.G. Beck 2425* (LPB); Murillo, subiendo el valle Khallapa (hacia Hampaturi), entrando al valle lateral del río Palcoma y subiendo hacia la serranía Murillo, 16°25'S, 67°57'W, 6 May 2012, *S.G. Beck 33940* (LPB); Murillo, fin del valle Kaluyo pasando el pueblo de Chacaltaya, Pampalarama, 150 m por debajo del glaciar, 16°19'S, 68°4'W, 14 Mar 2013, *S.G. Beck, D. Ibáñez & C. Beck 34084* (LPB); Larecaja, cumbre, arriba del abra hacia mina Fabulosa, 16°3'S, 68°18'W, 10 Mar 2016, *S.G. Beck 35111* (LPB); La Fabulosa, 16°3'S, 68°18'W, 26 Apr 1950, *W.M. Brooke 6322* (CONC); Murillo, en las faldas del cerro Huayna Potosí, camino a Tuni Condoriri, 28 Jun 1991, *V. Camacho & M. Rendon 1* (LPB); Loayza, sierra de Tres Cruces, 22 Feb 1979, *A. Ceballos et al. 472* (COL); Bautista Saavedra, camino entre Escoma y Charazani, zona del ANMI Apolobamba, 15°15'S, 69°2'W, 4 Oct 2005, *N. de la Barra et al. 1026* (BOLV, LPB); Humasani, 15 Jun 1974, *Essel 23* (LPB); Bautista Saavedra, Pumazani, am Beginn der Strabe nach Amarete, 15°13'S, 68°59'W, 25 May 1980, *T. Feuerer 4228* (LPB); Murillo, Zongo-Tal, am Lago Zongo, 16°17'S, 68°7'W, 1 Jun 1980, *T. Feuerer 4335a* (LPB); Los Andes, an der Strabe zur Mine Fabulosa, 10 Feb 1980, *T. Feuerer 8472* (LPB); valle de Zongo, 1.5 km avant la cumbre, 16°16'S, 68°7'W, 24 Apr 1988, *A. Fournet 815* (BOLV); Bautista Saavedra, Amarka, cerca del límite occidental del ANMI Apolobamba, 15°15'S, 69°2'W, 4 Sep 2004, *A. Fuentes & C. Aldana 6721* (BOLV, LPB); Murillo, nev. Huayna Potosí, E slopes above rd., 16°17'S, 68°8'W, 12 Apr 1995, *V.A. Funk & N. Bernal 11282* (LPB); Murillo, nev. Charquini, above aqueduct on N facing slope, 16°17'S, 68°6'W, 12 Apr 1995, *V.A. Funk & N. Bernal 11287* (LPB); Los Andes, Hichu-Kkota valley, 20 km from base of lgn. Khara Kkota along rd. to mina Fabulosa, 16°10'S, 68°20'W, 25 Apr 1995, *V.A. Funk & C. González-Quint 11371* (LPB, US); Los Andes, Hichu-Kkota valley, 21 km from base of lgn. Khara Kkota along rd. to mina Fabulosa, near the pass, 16°10'S, 68°20'W, 25 Apr 1995, *V.A. Funk & C. González-Quint 11372* (LPB, US); Los Andes, above cumbre (pass) on rd. through Hichu-Kkota valley on rd. to mina La Fabulosa, 21 km from base of lag. Khara Kkota, 16°10'S, 68°20'W, 29 Apr 1995, *V.A. Funk 11408* (LPB); Bautista Saavedra, Charazani, pampa de Ulla-Ulla, cerca de la estancia Medallani, 15°4'S, 69°6'W, 4 May 1993, *P. Gutte & B. Herzog 702* (LPB); Franz Tamayo, Ulla Ulla, cordillera Apolobamba cerca al pueblo de Pelechuco 17 km NE, 14°55'S, 69°10'W, 22 Aug 1986, *P. Holt 15A* (LPB); José M. Camacho, 60 km al E de Ulla Ulla, arriba del paso Osipal, 29 Jul 1993, *P. Holt 2* (LPB); José M. Camacho, 60 km al E de Ulla Ulla, arriba de Osipal, 29 Jul 1993, *P. Holt 3* (LPB); Murillo, cerro Charquini, a 1h 20min del centro de La Paz, 16°17'S, 68°6'W, Jun 2015, *A. Huon & R.I. Meneses 1* (LPB); Murillo, Huayna Potosí, 29 Apr 1977, *E. Jordan 1* (LPB); Bautista Saavedra, división camino Amarete-Charazani-Ulla Ulla, 15°14'S, 69°3'W, 14 Jun 1997, *M. Kessler et al. 10144* (LPB); Murillo, Zongo pass, slope up towards Huayna Potosí, 16°14'S, 68°8'W, 6 Aug 1991, *M. Kessler 2840* (LPB); Humasani, 17 Jun 1975, *R. Lara 1499* (LPB); Larecaja, viciniis Sorata, prope Anilaya, ad lacum Yuriguana, 15°45'S, 68°33'W, Mar/May 1858, *G. Mandon 101* (LE, RB, W); Bautista Saavedra, abra Pumasani, 15°15'S, 69°2'W, 20 Apr 1982, *X. Menhofer 1108* (LPB); Murillo, nevado Huayna Potosí, 14 Jul 1982, *X. Menhofer 1420* (LPB); Franz Tamayo, lado E de la cordillera de Apolobamba, camino de Pelechuco a Sorata, cerca de la mina abandonada de Sunchuli, 15°1'S, 69°0'W, 25 May 1984, *X. Menhofer 2344* (LPB); Franz Tamayo, lado E de la cordillera de Apolobamba, camino de Pelechuco a Sorata, cerca de la mina abandonada de Sunchuli, 15°1'S, 69°0'W, 25 May 1984, *X. Menhofer 2345* (LPB); Omasuyos, Hichu Cota, subiendo a la cumbre, 16°7'S, 68°21'W, 28 Apr 1985, *M. Moraes 150* (LPB); Los Andes, Hichu-Kkota, 26 Jan 1984, *C. Ostria 119* (LPB); Los Andes, valle de Hichu Kkota, 17 Nov 1983, *C. Ostria 48* (LPB); Inquisivi, cordillera Tres Cruces, 13 Sep 2001, *B.J. Ruthsatz 10533* (LPB); Murillo, 4.5 km N of Milluni on road to Zongo pass (ca. 3 km S of pass), 16°18'S, 68°8'W, 7 Feb 1985, *J.C. Solomon 13210* (LPB); Murillo, pass at the head of valle del Zongo, 16°17'S, 68°8'W, 6 Mar 1983, *J.C. Solomon 9785* (LPB); Larecaja, Sorata, laguna glacial nevado Illampu, 15°49'S, 68°33'W, 20 Jul 1996, *J.R.I. Wood 11255* (BOLV, HSB, LPB); Bautista Saavedra, on road between Escoma and Charazani, ca. 3 km before descent to Charazani, 15°16'S, 69°1'W, 10 Jun 2000, *J.R.I. Wood & C. Wendleburger 16400* (LPB); Larecaja, Sorata, zona de la laguna glacial nevado Illampu, 15°50'S, 68°36'W, 7 Apr 2004, *J.R.I. Wood 20663* (LPB); Murillo, cordillera Huayna Potosí, base de la cordillera de Huayna Potosí, 16°16'S, 68°7'W, 27 Mar 2007, *M. Zárate 2440* (BOLV, LPB); Franz Tamayo, Pelechuco, al N en línea recta a 3.8 km del campamento Chocollo, 14°43'S, 69°13'W, 25 Nov 2017, *F. Zenteno, D. Villalba & L. Mamani 21277* (LPB). **Peru. Ancash**: abra Yanashalla, 9°51'S, 77°4'W, 25 May 2013, *C. Aedo & J. Molina 20440* (MA); Huaylas, Macate, cerro al norte de la laguna Capalo, 21 May 2000, *A. Cano, A. Ramírez & C. Cáceres 10549* (USM); Asunción, Chacas, debajo de la punta Olímpica, entre las lagunas, 9°8'S, 77°30'W, 24 Apr 2004, *A. Cano, M.I. La Torre & W. Mendoza 14536* (USM); Asunción, Chacas, alrededores de la laguna Lebrón, 9°12'S, 77°29'W, 20 May 2009, *A. Cano et al. 19378* (USM); Huari, Rajupampa, laderas hacia el nevado Santa Rosa, 9°29'S, 77°17'W, 7 Oct 2012, *A. Cano et al. 21319* (USM); Huaylas, Riurín, alturas de Pueblo Libre y alrededores, cerro Huachoq Qocha y cerro Gallu Huaqanan, 9°12'S, 77°47'W, 19 May 1999, *A. Cano et al. 9252* (USM); Huaylas, Ocshapampa (Oxapampa), 9°11'S, 77°51'W, 11 Oct 1999, *A. Cano et al. 9733* (USM); Bolognesi, Pariac Punta, pampa de Lampas, Chiquián, 10°11'S, 77°12'W, 4 May 1952, *E. Cerrate 1513* (USM); Bolognesi, Ticllos, paso de Chonta, 10°15'S, 77°15'W, 29 Apr 1956, *E. Cerrate 2651* (USM); Bolognesi, Chilcas, laguna de Quitcka, 25 Nov 1981, *E. Cerrate, B. León & J. Campos 8400* (USM); near top of divide over cordillera Blanca, upper slopes of Huascarán, above lagunas Llanganuco, 9°1'S, 77°35'W, 10 Jul 1982, *A. Gentry et al. 37431* (USM); Asunción-Chacas, laguna Librón, 9°12'S, 77°29'W, 13 Aug 2008, *B. Roca Ramos 1* (USM); Asunción-Chacas, laguna Librón, 9°12'S, 77°29'W, 13 Aug 2008, *B. Roca Ramos 2* (USM); Asunción-Chacas, laguna Atlanta, a 2 km de la laguna Atlanta o Azulcocha, 9°3'S, 77°33'W, 12 Aug 2008, *B. Roca Ramos 3* (USM); Huari, camino Olleros a Chavín, desde el abra hasta el lado oriental de la cordillera Blanca, 9°36'S, 77°17'W, 21 Oct 1999, *J. Roque & K. Young 1221* (USM); Recuay, Huascarán N.P., pass between nevado Pasto Ruri and nevado Raria, río Pachacoto drainage, 9°52'S, 77°11'W, 31 Mar 1985, *D.N. Smith & F. Escalona 10207* (USM); Yungay, Huascarán N.P., Llanganuco sector, quebrada Demanda, W of Chacraraju base camp, 9°1'S, 77°36'W, 13 Apr 1985, *D.N. Smith & V. Cautivo 10290* (USM); Recuay, Huascarán N.P., quebrada Quenua Ragra, 9°58'S, 77°13'W, 10 May 1985, *D.N. Smith, R. Valencia & A. Gonzales 10700* (LPB, USM); Recuay, Huascarán N.P., camino de herradura passing over Cahuish tunnel, 9°41'S, 77°15'W, 10 Jul 1985, *D.N. Smith & M. Buddensiek 11126* (USM); Carhuaz, Huascarán N.P., quebrada Ishinca, side valley to laguna Ishinca, 9°23'S, 77°25'W, 16 Jul 1985, *D.N. Smith & M. Buddensiek 11208* (USM); Huari, Huascarán N.P., just crossing the Ulta pass, 9°7'S, 77°30'W, 28 Jul 1985, *D.N. Smith 11304* (USM); Carhuaz, Huascarán N.P., quebrada Honda, from pass towards valley bottom, N-side of valley, 9°18'S, 77°24'W, 3 Oct 1985, *D.N. Smith, M. Buddensiek & R. Valencia 11644* (USM); Recuay, Huascarán N.P., lateral valley of quebrada Queshque, toward río Pachacoto drainage, 9°50'S, 77°18'W, 19 Mar 1986, *D.N. Smith, R. Valencia & M. Torres 11900* (USM); Yungay, Huscarán N.P., between lake Llanganuco and Portachuelo, 9°3'S, 77°36'W, 16 Aug 1984, *D.N. Smith 8258* (USM); Huaylas, Huascarán N.P., pass between quebrada Los Cedros and Hatuncocha, 8°51'S, 77°45'W, 12 Mar 1985, *D.N. Smith & R. Valencia 9963* (USM); Huaylas, sector denominado Huiripunta, 6 Sep 1994, *G. Yarupaitán & E. Salas 1482* (USM); **Apurímac**: Abancay, Ampay, 13°34'S, 72°53'W, Aug 1937, *C. Vargas & Santander 514* (CUZ); **Arequipa**: pr. Chivay, ladera S del nevado Huarancante, 15°45'S, 71°32'W, 1 Apr 2005, *C. Aedo & A. Galán 11037* (MA, USM); Caylloma, pasando la cumbre Chucura, 15°45'S, 71°33'W, 16 Jan 1999, *S.G. Beck 26468* (LPB); Castilla, Orcopampa, minas de Poracota, cerca a quebrada Faculla, 15°14'S, 72°32'W, 20 Apr 2011, *H. Beltrán 7115* (USM); Ramón Castilla, Orcopampa, alrededores de Cia. Minera Ares, 15°16'S, 72°17'W, 31 Mar 2000, *A. Cano & N. Valencia 10106* (USM); La Unión, Huaynacotas, Sarajorepampa, 15°1'S, 72°47'W, 18 Mar 2011, *D. Montesinos 2951* (HSP, MOL); Castilla, Tapay, cerro Blanco, Apacheta, 17 Sep 2011, *N. Vega 1782* (USM); Condesuyos, nevado de Coropuna, 15°34'S, 72°42'W, Jun 1976, *M. Weibel s.n.* (USM); **Ayacucho**: Cangallo, Paras, abra Apacheta, 13°21'S, 74°44'W, 25 Jun 2001, *J. Roque & C. Arana 3289* (USM); Páucar del Sara Sara, Oyolo, a 15 km al NO de Pampamarca, camino a Sayla, 15°5'S, 73°2'W, 14 Sep 2013, *C. Tejada 233* (HSP); **Cusco**: Urubamba, Machupijchu, 13°10'S, 72°32'W, Nov 1982, *B. Peyton 97* (CUZ); Espinar, al oeste del poblado de Condoroma, a 1 km de la carretera, 15°16'S, 71°10'W, 30 Sep 2009, *W. Ramírez 585* (USM); Urubamba, Pumahuanca, 13°11'S, 72°8'W, 8 Jun 1991, *A. Tupayachi 1515* (CUZ); **Huancavelica**: Huaytará, alrededores de abra Apacheta, 13°18'S, 74°41'W, 3 Sep 2013, *P. Gonzáles et al. 2780* (USM); Huachocolpa, alrededores de la unidad minera Caudalosa, 13°4'S, 75°0'W, 23 Mar 2015, *P. Gonzáles 3528* (USM); paso de Chonta, 12°38'S, 74°26'W, 8 May 1958, *O. Tovar 2948* (USM); **Huánuco**: Lauricocha, Cauri, alrededores de las lagunas Tinkicocha, Lauricocha, Tauricocha y Patarcocha, 10°27'S, 76°44'W, 8 Sep 2012, *P. Gonzáles & N. Valencia 1921* (USM); Dos de Mayo, entre Lauricocha y Otuto, [without date], *A. Raimondi 1589* (USM); Lauricocha, San Miguel de Cauri, campamento Raura, laguna artificial antes de ingresar a Niñococha bajo, 10°27'S, 76°45'W, 7 May 2004, *F. Salvador, S. Ríos & E. Arias 735* (USM); Lauricocha, San Miguel de Cauri, a 100 m de puesto de control Santa Rosa (campamento Raura), ladera abajo, 11°35'S, 76°11'W, 13 May 2004, *F. Salvador, S. Ríos & E. Arias 944* (USM); **Junín**: Huancayo, Pucará, Raquina, 12°10'S, 75°7'W, [without date], *D. Barrón 57* (USM); Yauli, paso de Anticona, cumbre más alta de la carretera central, 11°35'S, 76°11'W, 17 Dec 1951, *E. Cerrate 998* (USM); Anticona pass, ca. 140 km E of Lima on Hwy. to La Oroya, 11°35'S, 76°11'W, 16 Dec 1978, *M. Dillon & B.L. Turner 1300* (USM); cercanías de Morococha, 11°35'S, 76°8'W, 13 Nov 1863, *J. Isern 404* (MA); La Oroya, 11°31'S, 75°53'W, 15 Jun 1915, *K. Maisch 150* (USM); Huaytapallana, 11°57'S, 75°2'W, 4 May 1961, *O. Tovar 3386* (USM); Yauli, Ticlio, 11°35'S, 76°11'W, Feb 1974, *O. Tovar 7187* (USM); Yauli, Milloc, camino del desvío de Casapalca, 20 Mar 1983, *M.A. Vargas 77* (USM); Jauja, al pie del nevado de Tullujuto, Aug 1920, *A. Weberbauer s.n.* (USM); Huancayo, Quilcas, Tunacorral, 11°51'S, 75°9'W, 5 Apr 1994, *G. Yarupaitán, E. Olivera & R. Canto 1355* (USM); **Lima**: cordillera Raura, pr. mina Raura, 10°29'S, 76°44'W, 30 May 2013, *C. Aedo & J. Molina 20538* (MA); Huarochirí, paso de Anticona, 11°35'S, 76°11'W, 1 May 1999, *H. Beltrán & E. Li 3307* (USM); Yauyos, Laraos, pampas de Quiray cerca a la laguna de Huinso, 12°23'S, 75°48'W, 4 Feb 2000, *H. Beltrán 3415* (USM); Yauyos, Laraos, estancia de Quiray, 6 horas de camino al pueblo, 12°24'S, 75°45'W, 11 May 2001, *H. Beltrán 4059* (USM); Yauyos, Laraos, camino de Jalcacha a Palca, 12°24'S, 75°45'W, 4 Nov 1992, *H. Beltrán 406* (USM); Yauyos, Laraos, Malpaso, 12°27'S, 75°40'W, 12 May 2001, *H. Beltrán 4204* (USM); Oyón, compañía minera Iscaycruz, 10°47'S, 76°43'W, 30 May 2008, *H. Beltrán 6509* (USM); Huarochirí, desvío de carretera central hacia Chinchan y Marcapomacocha, 11°34'S, 76°15'W, 23 Sep 2014, *H. Beltrán & W. Aparco 7737* (USM); Huarochirí, desvío de carretera central hacia Chinchan y Marcapomacocha, 11°34'S, 76°15'W, 23 Sep 2014, *H. Beltrán & W. Aparco 7749* (USM); Oyón, encima de mina Uchuchacua, parte más alta por carretera, 10°36'S, 76°40'W, 5 Apr 2015, *H. Beltrán & W. Aparco 7771* (USM); Huarochirí, Chicla, abra Anticona (Ticlio), 11°36'S, 76°11'W, 29 Apr 2017, *H. Beltrán, S. Castillo & M. Arakaki 7965* (USM); límite con dpto. Junín, entre Casapalca y Ticlio, 11°35'S, 76°11'W, 1 Dec 1977, *S. Castroviejo, M. Costa & E. Valdés-Bermejo 1118* (MA); Huarochirí, arriba de Chumpicocha, 11°57'S, 76°9'W, 28 May 1953, *E. Cerrate 2011* (USM); Anticona nevado, 11°35'S, 76°10'W, 8 Aug 1987, *M. Chanco, O. Tovar & A. Galán 1220* (USM); Huarochirí, lago Aguascocha, near mina Caprichosa, above Casapalca, 11°38'S, 76°13'W, 1 Mar 1964, *P.C. Hutchison & O. Tovar 4270* (USM); subida al puerto de Piedra Parada, camino de Lima a Tarma, 26 Sep 1863, *J. Isern 434* (MA); Canta, La Viuda, 11°21'S, 76°26'W, 7 Aug 1964, *I. Meza 228* (USM); Morococha, 11°35'S, 76°8'W, [without date], *A. Raimondi 8389* (USM); Cajatambo, Raura, 10°26'S, 76°47'W, 15 Apr 1988, *S. Rivas et al. s.n.* (USM); Canta, Cullhuay, alrededores de la laguna de Chuchún, 11°22'S, 76°26'W, 17 May 2003, *G. Vilcapoma 6070* (USM); **Moquegua**: General Sánchez Cerro, Yunga, Sura-Perusa, 16°11'S, 70°38'W, 13 Apr 2012, *D. Montesinos 3789* (HSP, USM); General Sánchez Cerro, Yunga, Sura, 16°11'S, 70°38'W, 3 Mar 2018, *D. Montesinos & J. Calvo 5938* (HSP); **Pasco**: abra Ucchuchacua, 10°36'S, 76°40'W, 31 May 2013, *C. Aedo & J. Molina 20584* (MA); Pasco, Huaron, 11°2'S, 76°27'W, Nov 1956, *H. Macedo s.n.* (USM); **Puno**: Carabaya, Corani, Minaspata, 14°2'S, 70°43'W, 15 Oct 2016, *P. Gonzáles 3829* (USM).

### 21. Xenophyllum staffordiae

Taxon classificationPlantaeAsteralesAsteraceae

(Sandwith) V.A.Funk, Novon 7(3): 240. 1997.

43EA3195-D7DE-5508-BF6A-753089957E6E

Werneria staffordiae Sandwith, Hooker’s Icon. Pl. 35: tabula 3424. 1940. Type. Peru. Puno: San Antonio de Esquilache, 4725 m, 14 May 1937, *D. Stafford 734* (lectotype: K-000527744 (digital image!), designated as “holotype” by Funk (1997a: 240); isolectotypes: K s.n.!, BM s.n.!, F-1508962!).

#### Description.

**Figure 26.**
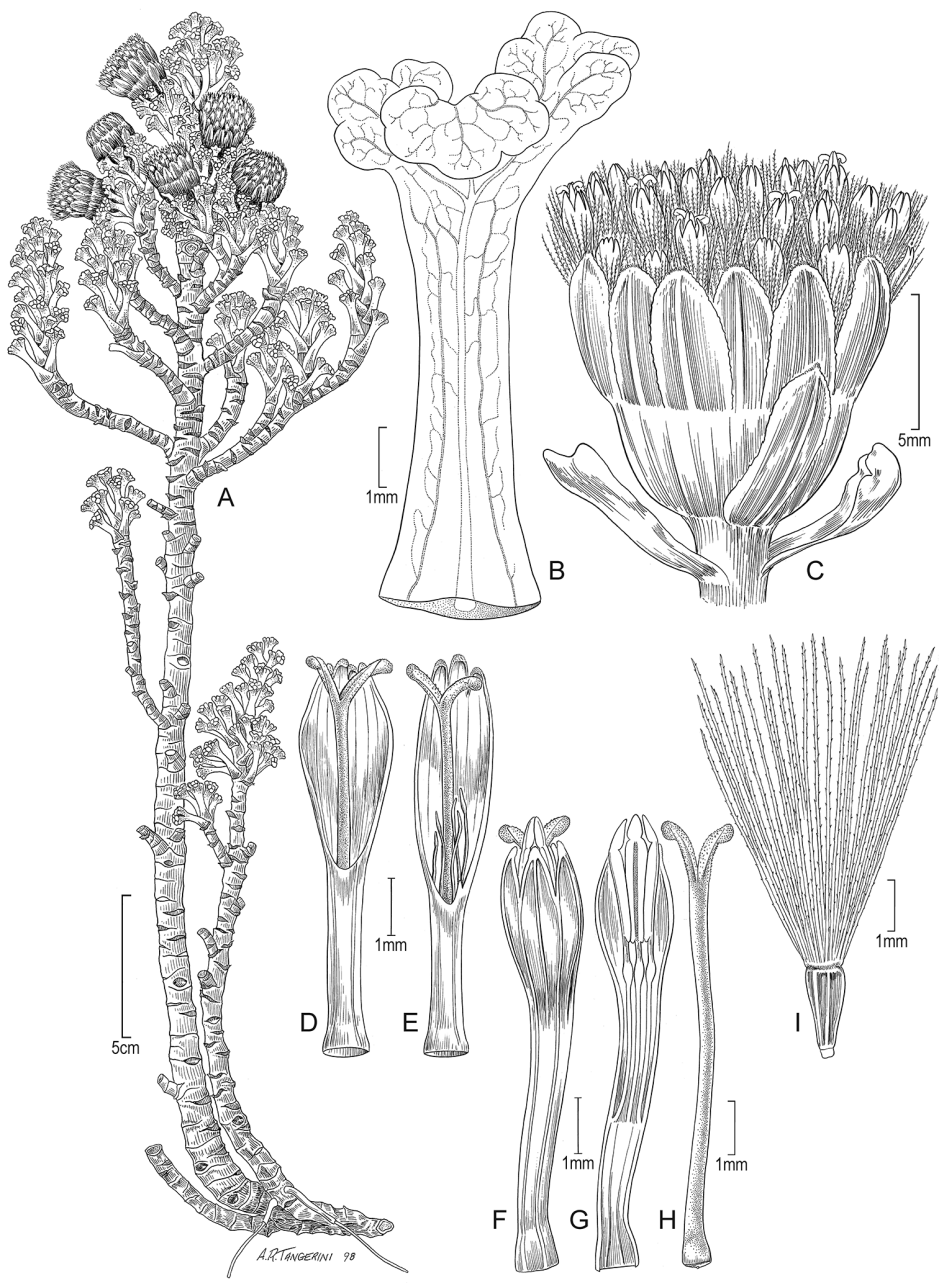
*Xenophyllum staffordiae***A** habit **B** leaf **C** capitulum **D** ray corolla and style **E** ray corolla and style (notice staminodes) **F** disc corolla and style branches **G** disc corolla and stamens (vertically sectioned, style removed) **H** style **I** immature achene with pappus. All details drawn from *Oliver & Pearson 86* (US) except for **A** (drawn from *Stafford 734*, K). Illustration by Alice Tangerini.

Shrubby plant. ***Rhizomes*** ca. 6 × 0.7–1 cm, horizontal to oblique, glabrous. ***Stems*** 30–60 cm tall, branched, glabrous, with leaves restricted to the upper part. ***Leaves*** imbricate, extending into a sheath-like base glabrous or with long silky trichomes; leaf laminas 6.8–9.3 × 3.2–3.7 mm, spatulate (finger-like at the apex), at least 12-divided at the apex, with all divisions similar in length, entire, elliptical to terete upwards in cross section, glabrous or floccose-lanate, 1-nerved above (barely visible), 1-nerved beneath, somewhat fleshy, matte; divisions ca. 1.5 mm long, rounded to truncate. ***Capitula*** radiate, rather nodding, subsessile to shortly pedunculate (up to 5 mm long, sometimes bearing 1 to 2 oblong bracts that reach the involucre). ***Involucres*** 8.6–10.5 × 8.2–10 mm, cupuliform; involucral bracts 11 to 20, 4.3–5 × 2.5–2.8 mm, obtuse at the apex, greenish to slightly purplish. ***Ray florets*** 15 to 28; corollas 6.4–8.3 × 0.8–1.1 mm, unconspicuously veined, subentire to 3-toothed at the apex, not surpassing the involucre, pale yellow. ***Disc florets*** 36 to 81; corollas 7.4–9.2 mm long, yellowish; style branches truncate with a crown of sweeping trichomes, yellowish. ***Achenes*** 3.3–3.6 × 0.9–1 mm, cylindrical, 7 to 9-ribbed, glabrous; pappus 8.2–14.2 mm long, barbellate, whitish. Chromosome number unknown. Fig. 26.

#### Additional iconography.

Sandwith (1940: tabula 3424, sub *Werneria staffordiae*); Beltrán (2016: 359, fig. 3D, as photo).

#### Distribution and habitat.

**Figure 27.**
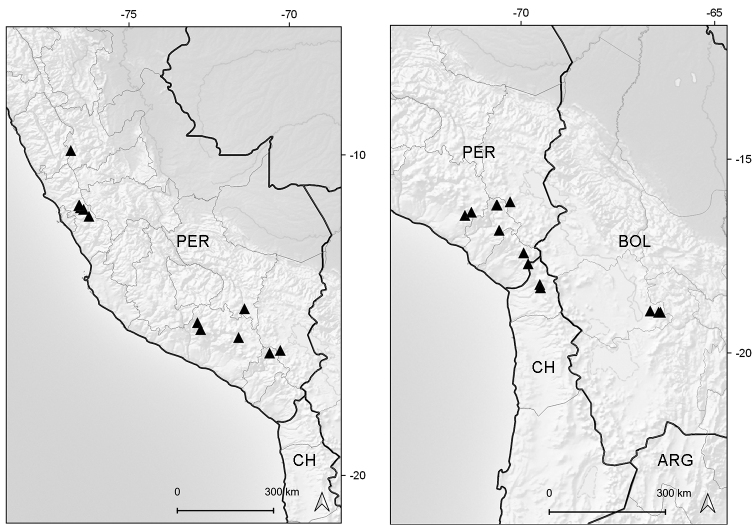
Distribution map of *Xenophyllum staffordiae* (left hand) and *X. esquilachense* (right hand).

Endemic to Peru (Arequipa, Cusco, Huánuco, Lima, Moquegua, Puno). It grows in rocky outcrops, scree slopes, and exposed grasslands of the subhumid puna ecoregion, between elevations of 3900–5200 m (Fig. 27).

*Xenophyllum staffordiae* is known from central and southern Peru, however, a remarkable gap exists between these two distribution centers. No collections were studied from Apurímac, Ayacucho, and Huancavelica.

#### Phenology.

Flowering from March to December.

#### Etymology.

The epithet *staffordiae* honors the English field botanist Dora Stafford, who collected in Peru in the 1930s.

#### Notes.

This is a shrub with 30–60 cm tall stems that have glabrous or floccose-lanate finger-like leaves, with at least 12 divisions at the apex. The divisions are similar in length, ca. 1.5 mm deep. It is characterized by its capitula that tend to be nodding as the plant ages, with 15 to 28 ray florets with pale yellow corollas that do not surpass the involucre. As in the case of *X. poposa*, we observed a few ray florets with staminodes (Fig. 26E). It only might be confused with *X. dactylophyllum* (see comments under it).

It should be mentioned that the elevation indicated on the isolectotype at F is 16000 feet, which slightly differs from that indicated on the lectotype (15500 feet). We believe that such mismatch is not a major reason for excluding it from the type material.

#### Additional specimens examined.

**Peru. Arequipa**: La Unión, Cotahuasi, 15°14'S, 72°52'W, 30 Jun 2002, *F. Cáceres 2806* (HUSA); Caylloma, Chivay, bajada a Chivay, 15°42'S, 71°35'W, 25 Oct 1988, *E. Linares 260* (CUZ, USM); La Unión, entre Solimana y Ccoropuna, base, 15°27'S, 72°46'W, 25 Apr 1967, *C. Vargas 19541* (CUZ, US); Castilla, Tapay, cerro Blanco, Apacheta, 13 Sep 2011, *N. Vega 1734* (USM); **Cusco**: Espinar, Yauri, 14°48'S, 71°24'W, 20 Jun 1944, *C. Vargas 4382* (CUZ, F); **Huánuco**: Huánuco viejo, 9°52'S, 76°49'W, 4 Oct 1968, *I. Meza s.n.* (USM); **Lima**: Huarochirí, cordillera de Carampoma, 11°39'S, 76°31'W, Dec 1929, *N. Esposto s.n.* (MOL, USM); Huarochirí, San Damián, abra entre Chanape y la comunidad Checca, 11°55'S, 76°15'W, 14 Jul 2013, *P. Gonzáles & B. Brito 2667* (USM); Canta, Lachaqui, más arriba de la laguna de Quinán, camino hacia Carampoma (Huarochirí), 11°35'S, 76°34'W, 20 May 1995, *A. Granda 1496* (MOL, US); Canta, cerca a Canta Mishquipuquio, 11°34'S, 76°33'W, 10 Aug 1949, *S. Sánchez 44* (USM); Canta, Lachaqui, arriba de laguna Quinan, 11°35'S, 76°34'W, 30 Jun 2000, *G. Vilcapoma 5194* (USM); Huarochirí, San Juan de Iris, alrededores de Tuktococha, 11°42'S, 76°25'W, 18 Aug 1993, *G. Yarupaitán & J. Albán 1012* (US, USM); **Moquegua**: General Sánchez Cerro, Yunga, Perusa, 16°11'S, 70°37'W, 3 Mar 2018, *D. Montesinos & J. Calvo 5957* (HSP); **Puno**: cerro Ichuasi, Caccachara, ca. 50 miles SW of Ilave, 18 Nov 1946, *P. Oliver & A. Pearson 86* (US); Crestón, San Antonio [de Esquilache], 1 Jul 1937, *T.G. Tutin 1201* (BM).

### 22. Xenophyllum esquilachense

Taxon classificationPlantaeAsteralesAsteraceae

(Cuatrec.) V.A.Funk, Novon 7(3): 239. 1997.

97F64DB2-2667-5409-B52A-DAFA143C0081

Werneria esquilachensis Cuatrec., Brittonia 8: 192. 1956. Type. Peru. Puno: San Antonio de Esquilache, 4725 m, 12 May 1937, *D. Stafford 716* (holotype: K s.n.!).
Senecio pfisteri Ricardi & Martic., Gayana, Bot. 11: 25. 1964. Type. Chile. Arica-Parinacota: Arica, Portezuelo de Chapiquiña, faldeos al lado norte del campamento, 4400 m, 10 Feb 1964, *C. Marticorena, O. Matthei & M. Quezada 116* (holotype: CONC-29863!; isotype: CONC s.n.!).

#### Description.

Suffruticose plant, forming clumps of erect or decumbent stems. ***Rhizomes*** 4–8 × 0.1–0.2 cm, horizontal to oblique, glabrous. ***Stems*** 5–10 cm tall, branched, glabrescent, usually with leaves restricted to the upper part. ***Leaves*** somewhat distantly arranged, abruptly narrowed at the base; leaf laminas 11.2–15.5 × 3.3–4.6 mm, broadly spatulate, 3-forked at the apex, entire and scabrous-ciliate (cilia ca. 0.17 mm long), flat in cross section, glabrous, 1-nerved above (barely visible), 1-nerved beneath, somewhat fleshy, matte, papillose; leaf lobes 2.4–3.9 × 0.7–1 mm, with 2 to 3 rough mucronate teeth up to 1.4 mm long. ***Capitula*** disciform, erect, sessile to subsessile. ***Involucres*** 10.4–15.1 × 6.5–7.4 mm, cupuliform; involucral bracts 8 to 12, 5.4–7.2 × 1.3–2.5 mm, acute to obtuse at the apex, greenish, sometimes purple-edged. ***Peripheral florets*** ca. 10, pistillate; corollas reduced to a vestigial tube ca. 2.1 mm long or without corolla. ***Disc florets*** ca. 15; corollas 6–10.6 mm long, purplish; style branches truncate with a crown of sweeping trichomes, purplish. ***Achenes*** cylindrical, glabrous (immature); pappus ca. 11.3 mm long, barbellate, whitish. Chromosome number unknown.

#### Iconography.

Calvo et al. (2018: 289, fig. 1, as photo).

#### Distribution and habitat.

Southern Peru to central-western Bolivia. Bolivia (Oruro), Chile (Arica-Parinacota), Peru (Arequipa, Moquegua, Puno, Tacna). It grows in rocky outcrops, steep volcanic slopes, and open shrublands of the subhumid and dry puna ecoregions, between elevations of 3700–4950 m (Fig. 27).

#### Phenology.

Flowering from February to June.

#### Etymology.

It is named after the Peruvian village of San Antonio de Esquilache (western Puno), where the type material was collected in 1937 by Dora Stafford.

#### Notes.

This is the unique species within the genus displaying disciform capitula. They have ca. 10, peripheral, pistillate florets with a corolla reduced to a vestigial tube ca. 2.1 mm long or without corolla, and therefore, the style is exsert in its whole length or almost so. Another distinctive feature is the morphology of the leaves; they are 3-forked, abruptly narrowed at the base, and marginally scabrous-ciliate. Each lobe usually has 2 to 3 rough mucronate teeth. Any confusion with the other members of the genus is unlikely.

See Calvo et al. (2018) for further details on the type material of the name *Senecio pfisteri* Ricardi & Martic.

#### Additional specimens examined.

**Bolivia. Oruro**: Challapata, Livichuco, Tarpata, estribaciones del cerro Toro en el collado antes de las lagunas, 18°57'S, 66°24'W, 15 Feb 2019, *J. Calvo & M. Zárate 7877* (BOLV); prov. Eduardo Abaroa, Challapata, comunidad Churacani, 18°55'S, 66°40'W, 1 Apr 2018, *M. Guzmán 124* (LPB); Abaroa, Challapata, cerro Toro Ichurata, en línea recta a 34.38 km al E, 18°57'S, 66°27'W, 27 Feb 2016, *F. Zenteno, L. Moya & D. Villalba 16745* (LPB). **Chile. Arica-Parinacota**: quebrada Cataguanchuta, 18°14'S, 69°31'W, 17 Dec 1988, *E. Belmonte 88644* (CONC); pasado azufrera Tacora, 17°42'S, 69°49'W, 17 Mar 2015, *A. Moreira-Muñoz & F. Luebert 2407* (SGO); Portezuelo de Chapiquiña, 18°19'S, 69°30'W, 26 Mar 1961, *M. Ricardi, C. Marticorena & O. Matthei 207* (CONC). **Peru. Arequipa**: Chiguata, faldas del nevado Pichu Pichu, 16°27'S, 71°27'W, 20 Jun 2004, *V. Quipuscoa et al. 2949* (HSP); Chiguata, ca. 2 km arriba del túnel del Simbral, carretera a Salinas, 16°22'S, 71°17'W, 15 Apr 2018, *V. Quipuscoa & M. Balvin 7541* (HSP); **Moquegua**: Mariscal Nieto, Carumas, cerca a un acueducto, 16°50'S, 70°34'W, 15 Jun 2013, *H. Beltrán 7734* (USM); General Sánchez Cerro, Yunga, Sura-Perusa, 16°11'S, 70°38'W, 13 Apr 2012, *D. Montesinos 3804* (HSP); General Sánchez Cerro, Yunga, Perusa, 16°11'S, 70°37'W, 3 Mar 2018, *D. Montesinos & J. Calvo 5958* (HSP); **Tacna**: Tarata, Poma, carretera Tarata-Puno, 17°25'S, 69°56'W, 25 Mar 1998, *A. Cano 8139* (USM).

## Supplementary Material

XML Treatment for Xenophyllum

XML Treatment for Xenophyllum acerosum

XML Treatment for Xenophyllum humile

XML Treatment for Xenophyllum sotarense

XML Treatment for Xenophyllum roseum

XML Treatment for Xenophyllum funkianum

XML Treatment for Xenophyllum rigidum

XML Treatment for Xenophyllum crassum

XML Treatment for Xenophyllum crassum subsp. crassum

XML Treatment for Xenophyllum crassum subsp. orientale

XML Treatment for Xenophyllum marcidum

XML Treatment for Xenophyllum ciliolatum

XML Treatment for Xenophyllum juniperinum

XML Treatment for Xenophyllum weddellii

XML Treatment for Xenophyllum incisum

XML Treatment for Xenophyllum poposa

XML Treatment for Xenophyllum lorochaqui

XML Treatment for Xenophyllum rosenii

XML Treatment for Xenophyllum digitatum

XML Treatment for Xenophyllum pseudodigitatum

XML Treatment for Xenophyllum decorum

XML Treatment for Xenophyllum amblydactylum

XML Treatment for Xenophyllum dactylophyllum

XML Treatment for Xenophyllum staffordiae

XML Treatment for Xenophyllum esquilachense

## Appendix I

Index to the taxonomic names treated, with the currently accepted names indicated in bold type.

*Oresigonia* Willd. ex Less.

*brevifolia* Willd. ex Rockh. = ***Xenophyllum humile***

*pycnophylla* Willd. ex Rockh. = ***Xenophyllum rigidum***

*Senecio* L.

*pfisteri* Ricardi & Martic. = ***Xenophyllum esquilachense***

*Werneria* Kunth

*acerosa* Cuatrec. = ***Xenophyllum acerosum***

*amblydactyla* S.F.Blake = ***Xenophyllum amblydactylum***

*articulata* S.F.Blake = ***Xenophyllum humile***

*castillensis* Hieron. = ***Xenophyllum pseudodigitatum***

*ciliata* Wedd. ex Sch.Bip. = ***Xenophyllum ciliolatum***

*ciliolata* A.Gray = ***Xenophyllum ciliolatum***

crassa f. minor Cuatrec. = **Xenophyllum crassum subsp. orientale**

*crassa* S.F.Blake = ***Xenophyllum crassum***

crassa subsp. orientalis Cuatrec. = **Xenophyllum crassum subsp. orientale**

dactylophylla f. glabriuscula Rockh. = ***Xenophyllum dactylophyllum***

*dactylophylla* Sch.Bip. = ***Xenophyllum dactylophyllum***

dactylophylla var. glanduloso-denticulata Rockh. [unverified]

*decora* S.F.Blake = ***Xenophyllum decorum***

*decumbens* Hieron. = ***Xenophyllum weddellii***

digitata var. lanata Rockh. = ***Xenophyllum digitatum***

*digitata* Wedd. = ***Xenophyllum digitatum***

*esquilachensis* Cuatrec. = ***Xenophyllum esquilachense***

*fontii* Cuatrec. = ***Xenophyllum humile***

humilis f. articulata (S.F.Blake) Rockh. = ***Xenophyllum humile***

*humilis* Griseb. ex Hieron. = ***Xenophyllum poposa***

*humilis* Kunth = ***Xenophyllum humile***

humilis var. angustifolia Cuatrec. = ***Xenophyllum humile***

humilis var. fontii Cuatrec. = ***Xenophyllum humile***

humilis var. lindenii Wedd. = ***Xenophyllum humile***

humilis var. rosea (Hieron.) Rockh. = ***Xenophyllum roseum***

*humilis* var. β Wedd. = ***Xenophyllum humile***

*incisa* Phil. = ***Xenophyllum incisum***

incisa var. pubescens Rockh. = ***Xenophyllum poposa***

*juniperina* Hieron. = ***Xenophyllum juniperinum***

*lehmannii* Hieron. = ***Xenophyllum humile***

*lehmannii* Klatt = ***Hypochaeris sessiliflora***

*leucobryoides* S.F.Blake = ***Xenophyllum sotarense***

*lorentziana* Hieron. = ***Xenophyllum poposa***

*lycopodioides* S.F.Blake = ***Xenophyllum juniperinum***

*marcida* S.F.Blake = ***Xenophyllum marcidum***

*poposa* Phil. = ***Xenophyllum poposa***

*pseudodigitata* Rockh. = ***Xenophyllum pseudodigitatum***

*purpurea* Spruce ex Rockh. = ***Xenophyllum roseum***

*rigida* Kunth = ***Xenophyllum rigidum***

*rosea* Hieron. = ***Xenophyllum roseum***

*rosenii* R.E.Fr. = ***Xenophyllum rosenii***

sect. Aciculares Rockh. = ***Xenophyllum***

sect. Digitifoliae Rockh. = ***Xenophyllum***

*sedoides* S.F.Blake = ***Xenophyllum marcidum***

*sotarensis* Hieron. = ***Xenophyllum sotarense***

*staffordiae* Sandwith = ***Xenophyllum staffordiae***

subg. Euwerneria Rockh. = ***Xenophyllum***

*weddellii* Phil. = ***Xenophyllum weddellii***

***Xenophyllum*** V.A.Funk

***acerosum*** (Cuatrec.) V.A.Funk

***amblydactylum*** (S.F.Blake) V.A.Funk

***ciliolatum*** (A.Gray) V.A.Funk

***crassum*** (S.F.Blake) V.A.Funk

**crassum subsp. crassum**

**crassum subsp. orientale** (Cuatrec.) J.Calvo

***dactylophyllum*** (Sch.Bip.) V.A.Funk

***decorum*** (S.F.Blake) V.A.Funk

***digitatum*** (Wedd.) V.A.Funk

***esquilachense*** (Cuatrec.) V.A.Funk

*fontii* (Cuatrec.) V.A.Funk = ***Xenophyllum humile***

***funkianum*** J.Calvo

***humile*** (Kunth) V.A.Funk

***incisum*** (Phil.) V.A.Funk

incisum var. pubescens (Rockh.) Cabrera & S.E.Freire = ***Xenophyllum poposa***

***juniperinum*** (Hieron.) J.Calvo

***lorochaqui*** J.Calvo & V.A.Funk

*lycopodioides* (S.F.Blake) V.A.Funk = ***Xenophyllum juniperinum***

***marcidum*** (S.F.Blake) V.A.Funk

*oscartovarii* E.Linares, J.Campos, Nauray, Vicente Orell. & A.Galán = ***Xenophyllum dactylophyllum***

***poposa*** (Phil.) V.A.Funk

***pseudodigitatum*** (Rockh.) V.A.Funk

***rigidum*** (Kunth) V.A.Funk

***rosenii*** (R.E.Fr.) V.A.Funk

***roseum*** (Hieron.) V.A.Funk

***sotarense*** (Hieron.) V.A.Funk

***staffordiae*** (Sandwith) V.A.Funk

***weddellii*** (Phil.) V.A.Funk

## Appendix II

List of exsiccatae. *Xenophyllum* specimens are listed alphabetically by collector, followed by collection number or date when a collection number was not assigned. Specimens without collection number and date are not listed. The number in parentheses corresponds to the number of the accepted species in the treatment.

***A****costa, C.E. 12* (2), *13* (2); *Aedo, C. 6968* (11); *Aedo, C. & A. Galán 11037* (20), *11103* (9); *Aedo, C. & J. Molina 20440* (20), *20538* (20), *20584* (20); *Aguirre, J. & A.M. Cleef 880* (7b); *Albán, J. & G. Yarupaitán 8094* (13); *André, E. 3281* (2); *Ángel, M. et al. 9* (2); *Anthony, H.E. & G.H.H. Tate 316* (6); *Arancio, G. 92-341* (12), *92-454* (11), *10676* (12), *10697* (12); *Argomedo, M. 9* (11); *Arroyo, M. 84-728* (11), *84-857* (10), *84-912* (10), *84-913* (13), *85-595* (13); *Arroyo, M., L. Cavieres & A. Humaña 97022* (13), *97217* (12), *97276* (12), *97315* (12), *97373* (12), *97463* (12); *Arroyo, M., C. Villagrán & J. Armesto 85-394B* (10); *Arroyo, M., C. Villagrán & J. Moreno 2622* (13), *2623A* (10), *2660* (16); *Asplund, E. 7819* (2), *8391* (6), *8612* (2), *17334* (6); *Ávila, F. 2232* (7a), *2236* (7a), *2247* (2), *2252* (2), *2259* (2), *2272* (2); *Ayarde, H. 369* (13).

***B****aeza, M., P. Aqueveque & G. Kottirsch 588* (13); *Baines, R. et al. 363* (11); *Balls, E.K. 7308* (6); *Balslev, H., J. Brandbyge & L. Coloma 3347* (2); *Barbosa, C. 9586* (2); *Barclay, H.G. 5017* (2), *5104* (2), *5137* (2), *5238* (2), *5285* (7a); *Barclay, H.G. & P. Juajibioy 6220* (2), *6245* (7a), *6409* (7a), *7352* (7b), *8782* (2), *8855* (7a), *8900* (2), *10363* (7b), *10370* (7b); *Bardón, A. 22 Jan 2000* (12); *Barra (de la), N. et al. 1026* (20); *Barrón, D. 57* (20); *Baumann, G. 352* (12); *Beck, S.G. 1* (20), *183* (20), *2425* (20), *11347* (20), *18750* (2), *24639* (9), *26468* (20), *27510* (12), *31109* (10), *31142* (13), *31148* (10), *32410* (12), *32617* (11), *32750* (10), *32753* (10), *32757* (10), *33940* (20), *34533* (10), *35111* (20); *Beck, S.G., D. Ibáñez & C. Beck 34084* (20); *Beck, S.G. et al. 21519* (2); *Becker, B. & F.M. Terrones 303* (2), *328* (2); *Behn, F. 19596* (12); *Belmonte, E. 20005* (10), *86119* (11), *88090* (13), *88619* (10), *88635* (10), *88644* (22), *88668* (16), *96068* (11), *97049* (11); *Beltrán, H. 406* (20), *414* (13), *1702* (9), *1715* (13), *2579* (16), *3415* (20), *3418* (18), *4059* (20 and 18), *4174* (19), *4188* (18), *4193* (16), *4204* (20), *5863* (16), *6509* (20), *7100* (9), *7101* (8), *7102* (13), *7111* (9 and 16), *7114* (16), *7115* (20), *7672* (16), *7734* (22), *7736* (13), *8086* (9), *8469* (18); *Beltrán, H. & W. Aparco 7737* (20), *7740* (18), *7749* (20), *7750* (8), *7769* (8), *7771* (20), *7842* (9); *Beltrán, H. & E. Li 3305* (16), *3306* (18), *3307* (20); *Beltrán, H., S. Castillo & M. Arakaki 7964* (16), *7965* (20), *7986* (8), *7988* (18), *7989* (16); *Benavides, J.C. 4859* (7a); *Bensman, R. 358* (6), *370* (2); *Bernardi, L., A. Charpin & F. Jacquemoud 16772* (9); *Betancur, J. & M. Gutiérrez 11215* (2); *Betancur, J., F. Gómez & J.N. Gómez 14253* (7a); *Biegman, K. 8 Aug 1982* (18); *Black, J. 163* (6); *Boeke, J.D. 650* (4); *Boeke, J.D. & J.B. McElroy 294* (2); *Bosco, J. 145* (2); *Bosco, J. & M. Marcillo 72B* (2); *Bourdy, G. 2197* (10); *Brooke, W.M. 6322* (20); *Bussmann, R. & S. Lange 22 Oct 1996* (2), *25 Oct 1997* (2).

***C****aballero, L.M. et al. 31* (2); *Cabrera, A.L. 8370* (13); *Cabrera, A.L., J. Crisci & R. Kiesling 19843* (17); *Cabrera, A.L. et al. 19009* (17); *Cáceres, F. 2806* (21), *2807* (13), *5508* (13), *5732* (13); *Cáceres, F., E. López & L. Castillo 6025* (11), *6063* (11), *6149* (11), *6178* (11); *Calderón, E. 43A* (2), *88A* (7a); *Calleja, J.A. & J.C. Jaramillo 204* (7b); *Calvo, J. 7681* (2), *7731* (13), *7909* (13), *7914* (12), *7924* (12); *Calvo, J. & A. Moreira-Muñoz 7930* (10), *7935* (13); *Calvo, J. & M. Zárate 7869* (13), *7871* (16), *7877* (22); *Calvo, J., T. Buira & C. Velasco 7622* (2), *7640* (3); *Camacho, V. & M. Rendon 1* (20); *Camargo, L.A. 7406* (2); *Camelo, L., J. Castillo & J. Bernal 205* (2); *Cano, A. 7944* (13), *7946* (10), *7953* (13), *8139* (22), *8244* (10), *11496* (8); *Cano, A. & H. Trinidad 20646* (18); *Cano, A. & N. Valencia 10106* (20); *Cano, A., B. Britto & N. Valencia 21926* (16); *Cano, A., M.I. La Torre & W. Mendoza 14536* (20); *Cano, A., W. Mendoza & A. Delgado 19658* (9), *19686* (16), *19721* (9), *19734* (16), *19760* (8); *Cano, A., A. Ramírez & C. Cáceres 10549* (20); *Cano, A. et al. 7388* (2), *9252* (20), *9733* (20), *14168* (18), *19378* (20), *19436* (9), *19849* (9), *19873* (16), *20203* (18), *21319* (20); *Capacho-Navia, D.I. et al. 243* (7b), *362* (7b); *Caranqui, J. 2439* (2); *Caranqui, J., W. Haro & F. Salas 2182* (2); *Cárate, D., F. Naranjo & S. Chiriboga 1054* (7a); *Cárate, D., J. Salvador & S. Rojas 151* (7a); *Cárate, D. et al. 593* (6), *609* (2), *646* (2), *675* (2), *950A* (2); *Cárdenas, M. 362* (13); *Cardich, A. 208* (8), *217* (8); *Castaño, N. & A. Orejuela 1429* (2); *Castellanos, A. 11 Mar 1927* (12), *28 Jan 1943* (14); *Castillo, M. 20 Oct 1976* (16); *Castillo, V. 3 Dec 1948* (11); *Castillón, L. 1761* (17), *3302* (13), *3360* (13), *3365* (14), *7140* (17), *Jan 1916* (13 and 14); *Castroviejo, S., M. Costa & E. Valdés-Bermejo 1053* (2), *1118* (20), *1120* (16); *Ceballos, A. et al. 8* (16), *472* (20); *Cerón, C.E. & R. Alarcón 12307* (2); *Cerón, C.E., M. Cerón & G. Viteri 4397* (2), *4408* (2), *4423* (2); *Cerrate, E. 998* (20), *1513* (20), *2006* (9), *2011* (20), *2651* (20); *Cerrate, E., C. Acleto & J. Gómez 4289* (16); *Cerrate, E., B. León & J. Campos 8400* (20); *Cerrate, E. et al. 4907* (16); *Chanco, M., O. Tovar & A. Galán 1220* (20); *Clark, J.L. 725* (6), *2219* (2), *2534* (2); *Clark, J.L. & J. Fair 3521* (7a); *Cleef, A.M. 294* (2), *1316* (2), *5277* (2), *7628* (2), *8736* (7b), *8762* (7b), *9411a* (7b); *Cleef, A.M. & P.A. Florschütz 5469* (2), *5784* (7b), *5870* (7b); *Collan, J.S. 44* (12); *Condo Klaus, S. 15* (13); *Core, E.L. 1587* (2); *Croat, T.B. et al. 104336* (2); *Cuamacás, B. & E. Gudiño 473* (2); *Cuatrecasas, J. 1486* (7b), *2862* (2), *9283* (2), *13505* (7b), *13511* (7b), *19095* (2), *20322* (7a), *23219* (2); *Cuatrecasas, J. & H. García Barriga 10012* (7b); *Cuatrecasas, J. & J.M. Idrobo 27005* (2); *Cuatrecasas, J. & R. Jaramillo 25746* (2); *Cuatrecasas, J. & L.E. Mora 26899* (2); *Cuatrecasas, J. & L. Rodríguez 27887* (7b); *Cuatrecasas, J., M.T. Murillo & R. Jaramillo 25628* (2); *Cuello, S. 154* (13); *Cueva, E. 207* (2).

***D****íaz, A. 28 Dec 1999* (2); *Díaz, (?) Mar 1933* (14); *Díaz Ibarra, S.L. 2087* (2); *Díaz-Piedrahita, S. 1131* (2); *Díaz-Piedrahita, S. & R. Jaramillo 1798* (2), *1918* (7a); *Díaz-Piedrahita, S., H. Valencia & R. Jaramillo 19700* (7a); *Díaz-Piedrahita, S. et al. 2792* (2); *Diers, L. 940* (8); *Dillon, M. & B.L. Turner 1300* (20), *1308* (18), *1478* (16); *Dorr, L.J. & L.C. Barnett 6035* (2); *Dorr, L.J. & I. Valdespino 6474* (5); *Downey, C. 131* (2); *Duque, A. 559* (2).

***E****hrenburg, A. 172* (6); *Ellenberg, H. 4295* (17); *Endara, L. & M. Nonhebel 496* (2), *554* (4); *Eriksen, B. 59003* (2), *59010* (7a); *Eriksen, B. & B.B. Larsen 45435* (7a); *Escobar, H. 206* (11); *Espinoza, I. 70* (13); *Esposto, N. Dec 1929* (21); *Essel, 23* (20); *Ewan, J.A. 16323* (2).

***F****ernández, A. 2897* (2); *Fernández, D. et al. 1739* (2), *1743* (2); *Fernández, A. & L.E. Mora 1136* (2); *Fernández, D., M. Cerna & P. Villacrés 325* (2); *Fernández, D., G. Pérez & L. Calvopiña 416* (7a); *Festa, E. Dec 1897* (3); *Feuerer, T. 4228* (20), *4335a* (20), *8472* (20); *Fiebrig, A. 15 Feb 1953* (17); *Filipòvich, R. 61* (13); *Firmín, F.G. 531* (2); *Fournet, A. 815* (20); *Freire, A. 27* (7a); *Freire, A. & L. Haro 2953* (6); *Fries, R.E. 862* (15); *Fuentes, A. & C. Aldana 6721* (20); *Funk, V.A. 8044* (7a), *8046* (7a), *8091* (2), *8093* (2), *11351* (13), *11352* (10), *11403* (9), *11408* (20), *11422* (6); *Funk, V.A. & N. Bernal 11282* (20), *11285* (8), *11287* (20); *Funk, V.A. & J.M. Bonifacino 14028* (2), *14031* (2), *14033* (4), *14053* (6), *14059* (5), *14061* (5), *14094* (2); *Funk, V.A. & M. Estárez 11360* (13); *Funk, V.A. & M. Gavilanes 11069* (7a), *11070* (7a), *11071* (2), *11072* (2), *11073* (7a), *11075* (2), *11077* (7a), *11078* (2); *Funk, V.A. & C. González-Quint 11371* (20), *11372* (20); *Funk, V.A. & L. Katinas 11157* (17), *11169* (17); *Funk, V.A. & X. Montezuma 11426* (4), *11431* (2), *11432* (2), *11433* (2), *11441* (4), *11442* (2), *11443* (2), *11444* (2); *Funk, V.A. & R.X. Zamora 11450* (2); *Funk, V.A., M. Diazgranados & J.M. Bonifacino 13082* (11), *13087* (11), *13127* (11), *13133* (11), *13077* (12), *13078* (12), *13103* (10), *13136* (10); *Funk, V.A., M. Diazgranados & E. Cochachin 13138* (13), *13139* (10), *13142* (13), *13147* (13), *13148* (13), *13149* (13), *13150* (13), *13151* (13), *13155* (13), *13167* (13), *13168* (13), *13169* (16), *13171* (16), *13180* (9); *Funk, V.A. et al. 11024* (2).

***G****arcía, E. 1101* (12); *García, C. 86* (16), *87* (16); *Gardner, M.F. & S.G. Knees 6266* (11), *6525* (11); *Garganta, M. 488* (2); *Gentry, A. et al. 30523* (2), *37431* (20), *39749* (16); *Gentry, A. & R. Tredwell 37301* (18), *37301* (16); *Gibby, M. & J.A. Barrett 185* (2); *Giraldo-Cañas, D. et al. 6026* (2); *Gonzáles, P. 3528* (20), *3544* (8), *3545* (9), *3559* (16), *3562* (16), *3829* (20), *3832* (8), *3837* (9); *Gonzáles, P. & B. Brito 2629* (16), *2632* (8), *2642* (13), *2655* (18), *2658* (9), *2665* (9), *2667* (21); *Gonzáles, P. & N. Valencia 1921* (20); *Gonzáles, P. et al. 2780* (20), *2781* (16), *2783* (18), *3329* (18); *Granda, A. 1496* (21), *2491* (2); *Grau, A. 26 Jul 1984* (13); *Grubb, P.J. et al. 569* (6), *630* (3), *704* (6); *Guerrón, E. 13* (2), *15* (7a); *Gutte, P. & B. Herzog 702* (20); *Guzmán, M. 124* (22).

***H****alloy, S. B-62* (6), *B-182* (6); *Hammen, T. 506* (2); *Heinrichs, E. 652* (6); *Herrera, F.L. 1033* (16), *1056* (16), *1081* (16); *Herzog, B. 795* (8); *Herzog, T. 2094* (20); *Hoffmann, A. 7 Dec 1988* (12); *Holm-Nielsen, L. 16403* (2), *20787* (2), *24224* (2), *24245* (2); *Holm-Nielsen, L. & J. Jaramillo 28012* (2), *28183* (2), *28186* (2), *28223* (2), *28423* (2), *28475* (2), *28722* (2), *28781* (2); *Holm-Nielsen, L., J. Jaramillo & T. Vries 17109* (2), *17126* (2), *17204* (2), *17312* (2); *Holm-Nielsen, L., B. Øllgaard & C. Sperling 24899* (2), *24959* (2), *24982* (2); *Holt, P. 2* (20), *3* (20), *14A* (9), *15A* (20); *Holzapfel, A. 17* (13); *Huertas, G. & L.A. Camargo 5831* (2); *Humbert, H. 29/30 May 1958* (16); *Humbert, H. et al. 30793* (16); *Hunziker, A.T. & A.E. Cocucci 20019* (13); *Huon, A. & R.I. Meneses 1* (20); *Hutchison, P.C. 1212* (16 and 18); *Hutchison, P.C. & O. Tovar 4270* (20); *Huttel, C. 1385* (2).

***I****drobo, J.M. & H.G. Barclay 4039* (2); *Idrobo, J.M., P. Pinto & H. Bischler 3109* (2); *Isern, J. 43* (2), *83* (6), *313* (2), *404* (20), *434* (20).

***J****ameson, W. Sep 1864* (4), *Oct 1864* (4), *Dec 1860* (2); *Jaramillo, J. 4129* (2), *6091* (7a), *6297* (2), *7202* (2), *7359* (2); *Jaramillo, J. & F. Coello 2078* (2); *Jaramillo, J. & M. Lascano 3142* (2); *Jaramillo, J. & V. Winnerskjold 5497* (2); *Jaramillo, J., R. Narváez & F. Coello 3168* (2); *Jaramillo, J., V. Zak & J. Yépez 6004* (7a), *6262* (7a); *Jiménez, I. 8406* (9); *Jiménez, I. & R. Villegas 8127* (13); *Jiménez, I. et al. 7981* (13), *7989* (13); *Jordan, E. 1* (20), *158* (10); *Jørgensen, P.M., C. Ulloa & M. Gavilanes 92210* (2); *Jørgensen, P.M., C. Ulloa & E. Narváez 2083* (4), *2098* (2), *2117* (2); *Jørgensen, P.M. et al. 1738* (4), *92321* (2).

***K****essler, M. 2840* (20); *Kessler, M. et al. 10144* (20); *King, R.M., P.M. Peterson & E.J. Judziewicz 10052* (2); *Krapovickas, A. 3209* (12), *3215* (14); *Kurtz, F. 14632* (13).

***L****a Torre, M.I. 2375* (10); *Laegaard, S. 51328* (7a), *52249* (2); *Laegaard, S. et al. 21588* (2); *Lara, R. 1499* (20); *Larsen, B.B. & B. Dall 190* (2), *191* (7a); *Lechler, W. 1807* (20); *Lehmann, F.C. 5687* (4), *6230* (2); *León, S. 1012* (7a), *1116* (2), *1135* (2), *1136* (2); *León, B. & K. Young 3481* (2); *Levi, U. 11* (11); *Liberman, M. 152* (13), *191* (16), *206* (12), *352* (16), *812* (10); *Lillo, M. Dec 1914* (13); *Linares, E. 260* (21); *Linares, E. & A. Galán 2354* (20), *3077* (8), *3099* (16), *3100* (16), *3101* (18); *Lloyd, J.R. & J.K. Marshall 309* (8); *Løjtnant, B. & U. Molau 12942* (2); *Lorentz, P.G. & G. Hieronymus 96* (17), *117* (13); *Loyola, L. 93-4* (13); *Loza de la Cruz, F. 264* (11); *Lozano, P. et al. 79* (2); *Luteyn, J.L., J. Fuertes & O. Rangel 12826* (2).

***M****aass, B. 575* (18); *Macbride, J.F. 3032* (8); *MacBryde, B. & J.D. Dwyer 1207* (2); *Macbride, J.F. & F. Featherstone 849* (18), *2475* (8); *Macedo, H. Nov 1956* (20); *Mahu, M. 4108* (16); *Maisch, K. 150* (20); *Mallea, J. 15* (10), *22* (13), *24* (10); *Mandon, G. 101* (20); *Marino, F. 183* (13); *Marticorena, C., O. Matthei & M. Quezada 110* (16), *116* (22), *198* (11), *405* (12); *Mena, P. 43* (2), *192* (2); *Meneses, R.I. et al. 5446* (16), *5467* (16); *Menhofer, X. 10* (9), *23* (9), *1108* (20), *1420* (20), *1461* (8), *1461A* (8), *1506* (10), *2344* (20), *2345* (20); *Merino, B., Á. Sánchez 14 Jan 1999* (2 and 7a); *Meyer, T. 8282* (13), *18476* (13); *Meza, I. 227* (18), *228* (20), *4 Oct 1968* (21); *Mille, L. 469* (2); *Minga, D. & F. Nugra 1594* (4); *Minga, D. & A. Verdugo 2567* (2); *Minga, D., M. Jiménez & N. Guzmán 3242* (4); *Molau, U., B. Eriksen & B.B. Klitgaard 2403* (2); *Mondragón, E. & J. Postigo 90* (16); *Montesinos, D. 549* (13), *2950* (9), *2951* (20), *3090* (11), *3098* (13), *3789* (20), *3804* (22), *3933* (13); *Montesinos, D. & J. Calvo 5938* (20), *5956* (9), *5957* (21), *5958* (22), *5984* (11), *5999* (16), *6004* (13), *6006* (9); *Mora, L.E. 1101* (2), *1881a* (2), *1883* (2), *7536* (2); *Moraes, M. 150* (20), *153* (9); *Moreira-Muñoz, A. 257* (12), *289* (12), *298* (12), *1936* (10), *1941* (11), *2495* (16), *2495A* (11), *2500* (13); *Moreira-Muñoz, A. & M. Diazgranados 2625* (11); *Moreira-Muñoz, A. & F. Luebert 2407* (22), *2408* (10), *2469* (13), *2471* (13); *Moreira-Muñoz, A., M. Muñoz & V. Morales 1640* (10), *1658* (11); *Moreira-Muñoz, A. et al. 2016* (11), *2022* (11), *2037* (10), *2038* (13); *Moreno, J. 2668* (11); *Morueta-Holme, N., K. Engemann & P. Sandoval 84* (2); *Muñoz, L. 339* (2), *365* (6); *Muñoz, E.L. 915* (2); *Murgia, O. 390* (9).

***N****aessany, L. 15* (13); *Narváez, E. & W. Quizhpe 302* (2); *Navarro, G. 596* (9), *628* (9), *637* (9); *Neill, D. et al. 11495* (6), *11959* (2), *12049* (7a), *12064* (7a), *12077* (2), *12095* (2), *12104* (3), *12112* (2), *12167* (3); *Niemeyer, H., C. Fernández & A. Hoffmann 8992* (11).

***O****lea, D. 252* (14); *Oliver, P. & A. Pearson 86* (21); *Øllgaard, B. & H. Balslev 8749* (2); *Øllgaard, B. & L. Holm-Nielsen 38755* (2); *Øllgaard, B. et al. 34196* (2), *34456* (2), *38514* (7a), *38552* (2), *38573* (2); *Orbigny, A.D. (d’) 1407* (16); *Ostria, C. 48* (20), *54* (9), *119* (20).

***P****alacios, W. 11636* (2), *11680* (7a); *Pallardelly, E. 1* (16); *Pasaca, V. 1397* (2); *Pedraza, P. & M. Alvear 667* (2); *Pedraza, P. & P. Franco 452* (2); *Pedraza, P., C. Pedraza & A. Varón 614* (2); *Pedraza, P. et al. 274* (2); *Pennell, F.W. 3039* (2); *Pennell, F.W. & T.E. Hazen 10020* (2), *10031* (7a); *Peña, L. 5* (12); *Peraldo, A. 3794* (13); *Petersen, E. & J.P. Hjerting 159* (17), *1090* (11); *Peterson, P.M., R.J. Soreng & S. Laegaard 13040* (15); *Peyton, B. 97* (20); *Philippi, F. 18 Feb 1885* (12 and 13), *19 Feb 1885* (12), *1 Mar 1885* (11); *Pierotti, S.A. 7496* (13), *7508* (13); *Pietrellini, F. 193* (13); *Pinto, P. & J. Hernández 485* (2); *Pinto, P. et al. 1829* (2); *Pisano, E. & J. Venturelli 1724* (16); *Pittier, H. 1418* (2); *Plowman, T. 1954* (2); *Prieto, F. 280* (1), *285* (2).

***Q****uipuscoa, V. & M. Balvin 7541* (22); *Quipuscoa, V. et al. 2947* (13), *2949* (22), *5570* (11).

***R****aimondi, A. 1589* (20), *8389* (20); *Ramírez, W. 585* (20); *Ramírez, B.R. & J.F. Álvarez 21060* (2); *Ramsay, P.M. & P.J. Merrow-Smith 140* (7a), *186* (2), *448* (4), *726* (2), *759* (2), *866* (2), *869* (7a); *Rangel, O. 1655* (2); *Rangel, O., H. Sturm & S. Zuluaga 1734* (2); *Rangel, O. et al. 4406* (7a); *Reina, G. 472* (2); *Restrepo, B. 430* (7a); *Reyes, D. et al. 3688* (2); *Ricardi, M. 3370* (13), *3377* (10), *3379* (11), *3405* (10); *Ricardi, M., C. Marticorena & O. Matthei 201* (16), *207* (22); *Rivas, S. et al. 15 Apr 1988* (20), *Dec 1986* (16); *Roca Ramos, B. 1* (20), *2* (20), *3* (20); *Rodríguez, D. 1382* (14), *1383* (13); *Roger, L. 15 Feb 1910* (13); *Rohmeder, E. 16 Feb 1942* (13), *19 Feb 1942* (13); *Romero-Castañeda, R. 3279* (2); *Romoleroux, K. & G. Peyre 5756* (2); *Romoleroux, K., D. Cárate & L.E. López 5355* (2); *Romoleroux, K. et al. 4642* (7a); *Roque, J. 559* (10 and 13), *560* (10), *4799* (9); *Roque, J. & C. Arana 3289* (20); *Roque, J. & K. Young 1221* (20); *Roque, J. et al. 538* (10); *Ruthsatz, B.J. 10533* (20), *10539* (10), *10549* (10), *10570* (13).

***S****ahonero (de), E.T. Apr 1973* (13); *Salgado, S. 686* (2); *Salinas, N. et al. 3294* (9); *Salles, C. 229* (10); *Salvador, F., S. Ríos & E. Arias 735* (20), *944* (20); *Sánchez, H. 351* (7a); *Sánchez, S. 36* (13), *44* (21); *Sánchez, R. 727* (2); *Sandoya-Sánchez, C.V., E. Gortaire & J. Irazábal 335* (7a); *Sarria, S. 591* (7a); *Sauvain, M. 827* (16), *828* (13); *Schlegel, F. 4751* (11); *Schneider, M. 1182* (2); *Schultes, R.E. & M. Villarreal 7955* (2); *Sklenář, P. 7314* (7a), *7328* (2), *7508* (2), *7528* (5), *7538* (6), *8175* (7a), *8369* (2), *9086* (2), *9125* (2), *9268* (2), *9269* (6), *9338* (6), *13132* (3), *14116* (3), *15820* (1); *Sklenář, P. & V. Kostečková 8-1* (2), *31-20* (2), *37-8* (7a), *48-1* (7a), *49-16* (2), *51-9* (2), *90-5* (3), *100-11* (2), *111-7* (2), *120-1* (2), *148-17* (2), *713* (7a), *1039* (2), *1274* (6); *Sklenář, P. & E. Rejzková 10707* (2); *Sklenář, P. & V. Sklenářová 2010* (7a), *2228* (6), *2556* (2), *2793* (6), *2810* (2), *2903* (2), *2941* (3), *3022* (3), *3099* (2), *3246* (2), *3403* (2), *3414* (3), *3418* (3), *3422* (2), *3424* (3), *3435* (2), *3538* (2), *3559* (7a), *3604* (2), *3632* (2), *3741* (3); *Sleumer, H. 3448* (17), *3452* (17), *3684* (13); *Sleumer, H. & F. Vervoorst 3015* (13); *Smeets, M. & M. Lind van Wijngaarden 736* (2); *Smith, D.N. 8258* (20), *11304* (20), *11323* (2); *Smith, D.N. & M. Buddensiek 11126* (20), *11208* (20); *Smith, D.N. & V. Cautivo 10290* (20); *Smith, D.N. & F. Escalona 10207* (20); *Smith, D.N. & R. Valencia 9963* (20); *Smith, D.N., M. Buddensiek & R. Valencia 11644* (20); *Smith, D.N., R. Valencia & A. Gonzales 9469* (8), *10700* (20); *Smith, D.N., R. Valencia & M. Torres 11900* (20); *Soderstrom, T.R. 1293* (2); *Sodiro, L. 61/3* (2); *Solomon, J.C. 9785* (20), *13210* (20); *Sparre, B. 8827* (13), *9734* (13); *Spruce, R. 6013* (4); *Stafford, D. 716* (22), *730* (9), *734* (21), *748* (16), *749* (8); *Stancik, D. 3381* (7a); *Steyermark, J.A. 53216* (2); *Stübel, M.A. 106* (10), *116* (10), *339b* (3); *Stuckert, T. 13110* (13); *Sturm, H. 115* (2); *Suárez, D., M. Chinchero & M. Cabascango 1325* (2), *1355* (7a).

***T****eillier, S. 3011* (16), *4058* (12), *4226* (12), *7740* (11); *Teillier, S. & A. Buben 6579* (10); *Teillier, S. & G. Mieres 5447* (11); *Tejada, C. 226* (9); *Torres, J.H. et al. 1633* (7a), *1663* (7a), *1944* (7a); *Tovar, O. 2560* (9), *2948* (20), *3386* (20), *3423* (16), *7187* (20), *7191* (16); *Tovar, O. & L. Constance 9194* (9 and 16 and 18); *Tovar, O., S. Rivas & Sáenz 7806* (18); *Tovar, O. et al. 9627* (8), *9666* (8), *9710* (18), *9842* (8); *Treiber de Espinosa, B. 101* (2); *Treviño, I. 761* (16); *Triana, J.J. 2803.1* (2); *Troll, C. 2076* (20), *3320* (11); *Tupayachi, A. 736* (16), *1515* (20); *Tutin, T.G. 1201* (21), *1203* (9).

***U****lloa, C. & D. Minga 1393* (2); *Ulloa, C., P.M. Jørgensen & X. Clavijo 1173* (4); *Ulloa, C. et al. 2416* (2), *2534* (4); *Uribe Uribe, L. 4492* (2), *6206* (2).

***V****alencia, R. & J.C. Matheus 395* (2); *Valles, H. & S. Chimbolema 373* (2); *Vargas, C. 4382* (21), *11222* (13), *11223* (9), *11871* (9), *13016* (13), *13620* (9), *19541* (21); *Vargas, C. & Santander 514* (20); *Vargas, H. & E. Narváez 2113* (6), *3103* (6), *3117* (6); *Vargas, H., E. Narváez & S. Orellana 2630* (7a), *2679* (2); *Vargas, H., E. Narváez & W. Quizhpe 1936* (2); *Vargas, H., J.C. Ronquillo & N. Granda 2837* (2), *2838* (2), *2869* (2), *2870* (7a); *Vargas, M.A. 77* (20); *Vargas, W.G. 4201* (7a), *8108* (7a), *15711* (7a), *18840* (7a), *19700* (7a), *21036* (7a), *22353* (2), *22382* (7a); *Vega, N. 1734* (21), *1744* (9), *1782* (20), *1785* (13); *Velayos, M., C. Aedo & C. Monge 11263* (11); *Venturi, S. 2964* (13), *4643* (13), *6254* (17), *6342* (14), *6646* (13), *6647* (14), *9289* (14); *Vervoorst, F. 749* (13); *Vilcapoma, G. 4461* (18), *5194* (21), *5540* (13), *6070* (20); *Villagrán, C. & M. Arroyo 4093* (11); *Villagrán, C., F. Hinojosa & C. Latorre 9167* (11), *9321* (12); *Villagrán, C. et al. 1175* (11).

***W****aechter, P. 27 Oct 1974* (13); *Weberbauer, A. 356* (16), *6628* (2), *Aug 1920* (20); *Weberling, F. 5891* (16); *Weibel, M. Jun 1976* (20); *Werdermann, E. 1164* (10); *Whymper, E. Mar 1880* (6); *Wood, J.R.I. 11255* (20), *20663* (20); *Wood, J.R.I. & C. Wendleburger 16400* (20).

***Y****ager, K. 4697* (16); *Yarupaitán, G. 1499b* (19), *1501* (16), *1688* (9), *1690* (16); *Yarupaitán, G. & J. Albán 954* (13), *1012* (21), *1063* (16), *1065* (13), *1067* (16); *Yarupaitán, G. & E. Salas 1482* (20); *Yarupaitán, G., E. Olivera & R. Canto 1355* (20).

***Z****apata, F. 66* (2); *Zárate, M. 2212* (13), *2216* (16), *2440* (20), *3770* (9); *Zeisek, V. 13161* (6); *Zenteno, F. 16389* (9), *16550* (13), *16686* (16), *17034* (9); *Zenteno, F., L. Moya & D. Villalba 16745* (22); *Zenteno, F., D. Villalba & L. Mamani 21274* (8), *21275* (9), *21277* (20); *Zenteno, F. et al. 16836* (13), *19280* (2).
